# Root Hair Development Is Suppressed by Long‐Term Mild Heat Through Down‐Regulation of *RHD6* and *RHD6‐like* Genes

**DOI:** 10.1111/pce.15563

**Published:** 2025-04-18

**Authors:** Gaigai Du, Xin Tian, Daan M. van den Brink, Jian Xu, Eric J. W. Visser, Ivo Rieu

**Affiliations:** ^1^ Department of Plant & Animal Biology, Radboud Institute for Biological and Environmental Sciences Radboud University Nijmegen The Netherlands

**Keywords:** cell fate, development, heat, initiation, *RHD6*, root hair

## Abstract

Roots located in the upper soil layers are prone to experiencing high temperatures. Despite their importance for water and nutrient absorption, little is known about the effect of high temperature on root hairs. Here, we found that exposure of *Arabidopsis thaliana* seedlings to long‐term mild heat suppressed root hair initiation. Epidermal patterning of hair and non‐hair cells was maintained, as observed with *GL2*‐ and *CPC*‐based marker genes, and the suppression was independent of the activity of GL2 and its upstream regulators. Instead, we found that expression of downstream *RHD6* and RHD6‐like bHLH transcription factor genes *RSL2* and *RSL4* was reduced and that overexpression of *RHD6* via an inducible transgene or ethylene treatment maintained the transcriptional expression of *RSL2* and *RSL4* and fully rescued the root hair phenotype. We conclude that GL2‐independent downregulation of *RHD6* and its homologues mediates the inhibition in root hair initiation under long‐term mild heat stress. This finding may contribute to the development of strategies for improving plant performance under high temperature.

## Summary statement

The suppression of *RHD6* and its homologues, independent of GL2, plays a crucial role in inhibiting root hair initiation in *Arabidopsis thaliana* when plants are subjected to prolonged mild heat stress.

## 1 Introduction

Root hairs are crucial structures in plants that improve water and nutrient uptake by increasing the root surface area contacting with soil (Bibikova and Gilroy 2002). Studies have shown that root hair traits and their response to abiotic stress are important for plant performance under unfavourable environmental conditions (Lynch 2019; Segal et al. 2008). Root hairs are produced from root epidermal cells, and their formation includes cell fate determination, bulge initiation and hair elongation (Ishida et al. 2008). The root epidermis consists of two distinct cell types, that is, root hair cells, also called trichoblasts, and non‐hair cells, or atrichoblasts, and these identities are determined by a position‐dependent signalling pattern (Grebe 2012). Epidermal cells located in the cleft between two underlying cortical cells (the ‘H’ cell position) are specified as hair cells, and epidermal cells located over a single cortical cell (the ‘N’ cell position) are specified as non‐hair cells (Schiefelbein et al. 2009). Once an epidermal cell has stopped elongating and root hair cell fate is determined, it expands asymmetrically on one side close to the basal end, leading to root hair initiation (Takatsuka and Ito 2020). This involves the establishment of root hair planar polarity contributed by the localization of Rho‐type GTPase proteins at the future initiation site (Denninger et al. 2019; Jones et al. 2002; Xu and Scheres 2005) and is followed by localized cell wall acidification, which induces the activation of expansins and xyloglucan endotransferases (*XETs*). Loosening of the cell wall then leads to local bulging upon which root hair elongation begins (Cho and Cosgrove 2002; Schiefelbein 2000; Vissenberg et al. 2001).

Root hair cell fate determination is the earliest event in root hair development. Previous studies have uncovered a network of transcriptional regulators responsible for root hair specification (Bruex et al. 2012; Schiefelbein et al. 2014). The central regulator is a MYB‐bHLH‐WD40 (MBW) complex containing an R2R3‐type MYB protein encoded by *WEREWOLF* (*WER*) (Lee and Schiefelbein 1999), a WD40‐repeat protein encoded by *TRANSPARENT TESTA GLABRA1* (*TTG1*) (Galway et al. 1994) and a bHLH protein encoded by the functionally redundant *GLABRA3* or *ENHANCER OF GLABRA3* (*GL3/EGL3*) genes (Bernhardt et al. 2003, 2005). This MBW complex preferentially accumulates in non‐hair cells where it directly promotes transcription of *GLABRA*2 (*GL2*), which activates non‐hair cell differentiation genes and inhibits root hair promoting genes such as *ROOT HAIR DEFECTIVE* 6 (*RHD6*) (Di Cristina et al. 1996; Masucci and Schiefelbein 1994; Masucci et al. 1996). The complex also induces the expression of the R3‐type MYB protein CAPRICE (CPC), which, together with TRIPTYCHON (TRY) and ENHANCER OF TRY AND CPC 1 (ETC1), moves to the neighbouring cell. Here, they suppress the expression of *GL2* by competing with WER for binding to GL3/EGL3 bHLHs (Lee and Schiefelbein 2002). As a result, the expression of *RHD6* and other root hair promoting bHLH genes, such as *RHD6*‐*LIKE1* (*RSL1*), *RSL2* and *RSL4*, is induced to promote root hair initiation and growth (Bruex et al. 2012; Masucci and Schiefelbein 1994; Menand et al. 2007).

Hormones have also been shown to be involved in root hair development, among which auxin and ethylene have been studied well. Ethylene can induce the ectopic production of root hairs by activating transcription factors *EIN3* and its homologue *EIL1*. EIN3/EIL1 competes with GL3 for interaction with TTG1 and thus prevents *GL2* activation. EIN3 also activates *RSL4* directly, through physical interaction with RHD6 (Feng et al. 2017; Qiu et al. 2021). Auxin has been shown to promote root hair growth synergistically with ethylene but seems to function only at the level of *RSL4* and not affect upstream cell fate determination. For example, *AUXIN RESPONSE FACTORs*, such as *ARF5*, *ARF7*, *ARF8* and *ARF19*, can activate expression of *RSL2* and *RSL4* by binding to the promoter region, promoting root hair elongation (Bhosale et al. 2018; Mangano et al. 2017; Vissenberg et al. 2020). Recent studies also indicated a role for cytokinin in root hair development. Cytokinin promotes root hair initiation and elongation in part by inducing the expression of *ZINC FINGER PROTEIN5* (*ZFP5*), which in turn promotes the expression of *CPC* (An et al. 2012). Root hair elongation is also stimulated by treatment with the synthetic strigolactone GR24, and this is dependent on ethylene synthesis (Kapulnik et al. 2011; Vissenberg et al. 2020). Jasmonate has also been reported to stimulate root hair growth, doing so by inducing the expression of *RHD6*/*RSL1* and *EIN3*/*EIL1* (Han et al. 2020). By contrast, it was found that brassinosteroids negatively affect root hair initiation. BR‐deficiency caused downregulation of *WER* and *GL2* and thus promoted root hair cell fate, whereas fewer root hairs were produced when BR signalling was enhanced (Vissenberg et al. 2020; Wei and Li 2016). Finally, ABA inhibits root hair growth by inducing *OBP4* to suppress *RSL2* expression (Rymen et al. 2017; Shibata and Sugimoto 2019). Together, these studies indicate that multiple hormones interactively regulate root hair development.

As crucial structures for water and nutrient absorption, the effects of abiotic stresses on root hair formation have been studied extensively. It was found that drought‐tolerant barley genotypes had more and longer root hairs than sensitive genotypes (He et al. 2015). In rice, *WOX11*‐overexpressing plants had increased root hair numbers and length and enhanced drought tolerance, while *wox11* mutant with fewer and shorter root hairs were drought sensitive (Cheng et al. 2016). The same relation has been found for different types of nutrient stress, including deficiencies in phosphate, iron, manganese and zinc (Janes et al. 2018; Powell et al. 1988; Wei Yang et al. 2008; Zhang et al. 2018). Root hair density and length are also stimulated in hypoxia, potentially as a response to reduced nutrient absorption (Kumar et al. 2020). By contrast to the above, under salt stress, root hair numbers and length showed significant decreases, thought to be a strategy to limit the uptake of toxic ions (Robin et al. 2016; Wang et al. 2008, 2013).

Roots, especially in the top soil layer, are also exposed to fluctuations in temperature. Depending on the soil characteristics, the soil may reach temperature equal to or even higher than air temperature (Munns et al. 2010; Pogačar et al. 2022). Despite being one of the main changing environmental factors globally, the effect of high temperature on root hair development has not been investigated yet. Increased transpiration is a well‐known acclimation response to heat, so it may be hypothesized that root hair density would increase to improve water uptake. In this study, we examined the responses of root hair development to heat stress and, contrary to expectations, found that root hair formation was inhibited. We used gene expression analyses, mutant analyses and hormone treatments to understand the molecular physiological basis of this response.

## 2 Results

### 2.1 Characterization of Root Hair Phenotypes in Response to High Temperature

**Figure 1:**
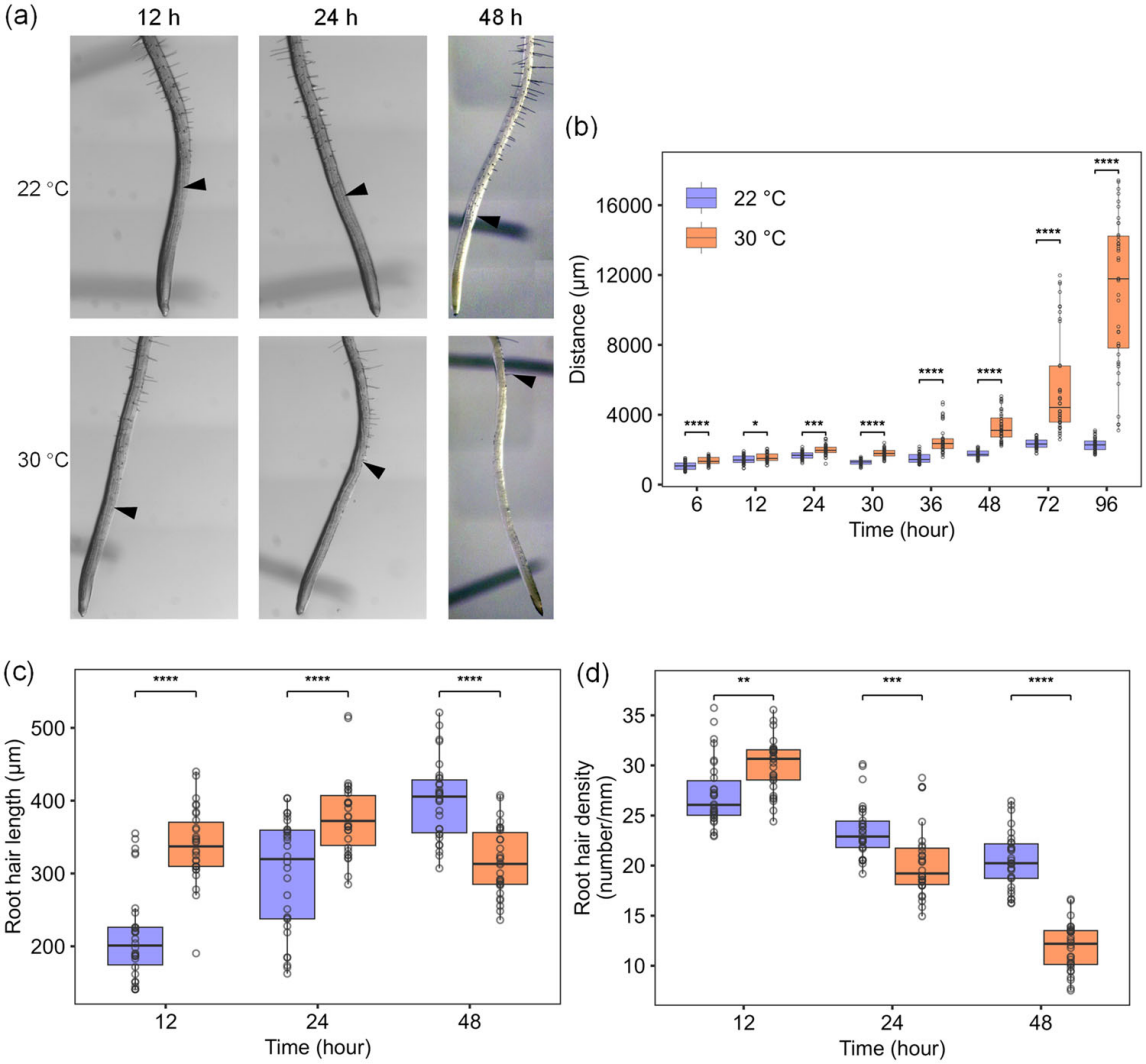
The effect of high temperature on root hair characteristics. (a) Example images of roots from control and high temperature at different time points. Arrow heads indicate the first visible root hair bulge. Bars = 200 μm. (b) Quantification of the distance between root tip and the first hair bulge (*n* = 40 seedlings from 8 plates; Supporting Information S1: Figure S1a). (c) Quantification of root hair length at different time points (*n* = 30 seedlings from 6 plates; Supporting Information S1: Figure S1b). (d) Quantification of root hair density at different time points after the start of heat exposure (*n* = 30 seedlings from 6 plates; Supporting Information S1: Figure S1c). The boxes represent the 25th to 75th percentiles, the upper and lower whisker, the maximum and minimum values, respectively, the outliers are represented by dots. The horizontal lines in the box represent the median. Differences were analyzed by one‐way ANOVA followed by LSD post hoc test (*p < 0.05; **p < 0.01; ***p < 0.001; ****p < 0.0001).

To explore how root hair development in Arabidopsis responds to high temperature, 5‐day‐old seedlings were subjected to heat stress at 30°C, and root hair phenotypes were observed for up to 4 days. The distance from the root tip to the first root hair bulge was measured as an indication of the timing of root hair initiation. Upon exposure to high temperature, this distance increased gradually (Figure 1a,b and Supporting Information S1: Figure S1a). After 30 h at 30°C, the distance was increased 1.5‐fold compared to the 22°C control, and it increased further to twofold after 48 h. After that, the distance kept increasing linearly while it remained stable in control. Root hair length was increased after 12 and 24 h of exposure but not anymore after 48 h (Figure 1c and Supporting Information S1: Figure S1b). Notably, very few non‐fully elongated root hairs were visible at 48 h and after, indicating that upon exposure to high temperature, root hair initiation was gradually delayed and finally inhibited completely. This pattern was reflected in root hair density (measured in a 4 mm root region above the first detected bulge/hair), which, after a small increase in the first 12 h, decreased markedly after 24 and 48 h of high temperature (Figure 1d and Supporting Information S1: Figure S1c).

### 2.2 High Temperature Delays the Switch of Root Cells From Elongation to Epidermal Differentiation

**Figure 2:**
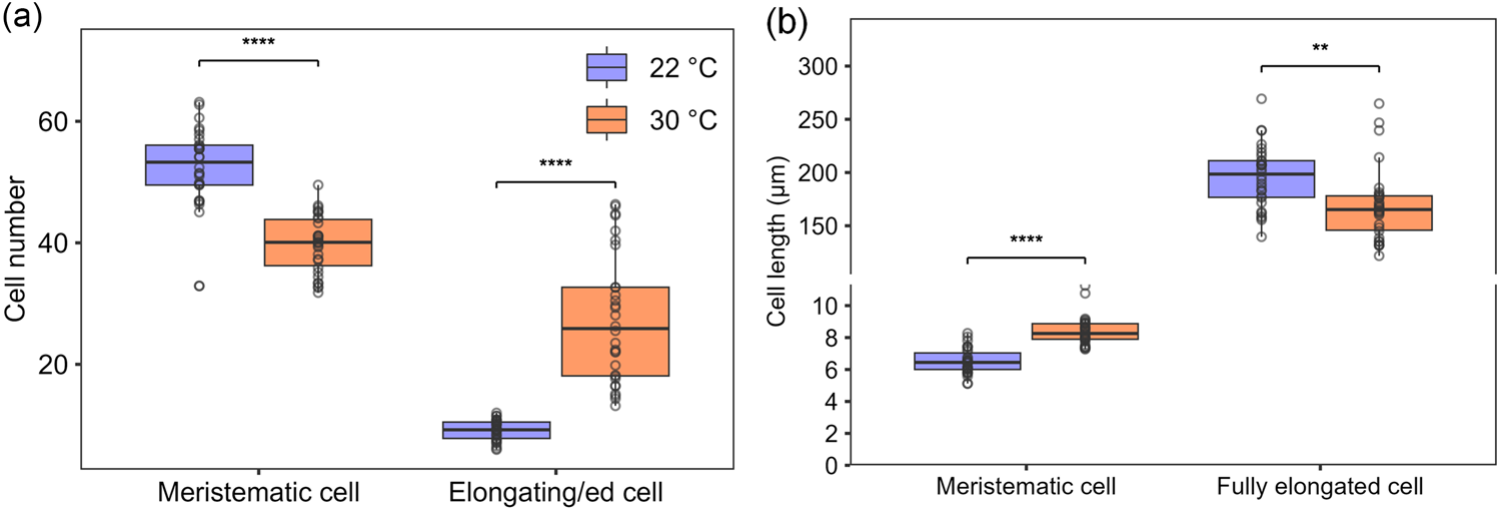
Effect of high temperature on cell size and number in the root tip. (a) Number of cells in the meristem and elongation zones (*n* = 30–33 seedlings from 6 plates; Supporting Information S2: Figure S2a). (b) Length of meristematic and fully elongated cells (*n* = 30–33 seedlings from 6 plates; Supporting Information S2: Figure S2b). Values indicate the mean ± SD. Differences were analyzed by one‐way ANOVA followed by LSD post hoc test (**p < 0.01; ****p < 0.0001). [Color figure can be viewed at wileyonlinelibrary.com]

To clarify the reason for the increase in the distance from the root tip to the first root hair at high temperature, we hypothesized that either there were more cells present before the root hair differentiation zone or the cells were longer. We found that the size of the meristematic zone was slightly reduced, which was due to a lower number of cells (Figure 2a,b and Supporting Information S2: Figure S2a,b), in agreement with slower root growth rate (Supporting Information S2: Figure S2c). By contrast, we found a strong increase in the number of cortical cells in the elongation zone, that is, before the appearance of the first root hair bulge, which was of almost the same maximum length in either condition (Figure 2a,b and Supporting Information S2: Figure S2a,b). Taken together, it can be concluded that the increased distance from the root tip to the first root hair bulge was caused by a delay in the switch of root epidermal cells from cell elongation to differentiation.

### 2.3 Root Hair Specification Genes Are Expressed Differentially at High Temperature

**Figure 3:**
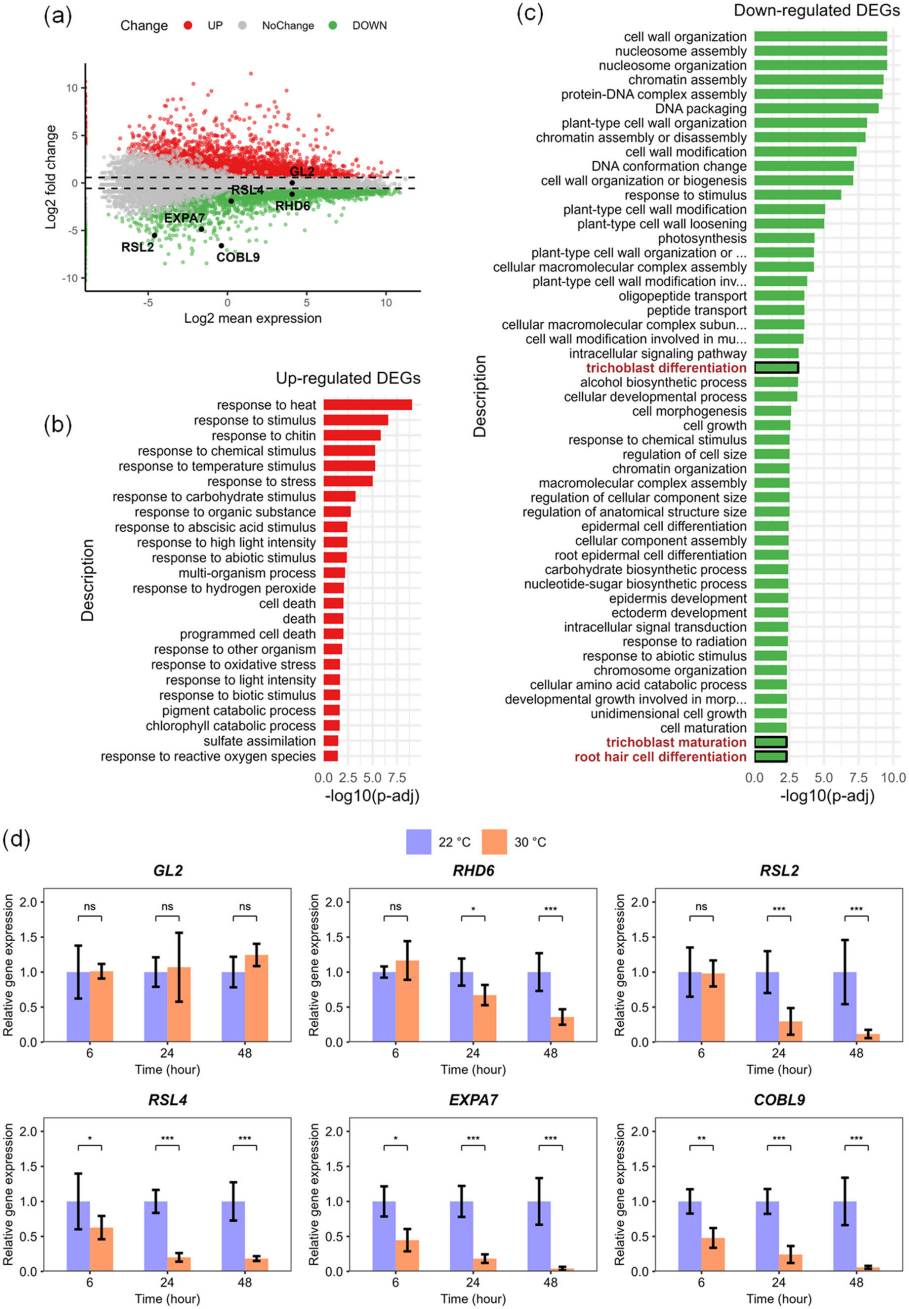
Transcriptomic response to high temperature in the root tip. (a) MA plot showing the DEGs according to fold change and significance. Grey dots indicate genes with no significant difference in expression, red dots indicate upregulated differential expression genes, green dots indicate downregulated differential expression genes. (b) GO enrichment analysis of DEGs from RNA‐seq analysis that were upregulated under heat stress. (c) GO enrichment analysis of DEGs that were downregulated under heat stress. The lengths of the bars indicate the −log_10_(*p* value) of the enrichment. The annotations highlighted in red represent the GO terms of our interest. (d) qRT‐PCR analysis of root hair specification genes at various time points after start of the high temperature treatment (*n* = 4 plates with 10 root tips each, with 3 technical replicates each). Data were normalized against control treatment for each time point, and values represent the mean ± SD. Differences were analyzed by one‐way ANOVA followed by LSD post hoc test (ns, not significantly different; *p < 0.05; **p < 0.01; ***p < 0.001). [Color figure can be viewed at wileyonlinelibrary.com]

To explore the mechanism by which high temperature inhibits root hair initiation, we evaluated RNA‐seq data from 1 mm root tips of 7‐day‐old seedlings exposed to control or high temperature for 48 h (Supporting Information S6: Table S1). According to the Gene Ontology (GO) functional classification with all the differentially expressed genes (DEGs) (Figure 3a,b), downregulated genes were enriched for various root hair, cell wall and cell growth‐associated terms (Figure 3c). Indeed, a number of key genes involved in trichoblast‐atrichoblast epidermal patterning, which is the earliest event in root hair development, were affected (Table 1). Transcription factor genes involved in the specification of non‐root hair identity, like *WEREWOLF* (*WER*), *GLABRA3* (*GL3*), *ENHANCER OF GLABRA3* (*EGL3*), were expressed lower in the heat, as were their targets *CAPRICE* (*CPC*) and *ENHANCER OF TRY AND CPC1* (*ETC1*), encoding mobile hair cell identity proteins. Neither of these, however, affected the expression of the downstream master regulator *GL2*, which was similar in control and high temperature. Interestingly, however, the root hair identity genes that are normally repressed by GL2, that is, *ROOT HAIR DEFECTIVE*6 (*RHD6*), *RHD6*‐*LIKE4* (*RSL4*) and *RSL2*, were downregulated at high temperature. The latter suggests increased GL2 activity in root epidermal cells or *GL2*‐independent downregulation of these genes.

**Table 1:** High temperature response of key regulators of root hair specification revealed by RNA‐seq analysis.

Gene name	Gene_ID	log_2_FoldChange	*P*_adj_	Category^a^
*WER*	AT5G14750	−0.56	0.000	N
*GL2*	AT1G79840	0.01	0.889	N
*TTG1*	AT5G24520	0.11	0.238	N
*GL3*	AT5G41315	−1.43	0.000	N
*EGL3*	AT1G63650	−1.40	0.000	N
*CPC*	AT2G46410	−0.97	0.000	H
*TRY*	AT5G53200	0.01	0.981	H
*ETC1*	AT1G01380	−4.70	0.000	H
*RHD6*	AT1G66470	−1.19	0.000	H
*RSL2*	AT4G33880	−5.53	0.000	H
*RSL4*	AT1G27740	−1.91	0.000	H

^a^ *N* refers to genes involved in non‐root‐hair cell identity, H to those involved in root‐hair cell identity.

Based on the RNA‐seq analysis, we analyzed the expression of *GL2*, *RHD6*, *RSL4* and *RSL2*, involved in root hair cell fate determination, and *EXPA7* and *COBL9*, involved in root hair tip growth, at different time points after high‐temperature exposure, using qRT‐PCR (Figure 3d). Consistent with the RNA‐seq analysis, *GL2* showed no significant difference in expression levels between control and high temperature, indicating *GL2* was not responsive to heat stress at the transcriptional level. *RHD6* and *RSL2* showed significant and sharp decreases from 24 h after the start of high‐temperature treatment, and for *RSL4*, this trend was visible from 6 h onwards. *EXPANSIN A7* (*EXPA7*) and *COBRA*‐*LIKE 9* (*COBL9*) were also downregulated, which was already detectable after 6 h of treatment.

### 2.4 Inhibition of Root Hair Development Is Independent of GL2 Activity

**Figure 4:**
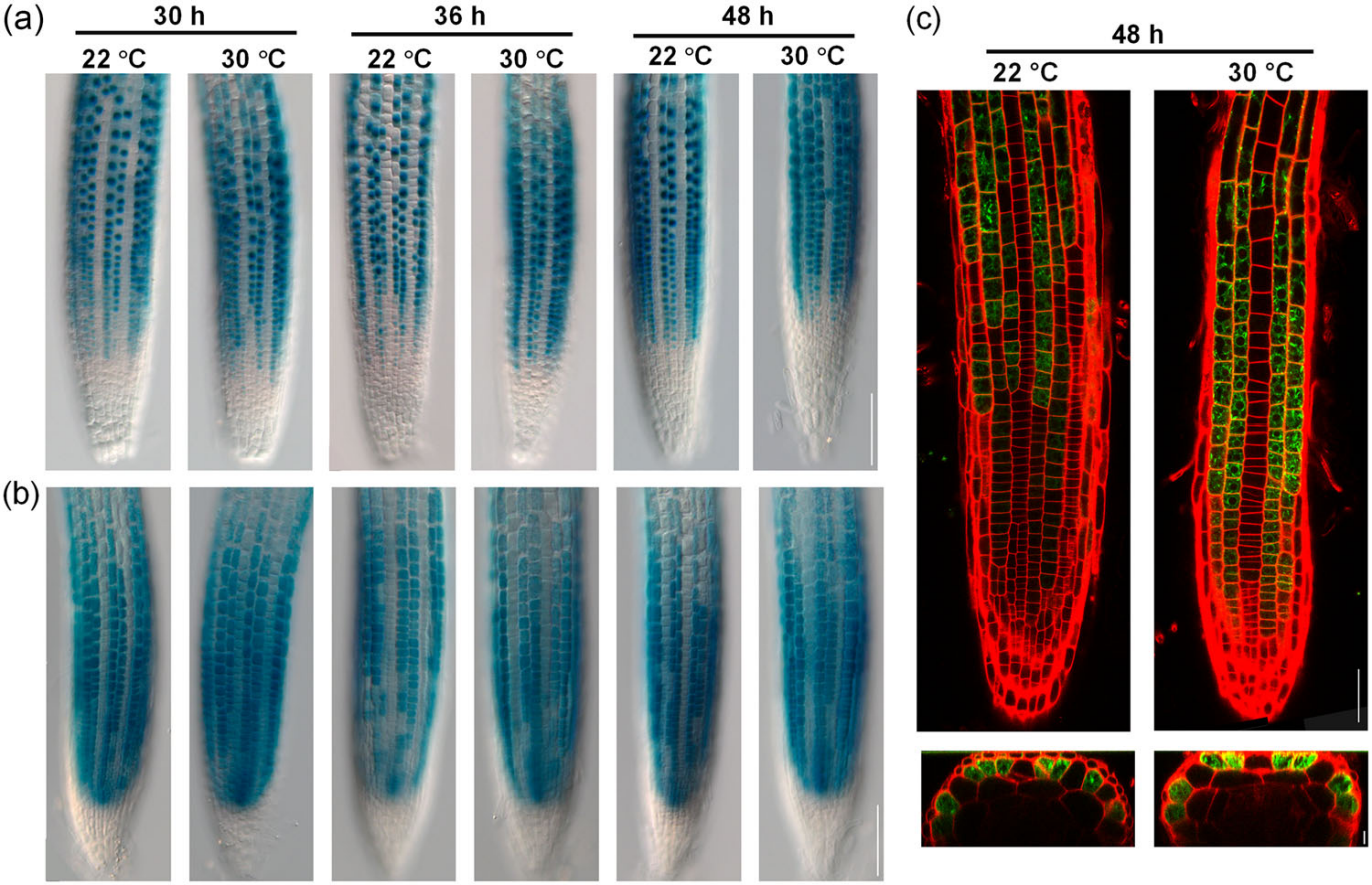
*GL2* and *CPC* promoter activity at high temperature. Expression pattern of *GL2::GUS* (a) and *CPC::GUS* (b) in H‐position cells. (c) Reconstructed transverse sections of the roots of plants expressing *GL2::GFP*, grown at control or high temperature for 48 h. Bars = 100 μm (a, b), 50 μm (c upper images) and 10 μm (c bottom images). [Color figure can be viewed at wileyonlinelibrary.com]

**Figure 5:**
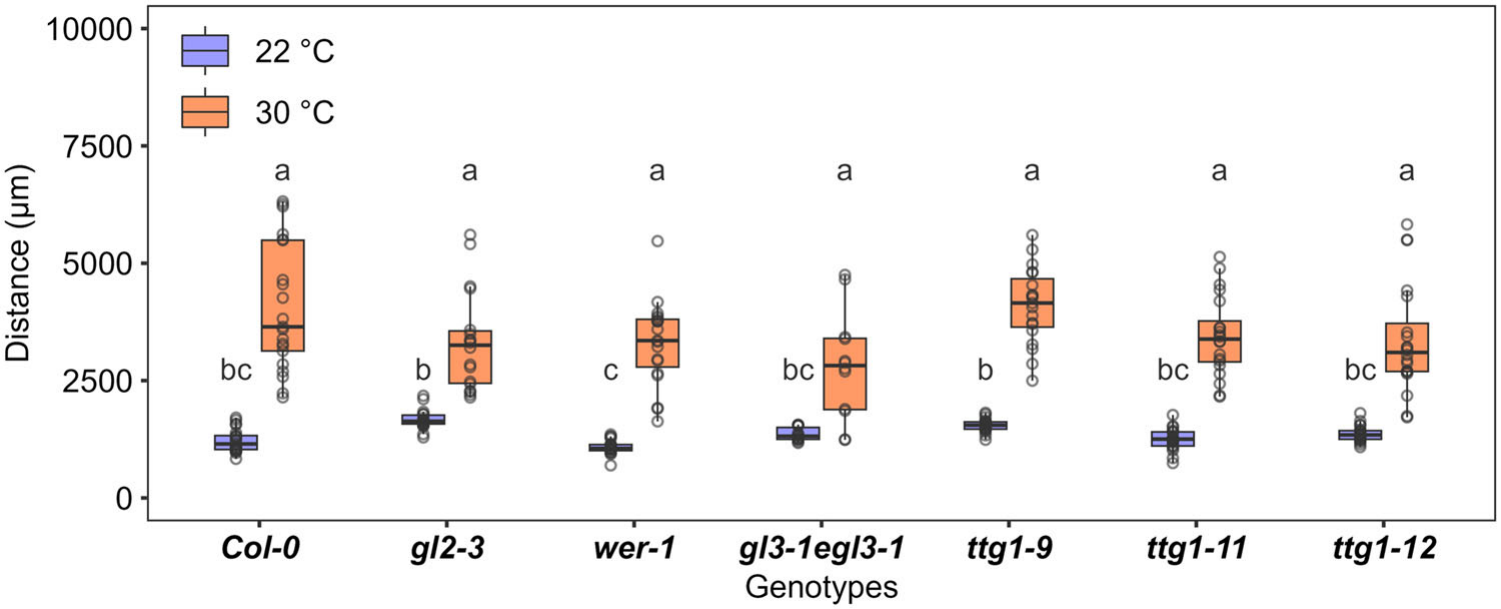
Root hair initiation in mutants of *GL2* and its upstream regulators at high temperature. Quantification of the distance between the root tip and the first root hair bulge of different genotypes grown under control and high temperature (*n* = 14–20 seedlings from 2 plates; Supporting Information S3: Figure S3). Differences were analyzed by one‐way ANOVA followed by Tukey post hoc test. Different letters indicate a significant difference (*p* < 0.05). [Color figure can be viewed at wileyonlinelibrary.com]

Previous studies reported that *GL2* and *CPC* are strictly expressed in non‐hair epidermal cells. To investigate whether the position‐dependent pattern of epidermis cell types is maintained at high temperature, we examined transgenic lines expressing β‐glucuronidase (GUS) under the control of the *GL2* or *CPC* promoter. GUS staining showed that after 48 h at high temperature, the root epidermis maintained a clear cell type pattern consisting of one to three GUS‐positive non‐hair cell files next to one GUS‐negative hair cell file (Figure 4a,b). We also investigated the position of *GL2* expressing epidermal cells with respect to cortical cells by imaging *GL2::GFP* in the root and digitally reconstructing transverse sections from stacked images. GFP expression was observed in epidermal cells overlaying a single cortical cell and never in those located over the cleft between two cortical cells (Figure 4c), indicating that root hair cell fate specification was maintained correctly. To further confirm that inhibition of root hair development upon high‐temperature exposure was not due to altered activity of *GL2* and its upstream regulators, we tested the high‐temperature response of hairy genotypes *gl2‐3*, *wer‐1*, *gl3‐1 egl3‐1*, *ttg1‐9*, *ttg1‐11* and *ttg1‐12*. All mutants showed a dramatic increase in the distance from the root tip to the first root hair under high temperature, indistinguishable from the wild type (Figure 5 and Supporting Information S3: Figure S3).

### 2.5 Overexpression of RHD6 Rescues Root Hair Initiation

**Figure 6:**
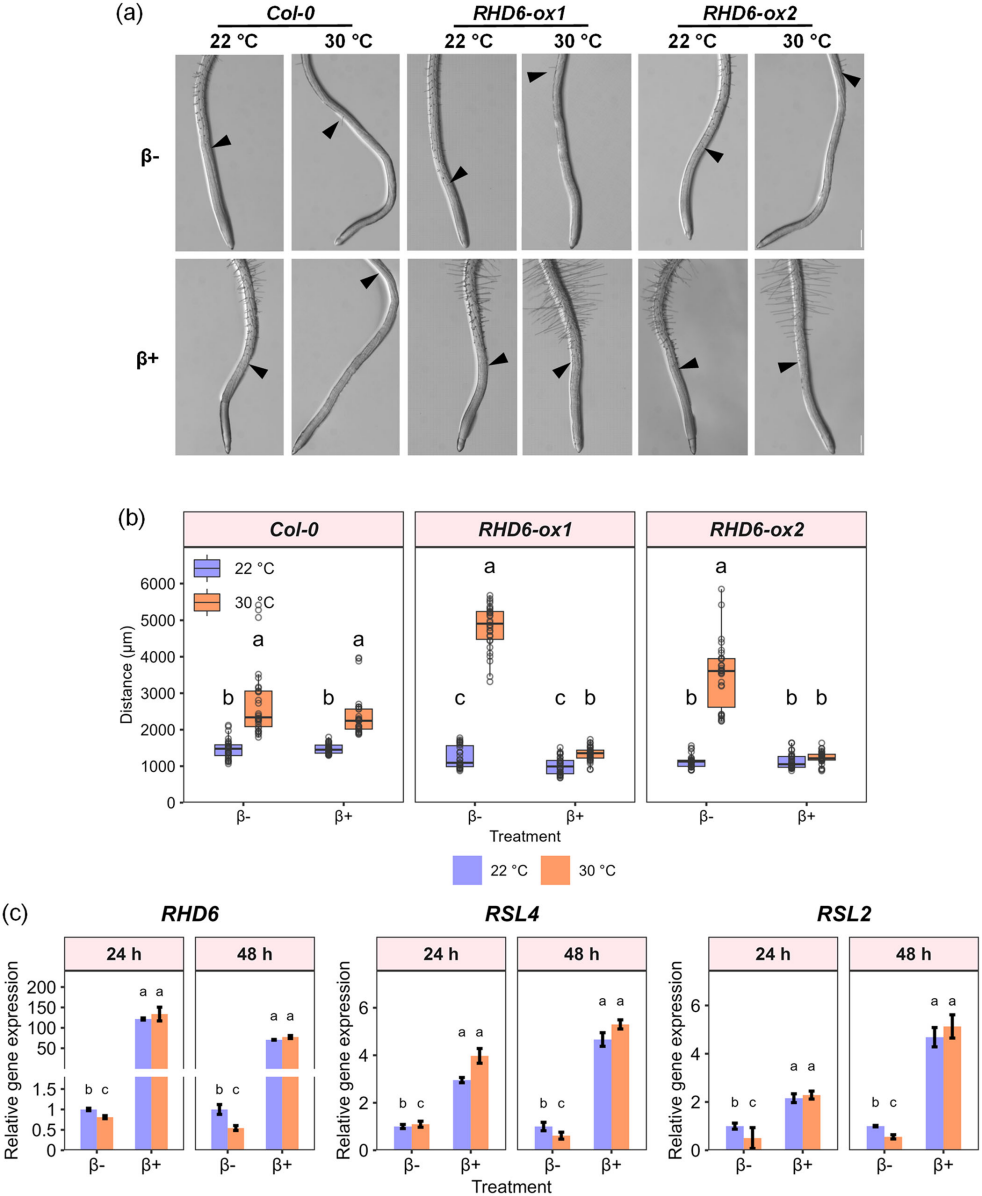
The effect of *RHD6* overexpression on root hair initiation at high temperature. (a) Representative images of root hairs in wild type (*Col‐0*) and two inducible *RHD6*‐overexpression lines, with and without β‐estradiol treatment indicated by β+ and β−, respectively, at control and high temperature. Bars = 300 μm. (b) Quantification of the distance from the root tip to the first root hair bulge in the overexpression lines (*n* = 30–31 seedlings from 6 plates; Supporting Information S4: Figure S4). Differences were analyzed by one‐way ANOVA followed by Tukey post hoc test. Different letters indicate a significant difference (*p* < 0.05). (c) Expression analysis of root hair initiation genes in *RHD6* overexpression roots (*n* = 4 plates with 10 root tips each, with 3 technical replicates each). Values indicate the mean ± SD. Differences at 24 and 48 h were analyzed separately by one‐way ANOVA followed by Tukey post hoc test. Different letters indicate a significant difference (*p* < 0.05). [Color figure can be viewed at wileyonlinelibrary.com]

Given that *GL2* and its upstream root hair cell fate pathway were not involved in the suppression of root hair development at high temperature, we hypothesized that it was caused by the defective expression of the downstream *bHLH* transcription factors *RHD6*, *RSL4* and *RSL2*. To this end, we examined the phenotypes of inducible overexpression lines of *RHD6* under high‐temperature conditions (Figure 6a,b and Supporting Information S4: Figure S4). While the estradiol induction treatment did not affect the root hair response to high temperature in the wild‐type line, induced overexpression of *RHD6* fully rescued root hair development in two independent transgenic lines, making them insensitive to high temperature. Gene expression analysis by qRT‐PCR indicated that the high‐temperature insensitivity of root hairs of *RHD6* overexpression plants was accompanied by sustained transcription levels of the downstream RHD6‐like bHLH transcription factor genes, *RSL4* and *RSL2* (Figure 6c).

### 2.6 Ethylene Treatment Rescues Root Hair Development at High Temperature

**Figure 7:**
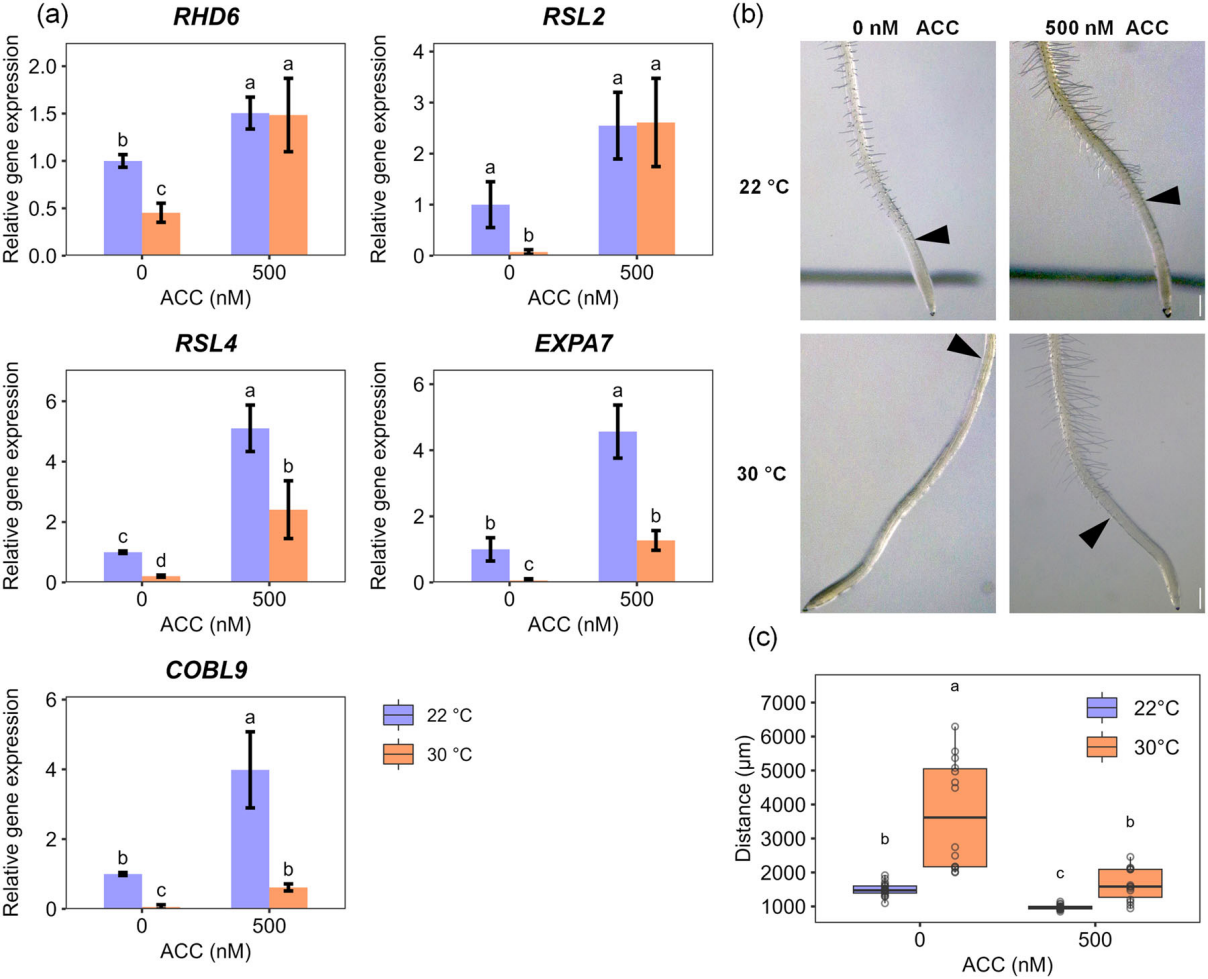
The effect of exogenous ACC on root hair development at high temperature. Five‐day‐old seedlings were treated with 500 nM ACC and high temperature for 48 h. (a) Expression of root hair developmental genes upon ACC treatment. Values indicate the mean ± SD (*n* = 4 plates with 10 root tips each, with 3 technical replicates each). (b) Representative roots from control and ACC treatment. Bars = 300 μm. (c) Quantification of the distance from root tip to the first root hair (*n* = 20 seedlings from 2 plates; Supporting Information S5: Figure S5). Differences were analyzed by one‐way ANOVA followed by Tukey post hoc test. Different letters indicate a significant difference (*p* ≤ 0.05). [Color figure can be viewed at wileyonlinelibrary.com]

Expression of *RHD6* and *RSL4* is induced by ethylene as a result of the co‐activation of *RHD6* by *EIN3*‐type transcription factors. To further confirm that low expression of *RHD6* and *RSL4* limited root hair development at high temperature, we applied ACC to seedlings growing at control and high temperature. In the presence of 500 nM ACC, *RHD6*, *RSL4*, *RSL2* as well as the downstream genes *EXPA7* and *COBL9* were all strongly induced. Expression levels of *RSL4*, *EXPA7* and *COBL9* were still heat sensitive but remained at or above the level found in untreated seedlings at control temperature (Figure 7a). This molecular phenotype was accompanied by a nearly full rescue of root hair initiation at high temperature (Figure 7b,c and Supporting Information S5: Figure S5).

## 3 Discussion

Climate change results in increased frequency, duration and severity of heatwaves, which not only affect the temperature of the air, and thus plant shoots, but also that of the top soil layer, and thus plant roots (Munns et al. 2010; Pogačar et al. 2022). Despite the crucial role of root hairs in water and nutrient physiology, little is known about the effect of high temperature on root hair formation. We found that exposure to long‐term mild heat suppressed root hair initiation and that this was due to a GL2‐independent downregulation of *RHD6* and *RHD6‐like* genes.

Previous research has indicated that root hair initiation and growth are highly responsive to stress factors, such as low water or low nutrient availability, and that the response can often be regarded as adaptive, that is, positively affecting tolerance to the stress factor (Cheng et al. 2016; Hanif and Davies 1998; He et al. 2015; Ma et al. 2001; Powell et al. 1988; Wang et al. 2008). During periods of high temperature, plants benefit from sustained or even enhanced stomatal opening, as it allows for the maintenance of photosynthesis and transpirational tissue cooling. As such, it may be hypothesized that high temperature would have a stimulatory effect on root water uptake capacity and thus lead to increased root hair numbers and root hair length, allowing for better exploration of the soil. Unexpectedly, we found that upon exposure to high temperature, root hair initiation was suppressed. While the distance between the root tip and the first root hair bulge increased mildly in the first 2 days of exposure, indicative of delayed initiation, from 48 h after the start of the high temperature onwards, it increased linearly, and at a rate very comparable to the root elongation rate in that period of treatment (several mm per day). Together with the fact that no immature root hairs were observed anymore at these later time points, it can be concluded that root hair initiation was fully inhibited after 48 h. Concomitantly, mature root hair length had decreased at 48 h of high‐temperature exposure. Also, in cotton, high temperature decreased root hair density and length significantly (Fan et al. 2022). Different from these findings, and in line with the hypothesis, Mohammad (2000) reported increased proximity of the hair zone to the root tip upon simultaneous application of water deficit and high‐temperature stress in trifoliate orange. However, this was assumed to be caused by severe inhibition in root cell growth rate under the heat and water deficit combination, rather than through stimulation of root hair initiation. Taken together, hair initiation and elongation are particularly sensitive to long‐term high temperature, and we suggest that the observed response reflects damage rather than physiological adaptation.

Interestingly, the response of root hair density and root hair length was opposite in the first 12 h after the start of the stress. We propose that this first phase reflects the increased speed of development and cellular growth at a higher temperature, according to the thermal time concept (Parent et al. 2019), or an interaction between early stress response and growth pathways, for example, due to hormonal changes such as ethylene and jasmonate signalling (Feng et al. 2017; Han et al. 2023; Zhu et al. 2006). Either way, the positive effect quickly becomes masked by accumulated heat damage.

We found that high temperature led to downregulation of the MBW complex genes *WER*, *GL3* and *EGL3* and MBW targets *CPC1* and *ETC1* but not *GL2*. The apparent uncoupling of MBW and *GL2* gene expression may be explained by differences between target genes in sensitivity to MBW level. However, this still does not explain the opposite, hairless outcome, as observed here, which would require ectopic upregulation of *GL2* in the H position (Won et al. 2009). Such an upregulation was not detected in either, the transcript analyses and the *GL2* promoter‐marker analysis. Furthermore, the knockout of *GL2* did not rescue root hair initiation upon high‐temperature exposure. Thus, the molecular reason for the inhibition of root hair development by high temperature seems to lie downstream of epidermal patterning and GL2 activity.

A notable deviation from the normal epidermal cell differentiation programme was the lower expression of *RHD6*, *RSL2* and *RSL4* at high temperature, despite the absence of upregulation of *GL2*, their major upstream negative regulator. *RHD6* and related bHLH transcription factors control the initiation and growth of root hairs, making them clear candidates for mediation of the high‐temperature effect (Bruex et al. 2012). Indeed, we found that induced overexpression of *RHD6* fully complemented the high‐temperature effect. Furthermore, ethylene treatment, which also led to higher expression of the RHD6‐type bHLH genes, complemented the phenotype, too. Upregulation of *RHD6* and *RSL4* by ethylene has been reported before (Feng et al. 2017). This effect is different from the reported induction of ectopic root hairs by ethylene treatment in normal conditions caused by reduced expression of *GL2* (Qiu et al. 2021), which was not observed here. The reason for the downregulation of RHD6‐type bHLH genes at high temperatures remains to be determined. It is unlikely that the heat effect is mediated by modulation of endogenous ethylene biosynthesis or signalling, because ethylene‐insensitive genotypes still produce root hairs (Feng et al. 2017; Harkey et al. 2018) and well‐known ethylene‐responsive genes with functions other than root hair development (e.g., *PIN1*, *PIN2*, *PIN4*, *AUX1* and various ERF, e.g., *ERF1*/*ERF104*; Harkey et al. 2018; Illgen et al. 2020; Růžička et al. 2007) were not significantly downregulated in our RNA‐seq data. High temperature could affect accessibility of the promoters of RHD6‐type genes, reduce stability of their transcripts or influence the expression or activity of other upstream regulators. The C2H2‐type zinc finger protein ZINC FINGER PROTEIN1 (ZP1) mediates GL2 effects on RHD6‐type gene expression (Han et al. 2020), but we did not observe upregulation of ZP1 in our RNA‐seq data (Supporting Information S7: Table S2). Similarly, we did not find induction of the trihelix transcription factor GT2‐LIKE1 (GTL1), which inhibits RHD6 protein activity (Shibata et al. 2022), or downregulation of AT‐HOOK MOTIF NUCLEAR LOCALIZED17 (AHL17) and AHL28, which have been shown to stimulate RHD6 protein activity (Zeng et al. 2023). However, Jin et al. (2023) identified *ABRE‐BINDING FACTOR1* (*ABF1*), *ABF3* and *ABF4* as negative regulators of *RHD6* activity and root hair development during salt stress, and we found that the transcript level of *ABF1* rose markedly in root tips subjected to heat stress, about 2.4‐fold. This warrants further investigation into its potential role in regulating *RHD6* and downstream root hair‐specific genes at high temperature.

In conclusion, high temperature suppresses root hair development through downregulation of the RHD6‐like bHLH transcription factor gene expression, via a mechanism that does not require GL2 activity. This part of the root hair development pathway seems a focal point for output modulation, as the effects of several other stresses on root hair development also take place through these genes. For example, suppression of root hair development at excessive nutrient levels is mediated by *RHD6* downregulation, whereas stimulation of root hair formation in response to cold acts via binding of a lncRNA to the *RHD6* locus and in response to phosphate starvation via overexpression of the primary ethylene transcription factor *EIN3* (Moison et al. 2021; Song et al. 2016; Shibata et al. 2022). This insight may support the future development of strategies to mitigate the negative effect of heatwaves and other stresses on plant performance.

## 4 Materials and Methods

### 4.1 Plant Materials and Growth Conditions

*Arabidopsis thaliana* ecotype *Columbia‐0* (*Col‐0*) was used as the wild‐type reference. *GL2::GUS*, *wer‐1*, *ttg‐9*, *ttg‐11* and *ttg‐12* were obtained from Martin Hülskamp's lab (Rishmawi et al. 2014, 2018). *gl3‐1egl3‐1* was obtained from Wolfgang Schmidt's lab (Bernhardt et al. 2003). *CPC::GUS* was obtained from Ikram Blilou's lab (Hassan et al. 2010). *gl2‐3* (N665830) (Wang et al. 2010), *GL2::GFP* (N66491), *RHD6‐ox1* (N2104357) and *RHD6‐ox2* (N2104358) TRANSPLANTA lines were derived from NASC stock centre (Nottingham Arabidopsis Stock Centre; http://arabidopsis.info/).

Arabidopsis seeds were surface sterilized in 75% ethanol, 0.05% tween‐20 for 12 min. After that, seeds were washed three times with 95% ethanol, dried on filter paper and finally soaked in sterilized Milli‐Q water for use. Seeds were sown on solid agar medium containing 0.5X Murashige and Skoog salts with Gamborg B5 (Duchefa, Haarlem, The Netherlands), 1% (w/v) sucrose, 0.05% (w/v) MES monohydrate and 1% (w/v) plant agar (Duchefa), pH5.7. Seeds on plates were stratified for 2 days at 4°C in the dark and then transferred to a climate chamber to grow vertically with a long‐day period (16 h light/8 h dark, fluorescent tubes) at 22°C. For heat treatment, plates with 5‐day‐old seedlings were transferred to 30°C. For the inducible *RHD6* overexpression lines, 5‐day‐old seedlings were transferred to a medium containing 10 μM estradiol and further incubated at 22°C or 30°C. As controls, 5‐day‐old seedlings were transferred to medium without supplements.

### 4.2 Phenotyping of Root Hair Under Heat Stress

For the time course of root hair phenotypes in response to heat stress, the roots were imaged with wide‐field stereomicroscopy (Leica MZFLIII; Leica Microsystems, Wetzlar, Germany). The distance from the root tip to the first visible root hair bulge was measured. Root hair density and root hair length were analyzed in a 4 mm region distal to the first visible root hair bulge. Image J (https://imagej.nih.gov/ij/) was used to measure root hair length, taking the average length of the 20 longest root hairs. For each sample type, 10 individual roots were analyzed.

### 4.3 Microscopy and Image Analysis

Fluorescence imaging was performed using confocal microscopy (Leica SP8x) with a 20× water immersion objective using 1× zoom, pinhole 1 AU, 1024 × 1024 pixel scanning field, 600 Hz scanning speed, 2× line average as settings. GFP was imaged after staining roots of 7‐day‐old seedlings in 10 μg mL^−1^ propidium iodide (PI) for cell wall visualization. GFP was excited with a 488 nm laser, and emission light was collected between 495 and 530 nm. PI was exited with a 561 nm laser, and emission light was collected between 570 and 650 nm. The cell number was determined as from the cortical cell closest to the QC to the first elongating cell (the end of apical meristem) or to the differentiated cell next to the epidermal cell bearing the first root hair. The fully elongated cell length was measured as the average cell length of 10 fully elongated cells.

### 4.4 RNA‐Seq Analysis

Five‐day‐old Col‐0 seedlings were grown at 22°C and 30°C for 48 h. Root tips of 1 mm were collected using a scalpel and immediately frozen in liquid nitrogen. Three biological replicates were used, and each replicate derived from 50 individual seedlings from 5 plates. Samples were grinded with a tissue lyser (Westburg, Utrecht, The Netherlands) at 25 Hz for 2 min 45 s. Total RNA was isolated using RNeasy Micro Kit (Qiagen, Hilden, Germany) according to the manufacturer's protocol. The RNA was dissolved in 20 μL of RNase‐free water. The quality and purity of the RNA was analyzed with a NanoDrop 1000 spectrophotometer (Thermo Fisher Scientific, Waltham, MA) to ensure that the A260/A280 and A260/A230 ratios were around 2.0 and the samples were separated on a 1% agarose gel to confirm the quality and the integrity of RNA. The quality of RNA samples was further examined with the RNA 6000 Nano reagent kit combined with a 2100 Bioanalyzer (Agilent Technologies, Santa Clara, CA). All samples had an integrity number greater than 7.0 and were considered of sufficient quality for RNA‐Sequencing. mRNA was first purified from the total RNA using poly‐T oligo‐attached beads. Then, the first strand of cDNA was synthesized using random hexamer primers, and the second strand of cDNA was synthesized with dUTP to generate a directional library. Qubit and real‐time PCR were used for library quantification and Agilent Bioanalyzer was used for detecting size distribution of the sequencing libraries. Cluster generation was performed according to the manufacturer's instructions. After that, the libraries were sequenced on the NovaSeq. 6000 platform (Illumina, San Diego, CA). Gene expression levels were estimated as RPKM. Differential expression analysis was performed using the DESeq. 2R package. Genes with *P*_adj_ < 0.05 and |log_2_foldchange| ≥ 0.58 were regarded as DEGs. DEGs were annotated to GO biological processes, molecular functions, and cellular components, and GO‐enrichment analysis was done with the ClusterProfiler R package. GO terms with *P*_adj_ < 0.05 were considered significantly enriched among DEGs.

### 4.5 GUS‐Staining Assay

Seedlings were incubated in GUS staining solution (0.1 M potassium ferricyanide, 50 mM potassium ferrocyanide, 0.5 M EDTA, 0.2 M sodium phosphate buffer pH 7.0, 10% Triton X‐100, 200 mM X‐Gluc) at 37°C until the blue staining colours showed significant differences visually or became very dark in different treatments, which was after 24 h for *GL2::GUS* and 3 h for *CPC::GUS*. The reaction was terminated by transferring the stained seedlings to the revised Hoyer's solution. Roots were mounted and cleared with revised Hoyer's solution (chloral hydrate:glycerol:water = 8:2:1) and then imaged using differential interference contrast microscopy.

### 4.6 qRT‐PCR Experiments

One‐mm‐long root tips were dissected and frozen immediately in liquid nitrogen, with 40 individual seedlings pooled per sample and 4 independent replicates. Samples were grinded completely with a tissue lyser (Westburg) at 25 Hz for 2 min 45 s. RNA was extracted using the RNeasy Micro Kit (Qiagen) according to the manufacturer's protocol. The RNA was dissolved in 20 μL of RNase‐free water. Samples were checked with a NanoDrop 1000 spectrophotometer (Thermo Fisher Scientific), to ensure that the A260/A280 and A260/A230 ratios were around 2.0. The purity and the integrity of RNA samples were also checked by electrophoresis on 1% agarose gel. 500 ng of RNA was used for reverse transcription using the iScript cDNA Synthesis Kit (Bio‐Rad, Hercules, CA) according to the manufacturer's protocol. qRT‐PCR samples contained 5 μL iQ SYBR Green Supermix, 500 nM each of forward and reverse primers, and 100 ng of cDNA, and reactions were performed on a real‐time PCR system (Bio‐Rad CFX 96; 95°C for 30 s, 40 cycles of 95°C for 5 s and 60°C for 30 s). Melt curve analysis was performed for every qRT‐PCR experiment to ensure the specificity of the reaction. Primers were designed using Primer Blast in NCBI (Supporting Information S7: Table S2), and *UBC19* and *EIF4A3* were used for the normalization of the expression of target genes.

### 4.7 Statistical Analysis

All data, except for cell number and cell length, were log‐transformed to correct for heterogeneity of variance. Two‐way ANOVA and one‐way ANOVA followed by LSD or Tukey post hoc test were applied to test for significant differences (*p* < 0.05). The factor batch number was never significant. Analyses were done at the plate level, and because no plate effects were observed, also at seedling level. All statistical analyses were performed with SPSS v20 (IBM, Armonk, NY).

### 4.8 Accession Numbers

Sequence data used in this article can be found in The Arabidopsis Information Resource (https://www.arabidopsis.org/index.jsp) under the following accession numbers: AT3G20060 (UBC19), AT3G19760 (EIF4A3), AT5G49270 (COBL9), AT1G12560 (EXPA7), AT1G79840 (GL2), AT1G66470 (RHD6), AT4G33880 (RSL2) and AT1G27740 (RSL4).

## Conflicts of Interest

The authors declare no conflicts of interest.

## Supporting information


**FIGURE S1** The effect of high temperature on root hair characteristics. (a) Quantification of distance between root tip and the first hair bulge (n = 8 plates with 5 seedlings each). (b) Quantification of root hair length at different time points (n = 6 plates with 5 seedlings each). (c) Quantification of root hair density at different time points after start of heat exposure (n = 6 plates with 5 seedlings each). In coding of the sample types, B refers to biological replicate, P refers to plate number. The boxes represent the 25th to 75th percentiles, the upper and lower whisker the maximum and minimum values respectively, the outliers are represented by dots. The horizontal lines in the box represent the median. Two‐way ANOVA: P (Temp), P (Time) and P (Temp x Time) all < 0.0001. Differences were analyzed by one‐way ANOVA followed by LSD post hoc test (*, p < 0.05; **, p < 0.01; ***, p < 0.001; ****, p < 0.0001).

**FIGURE S2** Effect of high temperature on cell size and number in the root tip and PR (Primary Root) growth rate of seedlings. (a) Number of cells in the meristem and elongation zones (n = 6 plates with 30‐33 seedlings in total). Two‐way ANOVA: P (Temp) < 0.05, P (Cell type) and P (Cell type x Temp) all < 0.0001. (b) Length of meristematic and fully elongated cells (n = 6 plates with 30‐33 seedlings in total). Values indicate the mean ± SD. Two‐way ANOVA: P (Temp) > 0.05, P (Cell type) and P (Cell type x Temp) all < 0.0001. (c) Time‐course measurement of PR growth rate in plants grown at 22°Cand 30°C (mean ± SD, n = 9 plates from 52 seedlings). In coding of the sample types, B refers to biological replicate, P refers to plate number. Differences were analyzed by one‐way ANOVA followed by LSD post hoc test (**, p < 0.01; ***, p < 0.001; ****, p < 0.0001).

**FIGURE S3** Root hair initiation in mutants of *GL2* and its upstream regulators at high temperature. Quantification of the distance between the root tip and the first root hair bulge of different genotypes grown under control and high temperature (n = 2 plates with 14‐20 seedlings in total). In coding of the sample types, B refers to biological replicate, P refers to plate number. Two‐way ANOVA: P (Temp) < 0.0001, P (Genotype) and P (Genotype x Temp) > 0.05. Differences were analyzed by one‐way ANOVA followed by Tukey post hoc test. Different letters indicate significant difference (p < 0.05).

**FIGURE S4** The effect of *RHD6* overexpression on root hair initiation at high temperature. Quantification of the distance from the root tip to the first root hair bulge in the overexpression lines (n = 6 plates with 30‐31 seedlings in total). In coding of the sample types, B refers to biological replicate, P refers to plate number. Two‐way ANOVA: P (Genotype), P (Temp), P (Treatment) and P (All interaction) all < 0.0001. Differences were analyzed by one‐way ANOVA followed by Tukey post hoc test. Different letters indicate significant difference (p < 0.05).

**FIGURE S5** The effect of exogenous ACC on root hair development at high temperature. Five‐day‐old seedlings were treated with 500 nM ACC and high temperature for 48 h. Quantification of the distance from root tip to the first root hair (n = 2 plates with 20 seedlings in total). In coding of the sample types, B refers to biological replicate, P refers to plate number. Two‐way ANOVA: P (Treatment) and P (Temp) < 0.0001, P (Treatment x Temp) < 0.001. Differences were analyzed by one‐way ANOVA followed by Tukey post hoc test. Different letters indicate significant difference (P≤ 0.05).

Supplemental Table S1 Gene expression levels of DEGs in root tips of seedlings grown at 22 and 30°C for 48 h.

**Supplemental Table S2** Sequence of primers used for qRT‐PCR.

## Data Availability

The data that support the findings of this study are openly available in GSE254973 at https://www.ncbi.nlm.nih.gov/geo/query/acc.cgi?acc=GSE254973.
