# Supplementary figures and images for: Root Hair Development Is Suppressed by Long‐Term Mild Heat Through Down‐Regulation of RHD6 and RHD6‐like Genes
Source: Plant Cell Environ. 2025 Apr 18;48(8):5861–73. doi: 10.1111/pce.15563 (PMC12223709; doi:10.1111/pce.15563)

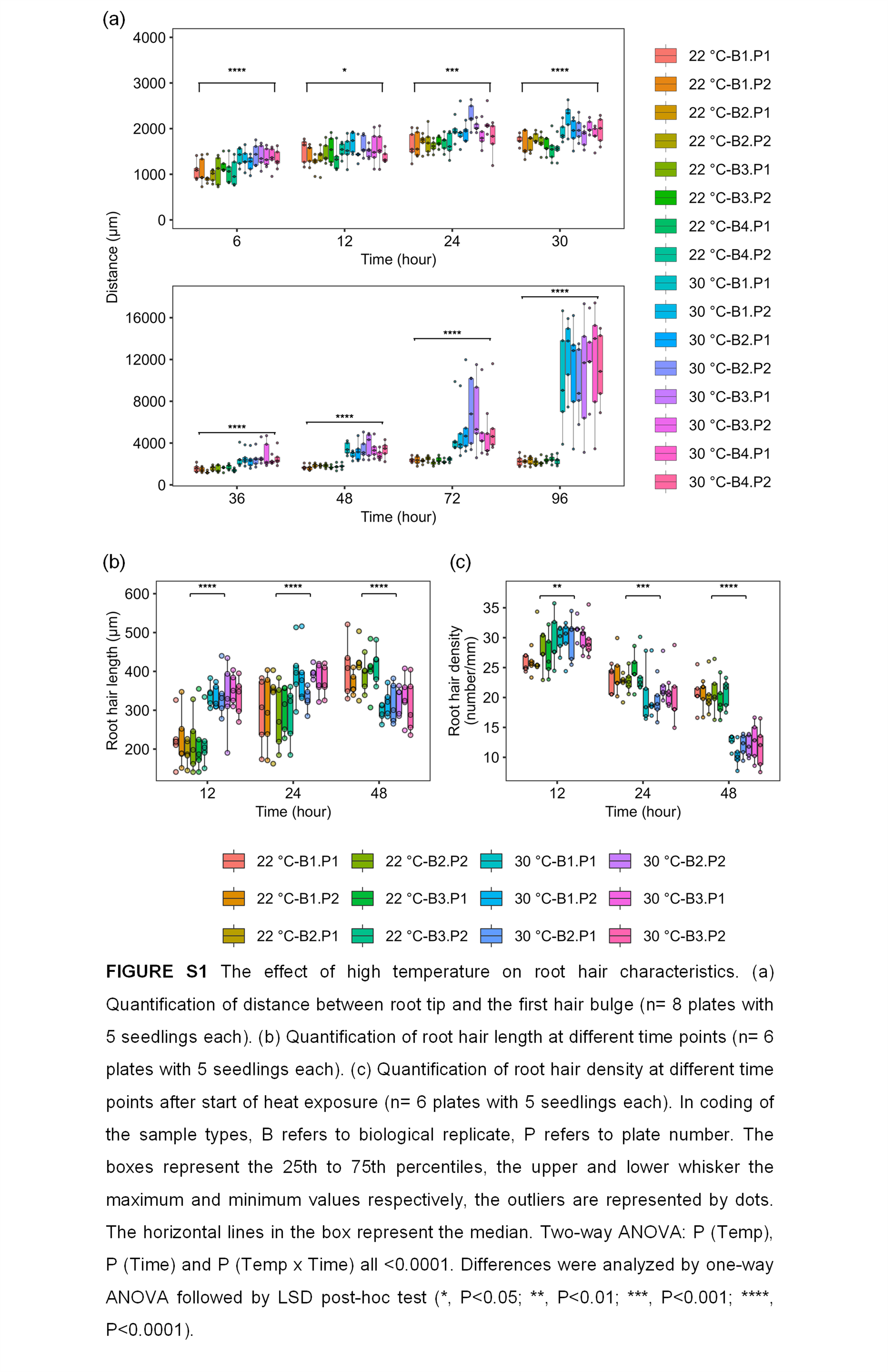

Supplement: Supplementary file 1 — FIGURE S1 The effect of high temperature on root hair characteristics. (a) Quantification of distance between root tip and the first hair bulge (n = 8 plates with 5 seedlings each). (b) Quantification of root hair length at different time points (n = 6 plates with 5 seedlings each). (c) Quantification of root hair density at different time points after start of heat exposure (n = 6 plates with 5 seedlings each). In coding of the sample types, B refers to biological replicate, P refers to plate number. The boxes represent the 25th to 75th percentiles, the upper and lower whisker the maximum and minimum values respectively, the outliers are represented by dots. The horizontal lines in the box represent the median. Two‐way ANOVA: P (Temp), P (Time) and P (Temp x Time) all < 0.0001. Differences were analyzed by one‐way ANOVA followed by LSD post hoc test (*, p < 0.05; **, p < 0.01; ***, p < 0.001; ****, p < 0.0001). [file PCE-48-5861-s005.png]

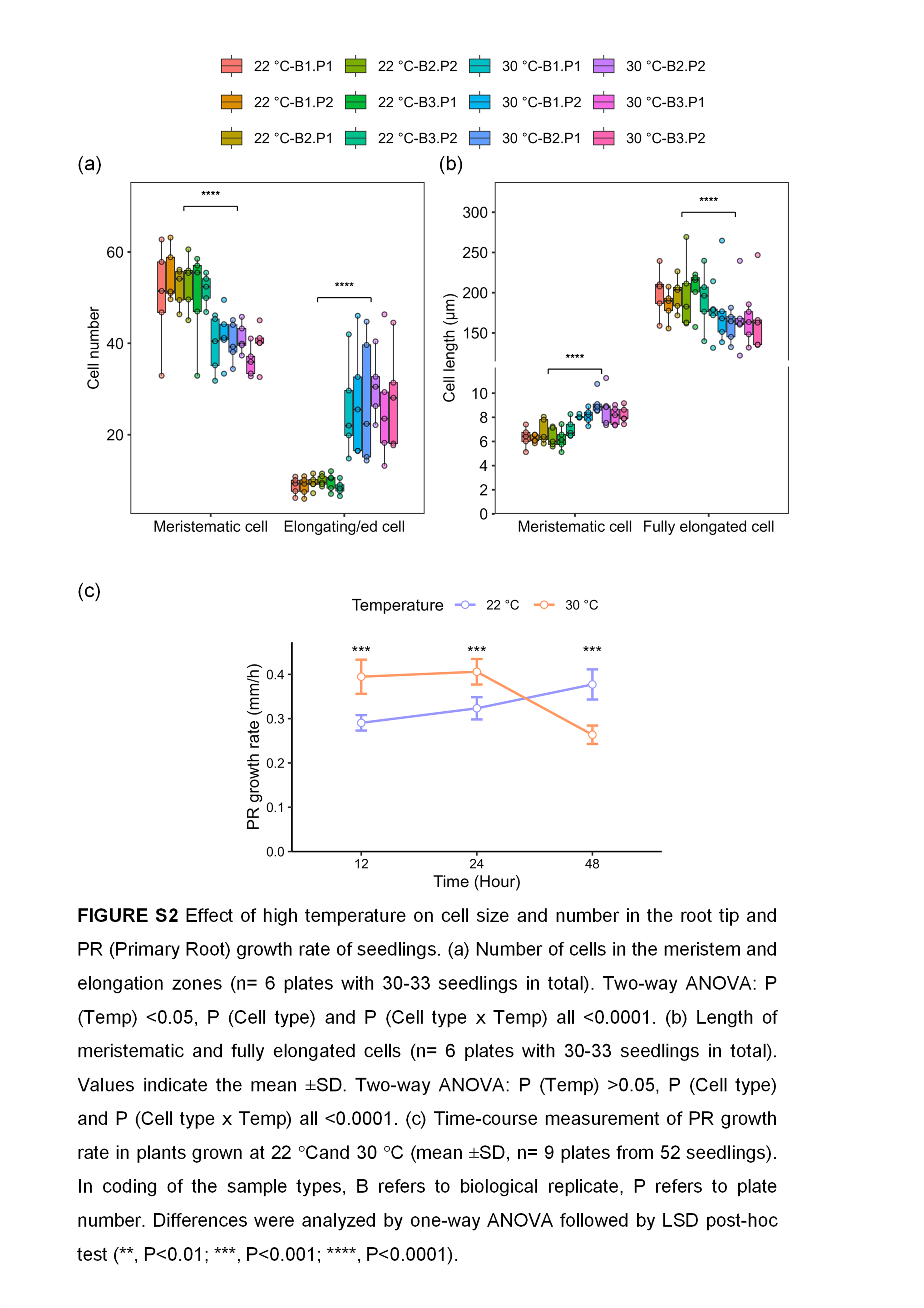

Supplement: Supplementary file 2 — FIGURE S2 Effect of high temperature on cell size and number in the root tip and PR (Primary Root) growth rate of seedlings. (a) Number of cells in the meristem and elongation zones (n = 6 plates with 30‐33 seedlings in total). Two‐way ANOVA: P (Temp) < 0.05, P (Cell type) and P (Cell type x Temp) all < 0.0001. (b) Length of meristematic and fully elongated cells (n = 6 plates with 30‐33 seedlings in total). Values indicate the mean ± SD. Two‐way ANOVA: P (Temp) > 0.05, P (Cell type) and P (Cell type x Temp) all < 0.0001. (c) Time‐course measurement of PR growth rate in plants grown at 22°Cand 30°C (mean ± SD, n = 9 plates from 52 seedlings). In coding of the sample types, B refers to biological replicate, P refers to plate number. Differences were analyzed by one‐way ANOVA followed by LSD post hoc test (**, p < 0.01; ***, p < 0.001; ****, p < 0.0001). [file PCE-48-5861-s001.png]

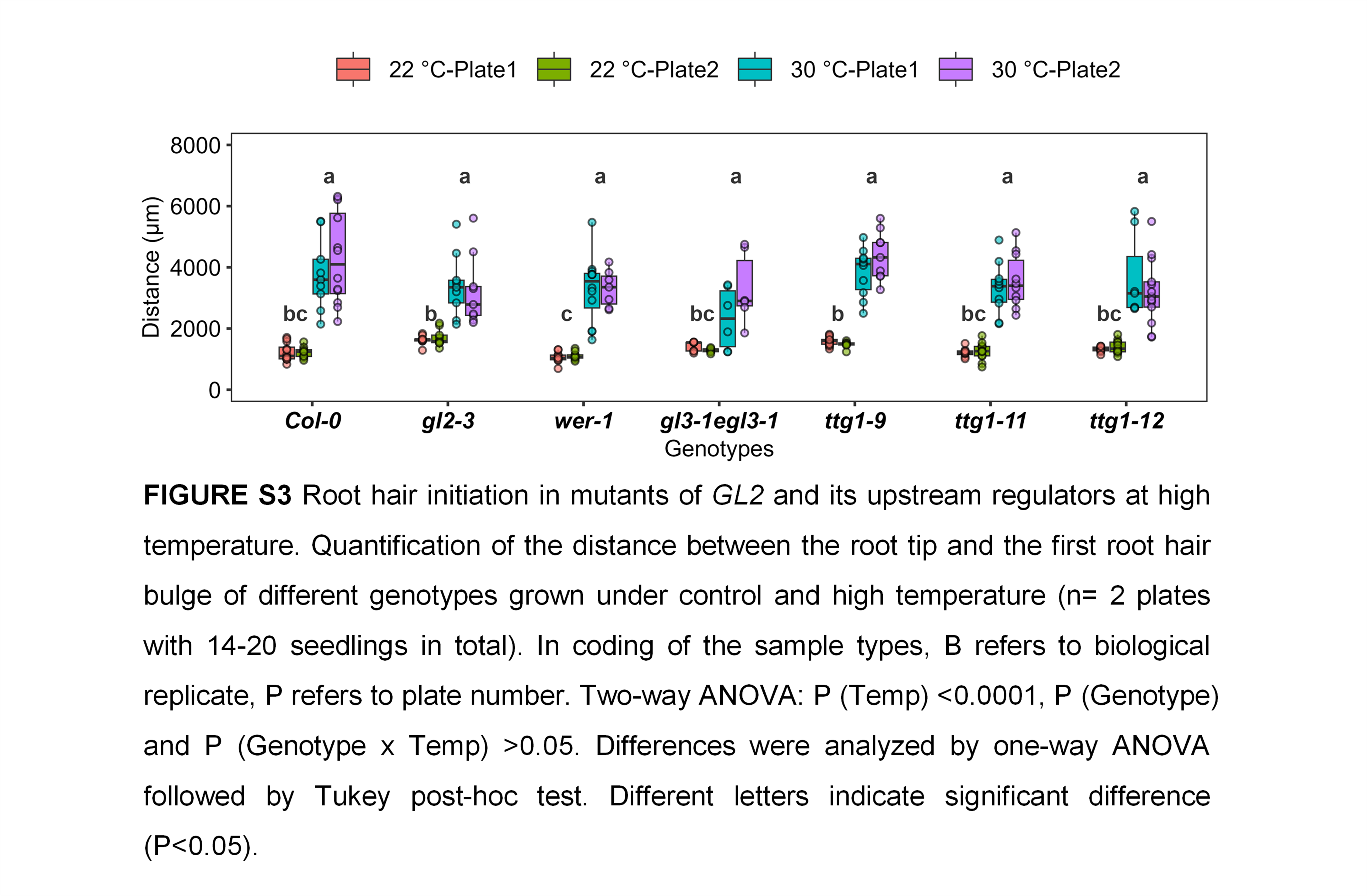

Supplement: Supplementary file 3 — FIGURE S3 Root hair initiation in mutants of GL2 and its upstream regulators at high temperature. Quantification of the distance between the root tip and the first root hair bulge of different genotypes grown under control and high temperature (n = 2 plates with 14‐20 seedlings in total). In coding of the sample types, B refers to biological replicate, P refers to plate number. Two‐way ANOVA: P (Temp) < 0.0001, P (Genotype) and P (Genotype x Temp) > 0.05. Differences were analyzed by one‐way ANOVA followed by Tukey post hoc test. Different letters indicate significant difference (p < 0.05). [file PCE-48-5861-s006.png]

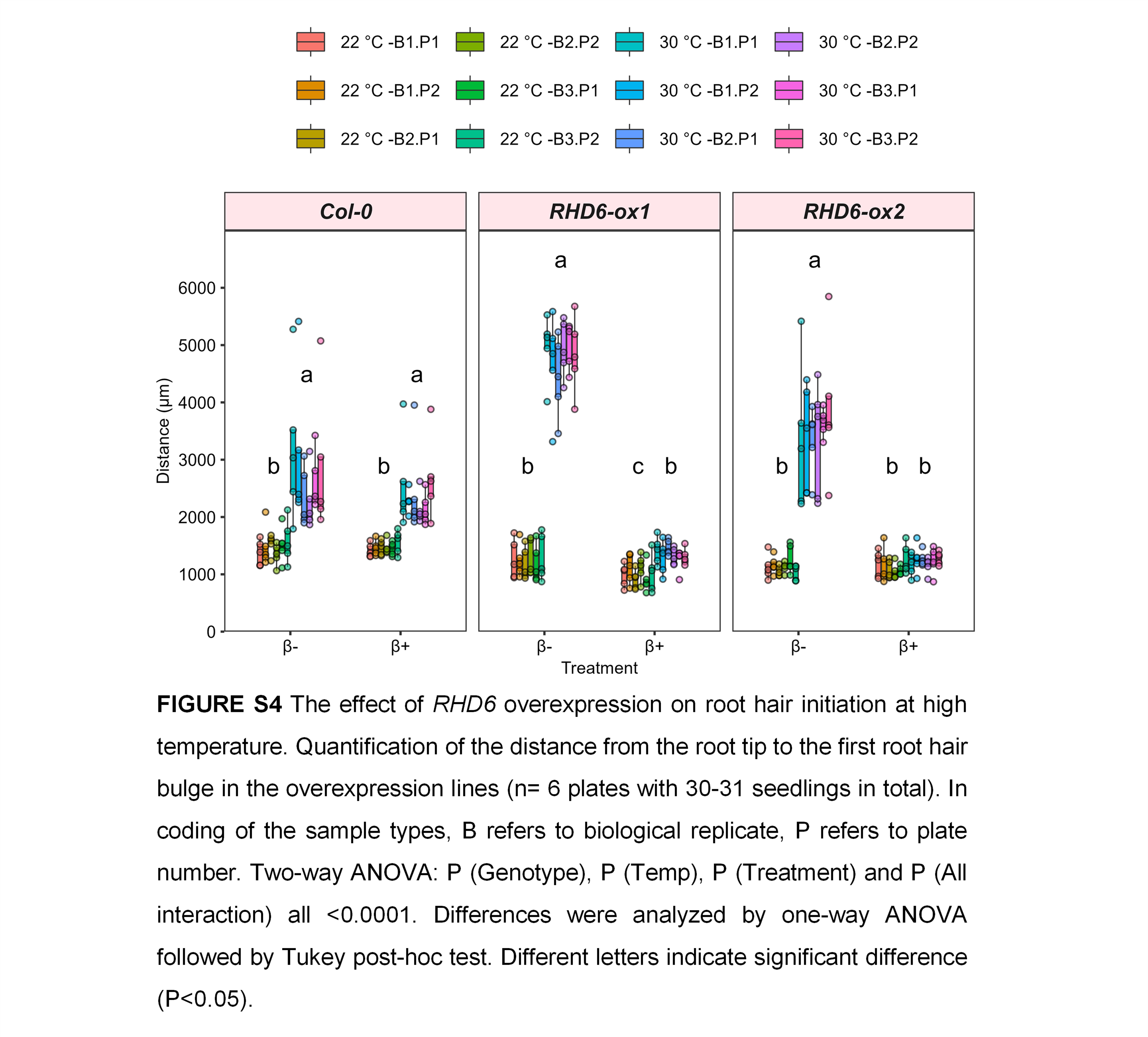

Supplement: Supplementary file 4 — FIGURE S4 The effect of RHD6 overexpression on root hair initiation at high temperature. Quantification of the distance from the root tip to the first root hair bulge in the overexpression lines (n = 6 plates with 30‐31 seedlings in total). In coding of the sample types, B refers to biological replicate, P refers to plate number. Two‐way ANOVA: P (Genotype), P (Temp), P (Treatment) and P (All interaction) all < 0.0001. Differences were analyzed by one‐way ANOVA followed by Tukey post hoc test. Different letters indicate significant difference (p < 0.05). [file PCE-48-5861-s003.png]

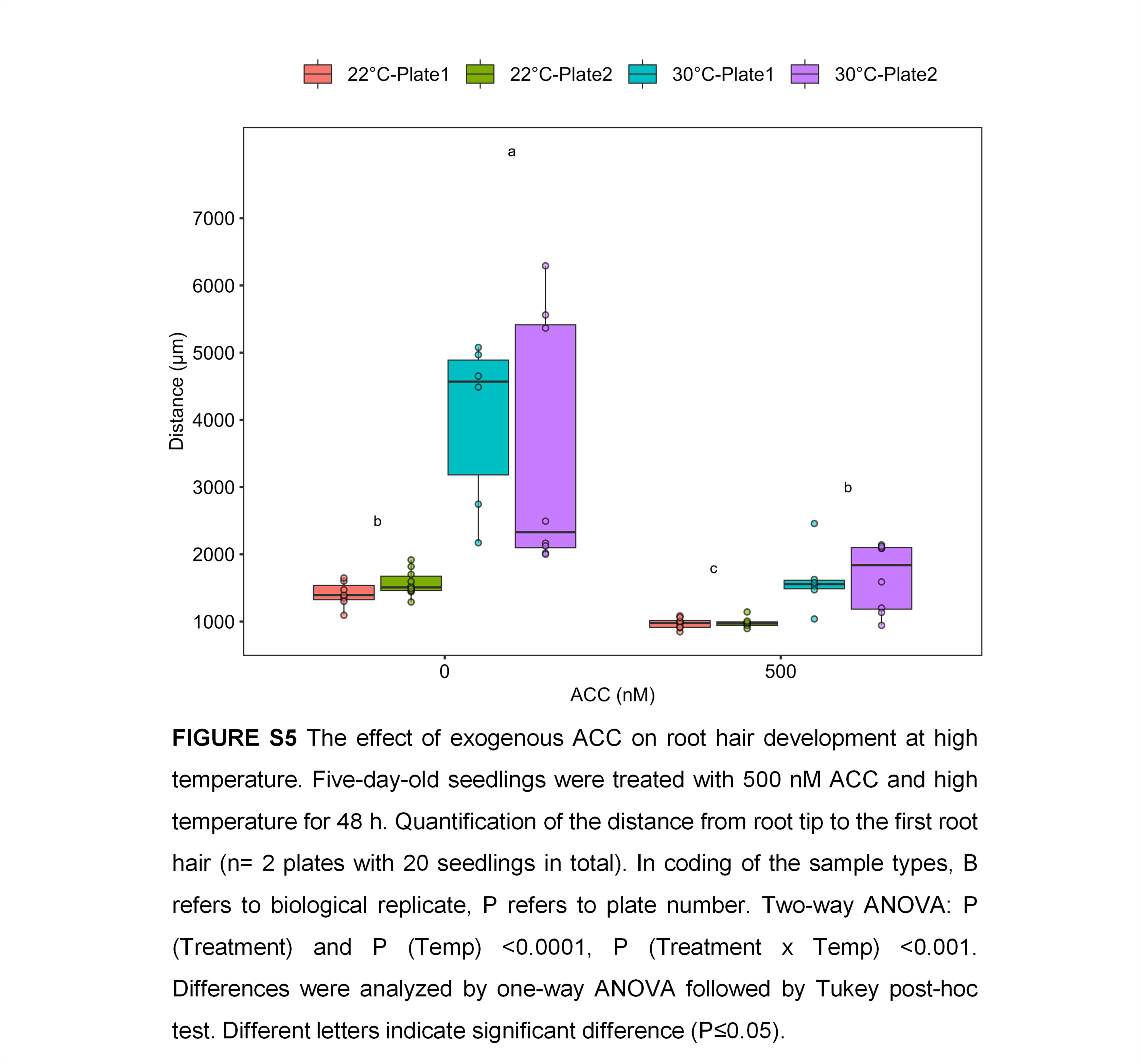

Supplement: Supplementary file 5 — FIGURE S5 The effect of exogenous ACC on root hair development at high temperature. Five‐day‐old seedlings were treated with 500 nM ACC and high temperature for 48 h. Quantification of the distance from root tip to the first root hair (n = 2 plates with 20 seedlings in total). In coding of the sample types, B refers to biological replicate, P refers to plate number. Two‐way ANOVA: P (Treatment) and P (Temp) < 0.0001, P (Treatment x Temp) < 0.001. Differences were analyzed by one‐way ANOVA followed by Tukey post hoc test. Different letters indicate significant difference (P≤ 0.05). [file PCE-48-5861-s004.png]
