# Supplementary material for: Root Hair Development Is Suppressed by Long‐Term Mild Heat Through Down‐Regulation of RHD6 and RHD6‐like Genes
Source: Plant Cell Environ. 2025 Apr 18;48(8):5861–73. doi: 10.1111/pce.15563 (PMC12223709; doi:10.1111/pce.15563)
Supplement: Supplementary file 6 — Supplemental Table S1 Gene expression levels of DEGs in root tips of seedlings grown at 22 and 30°C for 48 h. [file PCE-48-5861-s002.docx]

**Supplemental Table S1** Gene expression levels of DEGs in root tips of seedlings grown at 22 and 30 °C for 48 h.

| gene_id | log2FoldChange | padj | gene_name | gene_description |
| --- | --- | --- | --- | --- |
| AT1G01030 | 2,14 | 1,68E-08 | NGA3 | B3 domain-containing transcription factor NGA3 [Source:UniProtKB/Swiss-Prot;Acc:Q9MAN1] |
| AT1G01050 | -0,64 | 6,27E-86 | PPA1 | Soluble inorganic pyrophosphatase 1 [Source:UniProtKB/Swiss-Prot;Acc:Q93V56] |
| AT1G01060 | -3,08 | 1,73E-51 | LHY | LHY1 [Source:UniProtKB/TrEMBL;Acc:A0A178W761] |
| AT1G01070 | -3,13 | 7,11E-05 | - | WAT1-related protein [Source:UniProtKB/TrEMBL;Acc:A0A178WFU3] |
| AT1G01080 | 0,86 | 2,43E-08 | - | RNA-binding (RRM/RBD/RNP motifs) family protein [Source:UniProtKB/TrEMBL;Acc:F4HQH8] |
| AT1G01090 | -0,62 | 1,97E-70 | PDH-E1 ALPHA | Pyruvate dehydrogenase E1 component subunit alpha [Source:UniProtKB/TrEMBL;Acc:A0A178W8A7] |
| AT1G01100 | -0,72 | 7,26E-133 | RPP1A | 60S acidic ribosomal protein P1-1 [Source:UniProtKB/Swiss-Prot;Acc:Q8LCW9] |
| AT1G01130 | -0,96 | 2,21E-10 | - | CONTAINS InterPro DOMAIN/s: CBL-interacting protein kinase (InterPro:IPR020660), Calcium/calmodulin-dependent protein kinase-like (InterPro:IPR020636); BEST Arabidopsis thaliana protein match is: unknown protein (TAIR:AT5G47170.1); Has 176 Blast hit /.../76 proteins in 20 species: Archae - 0; Bacteria - 0; Metazoa - 0; Fungi - 0; Plants - 176; Viruses - 0; Other Eukaryotes - 0 (source: NCBI BLink). [Source:TAIR;Acc:AT1G01130] |
| AT1G01140 | -0,67 | 7,25E-04 | CIPK9 | CBL-interacting protein kinase 9 [Source:TAIR;Acc:AT1G01140] |
| AT1G01180 | -1,01 | 1,58E-03 | - | S-adenosyl-L-methionine-dependent methyltransferases superfamily protein [Source:UniProtKB/TrEMBL;Acc:F4HS78] |
| AT1G01190 | 4,03 | 1,91E-03 | CYP78A8 | CYP78A8 [Source:UniProtKB/TrEMBL;Acc:A0A178WIC4] |
| AT1G01200 | -1,61 | 1,06E-26 | RABA3 | RABA3 [Source:UniProtKB/TrEMBL;Acc:A0A178W3F7] |
| AT1G01220 | -0,92 | 1,96E-40 | FKGP | Bifunctional fucokinase/fucose pyrophosphorylase [Source:UniProtKB/Swiss-Prot;Acc:Q9LNJ9] |
| AT1G01230 | -0,80 | 2,53E-40 | - | ORMDL family protein [Source:UniProtKB/TrEMBL;Acc:Q9C5I0] |
| AT1G01310 | -4,46 | 5,57E-04 | - | At1g01310 [Source:UniProtKB/TrEMBL;Acc:Q6ID87] |
| AT1G01320 | 0,73 | 1,42E-37 | - | Tetratricopeptide repeat (TPR)-like superfamily protein [Source:UniProtKB/TrEMBL;Acc:F4HS99] |
| AT1G01340 | 1,32 | 1,97E-02 | CNGC10 | Probable cyclic nucleotide-gated ion channel 10 [Source:UniProtKB/Swiss-Prot;Acc:Q9LNJ0] |
| AT1G01360 | 0,61 | 5,72E-08 | PYL9 | RCAR1 [Source:UniProtKB/TrEMBL;Acc:A0A178W4H4] |
| AT1G01370 | -0,77 | 1,07E-12 | HTR12 | Histone H3-like centromeric protein HTR12 [Source:UniProtKB/Swiss-Prot;Acc:Q8RVQ9] |
| AT1G01380 | -4,71 | 7,62E-29 | ETC1 | MYB-like transcription factor ETC1 [Source:UniProtKB/Swiss-Prot;Acc:Q9LNI5] |
| AT1G01448 | 1,06 | 7,14E-26 | - | other RNA [Source:TAIR;Acc:AT1G01448] |
| AT1G01470 | 0,93 | 2,12E-42 | LEA14 | LSR3 [Source:UniProtKB/TrEMBL;Acc:A0A178W160] |
| AT1G01480 | 8,70 | 3,04E-16 | ACS2 | 1-aminocyclopropane-1-carboxylate synthase 2 [Source:UniProtKB/Swiss-Prot;Acc:Q06402] |
| AT1G01500 | 1,13 | 3,11E-17 | - | Uncharacterized protein At1g01500 [Source:UniProtKB/Swiss-Prot;Acc:Q8GUH2] |
| AT1G01520 | 1,45 | 1,98E-04 | - | ASG4 [Source:UniProtKB/TrEMBL;Acc:A0A384LJW3] |
| AT1G01570 | 0,80 | 1,08E-03 | - | F22L4.11 protein [Source:UniProtKB/TrEMBL;Acc:Q9LMM4] |
| AT1G01600 | -1,59 | 1,77E-07 | CYP86A4 | Cytochrome P450 86A4 [Source:UniProtKB/Swiss-Prot;Acc:Q9LMM1] |
| AT1G01610 | -2,19 | 8,61E-03 | GPAT4 | GPAT4 [Source:UniProtKB/TrEMBL;Acc:A0A178W9F8] |
| AT1G01640 | 2,60 | 1,22E-24 | - | BTB/POZ domain-containing protein At1g01640 [Source:UniProtKB/Swiss-Prot;Acc:Q9LQ95] |
| AT1G01670 | 0,84 | 2,31E-03 | PUB56 | U-box domain-containing protein 56 [Source:UniProtKB/Swiss-Prot;Acc:Q8GXQ7] |
| AT1G01690 | -1,02 | 6,10E-27 | ATPRD3 | Putative recombination initiation defects 3 [Source:UniProtKB/TrEMBL;Acc:F4HU48] |
| AT1G01715 | 1,22 | 1,39E-40 | - | - |
| AT1G01740 | 1,01 | 2,53E-34 | BSK4 | Serine/threonine-protein kinase BSK4 [Source:UniProtKB/Swiss-Prot;Acc:F4HU55] |
| AT1G01750 | -1,68 | 6,32E-16 | ADF11 | actin depolymerizing factor 11 [Source:TAIR;Acc:AT1G01750] |
| AT1G01790 | -1,43 | 1,96E-86 | KEA1 | K(+) efflux antiporter 1, chloroplastic [Source:UniProtKB/Swiss-Prot;Acc:Q9ZTZ7] |
| AT1G01810 | 1,57 | 1,78E-18 | - | T1N6.23 [Source:UniProtKB/TrEMBL;Acc:Q9LQ74] |
| AT1G01830 | -0,65 | 3,53E-18 | - | ARM repeat superfamily protein [Source:UniProtKB/TrEMBL;Acc:Q93YW3] |
| AT1G01880 | -0,87 | 7,52E-03 | GEN1 | Flap endonuclease GEN-like 1 [Source:UniProtKB/Swiss-Prot;Acc:Q9LPD2] |
| AT1G01910 | -0,61 | 3,06E-50 | - | ATPase [Source:UniProtKB/TrEMBL;Acc:A0A178WPT5] |
| AT1G01920 | 1,03 | 1,65E-148 | - | SET domain-containing protein [Source:UniProtKB/TrEMBL;Acc:F4HU79] |
| AT1G01940 | 1,12 | 3,10E-87 | CYP18-1 | Peptidyl-prolyl cis-trans isomerase CYP18-1 [Source:UniProtKB/Swiss-Prot;Acc:Q9LPC7] |
| AT1G02000 | 0,62 | 2,25E-23 | GAE2 | UDP-glucuronate 4-epimerase 2 [Source:UniProtKB/Swiss-Prot;Acc:Q9LPC1] |
| AT1G02050 | 1,01 | 2,69E-43 | PKSA | Type III polyketide synthase A [Source:UniProtKB/Swiss-Prot;Acc:O23674] |
| AT1G02060 | 0,87 | 9,29E-44 | - | Pentatricopeptide repeat-containing protein At1g02060, chloroplastic [Source:UniProtKB/Swiss-Prot;Acc:O81908] |
| AT1G02205 | -0,58 | 1,31E-06 | CER1 | Fatty acid hydroxylase superfamily [Source:TAIR;Acc:AT1G02205] |
| AT1G02220 | 1,26 | 6,81E-38 | NAC003 | NAC domain-containing protein 3 [Source:UniProtKB/Swiss-Prot;Acc:Q5PP28] |
| AT1G02300 | 2,61 | 1,89E-02 | CATHB1 | Cathepsin B-like protease 1 [Source:UniProtKB/Swiss-Prot;Acc:F4HVZ1] |
| AT1G02335 | 4,96 | 1,35E-04 | GL22 | Germin-like protein subfamily 2 member 2 [Source:UniProtKB/Swiss-Prot;Acc:Q9FZ27] |
| AT1G02360 | -1,07 | 5,12E-29 | - | Chitinase family protein [Source:TAIR;Acc:AT1G02360] |
| AT1G02380 | 1,04 | 3,61E-02 | - | T6A9.7 protein [Source:UniProtKB/TrEMBL;Acc:Q9FZ23] |
| AT1G02460 | -3,20 | 9,18E-31 | - | At1g02460 [Source:UniProtKB/TrEMBL;Acc:Q9FWX5] |
| AT1G02470 | 3,13 | 1,05E-17 | - | Polyketide cyclase/dehydrase and lipid transport superfamily protein [Source:UniProtKB/TrEMBL;Acc:F4HXI8] |
| AT1G02475 | 0,81 | 8,55E-07 | - | Polyketide cyclase/dehydrase and lipid transport superfamily protein [Source:UniProtKB/TrEMBL;Acc:Q94K52] |
| AT1G02520 | 2,32 | 0,00E+00 | ABCB11 | ABC transporter B family member 11 [Source:UniProtKB/Swiss-Prot;Acc:Q9FWX7] |
| AT1G02530 | 0,76 | 7,49E-06 | ABCB12 | PGP12 [Source:UniProtKB/TrEMBL;Acc:A0A178WBY9] |
| AT1G02575 | -2,46 | 5,75E-04 | - | unknown protein; BEST Arabidopsis thaliana protein match is: unknown protein (TAIR:AT1G02570.1). [Source:TAIR;Acc:AT1G02575] |
| AT1G02580 | -2,07 | 2,24E-05 | MEA | Histone-lysine N-methyltransferase MEDEA [Source:UniProtKB/Swiss-Prot;Acc:O65312] |
| AT1G02640 | -1,65 | 1,69E-26 | BXL2 | Probable beta-D-xylosidase 2 [Source:UniProtKB/Swiss-Prot;Acc:Q94KD8] |
| AT1G02670 | 0,97 | 2,26E-14 | - | P-loop containing nucleoside triphosphate hydrolases superfamily protein [Source:TAIR;Acc:AT1G02670] |
| AT1G02730 | -0,84 | 9,27E-36 | CSLD5 | Cellulose synthase-like protein D5 [Source:UniProtKB/Swiss-Prot;Acc:Q9SRW9] |
| AT1G02750 | 1,37 | 9,37E-42 | DI19-2 | Protein DEHYDRATION-INDUCED 19 homolog 2 [Source:UniProtKB/Swiss-Prot;Acc:Q8GWK1] |
| AT1G02780 | -0,61 | 8,30E-89 | RPL19A | 60S ribosomal protein L19-1 [Source:UniProtKB/Swiss-Prot;Acc:Q9SRX2] |
| AT1G02810 | -0,73 | 1,89E-53 | PME7 | Probable pectinesterase/pectinesterase inhibitor 7 [Source:UniProtKB/Swiss-Prot;Acc:Q9SRX4] |
| AT1G02813 | -1,68 | 4,41E-02 | - | Protein of unknown function, DUF538 [Source:TAIR;Acc:AT1G02813] |
| AT1G02820 | 1,07 | 3,03E-02 | LEA2 | LEA3 [Source:UniProtKB/TrEMBL;Acc:A0A178WJ88] |
| AT1G02850 | 1,87 | 5,59E-244 | BGLU11 | Beta-glucosidase 11 [Source:UniProtKB/Swiss-Prot;Acc:B3H5Q1] |
| AT1G02860 | 1,10 | 1,38E-30 | BAH1 | E3 ubiquitin-protein ligase BAH1 [Source:UniProtKB/Swiss-Prot;Acc:Q9SRX9] |
| AT1G02920 | -5,20 | 2,92E-04 | GSTF7 | Glutathione S-transferase F7 [Source:UniProtKB/Swiss-Prot;Acc:Q9SRY5] |
| AT1G02980 | 7,17 | 7,08E-11 | CUL2 | Cullin-2 [Source:UniProtKB/Swiss-Prot;Acc:Q9SRZ0] |
| AT1G03010 | 0,91 | 1,09E-02 | - | BTB/POZ domain-containing protein At1g03010 [Source:UniProtKB/Swiss-Prot;Acc:Q9SA69] |
| AT1G03106 | -5,68 | 9,85E-06 | - | At1g03105 [Source:UniProtKB/TrEMBL;Acc:Q8L760] |
| AT1G03130 | -0,96 | 9,22E-03 | PSAD2 | Photosystem I reaction center subunit II-2, chloroplastic [Source:UniProtKB/Swiss-Prot;Acc:Q9SA56] |
| AT1G03170 | 1,19 | 4,30E-05 | FAF2 | Protein FANTASTIC FOUR 2 [Source:UniProtKB/Swiss-Prot;Acc:Q8GXU9] |
| AT1G03260 | -0,60 | 1,64E-13 | - | At1g03260 [Source:UniProtKB/TrEMBL;Acc:Q6NMJ6] |
| AT1G03290 | 0,82 | 2,21E-41 | - | ELKS/Rab6-interacting/CAST family protein [Source:UniProtKB/TrEMBL;Acc:Q9ZVT0] |
| AT1G03410 | 2,70 | 8,28E-28 | 2A6 | 1-aminocyclopropane-1-carboxylate oxidase homolog 5 [Source:UniProtKB/Swiss-Prot;Acc:Q43383] |
| AT1G03440 | 1,28 | 3,50E-16 | - | Leucine-rich repeat (LRR) family protein [Source:UniProtKB/TrEMBL;Acc:F4I0W7] |
| AT1G03470 | 1,27 | 2,67E-21 | NET3A | NET3A [Source:UniProtKB/TrEMBL;Acc:A0A178W5C1] |
| AT1G03495 | 1,25 | 3,18E-29 | - | HXXXD-type acyl-transferase family protein [Source:TAIR;Acc:AT1G03495] |
| AT1G03510 | 0,72 | 8,13E-06 | PCMP-E3 | Putative pentatricopeptide repeat-containing protein At1g03510 [Source:UniProtKB/Swiss-Prot;Acc:Q9LR72] |
| AT1G03520 | -0,60 | 1,57E-12 | - | Core-2/I-branching beta-1,6-N-acetylglucosaminyltransferase family protein [Source:UniProtKB/TrEMBL;Acc:Q9LR71] |
| AT1G03550 | -1,32 | 3,00E-10 | SCAMP2 | Secretory carrier-associated membrane protein 2 [Source:UniProtKB/Swiss-Prot;Acc:Q9LR68] |
| AT1G03600 | -1,08 | 1,57E-02 | PSB27-1 | Photosystem II repair protein PSB27-H1, chloroplastic [Source:UniProtKB/Swiss-Prot;Acc:Q9LR64] |
| AT1G03620 | 1,53 | 6,29E-03 | - | ELMO/CED-12 family protein [Source:TAIR;Acc:AT1G03620] |
| AT1G03650 | 0,91 | 1,41E-14 | - | Acyl-CoA N-acyltransferases (NAT) superfamily protein [Source:UniProtKB/TrEMBL;Acc:Q8GWN1] |
| AT1G03660 | 4,71 | 5,36E-25 | - | Ankyrin-repeat containing protein [Source:UniProtKB/TrEMBL;Acc:F4I2G0] |
| AT1G03740 | 0,90 | 1,23E-33 | - | F21B7.34 [Source:UniProtKB/TrEMBL;Acc:Q9LR53] |
| AT1G03760 | 0,98 | 4,70E-43 | - | Prefoldin chaperone subunit family protein [Source:UniProtKB/TrEMBL;Acc:Q8GWP1] |
| AT1G03770 | 1,11 | 1,13E-99 | RING1B | RING 1B [Source:UniProtKB/TrEMBL;Acc:F4I2H4] |
| AT1G03800 | 0,90 | 1,03E-04 | ERF10 | At1g03800 [Source:UniProtKB/TrEMBL;Acc:Q08A59] |
| AT1G03820 | -5,08 | 5,28E-04 | - | At1g03820 [Source:UniProtKB/TrEMBL;Acc:Q9ZWA4] |
| AT1G03840 | -0,70 | 9,62E-23 | MGP | Uncharacterized protein At1g03840 (Fragment) [Source:UniProtKB/TrEMBL;Acc:C0SUS5] |
| AT1G03880 | 3,10 | 1,43E-04 | CRB | CRU2 [Source:UniProtKB/TrEMBL;Acc:A0A178W5K4] |
| AT1G03900 | -0,75 | 5,66E-89 | ATNAP4 | NAP4 [Source:UniProtKB/TrEMBL;Acc:A0A178WMR3] |
| AT1G03970 | 0,71 | 8,88E-10 | GBF4 | At1g03970 [Source:UniProtKB/TrEMBL;Acc:Q2HIT6] |
| AT1G03980 | 0,61 | 1,59E-04 | ATPCS2 | phytochelatin synthase 2 [Source:TAIR;Acc:AT1G03980] |
| AT1G03990 | 1,13 | 1,63E-14 | FAO1 | Long-chain-alcohol oxidase FAO1 [Source:UniProtKB/Swiss-Prot;Acc:Q9ZWB9] |
| AT1G04020 | -0,97 | 9,09E-49 | ATBARD1 | ROW1 [Source:UniProtKB/TrEMBL;Acc:A0A384KH31] |
| AT1G04030 | 0,60 | 3,35E-13 | - | unknown protein; BEST Arabidopsis thaliana protein match is: unknown protein (TAIR:AT5G44040.1); Ha. [Source:TAIR;Acc:AT1G04030] |
| AT1G04040 | -1,08 | 3,95E-19 | - | At1g04040/F21M11_2 [Source:UniProtKB/TrEMBL;Acc:Q9ZWC4] |
| AT1G04090 | 0,92 | 1,06E-03 | - | At1g04090 [Source:UniProtKB/TrEMBL;Acc:Q501D1] |
| AT1G04120 | -0,64 | 2,18E-41 | ABCC5 | MRP5 [Source:UniProtKB/TrEMBL;Acc:A0A178WGC2] |
| AT1G04160 | -1,10 | 2,04E-39 | XI-B | Myosin-8 [Source:UniProtKB/Swiss-Prot;Acc:F4I460] |
| AT1G04187 | 1,60 | 1,22E-07 | - | - |
| AT1G04200 | -0,63 | 9,16E-47 | - | Dyggve-melchior-clausen syndrome protein [Source:UniProtKB/TrEMBL;Acc:Q8RWV1] |
| AT1G04237 | -1,70 | 5,59E-04 | - | - |
| AT1G04240 | -1,64 | 3,29E-05 | SHY2 | AUX/IAA transcriptional regulator family protein [Source:TAIR;Acc:AT1G04240] |
| AT1G04270 | -0,83 | 8,35E-168 | RPS15A | 40S ribosomal protein S15-1 [Source:UniProtKB/Swiss-Prot;Acc:Q08112] |
| AT1G04280 | -0,86 | 1,89E-08 | - | p-loop containing nucleoside triphosphate hydrolases superfamily protein [Source:UniProtKB/TrEMBL;Acc:Q0WUY1] |
| AT1G04290 | -0,89 | 5,92E-07 | - | F19P19.27 protein [Source:UniProtKB/TrEMBL;Acc:P93828] |
| AT1G04330 | 1,59 | 5,30E-03 | - | At1g04330 [Source:UniProtKB/TrEMBL;Acc:O22691] |
| AT1G04340 | -1,11 | 6,02E-100 | - | At1g04340/F19P19_23 [Source:UniProtKB/TrEMBL;Acc:O22690] |
| AT1G04350 | 1,52 | 5,03E-04 | - | 1-aminocyclopropane-1-carboxylate oxidase homolog 6 [Source:UniProtKB/Swiss-Prot;Acc:P93824] |
| AT1G04360 | -1,20 | 1,66E-18 | ATL1 | RING-H2 finger protein ATL1 [Source:UniProtKB/Swiss-Prot;Acc:P93823] |
| AT1G04410 | -0,94 | 1,39E-205 | MDH1 | Malate dehydrogenase [Source:UniProtKB/TrEMBL;Acc:A0A178W4H0] |
| AT1G04417 | 1,41 | 1,78E-02 | - | - |
| AT1G04425 | -1,99 | 7,69E-64 | - | other RNA [Source:TAIR;Acc:AT1G04425] |
| AT1G04430 | -0,88 | 2,13E-59 | - | Probable methyltransferase PMT8 [Source:UniProtKB/Swiss-Prot;Acc:Q940J9] |
| AT1G04467 | 3,31 | 3,05E-02 | - | - |
| AT1G04483 | 2,75 | 2,08E-02 | - | - |
| AT1G04517 | 2,89 | 3,39E-14 | - | - |
| AT1G04523 | 2,25 | 8,27E-04 | - | - |
| AT1G04540 | -0,72 | 5,74E-13 | - | Calcium-dependent lipid-binding (CaLB domain) family protein [Source:UniProtKB/TrEMBL;Acc:F4I5P7] |
| AT1G04570 | 1,34 | 2,90E-06 | - | Probable folate-biopterin transporter 8, chloroplastic [Source:UniProtKB/Swiss-Prot;Acc:F4I5Q2] |
| AT1G04580 | 1,11 | 1,78E-04 | AAO4 | Benzaldehyde dehydrogenase (NAD(+)) [Source:UniProtKB/Swiss-Prot;Acc:Q7G191] |
| AT1G04610 | -1,18 | 1,22E-48 | YUC3 | Probable indole-3-pyruvate monooxygenase YUCCA3 [Source:UniProtKB/Swiss-Prot;Acc:O23024] |
| AT1G04620 | 2,00 | 1,77E-25 | HCAR | 7-hydroxymethyl chlorophyll a reductase, chloroplastic [Source:UniProtKB/Swiss-Prot;Acc:Q8GS60] |
| AT1G04630 | -0,61 | 7,03E-44 | MEE4 | NADH dehydrogenase [ubiquinone] 1 alpha subcomplex subunit 13-A [Source:UniProtKB/Swiss-Prot;Acc:Q8RWA7] |
| AT1G04650 | -0,67 | 3,45E-11 | - | unknown protein; Ha. [Source:TAIR;Acc:AT1G04650] |
| AT1G04660 | -6,51 | 3,59E-08 | - | Glycine-rich protein [Source:UniProtKB/TrEMBL;Acc:O23018] |
| AT1G04670 | 2,06 | 4,49E-03 | - | Putative uncharacterized protein [Source:UniProtKB/TrEMBL;Acc:Q5Q0I9] |
| AT1G04680 | -0,97 | 9,45E-150 | - | Pectate lyase [Source:UniProtKB/TrEMBL;Acc:A0A178W464] |
| AT1G04690 | -0,59 | 1,30E-48 | KAB1 | Probable voltage-gated potassium channel subunit beta [Source:UniProtKB/Swiss-Prot;Acc:O23016] |
| AT1G04700 | 0,68 | 1,34E-08 | - | PB1 domain-containing protein tyrosine kinase [Source:UniProtKB/TrEMBL;Acc:F4I5S1] |
| AT1G04710 | 0,62 | 1,56E-26 | KAT1 | PKT4 [Source:UniProtKB/TrEMBL;Acc:A0A178W7T5] |
| AT1G04760 | -0,59 | 1,58E-03 | VAMP726 | Putative vesicle-associated membrane protein 726 [Source:UniProtKB/Swiss-Prot;Acc:Q9MAS5] |
| AT1G04770 | 2,09 | 1,14E-23 | - | Protein SULFUR DEFICIENCY-INDUCED 2 [Source:UniProtKB/Swiss-Prot;Acc:Q8L730] |
| AT1G04820 | -0,83 | 9,21E-65 | TUBA4 | Tubulin alpha-2 chain [Source:UniProtKB/Swiss-Prot;Acc:B9DGT7] |
| AT1G04827 | 0,94 | 1,88E-02 | - | - |
| AT1G04910 | -0,81 | 5,63E-61 | OFUT1 | O-fucosyltransferase 1 [Source:UniProtKB/Swiss-Prot;Acc:Q8W486] |
| AT1G04920 | 0,93 | 6,19E-03 | SPS3 | Probable sucrose-phosphate synthase 3 [Source:UniProtKB/Swiss-Prot;Acc:Q8RY24] |
| AT1G04960 | 0,67 | 3,95E-19 | - | Protein of unknown function (DUF1664) [Source:TAIR;Acc:AT1G04960] |
| AT1G04970 | 0,67 | 1,58E-19 | - | Putative BPI/LBP family protein At1g04970 [Source:UniProtKB/Swiss-Prot;Acc:Q9MAU5] |
| AT1G04985 | 0,95 | 7,49E-78 | - | Triacylglycerol lipase-like protein [Source:UniProtKB/TrEMBL;Acc:Q84JL8] |
| AT1G05000 | -0,94 | 2,13E-06 | - | Phosphotyrosine protein phosphatases superfamily protein [Source:UniProtKB/TrEMBL;Acc:F4I780] |
| AT1G05030 | 0,63 | 4,00E-10 | - | Probable plastidic glucose transporter 1 [Source:UniProtKB/Swiss-Prot;Acc:Q0WVE9] |
| AT1G05043 | -1,21 | 9,12E-04 | - | - |
| AT1G05060 | 2,12 | 1,14E-78 | - | At1g05060 [Source:UniProtKB/TrEMBL;Acc:Q9ZVP0] |
| AT1G05065 | 3,89 | 1,25E-08 | CLE20 | CLAVATA3/ESR (CLE)-related protein 20 [Source:UniProtKB/Swiss-Prot;Acc:Q3EDI6] |
| AT1G05200 | 1,35 | 1,41E-25 | GLR3.4 | Glutamate receptor [Source:UniProtKB/TrEMBL;Acc:Q53YX3] |
| AT1G05210 | -1,20 | 6,81E-46 | - | At1g05210 [Source:UniProtKB/TrEMBL;Acc:O23047] |
| AT1G05230 | -1,23 | 2,53E-02 | HDG2 | AT1G05230 protein [Source:UniProtKB/TrEMBL;Acc:B9DFH8] |
| AT1G05260 | -0,78 | 1,34E-46 | PER3 | Peroxidase [Source:UniProtKB/TrEMBL;Acc:Q0WSR2] |
| AT1G05280 | 0,75 | 6,20E-31 | - | Protein of unknown function (DUF604) [Source:TAIR;Acc:AT1G05280] |
| AT1G05290 | 3,99 | 8,89E-03 | - | CCT motif family protein [Source:UniProtKB/TrEMBL;Acc:Q1PFY3] |
| AT1G05300 | 1,81 | 6,38E-65 | ZIP5 | Zinc transporter 5 [Source:UniProtKB/Swiss-Prot;Acc:O23039] |
| AT1G05320 | -1,57 | 1,24E-33 | - | FUNCTIONS IN: molecular_function unknown; INVOLVED IN: biological_process unknown; LOCATED IN: cellular_component unknown; EXPRESSED IN: fruit, egg cell; CONTAINS InterPro DOMAIN/s: Prefoldin (InterPro:IPR009053); BEST Arabidopsis thaliana protein m /.../s: unknown protein (TAIR:AT2G32240.1); Ha. [Source:TAIR;Acc:AT1G05320] |
| AT1G05340 | 2,96 | 3,86E-04 | - | Cysteine-rich TM module stress tolerance protein [Source:UniProtKB/TrEMBL;Acc:O23035] |
| AT1G05350 | -0,77 | 2,59E-53 | - | Ubiquitin-like modifier-activating enzyme 5 [Source:UniProtKB/Swiss-Prot;Acc:O23034] |
| AT1G05385 | 2,30 | 1,51E-33 | PSB27-2 | Psb27-H1 [Source:UniProtKB/TrEMBL;Acc:A0A178WJM6] |
| AT1G05397 | 1,91 | 1,57E-14 | - | - |
| AT1G05400 | 1,49 | 2,71E-10 | - | Putative uncharacterized protein [Source:UniProtKB/TrEMBL;Acc:Q5XVL8] |
| AT1G05420 | -1,48 | 1,56E-08 | ATOFP12 | ovate family protein 12 [Source:TAIR;Acc:AT1G05420] |
| AT1G05440 | -1,09 | 1,43E-14 | - | C-8 sterol isomerase [Source:UniProtKB/TrEMBL;Acc:Q9ZVZ4] |
| AT1G05490 | 2,39 | 6,32E-55 | CLSY3 | SNF2 domain-containing protein CLASSY 3 [Source:UniProtKB/Swiss-Prot;Acc:F4I8S3] |
| AT1G05517 | -1,33 | 1,66E-04 | - | - |
| AT1G05530 | -2,08 | 1,01E-91 | UGT75B2 | UGT75B2 [Source:UniProtKB/TrEMBL;Acc:A0A384L0M1] |
| AT1G05570 | -1,74 | 2,16E-69 | CALS1 | Callose synthase 1 [Source:UniProtKB/Swiss-Prot;Acc:Q9AUE0] |
| AT1G05583 | 3,62 | 1,80E-02 | - | - |
| AT1G05600 | 1,10 | 2,40E-16 | - | Pentatricopeptide repeat-containing protein At1g05600 [Source:UniProtKB/Swiss-Prot;Acc:Q9SYK1] |
| AT1G05630 | -0,61 | 1,93E-19 | AT5PTASE13 | Endonuclease/exonuclease/phosphatase family protein [Source:TAIR;Acc:AT1G05630] |
| AT1G05660 | -0,63 | 2,62E-17 | - | F3F20.11 protein [Source:UniProtKB/TrEMBL;Acc:Q9SYK7] |
| AT1G05675 | 4,02 | 1,06E-18 | UGT74E1 | Glycosyltransferase (Fragment) [Source:UniProtKB/TrEMBL;Acc:A0A1W6AJW6] |
| AT1G05680 | 2,35 | 0,00E+00 | UGT74E2 | Glycosyltransferase (Fragment) [Source:UniProtKB/TrEMBL;Acc:A0A0K1SBE8] |
| AT1G05687 | 1,10 | 2,70E-02 | - | - |
| AT1G05690 | 3,34 | 2,15E-35 | BT3 | BTB/POZ and TAZ domain-containing protein 3 [Source:UniProtKB/Swiss-Prot;Acc:Q9SYL0] |
| AT1G05700 | -2,36 | 2,14E-02 | - | Leucine-rich repeat transmembrane protein kinase protein [Source:TAIR;Acc:AT1G05700] |
| AT1G05710 | 1,30 | 1,82E-32 | - | - |
| AT1G05720 | -0,66 | 1,56E-23 | - | Selenoprotein family protein [Source:UniProtKB/TrEMBL;Acc:Q8GWP6] |
| AT1G05730 | 1,32 | 3,13E-66 | - | FAM136A-like protein (DUF842) [Source:UniProtKB/TrEMBL;Acc:Q5BQ23] |
| AT1G05740 | 3,27 | 7,24E-08 | - | F3F20.19 protein [Source:UniProtKB/TrEMBL;Acc:Q9SYL5] |
| AT1G05750 | 0,71 | 2,40E-08 | PDE247 | Pentatricopeptide repeat-containing protein At1g05750, chloroplastic [Source:UniProtKB/Swiss-Prot;Acc:Q9MA50] |
| AT1G05773 | -0,60 | 4,22E-03 | - | - |
| AT1G05783 | 2,32 | 1,79E-02 | - | - |
| AT1G05810 | 0,73 | 1,81E-16 | ARA | RAB GTPase homolog A5E [Source:TAIR;Acc:AT1G05810] |
| AT1G05820 | 1,06 | 3,70E-11 | SPPL5 | Signal peptide peptidase-like 5 [Source:UniProtKB/Swiss-Prot;Acc:Q9MA44] |
| AT1G05835 | -2,81 | 7,39E-04 | - | Uncharacterized protein At1g05835 [Source:UniProtKB/Swiss-Prot;Acc:A8MS78] |
| AT1G05840 | 0,80 | 2,87E-26 | - | Eukaryotic aspartyl protease family protein [Source:UniProtKB/TrEMBL;Acc:F4IAD5] |
| AT1G05890 | 0,78 | 1,60E-68 | ARI5 | RBR-type E3 ubiquitin transferase [Source:UniProtKB/TrEMBL;Acc:F4IAE4] |
| AT1G05970 | 0,77 | 5,51E-09 | - | RNA-binding (RRM/RBD/RNP motifs) family protein [Source:UniProtKB/TrEMBL;Acc:Q9LNE8] |
| AT1G05987 | 3,86 | 2,98E-02 | - | - |
| AT1G06033 | -0,80 | 2,58E-04 | - | - |
| AT1G06037 | 2,56 | 1,64E-04 | - | - |
| AT1G06050 | 0,61 | 1,40E-05 | - | Protein of unknown function (DUF1336) [Source:TAIR;Acc:AT1G06050] |
| AT1G06073 | 3,27 | 3,44E-03 | - | - |
| AT1G06080 | -0,72 | 2,24E-09 | ADS1 | Delta-9 acyl-lipid desaturase 1 [Source:UniProtKB/Swiss-Prot;Acc:O65797] |
| AT1G06083 | 1,21 | 6,51E-03 | - | - |
| AT1G06110 | 0,92 | 9,39E-56 | SKIP16 | F-box protein SKIP16 [Source:UniProtKB/Swiss-Prot;Acc:Q9LND7] |
| AT1G06130 | 0,84 | 2,15E-25 | GLX2-4 | GLX2-4 [Source:UniProtKB/TrEMBL;Acc:A0A178WGQ2] |
| AT1G06143 | 0,75 | 2,02E-08 | EMB1444 | Pentatricopeptide repeat-containing protein At1g06143 [Source:UniProtKB/Swiss-Prot;Acc:Q56X05] |
| AT1G06173 | 1,25 | 1,36E-02 | - | - |
| AT1G06190 | 0,76 | 9,08E-43 | - | Rho termination factor [Source:TAIR;Acc:AT1G06190] |
| AT1G06197 | -2,33 | 2,25E-02 | - | - |
| AT1G06250 | 1,58 | 6,16E-17 | - | Phospholipase A1-IIalpha [Source:UniProtKB/Swiss-Prot;Acc:Q9LNC2] |
| AT1G06265 | 0,92 | 6,18E-09 | - | other RNA [Source:TAIR;Acc:AT1G06265] |
| AT1G06310 | -0,96 | 1,48E-27 | ACX3.2 | Putative acyl-coenzyme A oxidase 3.2, peroxisomal [Source:UniProtKB/Swiss-Prot;Acc:Q9LMI7] |
| AT1G06320 | -1,37 | 5,69E-09 | - | Putative uncharacterized protein [Source:UniProtKB/TrEMBL;Acc:Q3EDH7] |
| AT1G06350 | -3,52 | 2,19E-08 | - | Delta-9 desaturase-like 4 protein [Source:UniProtKB/Swiss-Prot;Acc:Q9LMI4] |
| AT1G06383 | 3,35 | 3,05E-03 | - | - |
| AT1G06430 | 1,74 | 1,48E-125 | FTSH8 | ATP-dependent zinc metalloprotease FTSH 8, chloroplastic [Source:UniProtKB/Swiss-Prot;Acc:Q8W585] |
| AT1G06450 | 1,08 | 1,33E-03 | CAF1-1 | Probable CCR4-associated factor 1 homolog 1 [Source:UniProtKB/Swiss-Prot;Acc:Q9SHJ0] |
| AT1G06460 | 2,18 | 1,03E-12 | ACD32.1 | Alpha-crystallin domain 32.1 [Source:UniProtKB/TrEMBL;Acc:Q208N7] |
| AT1G06463 | -1,89 | 3,60E-03 | - | - |
| AT1G06470 | -0,70 | 8,11E-32 | - | Probable sugar phosphate/phosphate translocator At1g06470 [Source:UniProtKB/Swiss-Prot;Acc:Q8H184] |
| AT1G06483 | -1,04 | 5,70E-03 | - | - |
| AT1G06490 | 0,70 | 3,98E-10 | CALS7 | Callose synthase 7 [Source:UniProtKB/Swiss-Prot;Acc:Q9SHJ3] |
| AT1G06500 | 0,72 | 7,11E-13 | - | AT1G06500 protein [Source:UniProtKB/TrEMBL;Acc:Q8VY54] |
| AT1G06510 | 0,73 | 6,34E-11 | - | Forkhead-associated domain protein [Source:UniProtKB/TrEMBL;Acc:Q84K72] |
| AT1G06515 | -0,82 | 3,19E-26 | - | Protein of unknown function (DUF3317) [Source:TAIR;Acc:AT1G06515] |
| AT1G06540 | -1,45 | 1,28E-14 | - | F12K11.13 [Source:UniProtKB/TrEMBL;Acc:Q9SHJ7] |
| AT1G06560 | 0,86 | 2,21E-91 | - | At1g06560 [Source:UniProtKB/TrEMBL;Acc:Q84MA1] |
| AT1G06570 | 0,61 | 8,00E-18 | HPD | 4-hydroxyphenylpyruvate dioxygenase [Source:UniProtKB/Swiss-Prot;Acc:P93836] |
| AT1G06573 | 0,69 | 2,67E-03 | - | - |
| AT1G06630 | 0,73 | 7,03E-35 | - | F-box/RNI-like superfamily protein [Source:UniProtKB/TrEMBL;Acc:F4IDP6] |
| AT1G06640 | 2,12 | 0,00E+00 | - | 1-aminocyclopropane-1-carboxylate oxidase homolog 2 [Source:UniProtKB/Swiss-Prot;Acc:Q9C5K7] |
| AT1G06667 | -4,42 | 1,45E-02 | - | - |
| AT1G06680 | -1,17 | 4,43E-20 | PSBP1 | Oxygen-evolving enhancer protein 2-1, chloroplastic [Source:UniProtKB/Swiss-Prot;Acc:Q42029] |
| AT1G06740 | 0,77 | 4,40E-10 | - | At1g06740 [Source:UniProtKB/TrEMBL;Acc:Q9M9Y2] |
| AT1G06760 | -0,59 | 8,08E-48 | - | Histone H1.1 [Source:UniProtKB/Swiss-Prot;Acc:P26568] |
| AT1G06770 | 0,60 | 2,32E-15 | DRIP1 | E3 ubiquitin protein ligase DRIP1 [Source:UniProtKB/Swiss-Prot;Acc:Q9M9Y4] |
| AT1G06773 | -3,09 | 4,25E-05 | - | - |
| AT1G06780 | -0,73 | 3,44E-45 | GAUT6 | Hexosyltransferase [Source:UniProtKB/TrEMBL;Acc:F4IDS0] |
| AT1G06800 | 1,48 | 9,63E-29 | PLA-I{gamma}1 | PLA-I(gamma)1 [Source:UniProtKB/TrEMBL;Acc:A0A178WG46] |
| AT1G06837 | 2,08 | 9,59E-08 | - | - |
| AT1G06840 | 0,90 | 1,32E-52 | - | Probable LRR receptor-like serine/threonine-protein kinase At1g06840 [Source:UniProtKB/Swiss-Prot;Acc:C0LGD7] |
| AT1G06860 | 3,00 | 5,78E-04 | - | pre-tRNA [Source:TAIR;Acc:AT1G06860] |
| AT1G06930 | -1,97 | 1,92E-56 | - | Expressed protein [Source:UniProtKB/TrEMBL;Acc:Q5BQ22] |
| AT1G06933 | 4,32 | 8,53E-03 | - | - |
| AT1G06970 | 1,96 | 2,75E-10 | CHX14 | CHX14 [Source:UniProtKB/TrEMBL;Acc:A0A178WC73] |
| AT1G06983 | 4,12 | 2,66E-03 | - | - |
| AT1G07000 | -0,88 | 2,27E-15 | ATEXO70B2 | Exocyst subunit Exo70 family protein [Source:UniProtKB/TrEMBL;Acc:A0A178WCB5] |
| AT1G07010 | 0,66 | 4,19E-04 | - | Calcineurin-like metallo-phosphoesterase superfamily protein [Source:UniProtKB/TrEMBL;Acc:F4HNW0] |
| AT1G07020 | -0,64 | 6,09E-17 | - | Uncharacterized protein At1g07020 [Source:UniProtKB/TrEMBL;Acc:Q8LCS6] |
| AT1G07023 | 4,26 | 1,27E-06 | - | - |
| AT1G07027 | 1,98 | 2,38E-04 | - | - |
| AT1G07070 | -1,56 | 1,87E-161 | RPL35AA | 60S ribosomal protein L35a-1 [Source:UniProtKB/Swiss-Prot;Acc:Q9LMK0] |
| AT1G07090 | 1,58 | 2,01E-33 | LSH6 | LSH6 [Source:UniProtKB/TrEMBL;Acc:A0A178WE41] |
| AT1G07135 | 1,43 | 1,94E-03 | - | At1g07135 [Source:UniProtKB/TrEMBL;Acc:Q9LMK6] |
| AT1G07140 | -0,63 | 1,71E-38 | RANBP1A | Ran-binding protein 1 homolog a [Source:UniProtKB/Swiss-Prot;Acc:Q9LMK7] |
| AT1G07180 | 4,03 | 0,00E+00 | NDA1 | Internal alternative NAD(P)H-ubiquinone oxidoreductase A1, mitochondrial [Source:UniProtKB/Swiss-Prot;Acc:Q8GWA1] |
| AT1G07230 | 0,80 | 7,49E-25 | NPC1 | Non-specific phospholipase C1 [Source:UniProtKB/Swiss-Prot;Acc:Q8L7Y9] |
| AT1G07270 | -0,91 | 1,10E-08 | CDC6B | Cell division control protein 6 homolog B [Source:UniProtKB/Swiss-Prot;Acc:Q8W032] |
| AT1G07300 | 1,13 | 1,88E-15 | - | josephin protein-related [Source:TAIR;Acc:AT1G07300] |
| AT1G07310 | 0,60 | 5,48E-17 | - | Calcium-dependent lipid-binding (CaLB domain) family protein [Source:UniProtKB/TrEMBL;Acc:Q9LNV0] |
| AT1G07313 | 0,97 | 8,08E-03 | - | - |
| AT1G07350 | 0,85 | 2,56E-70 | SR45A | Serine/arginine-rich splicing factor SR45a [Source:UniProtKB/Swiss-Prot;Acc:Q84TH4] |
| AT1G07370 | -0,86 | 2,46E-64 | PCNA | Proliferating cellular nuclear antigen 1 [Source:UniProtKB/Swiss-Prot;Acc:Q9M7Q7] |
| AT1G07390 | 1,13 | 1,72E-15 | AtRLP1 | Receptor like protein 1 [Source:UniProtKB/TrEMBL;Acc:F4HQM4] |
| AT1G07393 | -1,85 | 2,67E-06 | - | - |
| AT1G07400 | 3,99 | 1,09E-116 | HSP17.8 | 17.8 kDa class I heat shock protein [Source:UniProtKB/Swiss-Prot;Acc:Q9LNW0] |
| AT1G07410 | -1,09 | 1,83E-50 | RABA2B | RABA2b [Source:UniProtKB/TrEMBL;Acc:A0A178WHD5] |
| AT1G07470 | -0,82 | 3,30E-39 | - | At1g07470/F22G5_13 [Source:UniProtKB/TrEMBL;Acc:Q93VP4] |
| AT1G07480 | -0,66 | 6,88E-24 | - | Transcription factor IIA, alpha/beta subunit [Source:TAIR;Acc:AT1G07480] |
| AT1G07483 | -3,36 | 1,97E-02 | - | - |
| AT1G07587 | 1,48 | 1,43E-02 | - | - |
| AT1G07590 | 2,08 | 1,36E-134 | - | Pentatricopeptide repeat-containing protein At1g07590, mitochondrial [Source:UniProtKB/Swiss-Prot;Acc:Q940Q2] |
| AT1G07650 | 1,15 | 1,43E-38 | - | Leucine-rich repeat transmembrane protein kinase [Source:UniProtKB/TrEMBL;Acc:F4HSE1] |
| AT1G07750 | -1,23 | 1,62E-68 | - | At1g07750/F24B9_13 [Source:UniProtKB/TrEMBL;Acc:Q9LQQ3] |
| AT1G07780 | 0,84 | 1,11E-46 | PAI1 | N-(5'-phosphoribosyl)anthranilate isomerase 1, chloroplastic [Source:UniProtKB/Swiss-Prot;Acc:Q42440] |
| AT1G07790 | -1,48 | 5,43E-194 | HTB1 | Histone H2B.1 [Source:UniProtKB/Swiss-Prot;Acc:Q9LQQ4] |
| AT1G07795 | -4,19 | 5,84E-09 | - | At1g07795 [Source:UniProtKB/TrEMBL;Acc:Q29PW8] |
| AT1G07820 | -1,24 | 1,81E-135 | - | Histone H4 [Source:UniProtKB/TrEMBL;Acc:Q6NR90] |
| AT1G07870 | 0,83 | 6,92E-08 | - | Protein kinase superfamily protein [Source:UniProtKB/TrEMBL;Acc:F4HSH9] |
| AT1G07880 | 2,53 | 1,30E-02 | MPK13 | Mitogen-activated protein kinase 13 [Source:UniProtKB/Swiss-Prot;Acc:Q9LQQ9] |
| AT1G07890 | -0,66 | 1,36E-88 | APX1 | MEE6 [Source:UniProtKB/TrEMBL;Acc:A0A178W5I1] |
| AT1G07897 | -0,84 | 5,30E-09 | - | - |
| AT1G07902 | 1,70 | 2,89E-26 | - | - |
| AT1G07937 | 0,77 | 6,34E-07 | - | - |
| AT1G08010 | -0,61 | 3,09E-25 | GATA11 | GATA11 [Source:UniProtKB/TrEMBL;Acc:A0A384LF57] |
| AT1G08050 | 0,88 | 6,72E-06 | - | T6D22.13 [Source:UniProtKB/TrEMBL;Acc:Q9LN03] |
| AT1G08190 | -0,83 | 1,02E-99 | VPS41 | Vacuolar protein sorting-associated protein 41 homolog [Source:UniProtKB/Swiss-Prot;Acc:P93043] |
| AT1G08200 | -1,14 | 8,85E-110 | AXS2 | UDP-D-apiose/UDP-D-xylose synthase 2 [Source:UniProtKB/Swiss-Prot;Acc:Q9SGE0] |
| AT1G08210 | 1,48 | 8,21E-78 | - | Eukaryotic aspartyl protease family protein [Source:TAIR;Acc:AT1G08210] |
| AT1G08270 | 0,89 | 2,38E-07 | - | CONTAINS InterPro DOMAIN/s: MIT (InterPro:IPR007330); BEST Arabidopsis thaliana protein match is: AAA-type ATPase family protein (TAIR:AT2G27600.1); Ha. [Source:TAIR;Acc:AT1G08270] |
| AT1G08293 | 1,27 | 1,09E-15 | - | - |
| AT1G08300 | 1,22 | 2,76E-38 | NVL | No vein-like protein [Source:UniProtKB/TrEMBL;Acc:F4HW09] |
| AT1G08310 | -1,22 | 1,51E-17 | - | Alpha/beta-Hydrolases superfamily protein [Source:UniProtKB/TrEMBL;Acc:A8MRK8] |
| AT1G08340 | -0,83 | 7,78E-08 | ROPGAP5 | Rho GTPase-activating protein 5 [Source:UniProtKB/Swiss-Prot;Acc:Q6NKT5] |
| AT1G08360 | -0,84 | 9,45E-127 | RPL10AA | 60S ribosomal protein L10a-1 [Source:UniProtKB/Swiss-Prot;Acc:Q8VZB9] |
| AT1G08380 | -1,83 | 1,86E-02 | PSAO | PSAO [Source:UniProtKB/TrEMBL;Acc:A0A178W5Y5] |
| AT1G08413 | 2,93 | 6,67E-04 | - | - |
| AT1G08430 | 1,80 | 5,70E-31 | ALMT1 | Aluminum-activated malate transporter 1 [Source:UniProtKB/Swiss-Prot;Acc:Q9SJE9] |
| AT1G08460 | 0,93 | 5,83E-34 | HDA8 | Histone deacetylase 8 [Source:UniProtKB/Swiss-Prot;Acc:Q94EJ2] |
| AT1G08470 | -0,65 | 1,44E-22 | SSL3 | SSL3 [Source:UniProtKB/TrEMBL;Acc:A0A178WK70] |
| AT1G08500 | -1,80 | 8,62E-52 | ENODL18 | Early nodulin-like protein 18 [Source:UniProtKB/TrEMBL;Acc:O82083] |
| AT1G08550 | 1,24 | 8,14E-21 | VDE1 | NPQ1 [Source:UniProtKB/TrEMBL;Acc:A0A384K8V4] |
| AT1G08560 | -0,69 | 8,33E-29 | KN | SYP111 [Source:UniProtKB/TrEMBL;Acc:A0A178WAC8] |
| AT1G08590 | -1,27 | 3,46E-14 | PXL1 | Leucine-rich repeat receptor-like protein kinase PXL1 [Source:UniProtKB/Swiss-Prot;Acc:Q9FRS6] |
| AT1G08610 | 0,97 | 6,84E-20 | - | Pentatricopeptide repeat-containing protein At1g08610 [Source:UniProtKB/Swiss-Prot;Acc:Q9FRS4] |
| AT1G08630 | 2,39 | 9,13E-13 | THA1 | Probable low-specificity L-threonine aldolase 1 [Source:UniProtKB/Swiss-Prot;Acc:Q8RXU4] |
| AT1G08643 | -3,61 | 2,10E-02 | - | - |
| AT1G08650 | -1,01 | 7,56E-23 | PPCK1 | Phosphoenolpyruvate carboxylase kinase 1 [Source:UniProtKB/Swiss-Prot;Acc:Q9SPK4] |
| AT1G08710 | 0,58 | 3,91E-09 | SKIP24 | F-box protein SKIP24 [Source:UniProtKB/Swiss-Prot;Acc:Q9CAZ0] |
| AT1G08733 | 1,58 | 1,99E-02 | - | - |
| AT1G08780 | -1,08 | 9,68E-89 | AIP3 | Probable prefoldin subunit 4 [Source:UniProtKB/Swiss-Prot;Acc:Q9M4B5] |
| AT1G08880 | -0,74 | 9,62E-98 | HTA5 | Histone H2A [Source:UniProtKB/TrEMBL;Acc:A0A178W8H7] |
| AT1G08900 | -1,69 | 8,71E-04 | SUGTL3 | Sugar transporter ERD6-like 2 [Source:UniProtKB/Swiss-Prot;Acc:Q4F7G0] |
| AT1G08930 | 0,81 | 7,18E-28 | ERD6 | ERD6 [Source:UniProtKB/TrEMBL;Acc:A0A178W9Q9] |
| AT1G08940 | 0,81 | 5,95E-23 | - | Phosphoglycerate mutase-like protein AT74H [Source:UniProtKB/Swiss-Prot;Acc:O04035] |
| AT1G08980 | 1,86 | 2,10E-06 | AMI1 | Amidase 1 [Source:UniProtKB/Swiss-Prot;Acc:Q9FR37] |
| AT1G08990 | -2,97 | 1,93E-11 | GUX5 | Putative UDP-glucuronate:xylan alpha-glucuronosyltransferase 5 [Source:UniProtKB/Swiss-Prot;Acc:F4HZC3] |
| AT1G09080 | -3,71 | 2,25E-05 | MED37B | Probable mediator of RNA polymerase II transcription subunit 37b [Source:UniProtKB/Swiss-Prot;Acc:Q8H1B3] |
| AT1G09090 | -1,53 | 1,07E-05 | RBOHB | Respiratory burst oxidase homolog protein B [Source:UniProtKB/Swiss-Prot;Acc:Q9SBI0] |
| AT1G09140 | 1,41 | 1,59E-155 | SR30 | Serine/arginine-rich splicing factor SR30 [Source:UniProtKB/Swiss-Prot;Acc:Q9XFR5] |
| AT1G09190 | 0,65 | 4,38E-10 | PCMP-E70 | Pentatricopeptide repeat-containing protein At1g09190 [Source:UniProtKB/Swiss-Prot;Acc:O80488] |
| AT1G09200 | -1,49 | 1,30E-266 | HTR2 | Histone H3 [Source:UniProtKB/TrEMBL;Acc:Q0WRA9] |
| AT1G09210 | -1,28 | 2,28E-238 | CRT2 | Calreticulin-2 [Source:UniProtKB/Swiss-Prot;Acc:Q38858] |
| AT1G09220 | 0,89 | 8,87E-08 | PCMP-E25 | Pentatricopeptide repeat-containing protein At1g09220, mitochondrial [Source:UniProtKB/Swiss-Prot;Acc:Q680Z7] |
| AT1G09260 | 0,75 | 2,76E-02 | - | Chaperone DnaJ-domain superfamily protein [Source:UniProtKB/TrEMBL;Acc:O80481] |
| AT1G09270 | -0,77 | 1,07E-71 | IMPA4 | Importin subunit alpha-4 [Source:UniProtKB/Swiss-Prot;Acc:O80480] |
| AT1G09300 | 1,16 | 9,08E-86 | ICP55 | Intermediate cleaving peptidase 55, mitochondrial [Source:UniProtKB/Swiss-Prot;Acc:F4HZG9] |
| AT1G09330 | -0,88 | 2,31E-81 | ECH | Golgi apparatus membrane protein-like protein ECHIDNA [Source:UniProtKB/Swiss-Prot;Acc:Q8LEK2] |
| AT1G09350 | -4,80 | 1,36E-03 | GOLS3 | Hexosyltransferase (Fragment) [Source:UniProtKB/TrEMBL;Acc:W8PVD7] |
| AT1G09390 | -1,34 | 5,16E-19 | - | GDSL esterase/lipase At1g09390 [Source:UniProtKB/Swiss-Prot;Acc:O80522] |
| AT1G09400 | 2,55 | 2,35E-03 | - | Putative 12-oxophytodienoate reductase-like protein 1 [Source:UniProtKB/Swiss-Prot;Acc:Q8GYA3] |
| AT1G09410 | 0,97 | 1,98E-12 | PCMP-H18 | Pentatricopeptide repeat-containing protein At1g09410, mitochondrial [Source:UniProtKB/Swiss-Prot;Acc:Q56XI1] |
| AT1G09415 | -0,65 | 1,22E-03 | NIMIN-3 | Protein NIM1-INTERACTING 3 [Source:UniProtKB/Swiss-Prot;Acc:Q9FNZ4] |
| AT1G09427 | 1,96 | 1,16E-02 | - | - |
| AT1G09450 | -0,67 | 2,39E-10 | HASPIN | Serine/threonine-protein kinase haspin homolog [Source:UniProtKB/Swiss-Prot;Acc:O80528] |
| AT1G09480 | 1,17 | 7,51E-06 | - | NAD(P)-binding Rossmann-fold superfamily protein [Source:UniProtKB/TrEMBL;Acc:F4I0Z5] |
| AT1G09490 | 0,70 | 9,04E-19 | - | At1g09490/F14J9_15 [Source:UniProtKB/TrEMBL;Acc:O80532] |
| AT1G09500 | 3,72 | 3,02E-26 | - | At1g09500/F14J9_16 [Source:UniProtKB/TrEMBL;Acc:O80533] |
| AT1G09520 | 0,72 | 1,81E-22 | - | At1g09520/F14J9_18 [Source:UniProtKB/TrEMBL;Acc:O80535] |
| AT1G09540 | -1,39 | 3,52E-11 | MYB61 | MYB61 [Source:UniProtKB/TrEMBL;Acc:A0A178WLE4] |
| AT1G09553 | 3,54 | 2,86E-02 | - | - |
| AT1G09573 | -1,97 | 3,50E-03 | - | - |
| AT1G09580 | -0,89 | 1,47E-83 | - | Transmembrane emp24 domain-containing protein p24delta3 [Source:UniProtKB/Swiss-Prot;Acc:Q6IDL4] |
| AT1G09590 | -1,07 | 8,91E-136 | RPL21A | 60S ribosomal protein L21-1 [Source:UniProtKB/Swiss-Prot;Acc:Q43291] |
| AT1G09630 | -1,29 | 1,32E-120 | RABA2A | Ras-related protein RABA2a [Source:UniProtKB/Swiss-Prot;Acc:O04486] |
| AT1G09663 | 1,41 | 2,34E-02 | - | - |
| AT1G09680 | 0,82 | 1,03E-11 | - | Putative pentatricopeptide repeat-containing protein At1g09680 [Source:UniProtKB/Swiss-Prot;Acc:O04491] |
| AT1G09690 | -0,71 | 1,05E-79 | RPL21A | 60S ribosomal protein L21-1 [Source:UniProtKB/Swiss-Prot;Acc:Q43291] |
| AT1G09700 | 0,74 | 7,30E-28 | DRB1 | Double-stranded RNA-binding protein 1 [Source:UniProtKB/Swiss-Prot;Acc:O04492] |
| AT1G09710 | 1,57 | 2,03E-46 | - | Homeodomain-like superfamily protein [Source:UniProtKB/TrEMBL;Acc:F4I129] |
| AT1G09740 | 0,81 | 5,75E-22 | - | Adenine nucleotide alpha hydrolases-like superfamily protein [Source:UniProtKB/TrEMBL;Acc:Q84TF6] |
| AT1G09750 | -2,85 | 2,02E-129 | AED3 | Aspartyl protease AED3 [Source:UniProtKB/Swiss-Prot;Acc:O04496] |
| AT1G09777 | -3,39 | 4,99E-02 | - | - |
| AT1G09780 | -1,65 | 9,37E-275 | PGM1 | IPGAM1 [Source:UniProtKB/TrEMBL;Acc:A0A178W6V9] |
| AT1G09790 | -3,61 | 1,53E-03 | COBL6 | COBRA-like protein 6 [Source:UniProtKB/Swiss-Prot;Acc:O04500] |
| AT1G09810 | 0,65 | 3,08E-18 | ECT11 | Evolutionarily conserved C-terminal region 11 [Source:UniProtKB/TrEMBL;Acc:Q56XH8] |
| AT1G09863 | 0,63 | 9,35E-04 | - | - |
| AT1G09867 | -1,48 | 2,84E-15 | - | - |
| AT1G09880 | -1,03 | 9,43E-05 | - | Rhamnogalacturonate lyase family protein [Source:TAIR;Acc:AT1G09880] |
| AT1G09910 | 0,96 | 1,99E-31 | - | Rhamnogalacturonate lyase family protein [Source:UniProtKB/TrEMBL;Acc:F4I2M8] |
| AT1G09950 | 1,36 | 4,11E-06 | RAS1 | At1g09950 [Source:UniProtKB/TrEMBL;Acc:O04515] |
| AT1G09960 | 0,59 | 3,43E-15 | SUC4 | Sucrose transport protein SUC4 [Source:UniProtKB/Swiss-Prot;Acc:Q9FE59] |
| AT1G10030 | -1,22 | 3,64E-107 | ERG28 | Ergosterol biosynthetic protein 28 [Source:UniProtKB/Swiss-Prot;Acc:O80594] |
| AT1G10140 | 1,08 | 1,14E-04 | - | At1g10140 [Source:UniProtKB/TrEMBL;Acc:Q9SY56] |
| AT1G10170 | 2,45 | 0,00E+00 | ATNFXL1 | NF-X-like 1 [Source:TAIR;Acc:AT1G10170] |
| AT1G10225 | 3,40 | 1,98E-02 | - | Transmembrane protein [Source:UniProtKB/TrEMBL;Acc:Q56YG0] |
| AT1G10230 | 0,81 | 1,56E-36 | ASK18 | SKP1-like protein 18 [Source:UniProtKB/Swiss-Prot;Acc:Q9SY65] |
| AT1G10240 | 0,62 | 3,72E-17 | FRS11 | Protein FAR1-RELATED SEQUENCE 11 [Source:UniProtKB/Swiss-Prot;Acc:Q9SY66] |
| AT1G10280 | -0,65 | 1,72E-11 | - | At1g10280 [Source:UniProtKB/TrEMBL;Acc:Q9SY70] |
| AT1G10290 | -0,72 | 3,17E-61 | DRP2A | DRP2A [Source:UniProtKB/TrEMBL;Acc:A0A178WBB3] |
| AT1G10300 | 0,96 | 5,14E-54 | - | Nucleolar GTP-binding protein 1 [Source:UniProtKB/TrEMBL;Acc:F4I4A6] |
| AT1G10330 | 0,59 | 2,45E-04 | PCMP-E71 | Putative pentatricopeptide repeat-containing protein At1g10330 [Source:UniProtKB/Swiss-Prot;Acc:Q9SY75] |
| AT1G10360 | 0,61 | 1,02E-02 | GSTU18 | Glutathione S-transferase U18 [Source:UniProtKB/Swiss-Prot;Acc:Q9FUS9] |
| AT1G10370 | 0,85 | 5,92E-27 | GSTU17 | Glutathione S-transferase U17 [Source:UniProtKB/Swiss-Prot;Acc:Q9FUS8] |
| AT1G10380 | 1,81 | 6,47E-14 | - | At1g10380/F14N23_32 [Source:UniProtKB/TrEMBL;Acc:Q93ZB0] |
| AT1G10385 | -6,34 | 4,36E-08 | EXO84A | Exocyst complex component EXO84A [Source:UniProtKB/Swiss-Prot;Acc:F4I4B6] |
| AT1G10400 | -0,66 | 2,81E-09 | UGT90A2 | Glycosyltransferase (Fragment) [Source:UniProtKB/TrEMBL;Acc:W8Q3T6] |
| AT1G10410 | 0,96 | 1,87E-65 | - | At1g10410/F14N23_31 [Source:UniProtKB/TrEMBL;Acc:Q8LPT2] |
| AT1G10430 | -0,84 | 1,45E-49 | PP2A2 | Serine/threonine-protein phosphatase PP2A-2 catalytic subunit [Source:UniProtKB/Swiss-Prot;Acc:Q07098] |
| AT1G10470 | -0,66 | 1,76E-27 | ARR4 | Two-component response regulator ARR4 [Source:UniProtKB/Swiss-Prot;Acc:O82798] |
| AT1G10480 | -2,21 | 8,40E-06 | ZFP5 | Zinc finger protein 5 [Source:UniProtKB/Swiss-Prot;Acc:Q39264] |
| AT1G10520 | 0,65 | 6,47E-10 | POLL | DNA polymerase lambda [Source:UniProtKB/Swiss-Prot;Acc:Q9FNY4] |
| AT1G10522 | 1,01 | 3,11E-38 | PRIN2 | Protein PLASTID REDOX INSENSITIVE 2, chloroplastic [Source:UniProtKB/Swiss-Prot;Acc:Q9XIK0] |
| AT1G10540 | 1,92 | 6,59E-09 | NAT8 | Nucleobase-ascorbate transporter 8 [Source:UniProtKB/Swiss-Prot;Acc:Q8VZQ5] |
| AT1G10550 | -1,44 | 2,96E-02 | XTH33 | Probable xyloglucan endotransglucosylase/hydrolase protein 33 [Source:UniProtKB/Swiss-Prot;Acc:Q8LC45] |
| AT1G10580 | -0,66 | 1,13E-35 | - | At1g10580 [Source:UniProtKB/TrEMBL;Acc:A4FVN8] |
| AT1G10600 | 1,03 | 3,49E-23 | AMSH2 | AMSH2 [Source:UniProtKB/TrEMBL;Acc:A0A178W3T5] |
| AT1G10630 | -0,70 | 4,41E-79 | ATARFA1F | ADP-ribosylation factor A1F [Source:UniProtKB/TrEMBL;Acc:Q6ID97] |
| AT1G10640 | -7,48 | 2,04E-12 | - | Pectin lyase-like superfamily protein [Source:UniProtKB/TrEMBL;Acc:F4I5U6] |
| AT1G10660 | 0,82 | 1,32E-28 | - | AT1G10660 protein [Source:UniProtKB/TrEMBL;Acc:Q8H101] |
| AT1G10670 | -0,67 | 1,70E-63 | ACLA-1 | ATP-citrate lyase A-1 [Source:UniProtKB/TrEMBL;Acc:F4I5V8] |
| AT1G10682 | -1,25 | 2,65E-30 | - | other RNA [Source:TAIR;Acc:AT1G10682] |
| AT1G10730 | 0,82 | 2,04E-17 | AP1M1 | AP-1 complex subunit mu-1 [Source:UniProtKB/Swiss-Prot;Acc:Q9SAC9] |
| AT1G10750 | 0,84 | 1,26E-13 | - | Carboxyl-terminal peptidase, putative (DUF239) [Source:UniProtKB/TrEMBL;Acc:Q84JS7] |
| AT1G10770 | 2,07 | 1,02E-04 | - | At1g10770 [Source:UniProtKB/TrEMBL;Acc:Q9SAC5] |
| AT1G10800 | 1,91 | 1,04E-02 | - | F20B24.22 [Source:UniProtKB/TrEMBL;Acc:Q9SGX2] |
| AT1G10810 | -1,54 | 1,88E-31 | - | Probable aldo-keto reductase 1 [Source:UniProtKB/Swiss-Prot;Acc:Q9C5B9] |
| AT1G10870 | -0,80 | 2,37E-55 | AGD4 | ARF-GAP domain 4 [Source:TAIR;Acc:AT1G10870] |
| AT1G10950 | -0,71 | 1,18E-95 | TMN1 | Transmembrane 9 superfamily member 1 [Source:UniProtKB/Swiss-Prot;Acc:Q940G0] |
| AT1G10980 | 2,55 | 5,11E-03 | - | Expressed protein [Source:UniProtKB/TrEMBL;Acc:O04088] |
| AT1G10990 | 0,63 | 7,56E-09 | - | unknown protein; Ha. [Source:TAIR;Acc:AT1G10990] |
| AT1G11020 | 0,98 | 1,11E-30 | - | RING/FYVE/PHD zinc finger superfamily protein [Source:UniProtKB/TrEMBL;Acc:Q8GYT4] |
| AT1G11100 | 1,66 | 3,72E-36 | - | SNF2 domain-containing protein / helicase domain-containing protein / zinc finger protein-like protein (Fragment) [Source:UniProtKB/TrEMBL;Acc:C0SUU4] |
| AT1G11170 | 1,58 | 2,45E-18 | - | At1g11170/T28P6_16 [Source:UniProtKB/TrEMBL;Acc:Q8S9J5] |
| AT1G11175 | 2,03 | 3,53E-14 | - | other RNA [Source:TAIR;Acc:AT1G11175] |
| AT1G11180 | 0,63 | 9,62E-03 | - | Secretory carrier membrane protein (SCAMP) family protein [Source:TAIR;Acc:AT1G11180] |
| AT1G11185 | -1,36 | 5,27E-03 | - | other RNA [Source:TAIR;Acc:AT1G11185] |
| AT1G11220 | -0,99 | 5,99E-12 | - | Cotton fiber, putative (DUF761) [Source:UniProtKB/TrEMBL;Acc:Q9SXA8] |
| AT1G11260 | -0,85 | 5,80E-29 | STP1 | STP1 [Source:UniProtKB/TrEMBL;Acc:A0A178WJ63] |
| AT1G11270 | 1,06 | 1,09E-24 | - | F-box protein At1g11270 [Source:UniProtKB/Swiss-Prot;Acc:Q7X7A9] |
| AT1G11290 | 0,75 | 2,71E-11 | PCMP-H40 | CRR22 [Source:UniProtKB/TrEMBL;Acc:A0A178WFR8] |
| AT1G11330 | 1,46 | 1,75E-24 | - | G-type lectin S-receptor-like serine/threonine-protein kinase At1g11330 [Source:UniProtKB/Swiss-Prot;Acc:Q9SXB8] |
| AT1G11400 | 0,89 | 6,04E-61 | PYM | PYM [Source:UniProtKB/TrEMBL;Acc:A0A178W3L1] |
| AT1G11420 | 0,61 | 5,85E-20 | DUF2 | DUF724 domain-containing protein 2 [Source:UniProtKB/Swiss-Prot;Acc:F4I8W1] |
| AT1G11490 | -2,28 | 4,57E-19 | - | Zinc finger (C2H2 type) family protein (Fragment) [Source:UniProtKB/TrEMBL;Acc:C0SUU5] |
| AT1G11545 | -1,08 | 3,37E-85 | XTH8 | Xyloglucan endotransglucosylase/hydrolase [Source:UniProtKB/TrEMBL;Acc:A0A178W0W2] |
| AT1G11580 | -1,83 | 0,00E+00 | ATPMEPCRA | methylesterase PCR A [Source:TAIR;Acc:AT1G11580] |
| AT1G11630 | 0,70 | 1,72E-16 | - | Pentatricopeptide repeat-containing protein At1g11630, mitochondrial [Source:UniProtKB/Swiss-Prot;Acc:Q9SAB4] |
| AT1G11655 | -5,77 | 1,46E-06 | - | At1g11655 [Source:UniProtKB/TrEMBL;Acc:Q84VW6] |
| AT1G11700 | -1,58 | 4,18E-05 | - | At1g11700 [Source:UniProtKB/TrEMBL;Acc:Q9SAA7] |
| AT1G11730 | 0,98 | 4,45E-03 | B3GALT1 | Hexosyltransferase (Fragment) [Source:UniProtKB/TrEMBL;Acc:W8Q7D4] |
| AT1G11800 | 0,63 | 4,84E-09 | - | At1g11800/F25C20_3 [Source:UniProtKB/TrEMBL;Acc:Q9SA95] |
| AT1G11860 | -1,05 | 2,25E-115 | - | Glycine cleavage T-protein family [Source:TAIR;Acc:AT1G11860] |
| AT1G11890 | -1,02 | 5,70E-111 | SEC22 | SEC22 [Source:UniProtKB/TrEMBL;Acc:A0A178WBU4] |
| AT1G12000 | -0,69 | 2,03E-66 | PFP-BETA1 | Pyrophosphate--fructose 6-phosphate 1-phosphotransferase subunit beta 1 [Source:UniProtKB/Swiss-Prot;Acc:Q8W4M5] |
| AT1G12040 | -2,01 | 2,42E-08 | LRX1 | Leucine-rich repeat extensin-like protein 1 [Source:UniProtKB/Swiss-Prot;Acc:O65375] |
| AT1G12060 | 1,04 | 3,94E-07 | BAG5 | BAG5 [Source:UniProtKB/TrEMBL;Acc:A0A178WFD4] |
| AT1G12080 | -0,65 | 2,71E-04 | - | At1g12080 [Source:UniProtKB/TrEMBL;Acc:O65370] |
| AT1G12090 | -1,20 | 1,83E-93 | ELP | ELP [Source:UniProtKB/TrEMBL;Acc:A0A178W1A4] |
| AT1G12100 | 0,69 | 1,56E-02 | - | Bifunctional inhibitor/lipid-transfer protein/seed storage 2S albumin superfamily protein [Source:UniProtKB/TrEMBL;Acc:F4IC43] |
| AT1G12110 | 1,11 | 3,52E-39 | NPF6.3 | NRT1.1 [Source:UniProtKB/TrEMBL;Acc:A0A178W8F7] |
| AT1G12130 | 1,09 | 1,26E-03 | - | Flavin-containing monooxygenase FMO GS-OX-like 6 [Source:UniProtKB/Swiss-Prot;Acc:Q9FWW3] |
| AT1G12200 | 1,58 | 7,02E-13 | - | Flavin-containing monooxygenase [Source:UniProtKB/TrEMBL;Acc:A0A178W1K3] |
| AT1G12230 | -0,62 | 2,84E-70 | - | Aldolase superfamily protein [Source:UniProtKB/TrEMBL;Acc:F4IC59] |
| AT1G12240 | -0,78 | 1,87E-75 | BFRUCT4 | Acid beta-fructofuranosidase 4, vacuolar [Source:UniProtKB/Swiss-Prot;Acc:Q39041] |
| AT1G12280 | 1,55 | 1,49E-37 | SUMM2 | Disease resistance protein SUMM2 [Source:UniProtKB/Swiss-Prot;Acc:P60838] |
| AT1G12290 | 1,05 | 2,02E-14 | - | Probable disease resistance protein At1g12290 [Source:UniProtKB/Swiss-Prot;Acc:P60839] |
| AT1G12330 | 1,28 | 1,12E-04 | - | Cyclin-dependent kinase-like protein [Source:UniProtKB/TrEMBL;Acc:Q9LNB2] |
| AT1G12440 | 2,26 | 0,00E+00 | SAP1 | Zinc finger A20 and AN1 domain-containing stress-associated protein 1 [Source:UniProtKB/Swiss-Prot;Acc:Q6NNI8] |
| AT1G12460 | 0,58 | 1,31E-16 | - | Probable LRR receptor-like serine/threonine-protein kinase At1g12460 [Source:UniProtKB/Swiss-Prot;Acc:C0LGE4] |
| AT1G12500 | -1,04 | 3,33E-84 | - | Probable sugar phosphate/phosphate translocator At1g12500 [Source:UniProtKB/Swiss-Prot;Acc:Q9LDH3] |
| AT1G12560 | -4,87 | 3,11E-03 | EXPA7 | Expansin [Source:UniProtKB/TrEMBL;Acc:A0A178W749] |
| AT1G12570 | 1,94 | 9,71E-03 | - | At1g12570 [Source:UniProtKB/TrEMBL;Acc:Q66GI5] |
| AT1G12600 | -0,90 | 3,53E-11 | UTR4 | UDP-galactose/UDP-glucose transporter 4 [Source:UniProtKB/Swiss-Prot;Acc:Q9LDX3] |
| AT1G12630 | -2,04 | 3,52E-02 | ERF027 | Ethylene-responsive transcription factor ERF027 [Source:UniProtKB/Swiss-Prot;Acc:Q38Q39] |
| AT1G12730 | 0,58 | 3,67E-09 | - | GPI transamidase subunit PIG-U [Source:UniProtKB/TrEMBL;Acc:Q94K70] |
| AT1G12750 | 0,80 | 1,20E-17 | RBL6 | RHOMBOID-like protein 6, mitochondrial [Source:UniProtKB/Swiss-Prot;Acc:Q8VZ48] |
| AT1G12840 | -0,58 | 8,86E-55 | VHA-C | V-type proton ATPase subunit C [Source:UniProtKB/Swiss-Prot;Acc:Q9SDS7] |
| AT1G12850 | -0,95 | 3,37E-88 | - | At1g12850/F13K23_8 [Source:UniProtKB/TrEMBL;Acc:Q94BZ6] |
| AT1G12860 | 0,80 | 5,06E-04 | SCRM2 | Transcription factor SCREAM2 [Source:UniProtKB/Swiss-Prot;Acc:Q9LPW3] |
| AT1G12890 | 2,69 | 3,04E-02 | ERF088 | Ethylene-responsive transcription factor ERF088 [Source:UniProtKB/Swiss-Prot;Acc:Q3E703] |
| AT1G12900 | -2,83 | 1,20E-04 | GAPA-2 | glyceraldehyde 3-phosphate dehydrogenase A subunit 2 [Source:TAIR;Acc:AT1G12900] |
| AT1G12950 | -3,75 | 2,15E-05 | DTX31 | Protein DETOXIFICATION [Source:UniProtKB/TrEMBL;Acc:A0A178WCP4] |
| AT1G12990 | -0,63 | 5,73E-11 | - | Beta-1,4-N-acetylglucosaminyltransferase family protein [Source:UniProtKB/TrEMBL;Acc:F4HP06] |
| AT1G13000 | -0,84 | 2,48E-58 | - | Transmembrane protein, putative (DUF707) [Source:UniProtKB/TrEMBL;Acc:Q8VYF6] |
| AT1G13020 | -0,78 | 4,03E-56 | EIF4B2 | eIF4B2 [Source:UniProtKB/TrEMBL;Acc:A0A178W345] |
| AT1G13060 | -0,72 | 3,41E-97 | PBE1 | 20S proteasome beta subunit E1 [Source:UniProtKB/TrEMBL;Acc:F4HP14] |
| AT1G13110 | -1,74 | 8,91E-12 | CYP71B7 | CYP71B7 [Source:UniProtKB/TrEMBL;Acc:A0A178WGC6] |
| AT1G13150 | 1,42 | 5,34E-13 | CYP86C4 | Cytochrome P450, family 86, subfamily C, polypeptide 4 [Source:UniProtKB/TrEMBL;Acc:Q9SAE8] |
| AT1G13170 | -0,88 | 4,65E-41 | ORP1D | OSBP(Oxysterol binding protein)-related protein 1D [Source:UniProtKB/TrEMBL;Acc:F4HP28] |
| AT1G13180 | -0,67 | 3,74E-22 | ARP3 | Actin-related protein 3 [Source:UniProtKB/Swiss-Prot;Acc:Q9SAF1] |
| AT1G13245 | 2,02 | 1,02E-04 | RTFL17 | At1g13245 [Source:UniProtKB/TrEMBL;Acc:Q9SAF8] |
| AT1G13250 | -1,91 | 2,37E-09 | GATL3 | Probable galacturonosyltransferase-like 3 [Source:UniProtKB/Swiss-Prot;Acc:Q0V7R1] |
| AT1G13280 | -1,07 | 9,24E-161 | AOC4 | Allene oxide cyclase 4, chloroplastic [Source:UniProtKB/Swiss-Prot;Acc:Q93ZC5] |
| AT1G13300 | -3,49 | 5,57E-49 | HRS1 | Transcription factor HRS1 [Source:UniProtKB/Swiss-Prot;Acc:Q9FX67] |
| AT1G13330 | 0,72 | 2,63E-07 | HOP2 | Homologous-pairing protein 2 homolog [Source:UniProtKB/Swiss-Prot;Acc:Q9FX64] |
| AT1G13360 | 1,87 | 3,96E-113 | - | T6J4.11 protein [Source:UniProtKB/TrEMBL;Acc:Q9FX61] |
| AT1G13390 | 0,70 | 3,24E-24 | - | At1g13390 [Source:UniProtKB/TrEMBL;Acc:Q9FX58] |
| AT1G13430 | -1,23 | 1,63E-05 | STO9 | Cytosolic sulfotransferase 9 [Source:UniProtKB/Swiss-Prot;Acc:Q9FX55] |
| AT1G13480 | -2,26 | 1,88E-15 | - | At1g13480 [Source:UniProtKB/TrEMBL;Acc:Q56Y29] |
| AT1G13500 | -6,48 | 1,66E-07 | - | Protein of unknown function (DUF1262) [Source:TAIR;Acc:AT1G13500] |
| AT1G13510 | -6,80 | 2,01E-14 | - | Uncharacterized protein At1g13510 [Source:UniProtKB/TrEMBL;Acc:Q8VZN5] |
| AT1G13530 | -0,67 | 2,78E-08 | - | Protein of unknown function (DUF1262) [Source:TAIR;Acc:AT1G13530] |
| AT1G13540 | -3,09 | 1,63E-07 | - | At1g13540 [Source:UniProtKB/TrEMBL;Acc:Q6NM24] |
| AT1G13570 | 0,64 | 2,59E-26 | - | F-box/FBD/LRR-repeat protein At1g13570 [Source:UniProtKB/Swiss-Prot;Acc:Q9FZ70] |
| AT1G13610 | 1,80 | 1,52E-03 | - | Alpha/beta-Hydrolases superfamily protein [Source:UniProtKB/TrEMBL;Acc:Q9FZ68] |
| AT1G13630 | 0,95 | 1,73E-19 | - | Tetratricopeptide repeat (TPR)-like superfamily protein [Source:TAIR;Acc:AT1G13630] |
| AT1G13635 | 4,17 | 1,36E-02 | - | DNA glycosylase superfamily protein [Source:UniProtKB/TrEMBL;Acc:F4HSJ2] |
| AT1G13640 | 0,75 | 1,20E-39 | PI4KG6 | Phosphatidylinositol 4-kinase gamma 6 [Source:UniProtKB/Swiss-Prot;Acc:Q8W4R8] |
| AT1G13730 | -0,62 | 3,34E-40 | - | At1g13730 [Source:UniProtKB/TrEMBL;Acc:Q9LMX6] |
| AT1G13790 | 2,45 | 1,25E-12 | FDM4 | Factor of DNA methylation 4 [Source:UniProtKB/Swiss-Prot;Acc:Q9LMH6] |
| AT1G13800 | 1,08 | 1,06E-26 | - | Putative pentatricopeptide repeat-containing protein At1g13800 [Source:UniProtKB/Swiss-Prot;Acc:Q9LMH5] |
| AT1G13810 | 0,72 | 4,38E-25 | - | Restriction endonuclease, type II-like superfamily protein [Source:UniProtKB/TrEMBL;Acc:Q5XVK9] |
| AT1G13820 | 1,32 | 5,39E-28 | - | Alpha/beta-Hydrolases superfamily protein [Source:UniProtKB/TrEMBL;Acc:Q6NL07] |
| AT1G13860 | -0,65 | 7,98E-18 | QUL1 | Probable methyltransferase PMT4 [Source:UniProtKB/Swiss-Prot;Acc:Q8GYW9] |
| AT1G13990 | 1,04 | 4,57E-44 | - | unknown protein; FUNCTIONS IN: molecular_function unknown; INVOLVED IN: biological_process unknown; LOCATED IN: chloroplast; EXPRESSED IN: 23 plant structures; EXPRESSED DURING: 13 growth stages; CONTAINS InterPro DOMAIN/s: Protein of unknown functi /.../3110 (InterPro:IPR021503); Ha. [Source:TAIR;Acc:AT1G13990] |
| AT1G14010 | -0,79 | 3,36E-87 | - | Transmembrane emp24 domain-containing protein p24delta7 [Source:UniProtKB/Swiss-Prot;Acc:Q8GYG1] |
| AT1G14020 | -1,03 | 3,93E-125 | OFUT3 | O-fucosyltransferase 3 [Source:UniProtKB/Swiss-Prot;Acc:Q6NQ51] |
| AT1G14170 | -1,29 | 1,91E-37 | - | F7A19.25 protein [Source:UniProtKB/TrEMBL;Acc:Q9XI71] |
| AT1G14200 | 3,31 | 3,06E-56 | - | At1g14200 [Source:UniProtKB/TrEMBL;Acc:Q9XI67] |
| AT1G14220 | -2,56 | 3,53E-120 | - | F7A19.32 protein [Source:UniProtKB/TrEMBL;Acc:Q9XI64] |
| AT1G14230 | -1,12 | 3,36E-90 | APY4 | Probable apyrase 4 [Source:UniProtKB/Swiss-Prot;Acc:Q8H1D8] |
| AT1G14240 | -2,96 | 1,09E-26 | APY3 | Probable apyrase 3 [Source:UniProtKB/Swiss-Prot;Acc:Q9XI62] |
| AT1G14260 | -1,95 | 1,18E-10 | - | At1g14260 [Source:UniProtKB/TrEMBL;Acc:Q4PS45] |
| AT1G14280 | -2,03 | 4,79E-42 | PKS2 | Protein PHYTOCHROME KINASE SUBSTRATE 2 [Source:UniProtKB/Swiss-Prot;Acc:Q9M9T4] |
| AT1G14290 | -1,22 | 3,63E-59 | SBH2 | SBH2 [Source:UniProtKB/TrEMBL;Acc:A0A178WPE2] |
| AT1G14320 | -0,83 | 4,25E-102 | RPL10A | 60S ribosomal protein L10-1 [Source:UniProtKB/Swiss-Prot;Acc:Q93VT9] |
| AT1G14370 | 0,59 | 3,88E-03 | PBL2 | PBL2 [Source:UniProtKB/TrEMBL;Acc:A0A178WI52] |
| AT1G14430 | -1,14 | 1,04E-07 | - | F14L17.20 protein [Source:UniProtKB/TrEMBL;Acc:Q9M9S1] |
| AT1G14450 | -1,05 | 1,29E-28 | - | NADH dehydrogenase (ubiquinone)s [Source:TAIR;Acc:AT1G14450] |
| AT1G14470 | 0,88 | 1,02E-09 | PCMP-A4 | Pentatricopeptide repeat-containing protein At1g14470 [Source:UniProtKB/Swiss-Prot;Acc:Q9M9R6] |
| AT1G14480 | 0,95 | 6,34E-13 | - | Ankyrin repeat family protein [Source:UniProtKB/TrEMBL;Acc:Q9M9R5] |
| AT1G14500 | -0,96 | 3,22E-05 | - | Ankyrin repeat family protein [Source:UniProtKB/TrEMBL;Acc:Q9M9R3] |
| AT1G14560 | -0,81 | 6,11E-38 | COAC1 | Mitochondrial carrier protein CoAc1 [Source:UniProtKB/Swiss-Prot;Acc:F4HW79] |
| AT1G14580 | -1,28 | 1,32E-15 | - | C2H2-like zinc finger protein [Source:TAIR;Acc:AT1G14580] |
| AT1G14620 | 0,80 | 3,89E-58 | DECOY | At1g14620/T5E21_15 [Source:UniProtKB/TrEMBL;Acc:Q8L7U3] |
| AT1G14630 | -4,49 | 1,05E-03 | - | unknown protein; BEST Arabidopsis thaliana protein match is: unknown protein (TAIR:AT2G01990.1); Ha. [Source:TAIR;Acc:AT1G14630] |
| AT1G14640 | 2,76 | 1,50E-26 | - | SWAP (Suppressor-of-White-APricot)/surp domain-containing protein [Source:UniProtKB/TrEMBL;Acc:F4HW92] |
| AT1G14642 | 2,84 | 5,78E-05 | - | unknown protein; FUNCTIONS IN: molecular_function unknown; INVOLVED IN: biological_process unknown; LOCATED IN: cellular_component unknown; Ha. [Source:TAIR;Acc:AT1G14642] |
| AT1G14670 | -0,75 | 2,16E-85 | TMN2 | Transmembrane 9 superfamily member 2 [Source:UniProtKB/Swiss-Prot;Acc:Q940S0] |
| AT1G14680 | 0,76 | 1,04E-02 | - | Early endosome antigen [Source:UniProtKB/TrEMBL;Acc:Q9LE70] |
| AT1G14688 | 5,43 | 3,76E-05 | - | FUNCTIONS IN: molecular_function unknown; INVOLVED IN: biological_process unknown; LOCATED IN: cellular_component unknown; CONTAINS InterPro DOMAIN/s: E3 Ubiquitin ligase (InterPro:IPR022170); Ha. [Source:TAIR;Acc:AT1G14688] |
| AT1G14690 | 0,75 | 2,70E-32 | MAP65-7 | 65-kDa microtubule-associated protein 7 [Source:UniProtKB/Swiss-Prot;Acc:Q8L836] |
| AT1G14730 | -1,80 | 2,28E-03 | CYB561C | Probable transmembrane ascorbate ferrireductase 3 [Source:UniProtKB/Swiss-Prot;Acc:Q67ZF6] |
| AT1G14740 | 0,77 | 1,22E-75 | OBE3 | Protein OBERON 3 [Source:UniProtKB/Swiss-Prot;Acc:Q94B71] |
| AT1G14750 | -1,73 | 3,44E-48 | SDS | Cyclin family protein [Source:TAIR;Acc:AT1G14750] |
| AT1G14780 | -0,72 | 1,29E-03 | - | MACPF domain-containing protein At1g14780 [Source:UniProtKB/Swiss-Prot;Acc:Q8L612] |
| AT1G14790 | 1,94 | 2,51E-21 | RDR1 | RNA-dependent RNA polymerase [Source:UniProtKB/TrEMBL;Acc:A0A178WKG4] |
| AT1G14830 | -0,77 | 8,24E-61 | DRP1C | DRP1C [Source:UniProtKB/TrEMBL;Acc:A0A178W4V6] |
| AT1G14860 | 0,89 | 7,52E-14 | NUDT18 | Nudix hydrolase 18, mitochondrial [Source:UniProtKB/Swiss-Prot;Acc:Q9LQU5] |
| AT1G14870 | 1,12 | 1,96E-49 | PCR2 | PCR2 [Source:UniProtKB/TrEMBL;Acc:A0A178WDU8] |
| AT1G14890 | 1,67 | 5,41E-08 | - | Plant invertase/pectin methylesterase inhibitor superfamily protein [Source:UniProtKB/TrEMBL;Acc:F4HXW0] |
| AT1G15002 | -1,42 | 2,83E-05 | - | Potential natural antisense gene, locus overlaps with AT1G15000 [Source:TAIR;Acc:AT1G15002] |
| AT1G15020 | -0,60 | 3,06E-27 | QSOX1 | Sulfhydryl oxidase 1 [Source:UniProtKB/Swiss-Prot;Acc:Q8W4J3] |
| AT1G15030 | -1,25 | 8,00E-89 | - | T15D22.8 [Source:UniProtKB/TrEMBL;Acc:Q9M9Q2] |
| AT1G15080 | -1,00 | 1,15E-04 | LPP2 | Lipid phosphate phosphatase 2 [Source:UniProtKB/Swiss-Prot;Acc:Q9XI60] |
| AT1G15085 | -0,82 | 2,63E-02 | - | - |
| AT1G15120 | -0,60 | 1,43E-53 | - | Ubiquinol-cytochrome C reductase hinge protein [Source:UniProtKB/TrEMBL;Acc:F4HXY8] |
| AT1G15140 | -0,66 | 2,34E-27 | - | F9L1.8 protein [Source:UniProtKB/TrEMBL;Acc:Q9XI55] |
| AT1G15170 | 0,78 | 1,21E-44 | DTX12 | Protein DETOXIFICATION [Source:UniProtKB/TrEMBL;Acc:A0A178WNL0] |
| AT1G15180 | 0,74 | 3,60E-09 | DTX13 | Protein DETOXIFICATION 13 [Source:UniProtKB/Swiss-Prot;Acc:Q94AL1] |
| AT1G15190 | 0,63 | 2,39E-02 | FLA19 | Fasciclin-like arabinogalactan protein 19 [Source:UniProtKB/Swiss-Prot;Acc:Q5Q0H2] |
| AT1G15210 | -1,07 | 5,17E-67 | ABCG35 | PDR7 [Source:UniProtKB/TrEMBL;Acc:A0A178WAD4] |
| AT1G15230 | 1,38 | 4,10E-81 | - | At1g15230/F9L1_18 [Source:UniProtKB/TrEMBL;Acc:Q9XI45] |
| AT1G15260 | -2,32 | 1,37E-03 | - | LOW protein: ATP-dependent RNA helicase-like protein [Source:UniProtKB/TrEMBL;Acc:Q8VY60] |
| AT1G15310 | 1,03 | 5,71E-65 | SRP-54A | Signal recognition particle 54 kDa protein [Source:UniProtKB/TrEMBL;Acc:Q56XJ0] |
| AT1G15320 | -1,85 | 2,30E-04 | - | Seed dormancy control protein [Source:UniProtKB/TrEMBL;Acc:Q1PFW1] |
| AT1G15350 | -1,26 | 1,01E-54 | - | DUF4050 family protein [Source:UniProtKB/TrEMBL;Acc:Q8L920] |
| AT1G15370 | -0,63 | 2,84E-34 | - | F9L1.32 protein [Source:UniProtKB/TrEMBL;Acc:Q9XI32] |
| AT1G15390 | 0,93 | 1,48E-69 | PDF1A | Peptide deformylase [Source:UniProtKB/TrEMBL;Acc:A0A178WDP8] |
| AT1G15460 | 2,32 | 1,81E-35 | BOR4 | BOR4 [Source:UniProtKB/TrEMBL;Acc:A0A178W6Z7] |
| AT1G15480 | 1,11 | 1,89E-119 | - | Pentatricopeptide repeat-containing protein At1g15480, mitochondrial [Source:UniProtKB/Swiss-Prot;Acc:Q9XI21] |
| AT1G15540 | 1,45 | 1,75E-02 | - | 2-oxoglutarate (2OG) and Fe(II)-dependent oxygenase superfamily protein [Source:TAIR;Acc:AT1G15540] |
| AT1G15550 | 0,63 | 3,13E-19 | GA3OX1 | Gibberellin 3-beta-dioxygenase 1 [Source:UniProtKB/Swiss-Prot;Acc:Q39103] |
| AT1G15630 | -4,31 | 4,31E-12 | - | Transmembrane protein [Source:UniProtKB/TrEMBL;Acc:Q5BQ07] |
| AT1G15640 | -5,83 | 1,58E-06 | - | T16N11.15 protein [Source:UniProtKB/TrEMBL;Acc:Q9M9D1] |
| AT1G15660 | -0,63 | 1,19E-30 | CENP-C | Centromere protein C [Source:UniProtKB/Swiss-Prot;Acc:Q66LG9] |
| AT1G15670 | 0,85 | 3,26E-26 | - | F-box/kelch-repeat protein At1g15670 [Source:UniProtKB/Swiss-Prot;Acc:Q9LMR5] |
| AT1G15690 | -1,26 | 1,30E-259 | AVP1 | VHP1 [Source:UniProtKB/TrEMBL;Acc:A0A178WA14] |
| AT1G15710 | -0,73 | 1,61E-19 | TYRAAT2 | Arogenate dehydrogenase 2, chloroplastic [Source:UniProtKB/Swiss-Prot;Acc:Q9LMR3] |
| AT1G15740 | 1,97 | 1,26E-56 | - | Leucine-rich repeat family protein [Source:UniProtKB/TrEMBL;Acc:Q8H1Q4] |
| AT1G15760 | -1,30 | 1,10E-30 | - | At1g15760 [Source:UniProtKB/TrEMBL;Acc:Q9LMQ8] |
| AT1G15772 | 3,79 | 1,52E-36 | - | unknown protein; FUNCTIONS IN: molecular_function unknown; INVOLVED IN: biological_process unknown; LOCATED IN: cellular_component unknown; BEST Arabidopsis thaliana protein match is: unknown protein (TAIR:AT1G15780.1); Ha. [Source:TAIR;Acc:AT1G15772] |
| AT1G15790 | 2,26 | 5,46E-106 | - | unknown protein; BEST Arabidopsis thaliana protein match is: unknown protein (TAIR:AT1G15780.1); Ha. [Source:TAIR;Acc:AT1G15790] |
| AT1G15800 | 1,16 | 1,41E-33 | - | At1g15800 [Source:UniProtKB/TrEMBL;Acc:Q6NL17] |
| AT1G15820 | -1,22 | 1,25E-27 | LHCB6 | Chlorophyll a-b binding protein, chloroplastic [Source:UniProtKB/TrEMBL;Acc:Q9LMQ2] |
| AT1G15870 | 1,27 | 4,06E-44 | - | At1g15870/F7H2_19 [Source:UniProtKB/TrEMBL;Acc:Q9LMP8] |
| AT1G15890 | 1,03 | 6,76E-06 | - | Probable disease resistance protein At1g15890 [Source:UniProtKB/Swiss-Prot;Acc:Q9LMP6] |
| AT1G15900 | -1,20 | 3,13E-07 | - | unknown protein; Ha. [Source:TAIR;Acc:AT1G15900] |
| AT1G15910 | 0,96 | 3,40E-87 | FDM1 | IDP1 [Source:UniProtKB/TrEMBL;Acc:A0A178W3T4] |
| AT1G15920 | 0,84 | 1,43E-39 | CAF1-2 | Probable CCR4-associated factor 1 homolog 2 [Source:UniProtKB/Swiss-Prot;Acc:Q9S9P2] |
| AT1G15940 | 1,04 | 7,64E-103 | - | T24D18.4 [Source:UniProtKB/TrEMBL;Acc:Q9S9P0] |
| AT1G16000 | -0,63 | 2,04E-38 | - | GAG1At protein [Source:UniProtKB/TrEMBL;Acc:P93048] |
| AT1G16020 | -0,74 | 2,48E-41 | CCZ1A | Vacuolar fusion protein CCZ1 homolog A [Source:UniProtKB/Swiss-Prot;Acc:F4I2S4] |
| AT1G16030 | 0,62 | 6,79E-09 | HSP70-5 | Hsp70b [Source:UniProtKB/TrEMBL;Acc:A0A178W9N7] |
| AT1G16080 | 0,61 | 2,50E-15 | - | Nuclear protein [Source:UniProtKB/TrEMBL;Acc:Q9S9M7] |
| AT1G16120 | -3,40 | 2,58E-06 | WAKL1 | Wall-associated receptor kinase-like 1 [Source:UniProtKB/Swiss-Prot;Acc:Q9S9M5] |
| AT1G16150 | -4,91 | 5,67E-19 | WAKL4 | Wall-associated receptor kinase-like 4 [Source:UniProtKB/Swiss-Prot;Acc:Q9S9M2] |
| AT1G16160 | -0,78 | 5,16E-05 | WAKL5 | WAKL5 [Source:UniProtKB/TrEMBL;Acc:A0A178W9G0] |
| AT1G16190 | -0,77 | 3,73E-54 | RAD23A | RAD23A [Source:UniProtKB/TrEMBL;Acc:A0A178W4Q3] |
| AT1G16330 | -0,69 | 3,99E-13 | CYCB3;1 | CYCB3 [Source:UniProtKB/TrEMBL;Acc:A0A178W969] |
| AT1G16360 | 2,00 | 2,06E-30 | - | LEM3 (ligand-effect modulator 3) family protein / CDC50 family protein [Source:TAIR;Acc:AT1G16360] |
| AT1G16370 | -1,87 | 4,88E-23 | 38991 | Organic cation/carnitine transporter 6 [Source:UniProtKB/Swiss-Prot;Acc:Q9SA36] |
| AT1G16430 | -0,62 | 6,01E-13 | MED22A | Mediator of RNA polymerase II transcription subunit 22a [Source:UniProtKB/Swiss-Prot;Acc:Q9SA42] |
| AT1G16500 | -0,63 | 2,64E-02 | - | >F3O9.30 [Source:UniProtKB/TrEMBL;Acc:Q9SA48] |
| AT1G16560 | -0,88 | 8,96E-91 | - | AT1G16560 protein [Source:UniProtKB/TrEMBL;Acc:Q9FX73] |
| AT1G16630 | -0,91 | 2,65E-30 | - | At1g16630 [Source:UniProtKB/TrEMBL;Acc:Q9FX76] |
| AT1G16670 | 0,63 | 1,24E-06 | CRPK1 | Cold-responsive protein kinase 1 [Source:UniProtKB/Swiss-Prot;Acc:Q93YN1] |
| AT1G16740 | -0,74 | 2,61E-63 | - | 50S ribosomal protein L20 [Source:UniProtKB/TrEMBL;Acc:A0A178WF80] |
| AT1G16780 | 0,89 | 4,99E-39 | AVPL2 | Pyrophosphate-energized membrane proton pump 3 [Source:UniProtKB/Swiss-Prot;Acc:Q9FWR2] |
| AT1G16850 | 1,75 | 2,89E-74 | - | At1g16880 [Source:UniProtKB/TrEMBL;Acc:Q9FE54] |
| AT1G16880 | -0,78 | 6,13E-45 | ACR11 | ACR11 [Source:UniProtKB/TrEMBL;Acc:A0A178WLF3] |
| AT1G16920 | -0,69 | 8,53E-43 | RABA1B | RABA1b [Source:UniProtKB/TrEMBL;Acc:A0A178WJV5] |
| AT1G17020 | 4,87 | 5,36E-36 | SRG1 | Protein SRG1 [Source:UniProtKB/Swiss-Prot;Acc:Q39224] |
| AT1G17090 | 1,38 | 6,66E-10 | - | At1g17090/F6I1_23 [Source:UniProtKB/TrEMBL;Acc:Q93Z61] |
| AT1G17130 | -0,89 | 5,85E-70 | - | Family of unknown function (DUF572) [Source:TAIR;Acc:AT1G17130] |
| AT1G17160 | -0,75 | 1,26E-33 | - | Ribokinase [Source:UniProtKB/TrEMBL;Acc:A1A6H3] |
| AT1G17170 | 0,96 | 6,66E-142 | GSTU24 | Glutathione S-transferase U24 [Source:UniProtKB/Swiss-Prot;Acc:Q9SHH6] |
| AT1G17190 | -2,45 | 2,36E-52 | GSTU26 | Glutathione S-transferase U26 [Source:UniProtKB/Swiss-Prot;Acc:Q9SHH8] |
| AT1G17240 | 1,09 | 4,84E-08 | AtRLP2 | receptor like protein 2 [Source:TAIR;Acc:AT1G17240] |
| AT1G17260 | 0,80 | 2,09E-07 | AHA10 | Plasma membrane ATPase [Source:UniProtKB/TrEMBL;Acc:A0A178W776] |
| AT1G17430 | -0,75 | 8,19E-16 | - | Alpha/beta-Hydrolases superfamily protein [Source:UniProtKB/TrEMBL;Acc:Q9LNR2] |
| AT1G17455 | -0,86 | 6,07E-10 | EFL4 | ELF4-L4 [Source:UniProtKB/TrEMBL;Acc:A0A178WKX5] |
| AT1G17460 | 1,20 | 2,61E-31 | TRFL3 | TRF-like 3 [Source:UniProtKB/TrEMBL;Acc:F4I7K3] |
| AT1G17530 | 1,68 | 6,12E-150 | TIM23-1 | Mitochondrial import inner membrane translocase subunit TIM23-1 [Source:UniProtKB/Swiss-Prot;Acc:Q9LNQ1] |
| AT1G17540 | 1,28 | 3,48E-02 | - | Kinase with adenine nucleotide alpha hydrolases-like domain-containing protein [Source:UniProtKB/TrEMBL;Acc:Q681A5] |
| AT1G17620 | -0,67 | 1,44E-24 | - | At1g17620/F11A6_23 [Source:UniProtKB/TrEMBL;Acc:Q9LNP3] |
| AT1G17665 | 0,92 | 4,65E-15 | - | unknown protein; FUNCTIONS IN: molecular_function unknown; INVOLVED IN: biological_process unknown; LOCATED IN: endomembrane system; EXPRESSED IN: 15 plant structures; EXPRESSED DURING: 11 growth stages; Ha. [Source:TAIR;Acc:AT1G17665] |
| AT1G17720 | -0,67 | 2,27E-55 | PP2AB2 | Serine/threonine-protein phosphatase 2A 55 kDa regulatory subunit B [Source:UniProtKB/TrEMBL;Acc:Q0WW19] |
| AT1G17870 | 0,63 | 3,31E-07 | EGY3 | Probable zinc metallopeptidase EGY3, chloroplastic [Source:UniProtKB/Swiss-Prot;Acc:Q9LMU1] |
| AT1G17880 | -0,67 | 1,22E-90 | BTF3 | Basic transcription factor 3 [Source:UniProtKB/Swiss-Prot;Acc:Q9SMW7] |
| AT1G17890 | -0,72 | 1,19E-80 | GER2 | Putative GDP-L-fucose synthase 2 [Source:UniProtKB/Swiss-Prot;Acc:Q9LMU0] |
| AT1G17960 | 1,93 | 4,86E-08 | - | Probable threonine--tRNA ligase, cytoplasmic [Source:UniProtKB/Swiss-Prot;Acc:Q8GZ45] |
| AT1G18060 | 1,13 | 1,61E-27 | - | AT1g18060/T10F20.23 [Source:UniProtKB/TrEMBL;Acc:Q9LM40] |
| AT1G18070 | -0,67 | 3,96E-90 | - | T10F20.8 protein [Source:UniProtKB/TrEMBL;Acc:Q9LMS7] |
| AT1G18075 | -0,86 | 3,74E-09 | MIR159B | MIR159/MIR159B; miRNA [Source:TAIR;Acc:AT1G18075] |
| AT1G18080 | -0,69 | 3,86E-78 | RACK1A | RACK1A_AT [Source:UniProtKB/TrEMBL;Acc:A0A178WHX3] |
| AT1G18140 | -1,84 | 2,38E-06 | LAC1 | Laccase-1 [Source:UniProtKB/Swiss-Prot;Acc:Q9LMS3] |
| AT1G18250 | -0,91 | 2,32E-31 | ATLP-1 | Pathogenesis-related thaumatin superfamily protein [Source:UniProtKB/TrEMBL;Acc:F4IAP1] |
| AT1G18290 | -1,11 | 6,65E-03 | - | DUF4228 domain protein [Source:UniProtKB/TrEMBL;Acc:Q9LE02] |
| AT1G18320 | 0,67 | 3,81E-14 | - | Mitochondrial import inner membrane translocase subunit Tim17/Tim22/Tim23 family protein [Source:TAIR;Acc:AT1G18320] |
| AT1G18370 | -0,81 | 3,57E-29 | KIN7A | Kinesin-like protein KIN-7A [Source:UniProtKB/Swiss-Prot;Acc:Q8S905] |
| AT1G18390 | 0,63 | 2,05E-07 | - | Protein kinase superfamily protein [Source:TAIR;Acc:AT1G18390] |
| AT1G18400 | 3,34 | 4,96E-02 | BEE1 | Transcription factor BEE 1 [Source:UniProtKB/Swiss-Prot;Acc:Q8GZ13] |
| AT1G18410 | 1,47 | 2,00E-13 | - | P-loop containing nucleoside triphosphate hydrolases superfamily protein [Source:TAIR;Acc:AT1G18410] |
| AT1G18450 | 0,77 | 9,43E-71 | ARP4 | Actin-related protein 4 [Source:UniProtKB/Swiss-Prot;Acc:Q84M92] |
| AT1G18485 | 0,74 | 6,95E-09 | PCMP-H8 | Pentatricopeptide repeat-containing protein At1g18485 [Source:UniProtKB/Swiss-Prot;Acc:Q0WN60] |
| AT1G18540 | -0,88 | 2,74E-93 | RPL6A | 60S ribosomal protein L6-1 [Source:UniProtKB/Swiss-Prot;Acc:Q9FZ76] |
| AT1G18550 | -0,75 | 4,26E-17 | KIN8A | Kinesin-like protein KIN-8A [Source:UniProtKB/Swiss-Prot;Acc:F4ICA0] |
| AT1G18570 | 1,75 | 9,47E-04 | MYB51 | Transcription factor MYB51 [Source:UniProtKB/Swiss-Prot;Acc:O49782] |
| AT1G18580 | -0,63 | 2,75E-27 | GAUT11 | Hexosyltransferase [Source:UniProtKB/TrEMBL;Acc:A0A178WM53] |
| AT1G18590 | -3,20 | 4,59E-34 | SOT17 | SOT17 [Source:UniProtKB/TrEMBL;Acc:A0A384KIF3] |
| AT1G18670 | 1,73 | 2,80E-63 | IBS1 | Protein kinase superfamily protein [Source:TAIR;Acc:AT1G18670] |
| AT1G18680 | 0,95 | 1,89E-23 | - | F6A14.21 protein [Source:UniProtKB/TrEMBL;Acc:Q9M9T9] |
| AT1G18745 | 0,58 | 4,69E-12 | - | - |
| AT1G18750 | 1,04 | 2,99E-06 | AGL65 | AGAMOUS-like 65 [Source:TAIR;Acc:AT1G18750] |
| AT1G18773 | 1,19 | 1,36E-04 | - | FUNCTIONS IN: molecular_function unknown; INVOLVED IN: biological_process unknown; LOCATED IN: endomembrane system; BEST Arabidopsis thaliana protein match is: carboxylesterases (TAIR:AT4G22300.1); Ha. [Source:TAIR;Acc:AT1G18773] |
| AT1G18860 | -1,46 | 3,18E-05 | WRKY61 | WRKY DNA-binding protein 61 [Source:TAIR;Acc:AT1G18860] |
| AT1G18880 | -1,52 | 4,65E-11 | NPF2.9 | Protein NRT1/ PTR FAMILY 2.9 [Source:UniProtKB/Swiss-Prot;Acc:Q9M9V7] |
| AT1G18900 | 0,77 | 4,64E-52 | - | Pentatricopeptide repeat (PPR) superfamily protein [Source:UniProtKB/TrEMBL;Acc:F4IDY2] |
| AT1G18940 | -2,66 | 1,54E-16 | - | F14D16.8 [Source:UniProtKB/TrEMBL;Acc:Q9LMD4] |
| AT1G18960 | 2,04 | 4,18E-02 | - | F14D16.11 [Source:UniProtKB/TrEMBL;Acc:Q9LMD1] |
| AT1G18980 | -2,49 | 2,90E-10 | - | At1g18980 [Source:UniProtKB/TrEMBL;Acc:Q29PR5] |
| AT1G19020 | 1,87 | 1,38E-09 | - | CDP-diacylglycerol-glycerol-3-phosphate 3-phosphatidyltransferase [Source:UniProtKB/TrEMBL;Acc:Q8VYY6] |
| AT1G19050 | -1,15 | 3,00E-03 | ARR7 | At1g19050 [Source:UniProtKB/TrEMBL;Acc:Q2HIJ6] |
| AT1G19160 | 2,78 | 3,02E-03 | - | Putative F-box protein At1g19160 [Source:UniProtKB/Swiss-Prot;Acc:Q9LMB0] |
| AT1G19220 | -1,68 | 2,75E-76 | ARF19 | Auxin response factor 19 [Source:UniProtKB/Swiss-Prot;Acc:Q8RYC8] |
| AT1G19230 | -0,85 | 1,09E-05 | RBOHE | Respiratory burst oxidase homolog protein E [Source:UniProtKB/Swiss-Prot;Acc:O81211] |
| AT1G19240 | 0,68 | 7,80E-28 | - | T29M8.11 [Source:UniProtKB/TrEMBL;Acc:Q9LMA2] |
| AT1G19290 | 0,67 | 4,48E-25 | - | Putative pentatricopeptide repeat-containing protein At1g19290 [Source:UniProtKB/Swiss-Prot;Acc:Q9LN69] |
| AT1G19300 | -0,89 | 1,82E-15 | GATL1 | Probable galacturonosyltransferase-like 1 [Source:UniProtKB/Swiss-Prot;Acc:Q9LN68] |
| AT1G19330 | -3,60 | 2,63E-03 | - | unknown protein; BEST Arabidopsis thaliana protein match is: unknown protein (TAIR:AT1G75060.1); Ha. [Source:TAIR;Acc:AT1G19330] |
| AT1G19360 | -0,81 | 4,97E-55 | RRA3 | Arabinosyltransferase RRA3 [Source:UniProtKB/Swiss-Prot;Acc:Q9LN62] |
| AT1G19376 | -1,83 | 8,06E-05 | - | snoRNA [Source:TAIR;Acc:AT1G19376] |
| AT1G19390 | -3,71 | 1,59E-02 | WAKL11 | Putative wall-associated receptor kinase-like 11 [Source:UniProtKB/Swiss-Prot;Acc:Q9LN59] |
| AT1G19440 | -0,58 | 6,75E-26 | KCS4 | 3-ketoacyl-CoA synthase 4 [Source:UniProtKB/Swiss-Prot;Acc:Q9LN49] |
| AT1G19464 | 5,61 | 1,11E-06 | MIR864A | MIR864a; miRNA [Source:TAIR;Acc:AT1G19464] |
| AT1G19470 | 5,98 | 8,72E-07 | - | Kelch repeat-containing protein At1g19470 [Source:UniProtKB/Swiss-Prot;Acc:P0C2F7] |
| AT1G19500 | 4,51 | 1,07E-02 | - | Putative uncharacterized protein [Source:UniProtKB/TrEMBL;Acc:Q5Q0G4] |
| AT1G19570 | -0,75 | 5,69E-04 | DHAR1 | Glutathione S-transferase DHAR1, mitochondrial [Source:UniProtKB/Swiss-Prot;Acc:Q9FWR4] |
| AT1G19600 | 1,20 | 2,56E-148 | - | At1g19600 [Source:UniProtKB/TrEMBL;Acc:Q9LN35] |
| AT1G19640 | 2,48 | 3,01E-11 | JMT | Jasmonate O-methyltransferase [Source:UniProtKB/Swiss-Prot;Acc:Q9AR07] |
| AT1G19680 | 0,99 | 4,75E-47 | - | At1g19680 [Source:UniProtKB/TrEMBL;Acc:Q9FXG6] |
| AT1G19690 | -0,71 | 6,42E-22 | - | At1g19690 [Source:UniProtKB/TrEMBL;Acc:Q147R9] |
| AT1G19700 | -0,66 | 2,86E-03 | BLH10 | BEL1-like homeodomain protein 10 [Source:UniProtKB/Swiss-Prot;Acc:Q9FXG8] |
| AT1G19770 | 1,56 | 3,71E-44 | PUP14 | PUP14 [Source:UniProtKB/TrEMBL;Acc:A0A178WHV5] |
| AT1G19840 | -1,63 | 1,20E-32 | - | F6F9.11 protein [Source:UniProtKB/TrEMBL;Acc:Q9FXI2] |
| AT1G19850 | 0,83 | 4,78E-71 | ARF5 | Auxin response factor [Source:UniProtKB/TrEMBL;Acc:A0A178W993] |
| AT1G19900 | -8,48 | 2,18E-15 | - | At1g19900/F6F9_4 [Source:UniProtKB/TrEMBL;Acc:Q93Z02] |
| AT1G19920 | -1,00 | 1,45E-105 | APS2 | ATP sulfurylase 2 [Source:UniProtKB/Swiss-Prot;Acc:Q43870] |
| AT1G19940 | -4,04 | 1,83E-30 | AtGH9B5 | Endoglucanase 2 [Source:UniProtKB/Swiss-Prot;Acc:Q9FXI9] |
| AT1G19968 | -0,63 | 9,25E-03 | - | other RNA [Source:TAIR;Acc:AT1G19968] |
| AT1G19970 | -0,72 | 2,70E-13 | - | ER lumen protein retaining receptor family protein [Source:UniProtKB/TrEMBL;Acc:F4HR00] |
| AT1G20010 | -1,46 | 1,43E-105 | TUBB5 | Tubulin beta-5 chain [Source:UniProtKB/Swiss-Prot;Acc:P29513] |
| AT1G20020 | -1,54 | 1,18E-26 | LFNR2 | Ferredoxin--NADP reductase, leaf isozyme 2, chloroplastic [Source:UniProtKB/Swiss-Prot;Acc:Q8W493] |
| AT1G20050 | -0,90 | 1,12E-127 | HYD1 | Probable 3-beta-hydroxysteroid-Delta(8),Delta(7)-isomerase [Source:UniProtKB/Swiss-Prot;Acc:O48962] |
| AT1G20080 | -1,59 | 3,28E-08 | SYT2 | Synaptotagmin-2 [Source:UniProtKB/Swiss-Prot;Acc:B6ETT4] |
| AT1G20130 | -0,95 | 4,23E-02 | APG | Anther-specific proline-rich protein APG [Source:UniProtKB/Swiss-Prot;Acc:P40602] |
| AT1G20140 | 0,74 | 5,71E-28 | ASK4 | SK4 [Source:UniProtKB/TrEMBL;Acc:A0A178W187] |
| AT1G20160 | 2,39 | 1,31E-26 | CRSP | CO(2)-response secreted protease [Source:UniProtKB/Swiss-Prot;Acc:Q9LNU1] |
| AT1G20190 | -2,02 | 4,52E-275 | EXPA11 | Expansin-A11 [Source:UniProtKB/Swiss-Prot;Acc:Q9LNU3] |
| AT1G20200 | -0,72 | 1,07E-119 | RPN3A | 26S proteasome non-ATPase regulatory subunit 3 homolog A [Source:UniProtKB/Swiss-Prot;Acc:Q9LNU4] |
| AT1G20340 | -1,01 | 1,76E-12 | DRT112 | Plastocyanin major isoform, chloroplastic [Source:UniProtKB/Swiss-Prot;Acc:P42699] |
| AT1G20350 | 4,90 | 2,18E-04 | TIM17-1 | Mitochondrial import inner membrane translocase subunit TIM17-1 [Source:UniProtKB/Swiss-Prot;Acc:Q9LN27] |
| AT1G20380 | 0,86 | 2,82E-35 | - | Prolyl oligopeptidase family protein [Source:UniProtKB/TrEMBL;Acc:F4HSS5] |
| AT1G20410 | -0,76 | 4,04E-38 | - | Pseudouridine synthase family protein [Source:UniProtKB/TrEMBL;Acc:F4HSS8] |
| AT1G20515 | 2,07 | 4,55E-04 | - | other RNA [Source:TAIR;Acc:AT1G20515] |
| AT1G20530 | 0,88 | 5,41E-05 | - | F5M15.15 [Source:UniProtKB/TrEMBL;Acc:Q9LMW0] |
| AT1G20620 | 0,59 | 1,15E-10 | CAT3 | catalase 3 [Source:TAIR;Acc:AT1G20620] |
| AT1G20630 | 1,44 | 4,91E-78 | CAT1 | CAT1 [Source:UniProtKB/TrEMBL;Acc:A0A384KXY6] |
| AT1G20650 | 1,09 | 3,77E-03 | PBL21 | ASG5 [Source:UniProtKB/TrEMBL;Acc:A0A178W8H8] |
| AT1G20720 | -0,88 | 2,03E-22 | - | RAD3-like DNA-binding helicase protein [Source:TAIR;Acc:AT1G20720] |
| AT1G20770 | -0,94 | 3,37E-75 | - | At1g20770 [Source:UniProtKB/TrEMBL;Acc:Q9LM77] |
| AT1G20790 | 2,77 | 5,96E-25 | - | Putative F-box/kelch-repeat protein At1g20790 [Source:UniProtKB/Swiss-Prot;Acc:Q9LM75] |
| AT1G20816 | 0,94 | 2,85E-16 | OEP21A | Outer envelope pore protein 21A, chloroplastic [Source:UniProtKB/Swiss-Prot;Acc:Q6ID99] |
| AT1G20840 | -1,10 | 1,26E-142 | MSSP1 | TMT1 [Source:UniProtKB/TrEMBL;Acc:A0A178W059] |
| AT1G20850 | -1,10 | 2,05E-10 | XCP2 | Cysteine protease XCP2 [Source:UniProtKB/Swiss-Prot;Acc:Q9LM66] |
| AT1G20870 | -0,80 | 3,33E-02 | IDM3 | Increased DNA methylation 3 [Source:UniProtKB/Swiss-Prot;Acc:Q9SYQ0] |
| AT1G20880 | 1,65 | 1,87E-19 | - | RNA-binding (RRM/RBD/RNP motifs) family protein [Source:UniProtKB/TrEMBL;Acc:F4HUQ5] |
| AT1G20920 | 0,77 | 7,52E-93 | RH42 | DEAD-box ATP-dependent RNA helicase 42 [Source:UniProtKB/Swiss-Prot;Acc:Q8H0U8] |
| AT1G20950 | -1,15 | 3,49E-145 | PFP-ALPHA1 | Pyrophosphate--fructose 6-phosphate 1-phosphotransferase subunit alpha [Source:UniProtKB/TrEMBL;Acc:A0A178WMV9] |
| AT1G21000 | 2,18 | 1,87E-45 | - | At1g21000/F9H16_1 [Source:UniProtKB/TrEMBL;Acc:Q93V70] |
| AT1G21050 | 1,15 | 1,70E-10 | - | Protein of unknown function, DUF617 [Source:TAIR;Acc:AT1G21050] |
| AT1G21070 | -1,02 | 3,74E-39 | URGT2 | UDP-rhamnose/UDP-galactose transporter 2 [Source:UniProtKB/Swiss-Prot;Acc:Q9LPU2] |
| AT1G21210 | 1,05 | 5,39E-04 | WAK4 | Wall-associated receptor kinase 4 [Source:UniProtKB/Swiss-Prot;Acc:Q9LMN6] |
| AT1G21270 | 6,52 | 2,66E-08 | WAK2 | Wall-associated receptor kinase 2 [Source:UniProtKB/Swiss-Prot;Acc:Q9LMP1] |
| AT1G21310 | -0,65 | 1,15E-38 | EXT3 | Extensin-3 [Source:UniProtKB/Swiss-Prot;Acc:Q9FS16] |
| AT1G21320 | -1,19 | 1,92E-02 | NSRB | Nuclear speckle RNA-binding protein B [Source:UniProtKB/Swiss-Prot;Acc:F4HWF9] |
| AT1G21350 | 0,90 | 1,62E-14 | - | Thioredoxin superfamily protein [Source:TAIR;Acc:AT1G21350] |
| AT1G21390 | 1,52 | 5,71E-34 | emb2170 | Emb2170 [Source:UniProtKB/TrEMBL;Acc:A0A178W1S8] |
| AT1G21410 | 1,64 | 1,11E-09 | SKP2A | F-box protein SKP2A [Source:UniProtKB/Swiss-Prot;Acc:Q9LPL4] |
| AT1G21460 | 1,30 | 3,01E-05 | SWEET1 | Bidirectional sugar transporter SWEET1 [Source:UniProtKB/Swiss-Prot;Acc:Q8L9J7] |
| AT1G21530 | 1,03 | 1,23E-03 | AEE10 | Probable acyl-activating enzyme 10 [Source:UniProtKB/Swiss-Prot;Acc:Q9LPK7] |
| AT1G21550 | 1,35 | 9,58E-08 | CML44 | Probable calcium-binding protein CML44 [Source:UniProtKB/Swiss-Prot;Acc:Q9LPK5] |
| AT1G21660 | -0,58 | 2,28E-18 | - | At1g21660 [Source:UniProtKB/TrEMBL;Acc:Q9XI12] |
| AT1G21670 | -0,95 | 1,00E-09 | - | DPP6 amino-terminal domain protein [Source:UniProtKB/TrEMBL;Acc:Q9XI11] |
| AT1G21680 | 1,84 | 4,89E-29 | - | DPP6 N-terminal domain-like protein [Source:UniProtKB/TrEMBL;Acc:Q9XI10] |
| AT1G21695 | -0,86 | 1,05E-03 | - | At1g21695 [Source:UniProtKB/TrEMBL;Acc:Q9XI08] |
| AT1G21750 | -1,61 | 1,37E-281 | PDIL1-1 | Protein disulfide isomerase-like 1-1 [Source:UniProtKB/Swiss-Prot;Acc:Q9XI01] |
| AT1G21760 | 1,65 | 1,52E-62 | SKIP32 | F-box protein 7 [Source:UniProtKB/Swiss-Prot;Acc:Q9XI00] |
| AT1G21900 | -0,64 | 2,02E-30 | - | Transmembrane emp24 domain-containing protein p24delta5 [Source:UniProtKB/Swiss-Prot;Acc:Q8RWM6] |
| AT1G21910 | 1,53 | 3,16E-75 | ERF012 | DREB26 [Source:UniProtKB/TrEMBL;Acc:A0A178W3Q9] |
| AT1G21940 | 9,12 | 7,15E-17 | - | unknown protein; FUNCTIONS IN: molecular_function unknown; INVOLVED IN: biological_process unknown; LOCATED IN: endomembrane system; BEST Arabidopsis thaliana protein match is: unknown protein (TAIR:AT1G21950.1); Ha. [Source:TAIR;Acc:AT1G21940] |
| AT1G22040 | 1,15 | 1,62E-52 | - | F-box/kelch-repeat protein At1g22040 [Source:UniProtKB/Swiss-Prot;Acc:Q9LM55] |
| AT1G22070 | 0,89 | 4,66E-22 | TGA3 | At1g22070 [Source:UniProtKB/TrEMBL;Acc:Q147Q9] |
| AT1G22160 | 1,97 | 2,87E-02 | FLZ5 | FCS-Like Zinc finger 5 [Source:UniProtKB/Swiss-Prot;Acc:Q8VY80] |
| AT1G22180 | 2,30 | 7,93E-105 | - | F16L1.9 protein [Source:UniProtKB/TrEMBL;Acc:Q9LM14] |
| AT1G22190 | 0,64 | 8,89E-28 | RAP2-13 | Ethylene-responsive transcription factor RAP2-13 [Source:UniProtKB/Swiss-Prot;Acc:Q9LM15] |
| AT1G22220 | -1,00 | 1,08E-04 | - | AUF2 [Source:UniProtKB/TrEMBL;Acc:A0A178W7K5] |
| AT1G22230 | -0,79 | 5,14E-22 | - | Nucleolar GTP-binding protein [Source:UniProtKB/TrEMBL;Acc:Q5XVK1] |
| AT1G22370 | 2,31 | 4,70E-11 | UGT85A5 | UDP-glycosyltransferase 85A5 [Source:UniProtKB/Swiss-Prot;Acc:Q9LMF0] |
| AT1G22380 | 4,59 | 3,40E-03 | UGT85A3 | Glycosyltransferase (Fragment) [Source:UniProtKB/TrEMBL;Acc:W8PW16] |
| AT1G22400 | 1,03 | 4,45E-73 | UGT85A1 | UDP-glycosyltransferase 85A1 [Source:UniProtKB/Swiss-Prot;Acc:Q9SK82] |
| AT1G22403 | 0,74 | 2,45E-12 | - | other RNA [Source:TAIR;Acc:AT1G22403] |
| AT1G22410 | -0,92 | 4,42E-121 | - | Phospho-2-dehydro-3-deoxyheptonate aldolase [Source:UniProtKB/TrEMBL;Acc:Q9SK84] |
| AT1G22430 | 2,05 | 8,76E-07 | - | Alcohol dehydrogenase-like 1 [Source:UniProtKB/Swiss-Prot;Acc:Q9SK86] |
| AT1G22450 | -0,75 | 6,29E-108 | COX6B-1 | COX6B [Source:UniProtKB/TrEMBL;Acc:A0A178WPQ4] |
| AT1G22470 | 0,61 | 2,09E-10 | - | At1g22470/F12K8_18 [Source:UniProtKB/TrEMBL;Acc:Q8L443] |
| AT1G22490 | 0,80 | 1,28E-04 | - | basic helix-loop-helix (bHLH) DNA-binding superfamily protein [Source:TAIR;Acc:AT1G22490] |
| AT1G22500 | -2,70 | 2,18E-14 | ATL15 | E3 ubiquitin-protein ligase ATL15 [Source:UniProtKB/Swiss-Prot;Acc:Q9SK92] |
| AT1G22520 | -1,07 | 5,46E-108 | - | Domain of unknown function (DUF543) [Source:TAIR;Acc:AT1G22520] |
| AT1G22530 | -0,97 | 4,51E-06 | PATL2 | Patellin-2 [Source:UniProtKB/Swiss-Prot;Acc:Q56ZI2] |
| AT1G22550 | 0,79 | 1,86E-20 | NPF5.16 | Protein NRT1/ PTR FAMILY 5.16 [Source:UniProtKB/Swiss-Prot;Acc:Q9SK96] |
| AT1G22570 | -1,29 | 1,10E-17 | NPF5.15 | Protein NRT1/ PTR FAMILY 5.15 [Source:UniProtKB/Swiss-Prot;Acc:Q9SK99] |
| AT1G22590 | -3,07 | 2,34E-09 | AGL87 | AGAMOUS-like 87 [Source:UniProtKB/TrEMBL;Acc:Q7X9H1] |
| AT1G22600 | -4,30 | 1,87E-03 | - | Late embryogenesis abundant protein (LEA) family protein [Source:UniProtKB/TrEMBL;Acc:Q9SKA2] |
| AT1G22640 | -0,66 | 3,78E-15 | MYB3 | MYB3 [Source:UniProtKB/TrEMBL;Acc:A0A178WFN1] |
| AT1G22650 | -1,56 | 1,20E-16 | INVD | Probable alkaline/neutral invertase D [Source:UniProtKB/Swiss-Prot;Acc:F4I2X9] |
| AT1G22740 | -0,91 | 1,28E-08 | RABG3B | Ras-related protein RABG3b [Source:UniProtKB/Swiss-Prot;Acc:O04157] |
| AT1G22770 | 0,72 | 6,71E-94 | GI | Protein GIGANTEA [Source:UniProtKB/Swiss-Prot;Acc:Q9SQI2] |
| AT1G22780 | -0,66 | 5,75E-68 | RPS18C | 40S ribosomal protein S18 [Source:UniProtKB/Swiss-Prot;Acc:P34788] |
| AT1G22830 | 0,82 | 2,19E-21 | PCMP-E24 | Pentatricopeptide repeat-containing protein At1g22830 [Source:UniProtKB/Swiss-Prot;Acc:Q4V389] |
| AT1G22880 | -0,66 | 4,17E-19 | CEL5 | Endoglucanase 3 [Source:UniProtKB/Swiss-Prot;Acc:Q2V4L8] |
| AT1G22885 | 3,84 | 4,87E-02 | - | unknown protein; FUNCTIONS IN: molecular_function unknown; INVOLVED IN: biological_process unknown; LOCATED IN: endomembrane system; EXPRESSED IN: 22 plant structures; EXPRESSED DURING: 13 growth stages; Ha. [Source:TAIR;Acc:AT1G22885] |
| AT1G22930 | 0,77 | 5,62E-49 | - | T-complex protein 11 [Source:UniProtKB/TrEMBL;Acc:O23129] |
| AT1G22985 | 0,93 | 3,63E-03 | ERF069 | CRF7 [Source:UniProtKB/TrEMBL;Acc:A0A178W874] |
| AT1G23000 | -0,75 | 1,90E-18 | - | Heavy metal transport/detoxification superfamily protein [Source:TAIR;Acc:AT1G23000] |
| AT1G23020 | 1,51 | 8,62E-80 | FRO3 | Ferric reduction oxidase 3, mitochondrial [Source:UniProtKB/Swiss-Prot;Acc:F4I4K7] |
| AT1G23080 | -0,86 | 4,37E-30 | PIN7 | Auxin efflux carrier component 7 [Source:UniProtKB/Swiss-Prot;Acc:Q940Y5] |
| AT1G23090 | 0,97 | 1,20E-04 | SULTR3;3 | Probable sulfate transporter 3.3 [Source:UniProtKB/Swiss-Prot;Acc:Q9SXS2] |
| AT1G23110 | -2,86 | 1,59E-06 | - | Fold protein [Source:UniProtKB/TrEMBL;Acc:Q6DSU0] |
| AT1G23150 | 1,27 | 9,54E-38 | - | Uncharacterized protein At1g23150 [Source:UniProtKB/TrEMBL;Acc:Q84JS3] |
| AT1G23160 | -0,79 | 1,50E-03 | - | Auxin-responsive GH3 family protein [Source:UniProtKB/TrEMBL;Acc:O49301] |
| AT1G23170 | -0,92 | 9,73E-82 | - | Protein of unknown function DUF2359, transmembrane [Source:TAIR;Acc:AT1G23170] |
| AT1G23205 | 1,22 | 1,17E-04 | - | At1g23205 [Source:UniProtKB/TrEMBL;Acc:O49297] |
| AT1G23450 | 0,88 | 4,52E-09 | - | Tetratricopeptide repeat (TPR)-like superfamily protein [Source:TAIR;Acc:AT1G23450] |
| AT1G23490 | -1,34 | 1,52E-143 | ARF2-A | ADP-ribosylation factor 2-B [Source:UniProtKB/Swiss-Prot;Acc:P0DH91] |
| AT1G23550 | 3,59 | 1,08E-101 | SRO2 | Probable inactive poly [ADP-ribose] polymerase SRO2 [Source:UniProtKB/Swiss-Prot;Acc:Q9ZUD9] |
| AT1G23560 | 6,62 | 6,09E-18 | - | F5O8.12 protein [Source:UniProtKB/TrEMBL;Acc:Q9ZUD8] |
| AT1G23710 | 1,73 | 4,19E-26 | - | At1g23710 [Source:UniProtKB/TrEMBL;Acc:Q9ZUC4] |
| AT1G23740 | 3,48 | 2,14E-39 | AOR | NADPH-dependent alkenal/one oxidoreductase, chloroplastic [Source:UniProtKB/Swiss-Prot;Acc:Q9ZUC1] |
| AT1G23750 | -1,59 | 8,08E-154 | - | F5O8.30 protein [Source:UniProtKB/TrEMBL;Acc:Q9ZUC0] |
| AT1G23760 | -2,77 | 9,35E-11 | PGL1 | Polygalacturonase 1 beta-like protein 1 [Source:UniProtKB/Swiss-Prot;Acc:P92982] |
| AT1G23790 | -0,80 | 3,53E-08 | - | At1g23790 [Source:UniProtKB/TrEMBL;Acc:Q9ZUB7] |
| AT1G23850 | 1,40 | 4,71E-29 | - | T23E23.1 [Source:UniProtKB/TrEMBL;Acc:Q9LRA9] |
| AT1G23860 | 0,99 | 2,72E-92 | RSZ21 | SRZ21 [Source:UniProtKB/TrEMBL;Acc:A0A178WC08] |
| AT1G23880 | 0,70 | 1,56E-11 | - | At1g23880/T23E23_8 [Source:UniProtKB/TrEMBL;Acc:Q8S9K1] |
| AT1G23900 | -0,82 | 2,85E-81 | GAMMA-ADR | AP-1 complex subunit gamma-1 [Source:UniProtKB/Swiss-Prot;Acc:Q84K16] |
| AT1G23950 | 1,02 | 2,14E-33 | - | UPF0725 protein At1g23950 [Source:UniProtKB/Swiss-Prot;Acc:Q9LR97] |
| AT1G23970 | 1,28 | 1,69E-68 | - | UPF0725 protein At1g23970 [Source:UniProtKB/Swiss-Prot;Acc:Q94A88] |
| AT1G24020 | -4,53 | 3,58E-03 | MLP423 | MLP-like protein 423 [Source:UniProtKB/Swiss-Prot;Acc:Q93VR4] |
| AT1G24050 | -0,61 | 5,23E-40 | - | At1g24050/T23E23_11 [Source:UniProtKB/TrEMBL;Acc:Q8L466] |
| AT1G24068 | 0,94 | 7,09E-05 | - | other RNA [Source:TAIR;Acc:AT1G24068] |
| AT1G24090 | 0,82 | 1,49E-34 | - | RNase H domain-containing protein [Source:UniProtKB/TrEMBL;Acc:F4I7R5] |
| AT1G24095 | 2,02 | 0,00E+00 | - | Putative thiol-disulfide oxidoreductase DCC [Source:UniProtKB/TrEMBL;Acc:Q9LR85] |
| AT1G24130 | -0,68 | 2,11E-04 | - | F3I6.5 protein [Source:UniProtKB/TrEMBL;Acc:O48679] |
| AT1G24150 | 1,73 | 2,27E-125 | ATFH4 | formin homologue 4 [Source:TAIR;Acc:AT1G24150] |
| AT1G24210 | 1,43 | 3,00E-07 | - | At1g24210 [Source:UniProtKB/TrEMBL;Acc:Q8LCT6] |
| AT1G24240 | -0,75 | 1,58E-30 | - | At1g24240/F3I6_17 [Source:UniProtKB/TrEMBL;Acc:O48691] |
| AT1G24280 | -0,96 | 1,46E-66 | G6PD3 | Glucose-6-phosphate 1-dehydrogenase [Source:UniProtKB/TrEMBL;Acc:A0A178WP00] |
| AT1G24320 | -2,34 | 9,02E-46 | GCS2 | Alpha-glucosidase 2 [Source:UniProtKB/Swiss-Prot;Acc:Q84M89] |
| AT1G24330 | 0,74 | 9,26E-05 | PUB6 | U-box domain-containing protein 6 [Source:UniProtKB/Swiss-Prot;Acc:O48700] |
| AT1G24340 | 0,92 | 2,23E-55 | EMB260 | FAD/NAD(P)-binding oxidoreductase family protein [Source:UniProtKB/TrEMBL;Acc:Q8GYJ7] |
| AT1G24360 | -0,74 | 1,89E-68 | - | 3-oxoacyl-[acyl-carrier-protein] reductase, chloroplastic [Source:UniProtKB/Swiss-Prot;Acc:P33207] |
| AT1G24510 | -0,80 | 5,73E-104 | CCT5 | T-complex protein 1 subunit epsilon [Source:UniProtKB/Swiss-Prot;Acc:O04450] |
| AT1G24600 | 0,80 | 8,15E-07 | - | At1g24600 [Source:UniProtKB/TrEMBL;Acc:Q9FYK4] |
| AT1G24620 | -5,51 | 2,26E-11 | CML25 | Probable calcium-binding protein CML25 [Source:UniProtKB/Swiss-Prot;Acc:Q9FYK2] |
| AT1G24735 | -2,33 | 1,47E-17 | - | S-adenosyl-L-methionine-dependent methyltransferases superfamily protein [Source:UniProtKB/TrEMBL;Acc:F4IAT4] |
| AT1G24996 | 1,00 | 1,08E-02 | - | F5A9.10 [Source:UniProtKB/TrEMBL;Acc:Q9FXK6] |
| AT1G25054 | -2,40 | 2,97E-05 | LPXC5 | Probable UDP-3-O-acyl-N-acetylglucosamine deacetylase 3, mitochondrial [Source:UniProtKB/Swiss-Prot;Acc:P0DKB8] |
| AT1G25230 | -1,74 | 5,34E-34 | - | Calcineurin-like metallo-phosphoesterase superfamily protein [Source:TAIR;Acc:AT1G25230] |
| AT1G25250 | -1,97 | 9,65E-21 | AtIDD16 | IDD16 [Source:UniProtKB/TrEMBL;Acc:A0A178WG74] |
| AT1G25275 | 0,83 | 6,80E-50 | - | AT1G25275 protein [Source:UniProtKB/TrEMBL;Acc:B9DG55] |
| AT1G25277 | 4,42 | 9,57E-06 | - | - |
| AT1G25450 | -0,63 | 2,06E-14 | KCS5 | 3-ketoacyl-CoA synthase 5 [Source:UniProtKB/Swiss-Prot;Acc:Q9C6L5] |
| AT1G25480 | -0,66 | 1,57E-27 | ALMT4 | Aluminum-activated malate transporter 4 [Source:UniProtKB/Swiss-Prot;Acc:Q9C6L8] |
| AT1G25490 | -0,71 | 5,92E-65 | PP2AA1 | Serine/threonine-protein phosphatase 2A 65 kDa regulatory subunit A alpha isoform [Source:UniProtKB/Swiss-Prot;Acc:Q38845] |
| AT1G25560 | 0,74 | 8,60E-19 | TEM1 | AP2/ERF and B3 domain-containing transcription repressor TEM1 [Source:UniProtKB/Swiss-Prot;Acc:Q9C6M5] |
| AT1G25570 | -0,59 | 1,02E-43 | - | Di-glucose binding protein with Leucine-rich repeat domain-containing protein [Source:UniProtKB/TrEMBL;Acc:F4ICJ5] |
| AT1G26100 | -1,03 | 1,30E-23 | CYB561D | Probable transmembrane ascorbate ferrireductase 4 [Source:UniProtKB/Swiss-Prot;Acc:Q9C540] |
| AT1G26150 | 0,72 | 1,39E-23 | PERK10 | Proline-rich receptor-like protein kinase PERK10 [Source:UniProtKB/Swiss-Prot;Acc:Q9C660] |
| AT1G26190 | -0,91 | 1,70E-59 | TTM2 | Inorganic pyrophosphatase TTM2 [Source:UniProtKB/Swiss-Prot;Acc:Q9C664] |
| AT1G26230 | -1,32 | 2,38E-20 | CPN60B4 | Cpn60beta4 [Source:UniProtKB/TrEMBL;Acc:A0A178WH16] |
| AT1G26233 | -0,64 | 3,04E-02 | SNOR95 | SNOR95; snoRNA [Source:TAIR;Acc:AT1G26233] |
| AT1G26440 | 1,08 | 1,10E-36 | UPS5 | Ureide permease 5 [Source:UniProtKB/Swiss-Prot;Acc:Q93Z75] |
| AT1G26500 | 0,58 | 2,71E-02 | - | Putative pentatricopeptide repeat-containing protein At1g26500 [Source:UniProtKB/Swiss-Prot;Acc:Q9FZD4] |
| AT1G26515 | 2,53 | 1,12E-05 | - | Putative F-box protein At1g26515 [Source:UniProtKB/Swiss-Prot;Acc:Q9FZD6] |
| AT1G26590 | -2,23 | 8,53E-29 | - | C2H2-like zinc finger protein [Source:TAIR;Acc:AT1G26590] |
| AT1G26630 | -0,60 | 7,56E-64 | ELF5A-2 | Eukaryotic translation initiation factor 5A-2 [Source:UniProtKB/Swiss-Prot;Acc:Q93VP3] |
| AT1G26665 | 1,73 | 1,50E-180 | - | Mediator complex, subunit Med10 [Source:TAIR;Acc:AT1G26665] |
| AT1G26680 | 2,40 | 1,67E-175 | - | transcriptional factor B3 family protein [Source:TAIR;Acc:AT1G26680] |
| AT1G26700 | -2,57 | 8,58E-78 | MLO14 | MLO-like protein 14 [Source:UniProtKB/Swiss-Prot;Acc:Q94KB1] |
| AT1G26720 | 1,21 | 7,74E-08 | - | T24P13.10 [Source:UniProtKB/TrEMBL;Acc:Q9LQY0] |
| AT1G26730 | 1,66 | 9,65E-29 | PHO1-H7 | Phosphate transporter PHO1 homolog 7 [Source:UniProtKB/Swiss-Prot;Acc:Q6R8G3] |
| AT1G26761 | -4,71 | 2,66E-03 | - | Arabinanase/levansucrase/invertase [Source:UniProtKB/TrEMBL;Acc:Q93ZE3] |
| AT1G26762 | 4,25 | 2,65E-13 | - | unknown protein; FUNCTIONS IN: molecular_function unknown; INVOLVED IN: biological_process unknown; LOCATED IN: endomembrane system; Ha. [Source:TAIR;Acc:AT1G26762] |
| AT1G26770 | -1,26 | 5,23E-50 | ATEXPA10 | Expansin [Source:UniProtKB/TrEMBL;Acc:F4HPC1] |
| AT1G26790 | -6,10 | 1,77E-07 | - | Dof-type zinc finger DNA-binding family protein [Source:TAIR;Acc:AT1G26790] |
| AT1G26800 | 6,30 | 8,14E-09 | MPSR1 | E3 ubiquitin-protein ligase MPSR1 [Source:UniProtKB/Swiss-Prot;Acc:Q9LQX2] |
| AT1G26840 | -0,91 | 3,09E-29 | ORC6 | Origin of replication complex subunit 6 [Source:UniProtKB/Swiss-Prot;Acc:Q9ZVH3] |
| AT1G26850 | -1,07 | 7,74E-139 | - | Probable methyltransferase PMT2 [Source:UniProtKB/Swiss-Prot;Acc:B9DFI7] |
| AT1G26880 | -0,93 | 2,72E-120 | RPL34A | 60S ribosomal protein L34-1 [Source:UniProtKB/Swiss-Prot;Acc:Q42351] |
| AT1G26940 | -0,66 | 5,86E-20 | CYP23 | Peptidyl-prolyl cis-trans isomerase CYP23 [Source:UniProtKB/Swiss-Prot;Acc:Q8LDR3] |
| AT1G26960 | -0,97 | 3,66E-33 | ATHB-23 | Homeobox-leucine zipper protein ATHB-23 [Source:UniProtKB/Swiss-Prot;Acc:Q8LFD3] |
| AT1G27000 | 0,65 | 1,79E-59 | - | At1g27000/T7N9_6 [Source:UniProtKB/TrEMBL;Acc:Q9C5M5] |
| AT1G27020 | 3,88 | 1,70E-17 | - | Plant/protein [Source:UniProtKB/TrEMBL;Acc:O04551] |
| AT1G27120 | -0,60 | 5,43E-09 | GALT4 | Hydroxyproline O-galactosyltransferase GALT4 [Source:UniProtKB/Swiss-Prot;Acc:Q8GXG6] |
| AT1G27130 | -1,17 | 3,87E-127 | GSTU13 | Glutathione S-transferase U13 [Source:UniProtKB/Swiss-Prot;Acc:Q9FUS6] |
| AT1G27140 | -6,20 | 1,01E-131 | GSTU14 | GSTU14 [Source:UniProtKB/TrEMBL;Acc:A0A178WN03] |
| AT1G27170 | 1,29 | 1,92E-38 | - | Transmembrane receptors / ATP binding protein [Source:UniProtKB/TrEMBL;Acc:F4HR53] |
| AT1G27200 | 0,60 | 7,63E-07 | - | DUF23/GT0 (Fragment) [Source:UniProtKB/TrEMBL;Acc:I1VCA1] |
| AT1G27210 | -0,78 | 1,46E-44 | TOR1L4 | TORTIFOLIA1-like protein 4 [Source:UniProtKB/Swiss-Prot;Acc:Q93ZH1] |
| AT1G27330 | -0,75 | 1,81E-32 | - | At1g27330 [Source:UniProtKB/TrEMBL;Acc:Q84K46] |
| AT1G27350 | -0,75 | 5,00E-53 | - | At1g27330 [Source:UniProtKB/TrEMBL;Acc:Q84K46] |
| AT1G27380 | 1,59 | 7,66E-03 | RIC2 | RIC2 [Source:UniProtKB/TrEMBL;Acc:A0A178WAB0] |
| AT1G27385 | 0,66 | 5,80E-18 | - | unknown protein; FUNCTIONS IN: molecular_function unknown; INVOLVED IN: biological_process unknown; LOCATED IN: chloroplast; EXPRESSED IN: 24 plant structures; EXPRESSED DURING: 13 growth stages; CONTAINS InterPro DOMAIN/s: Protein of unknown functi /.../493 (InterPro:IPR007454); Ha. [Source:TAIR;Acc:AT1G27385] |
| AT1G27400 | -0,60 | 1,55E-77 | RPL17A | 60S ribosomal protein L17-1 [Source:UniProtKB/Swiss-Prot;Acc:Q93VI3] |
| AT1G27410 | 1,46 | 3,35E-56 | - | DNA repair metallo-beta-lactamase family protein [Source:UniProtKB/TrEMBL;Acc:Q9FZJ4] |
| AT1G27420 | 1,53 | 7,68E-47 | - | Putative F-box/kelch-repeat protein At1g27420 [Source:UniProtKB/Swiss-Prot;Acc:Q9FZJ3] |
| AT1G27580 | 4,40 | 5,15E-03 | - | Protein of unknown function (DUF295) [Source:TAIR;Acc:AT1G27580] |
| AT1G27600 | -0,62 | 2,26E-37 | IRX9H | Probable beta-1,4-xylosyltransferase IRX9H [Source:UniProtKB/Swiss-Prot;Acc:Q9SXC4] |
| AT1G27620 | -0,83 | 1,94E-03 | - | At1g27620 [Source:UniProtKB/TrEMBL;Acc:Q9S808] |
| AT1G27660 | -1,56 | 4,02E-29 | BHLH110 | Transcription factor bHLH110 [Source:UniProtKB/Swiss-Prot;Acc:Q9SFZ3] |
| AT1G27680 | -0,63 | 4,03E-23 | APL2 | Glucose-1-phosphate adenylyltransferase large subunit 2, chloroplastic [Source:UniProtKB/Swiss-Prot;Acc:P55230] |
| AT1G27700 | -0,66 | 9,36E-19 | - | At1g27700/T22C5_14 [Source:UniProtKB/TrEMBL;Acc:Q940U5] |
| AT1G27720 | 1,00 | 1,38E-16 | TAF4 | Transcription initiation factor TFIID subunit 4 [Source:UniProtKB/Swiss-Prot;Acc:Q6SJR1] |
| AT1G27730 | 3,70 | 2,03E-89 | ZAT10 | Zinc finger protein ZAT10 [Source:UniProtKB/Swiss-Prot;Acc:Q96289] |
| AT1G27740 | -1,91 | 9,45E-17 | BHLH54 | Transcription factor bHLH54 [Source:UniProtKB/Swiss-Prot;Acc:Q8LEG1] |
| AT1G27880 | -0,68 | 3,71E-14 | RECQL5 | ATP-dependent DNA helicase Q-like 5 [Source:UniProtKB/Swiss-Prot;Acc:Q0WVW7] |
| AT1G27930 | -0,83 | 1,70E-45 | - | Probable methyltransferase At1g27930 [Source:UniProtKB/Swiss-Prot;Acc:Q9C7F9] |
| AT1G27950 | -0,80 | 1,53E-34 | LTPG1 | Non-specific lipid transfer protein GPI-anchored 1 [Source:UniProtKB/Swiss-Prot;Acc:Q9C7F7] |
| AT1G27970 | -0,74 | 2,40E-73 | NTF2B | Nuclear transport factor 2B [Source:UniProtKB/TrEMBL;Acc:A8MS55] |
| AT1G28050 | 0,71 | 7,90E-19 | COL15 | Zinc finger protein CONSTANS-LIKE 15 [Source:UniProtKB/Swiss-Prot;Acc:Q9C7E8] |
| AT1G28110 | 0,90 | 5,46E-39 | SCPL45 | Serine carboxypeptidase-like 45 [Source:UniProtKB/Swiss-Prot;Acc:Q93Y09] |
| AT1G28160 | -3,65 | 1,35E-03 | ERF087 | Ethylene-responsive transcription factor ERF087 [Source:UniProtKB/Swiss-Prot;Acc:Q9FZ90] |
| AT1G28190 | 1,48 | 3,78E-17 | - | F3H9.15 protein [Source:UniProtKB/TrEMBL;Acc:Q9FZ93] |
| AT1G28210 | 1,38 | 2,06E-50 | ATJ1 | DNAJ heat shock family protein [Source:UniProtKB/TrEMBL;Acc:F4HWI4] |
| AT1G28260 | 0,89 | 9,29E-05 | SMG7L | Protein SMG7L [Source:UniProtKB/Swiss-Prot;Acc:Q9FZ99] |
| AT1G28330 | 0,70 | 1,21E-02 | DRM1 | Dormancy-associated protein 1 [Source:UniProtKB/Swiss-Prot;Acc:B9DGG8] |
| AT1G28350 | -0,59 | 3,68E-47 | - | Tyrosine--tRNA ligase 2, cytoplasmic [Source:UniProtKB/Swiss-Prot;Acc:F4HWL4] |
| AT1G28380 | 0,64 | 1,04E-05 | NSL1 | NSL1 [Source:UniProtKB/TrEMBL;Acc:A0A178WFB2] |
| AT1G28390 | -0,79 | 3,32E-33 | - | Protein kinase superfamily protein [Source:UniProtKB/TrEMBL;Acc:F4HWL9] |
| AT1G28400 | -1,54 | 5,87E-55 | - | F3M18.16 [Source:UniProtKB/TrEMBL;Acc:Q9SGN8] |
| AT1G28470 | 0,65 | 4,02E-04 | NAC010 | NAC domain-containing protein 10 [Source:UniProtKB/Swiss-Prot;Acc:F4HY61] |
| AT1G28480 | 1,01 | 2,24E-10 | GRXC9 | Glutaredoxin-C9 [Source:UniProtKB/Swiss-Prot;Acc:Q9SGP6] |
| AT1G28510 | -0,94 | 1,76E-100 | - | At1g28510/F3M18_5 [Source:UniProtKB/TrEMBL;Acc:Q9SGP9] |
| AT1G28530 | 0,59 | 3,91E-20 | - | Deneddylase [Source:UniProtKB/TrEMBL;Acc:Q84TH8] |
| AT1G28600 | 4,38 | 7,64E-65 | - | GDSL esterase/lipase At1g28600 [Source:UniProtKB/Swiss-Prot;Acc:Q94F40] |
| AT1G28610 | 1,99 | 2,44E-03 | - | GDSL esterase/lipase At1g28610 [Source:UniProtKB/Swiss-Prot;Acc:Q9SHP6] |
| AT1G28660 | 1,03 | 1,16E-10 | - | GDSL esterase/lipase At1g28660 [Source:UniProtKB/Swiss-Prot;Acc:Q9FPE4] |
| AT1G28680 | -1,43 | 6,82E-40 | - | Anthranilate N-hydroxycinnamoyl/benzoyltransferase, putative [Source:UniProtKB/TrEMBL;Acc:Q8LF28] |
| AT1G28690 | 0,92 | 2,98E-07 | PCMP-E34 | Pentatricopeptide repeat-containing protein At1g28690, mitochondrial [Source:UniProtKB/Swiss-Prot;Acc:Q1PFQ9] |
| AT1G28760 | 0,87 | 1,47E-18 | - | F1K23.6 [Source:UniProtKB/TrEMBL;Acc:Q9SHQ6] |
| AT1G28765 | 2,20 | 6,59E-24 | - | - |
| AT1G28850 | 2,01 | 2,68E-02 | - | pre-tRNA [Source:TAIR;Acc:AT1G28850] |
| AT1G28930 | 2,21 | 2,20E-05 | - | pre-tRNA [Source:TAIR;Acc:AT1G28930] |
| AT1G28940 | 1,91 | 3,83E-03 | - | pre-tRNA [Source:TAIR;Acc:AT1G28940] |
| AT1G28950 | 2,05 | 2,67E-03 | - | pre-tRNA [Source:TAIR;Acc:AT1G28950] |
| AT1G28970 | 4,07 | 1,43E-04 | - | pre-tRNA [Source:TAIR;Acc:AT1G28970] |
| AT1G28980 | 4,47 | 1,30E-03 | - | pre-tRNA [Source:TAIR;Acc:AT1G28980] |
| AT1G28990 | 2,34 | 2,95E-03 | - | pre-tRNA [Source:TAIR;Acc:AT1G28990] |
| AT1G29020 | -2,17 | 5,33E-09 | - | Calcium-binding EF-hand family protein [Source:TAIR;Acc:AT1G29020] |
| AT1G29030 | 0,90 | 1,74E-73 | - | Apoptosis inhibitory protein 5 (API5) [Source:UniProtKB/TrEMBL;Acc:Q8GXH2] |
| AT1G29040 | -0,64 | 1,03E-34 | - | 50S ribosomal protein L34 [Source:UniProtKB/TrEMBL;Acc:Q8LDL9] |
| AT1G29060 | -0,99 | 1,05E-19 | - | Bet1-like protein At1g29060 [Source:UniProtKB/Swiss-Prot;Acc:Q8L9S0] |
| AT1G29179 | 1,32 | 2,37E-04 | - | BEST Arabidopsis thaliana protein match is: Cysteine/Histidine-rich C1 domain family protein (TAIR:AT1G44030.1); Ha. [Source:TAIR;Acc:AT1G29179] |
| AT1G29180 | 1,33 | 2,93E-03 | - | Cysteine/Histidine-rich C1 domain family protein [Source:UniProtKB/TrEMBL;Acc:F4HZX5] |
| AT1G29240 | 0,66 | 1,24E-02 | - | F28N24.8 protein [Source:UniProtKB/TrEMBL;Acc:Q9LP52] |
| AT1G29270 | -2,06 | 2,07E-15 | - | unknown protein; BEST Arabidopsis thaliana protein match is: unknown protein (TAIR:AT2G40435.1); Ha. [Source:TAIR;Acc:AT1G29270] |
| AT1G29290 | 1,23 | 7,78E-10 | CEP14 | Precursor of CEP14 [Source:UniProtKB/Swiss-Prot;Acc:Q52K95] |
| AT1G29310 | -1,34 | 3,07E-217 | - | SecY protein transport family protein [Source:UniProtKB/TrEMBL;Acc:Q8RWJ5] |
| AT1G29390 | -1,16 | 3,61E-32 | COR413IM2 | COR413IM2 [Source:UniProtKB/TrEMBL;Acc:A0A178WEF2] |
| AT1G29395 | -1,37 | 3,11E-04 | COR413IM1 | Cold-regulated 413 inner membrane protein 1, chloroplastic [Source:UniProtKB/Swiss-Prot;Acc:Q94AL8] |
| AT1G29460 | -3,94 | 3,41E-02 | - | SAUR-like auxin-responsive protein family [Source:TAIR;Acc:AT1G29460] |
| AT1G29640 | 1,30 | 6,86E-30 | - | At1g29640 [Source:UniProtKB/TrEMBL;Acc:Q9C7N7] |
| AT1G29660 | -4,89 | 8,61E-04 | - | GDSL esterase/lipase At1g29660 [Source:UniProtKB/Swiss-Prot;Acc:Q9C7N5] |
| AT1G29730 | -1,31 | 7,61E-40 | - | Leucine-rich repeat transmembrane protein kinase [Source:TAIR;Acc:AT1G29730] |
| AT1G29740 | -0,86 | 6,15E-07 | - | Leucine-rich repeat transmembrane protein kinase [Source:UniProtKB/TrEMBL;Acc:F4I337] |
| AT1G29770 | 0,81 | 2,48E-08 | - | At1g29770 [Source:UniProtKB/TrEMBL;Acc:Q9FXF4] |
| AT1G29785 | -0,95 | 5,93E-41 | - | other RNA [Source:TAIR;Acc:AT1G29785] |
| AT1G29790 | -0,85 | 1,09E-66 | - | At1g29790 [Source:UniProtKB/TrEMBL;Acc:Q8RWB7] |
| AT1G29820 | -0,62 | 4,47E-17 | - | Magnesium transporter CorA-like family protein [Source:UniProtKB/TrEMBL;Acc:F4I348] |
| AT1G29830 | -0,82 | 2,30E-02 | - | Magnesium transporter CorA-like family protein [Source:UniProtKB/TrEMBL;Acc:F4I350] |
| AT1G29890 | -0,93 | 2,44E-67 | - | O-acetyltransferase family protein [Source:TAIR;Acc:AT1G29890] |
| AT1G29910 | -0,78 | 1,51E-02 | LHCB1.1 | Chlorophyll a-b binding protein 3, chloroplastic [Source:UniProtKB/Swiss-Prot;Acc:Q8VZ87] |
| AT1G29920 | -3,78 | 4,58E-03 | LHCB1.1 | Chlorophyll a-b binding protein 3, chloroplastic [Source:UniProtKB/Swiss-Prot;Acc:Q8VZ87] |
| AT1G29930 | -2,90 | 5,69E-27 | LHCB1.3 | Chlorophyll a-b binding protein 1, chloroplastic [Source:UniProtKB/Swiss-Prot;Acc:P04778] |
| AT1G30040 | 6,06 | 3,58E-79 | GA2OX2 | Gibberellin 2-beta-dioxygenase 2 [Source:UniProtKB/Swiss-Prot;Acc:Q9XFR9] |
| AT1G30070 | 0,94 | 7,65E-60 | - | SGS domain-containing protein [Source:UniProtKB/TrEMBL;Acc:F4I4Q9] |
| AT1G30090 | 1,22 | 7,70E-51 | - | F-box/kelch-repeat protein At1g30090 [Source:UniProtKB/Swiss-Prot;Acc:Q9C6Z0] |
| AT1G30120 | -0,77 | 2,10E-73 | PDH-E1 BETA | Pyruvate dehydrogenase E1 component subunit beta-2, chloroplastic [Source:UniProtKB/Swiss-Prot;Acc:Q9C6Z3] |
| AT1G30220 | 4,48 | 3,96E-03 | INT2 | Probable inositol transporter 2 [Source:UniProtKB/Swiss-Prot;Acc:Q9C757] |
| AT1G30230 | 1,13 | 6,77E-259 | - | Translation elongation factor EF1B/ribosomal protein S6 family protein [Source:UniProtKB/TrEMBL;Acc:A8MRC4] |
| AT1G30380 | -1,47 | 2,11E-11 | PSAK | Photosystem I reaction center subunit psaK, chloroplastic [Source:UniProtKB/Swiss-Prot;Acc:Q9SUI5] |
| AT1G30450 | -0,71 | 3,39E-75 | CCC1 | Cation-chloride cotransporter 1 [Source:UniProtKB/Swiss-Prot;Acc:Q2UVJ5] |
| AT1G30475 | 0,78 | 1,64E-13 | - | AT1G30475 protein [Source:UniProtKB/TrEMBL;Acc:A8MQH6] |
| AT1G30500 | 3,87 | 2,56E-16 | NFYA7 | Nuclear transcription factor Y subunit A-7 [Source:UniProtKB/Swiss-Prot;Acc:Q84JP1] |
| AT1G30510 | -1,06 | 7,82E-105 | RFNR2 | Ferredoxin--NADP reductase, root isozyme 2, chloroplastic [Source:UniProtKB/Swiss-Prot;Acc:Q9S9P8] |
| AT1G30520 | -0,68 | 3,81E-06 | AAE14 | 2-succinylbenzoate--CoA ligase, chloroplastic/peroxisomal [Source:UniProtKB/Swiss-Prot;Acc:Q8VYJ1] |
| AT1G30530 | 0,70 | 7,90E-06 | UGT78D1 | Glycosyltransferase (Fragment) [Source:UniProtKB/TrEMBL;Acc:W8PVA4] |
| AT1G30550 | -0,72 | 4,70E-21 | - | S-adenosyl-L-methionine-dependent methyltransferases superfamily protein [Source:TAIR;Acc:AT1G30550] |
| AT1G30630 | -0,95 | 1,43E-132 | - | Coatomer subunit epsilon-1 [Source:UniProtKB/Swiss-Prot;Acc:Q9SA78] |
| AT1G30660 | 2,87 | 3,48E-10 | - | Primase homolog protein [Source:UniProtKB/Swiss-Prot;Acc:F4I6E6] |
| AT1G30700 | -0,59 | 1,85E-05 | - | Berberine bridge enzyme-like 8 [Source:UniProtKB/Swiss-Prot;Acc:Q9SA85] |
| AT1G30720 | -0,61 | 1,15E-03 | - | Berberine bridge enzyme-like 10 [Source:UniProtKB/Swiss-Prot;Acc:Q9SA87] |
| AT1G30730 | -1,45 | 2,82E-21 | - | Berberine bridge enzyme-like 11 [Source:UniProtKB/Swiss-Prot;Acc:Q9SA88] |
| AT1G30755 | 0,89 | 7,57E-14 | - | Elongation factor G, putative (DUF668) [Source:UniProtKB/TrEMBL;Acc:Q8L5Y3] |
| AT1G30840 | 4,10 | 3,89E-03 | PUP4 | Probable purine permease 4 [Source:UniProtKB/Swiss-Prot;Acc:Q9SY29] |
| AT1G30845 | -0,72 | 8,41E-10 | - | Cell growth defect factor-2 [Source:UniProtKB/TrEMBL;Acc:Q3C1C7] |
| AT1G30850 | -5,88 | 3,46E-06 | RSH4 | Root hair specific 4 [Source:UniProtKB/TrEMBL;Acc:Q9SY31] |
| AT1G30860 | -1,47 | 2,66E-07 | - | At1g30860 [Source:UniProtKB/TrEMBL;Acc:Q6NQ80] |
| AT1G30870 | -7,16 | 5,43E-20 | PER7 | Peroxidase 7 [Source:UniProtKB/Swiss-Prot;Acc:Q9SY33] |
| AT1G30880 | 0,89 | 3,36E-117 | - | At1g30880 [Source:UniProtKB/TrEMBL;Acc:Q9FYH5] |
| AT1G30890 | -0,87 | 2,56E-51 | - | Integral membrane HRF1 family protein [Source:UniProtKB/TrEMBL;Acc:Q94BQ9] |
| AT1G30900 | -2,08 | 1,08E-67 | VSR6 | Vacuolar-sorting receptor 6 [Source:UniProtKB/Swiss-Prot;Acc:Q9FYH7] |
| AT1G30910 | 0,64 | 4,41E-21 | - | F17F8.22 [Source:UniProtKB/TrEMBL;Acc:Q9FYH8] |
| AT1G30990 | -6,46 | 7,19E-08 | - | F17F8.9 [Source:UniProtKB/TrEMBL;Acc:Q9FYJ0] |
| AT1G31070 | -0,62 | 2,70E-40 | GLCNAC1PUT1 | GlcNAc1pUT1 [Source:UniProtKB/TrEMBL;Acc:A0A178W850] |
| AT1G31120 | 0,86 | 9,34E-21 | POT10 | Potassium transporter 10 [Source:UniProtKB/Swiss-Prot;Acc:Q9SA05] |
| AT1G31170 | 0,76 | 1,54E-08 | ATSRX | Sulfiredoxin [Source:UniProtKB/TrEMBL;Acc:F4I7W2] |
| AT1G31230 | -0,65 | 8,91E-46 | AKHSDH1 | Bifunctional aspartokinase/homoserine dehydrogenase 1, chloroplastic [Source:UniProtKB/Swiss-Prot;Acc:Q9SA18] |
| AT1G31240 | -0,94 | 5,33E-04 | - | At1g31240 [Source:UniProtKB/TrEMBL;Acc:Q9SA19] |
| AT1G31290 | 4,69 | 4,54E-04 | AGO3 | Protein argonaute 3 [Source:UniProtKB/Swiss-Prot;Acc:Q9SHF2] |
| AT1G31320 | 1,34 | 1,46E-04 | LBD4 | LOB domain-containing protein 4 [Source:UniProtKB/Swiss-Prot;Acc:Q9SHE9] |
| AT1G31330 | -2,09 | 2,58E-22 | PSAF | Photosystem I reaction center subunit III, chloroplastic [Source:UniProtKB/Swiss-Prot;Acc:Q9SHE8] |
| AT1G31360 | 0,63 | 1,46E-33 | RECQL2 | RECQ helicase L2 [Source:TAIR;Acc:AT1G31360] |
| AT1G31370 | 2,81 | 1,07E-02 | - | Ubiquitin-specific protease family C19-related protein [Source:UniProtKB/TrEMBL;Acc:Q9C872] |
| AT1G31540 | 1,29 | 2,19E-45 | - | Disease resistance protein (TIR-NBS-LRR class) family [Source:UniProtKB/TrEMBL;Acc:F4I9F1] |
| AT1G31730 | -0,62 | 1,11E-58 | - | AP-4 complex subunit epsilon [Source:UniProtKB/Swiss-Prot;Acc:Q8L7A9] |
| AT1G31760 | 1,01 | 5,53E-22 | - | At1g31760 [Source:UniProtKB/TrEMBL;Acc:Q9C504] |
| AT1G31772 | 0,98 | 1,28E-07 | - | Defensin-like (DEFL) family protein [Source:TAIR;Acc:AT1G31772] |
| AT1G31780 | -0,58 | 2,07E-55 | - | At1g31780 [Source:UniProtKB/TrEMBL;Acc:Q6NMI3] |
| AT1G31790 | 1,33 | 2,75E-08 | PCMP-A1 | Pentatricopeptide repeat-containing protein At1g31790 [Source:UniProtKB/Swiss-Prot;Acc:Q9C6R9] |
| AT1G31800 | 0,76 | 6,26E-07 | CYP97A3 | Protein LUTEIN DEFICIENT 5, chloroplastic [Source:UniProtKB/Swiss-Prot;Acc:Q93VK5] |
| AT1G31812 | -0,61 | 2,17E-50 | ACBP6 | ACBP6 [Source:UniProtKB/TrEMBL;Acc:A0A178WJ33] |
| AT1G31814 | 0,90 | 1,82E-18 | FRL2 | Inactive FRIGIDA-like protein 2 [Source:UniProtKB/Swiss-Prot;Acc:Q9C6S2] |
| AT1G31820 | 1,12 | 4,47E-04 | - | Probable polyamine transporter At1g31820 [Source:UniProtKB/Swiss-Prot;Acc:Q9C6S4] |
| AT1G31840 | 0,68 | 5,94E-06 | - | Putative pentatricopeptide repeat-containing protein At1g31840 [Source:UniProtKB/Swiss-Prot;Acc:Q9C6S6] |
| AT1G31880 | 1,20 | 3,28E-11 | NLM9 | DZC (Disease resistance/zinc finger/chromosome condensation-like region) domain containing protein [Source:TAIR;Acc:AT1G31880] |
| AT1G31885 | 1,73 | 4,31E-02 | NIP3-1 | Aquaporin NIP3-1 [Source:UniProtKB/Swiss-Prot;Acc:Q9C6T0] |
| AT1G32120 | -0,91 | 8,38E-03 | - | FUNCTIONS IN: molecular_function unknown; INVOLVED IN: biological_process unknown; LOCATED IN: membrane; EXPRESSED IN: 14 plant structures; EXPRESSED DURING: 4 anthesis, C globular stage, F mature embryo stage, petal differentiation and expansion st /.../ expanded cotyledon stage; CONTAINS InterPro DOMAIN/s: Aminotransferase-like, plant mobile domain (InterPro:IPR019557), Protein of unknown function DUF716 (InterPro:IPR006904); BEST Arabidopsis thaliana protein match is: Aminotransferase-like, plant mobile domain family protein (TAIR:AT1G51538.1); Ha. [Source:TAIR;Acc:AT1G32120] |
| AT1G32160 | 0,69 | 7,71E-10 | - | At1g32160/F3C3_6 [Source:UniProtKB/TrEMBL;Acc:Q9FVR1] |
| AT1G32172 | -3,27 | 2,58E-02 | - | other RNA [Source:TAIR;Acc:AT1G32172] |
| AT1G32210 | -1,10 | 1,91E-122 | DAD1 | Dolichyl-diphosphooligosaccharide--protein glycosyltransferase subunit DAD1 [Source:UniProtKB/Swiss-Prot;Acc:Q39080] |
| AT1G32220 | 0,61 | 6,90E-08 | - | Uncharacterized protein At1g32220, chloroplastic [Source:UniProtKB/Swiss-Prot;Acc:Q9FVR6] |
| AT1G32230 | 1,91 | 0,00E+00 | RCD1 | Poly [ADP-ribose] polymerase [Source:UniProtKB/TrEMBL;Acc:M5BF30] |
| AT1G32340 | 0,83 | 1,43E-29 | NHL8 | RBR-type E3 ubiquitin transferase [Source:UniProtKB/TrEMBL;Acc:Q9LQM5] |
| AT1G32450 | 4,26 | 1,53E-11 | NPF7.3 | Protein NRT1/ PTR FAMILY 7.3 [Source:UniProtKB/Swiss-Prot;Acc:Q9LQL2] |
| AT1G32520 | 1,62 | 9,33E-16 | - | TLDc domain protein [Source:UniProtKB/TrEMBL;Acc:Q682Q6] |
| AT1G32530 | 0,80 | 1,80E-41 | MIP1 | MND1-interacting protein 1 [Source:UniProtKB/Swiss-Prot;Acc:Q8RX22] |
| AT1G32580 | 0,73 | 4,93E-69 | MORF5 | Multiple organellar RNA editing factor 5, chloroplastic/mitochondrial [Source:UniProtKB/Swiss-Prot;Acc:Q9C7Y2] |
| AT1G32583 | 0,66 | 6,04E-04 | TDL1 | TPD1 protein homolog 1 [Source:UniProtKB/Swiss-Prot;Acc:Q1G3T1] |
| AT1G32610 | 1,17 | 9,10E-06 | - | At1g32610 [Source:UniProtKB/TrEMBL;Acc:Q9LPK0] |
| AT1G32630 | 0,73 | 6,05E-04 | - | At1g32630 [Source:UniProtKB/TrEMBL;Acc:Q9LPJ9] |
| AT1G32690 | -1,41 | 3,37E-17 | - | At1g32690 [Source:UniProtKB/TrEMBL;Acc:Q6NNH5] |
| AT1G32730 | -0,76 | 3,83E-13 | - | Electron carrier/iron ion-binding protein [Source:UniProtKB/TrEMBL;Acc:Q9LPJ1] |
| AT1G32860 | 1,21 | 2,96E-127 | - | Glucan endo-1,3-beta-glucosidase 11 [Source:UniProtKB/Swiss-Prot;Acc:Q8L868] |
| AT1G32870 | 4,16 | 0,00E+00 | ANAC13 | NAC domain protein 13 [Source:TAIR;Acc:AT1G32870] |
| AT1G32880 | 4,14 | 9,32E-24 | - | ARM repeat superfamily protein [Source:UniProtKB/TrEMBL;Acc:F4HPE4] |
| AT1G32900 | 0,65 | 2,27E-08 | GBSS1 | Granule-bound starch synthase 1, chloroplastic/amyloplastic [Source:UniProtKB/Swiss-Prot;Acc:Q9MAQ0] |
| AT1G32920 | 2,16 | 4,30E-251 | - | At1g32920/F9L11_25 [Source:UniProtKB/TrEMBL;Acc:Q93WI1] |
| AT1G32928 | 2,34 | 3,38E-23 | - | Avr9/Cf-9 rapidly elicited protein [Source:UniProtKB/TrEMBL;Acc:Q56XA0] |
| AT1G33030 | 1,93 | 6,53E-12 | - | At1g33030/F9L11_18 [Source:UniProtKB/TrEMBL;Acc:Q9MAP0] |
| AT1G33040 | -0,80 | 1,36E-56 | NACA5 | Nascent polypeptide-associated complex subunit alpha-like protein 5 [Source:UniProtKB/Swiss-Prot;Acc:Q8LGC6] |
| AT1G33060 | 0,63 | 5,50E-11 | NAC014 | NAC014 [Source:UniProtKB/TrEMBL;Acc:A0A178WRQ2] |
| AT1G33080 | 0,85 | 1,07E-16 | DTX23 | Protein DETOXIFICATION 23 [Source:UniProtKB/Swiss-Prot;Acc:Q8RXK1] |
| AT1G33090 | -5,01 | 4,22E-56 | DTX22 | Protein DETOXIFICATION 22 [Source:UniProtKB/Swiss-Prot;Acc:F4HPH1] |
| AT1G33100 | -3,31 | 1,61E-05 | DTX20 | Protein DETOXIFICATION 20 [Source:UniProtKB/Swiss-Prot;Acc:F4HPH2] |
| AT1G33110 | 1,50 | 2,24E-157 | DTX21 | Protein DETOXIFICATION [Source:UniProtKB/TrEMBL;Acc:A0A178WPH4] |
| AT1G33120 | -0,88 | 7,32E-100 | RPL9B | 60S ribosomal protein L9-1 [Source:UniProtKB/Swiss-Prot;Acc:P49209] |
| AT1G33140 | -1,03 | 9,52E-84 | RPL9B | 60S ribosomal protein L9-1 [Source:UniProtKB/Swiss-Prot;Acc:P49209] |
| AT1G33170 | 0,59 | 8,56E-11 | - | Probable methyltransferase PMT18 [Source:UniProtKB/Swiss-Prot;Acc:Q9C884] |
| AT1G33260 | 2,69 | 5,91E-10 | - | Probable receptor-like protein kinase At1g33260 [Source:UniProtKB/Swiss-Prot;Acc:P0DKI6] |
| AT1G33265 | -0,65 | 1,93E-34 | FAX4 | Protein FATTY ACID EXPORT 4, chloroplastic [Source:UniProtKB/Swiss-Prot;Acc:Q8LPG1] |
| AT1G33290 | 1,04 | 6,38E-43 | - | At1g33290 [Source:UniProtKB/TrEMBL;Acc:Q9C877] |
| AT1G33340 | 2,00 | 3,70E-10 | - | Putative clathrin assembly protein At1g33340 [Source:UniProtKB/Swiss-Prot;Acc:Q9C502] |
| AT1G33360 | -1,23 | 5,21E-148 | CLPX3 | CLP protease regulatory subunit CLPX3, mitochondrial [Source:UniProtKB/Swiss-Prot;Acc:Q66GN9] |
| AT1G33440 | -2,20 | 2,10E-05 | NPF4.4 | Protein NRT1/ PTR FAMILY 4.4 [Source:UniProtKB/Swiss-Prot;Acc:Q56XQ6] |
| AT1G33560 | 0,78 | 1,15E-22 | ADR1 | Disease resistance protein ADR1 [Source:UniProtKB/Swiss-Prot;Acc:Q9FW44] |
| AT1G33590 | -0,61 | 4,61E-24 | - | Leucine-rich repeat (LRR) family protein [Source:TAIR;Acc:AT1G33590] |
| AT1G33600 | -1,32 | 1,36E-04 | - | Leucine-rich repeat (LRR) family protein [Source:UniProtKB/TrEMBL;Acc:Q9FW48] |
| AT1G33670 | -8,34 | 1,74E-15 | - | Leucine-rich repeat (LRR) family protein [Source:UniProtKB/TrEMBL;Acc:Q4PT10] |
| AT1G33770 | 2,06 | 4,49E-05 | - | Protein kinase superfamily protein [Source:UniProtKB/TrEMBL;Acc:Q9LQ29] |
| AT1G33790 | 3,61 | 1,35E-96 | JAL4 | Jacalin-related lectin 4 [Source:UniProtKB/Swiss-Prot;Acc:Q9LQ31] |
| AT1G33800 | -1,65 | 3,72E-84 | GXM3 | GXMT1 [Source:UniProtKB/TrEMBL;Acc:A0A178W7W0] |
| AT1G33970 | 2,65 | 1,94E-09 | IAN9 | Immune-associated nucleotide-binding protein 9 [Source:UniProtKB/Swiss-Prot;Acc:F4HT21] |
| AT1G34000 | 0,80 | 1,33E-11 | OHP2 | OHP2 [Source:UniProtKB/TrEMBL;Acc:A0A178WAB5] |
| AT1G34010 | -0,60 | 2,60E-21 | - | unknown protein; BEST Arabidopsis thaliana protein match is: unknown protein (TAIR:AT1G22790.2); Ha. [Source:TAIR;Acc:AT1G34010] |
| AT1G34020 | -0,76 | 1,53E-58 | URGT6 | UDP-rhamnose/UDP-galactose transporter 6 [Source:UniProtKB/Swiss-Prot;Acc:Q9FDZ5] |
| AT1G34030 | -0,67 | 3,39E-58 | RPS18C | 40S ribosomal protein S18 [Source:UniProtKB/Swiss-Prot;Acc:P34788] |
| AT1G34040 | -0,77 | 1,37E-02 | TAR3 | Tryptophan aminotransferase-related protein 3 [Source:UniProtKB/Swiss-Prot;Acc:Q9FE98] |
| AT1G34060 | 1,27 | 1,90E-20 | TAR4 | Tryptophan aminotransferase-related protein 4 [Source:UniProtKB/Swiss-Prot;Acc:Q93Z38] |
| AT1G34130 | -0,83 | 2,84E-95 | STT3B | Glycosyltransferase (Fragment) [Source:UniProtKB/TrEMBL;Acc:W8PVA0] |
| AT1G34180 | 2,89 | 1,43E-30 | anac016 | NAC domain containing protein 16 [Source:TAIR;Acc:AT1G34180] |
| AT1G34200 | -0,73 | 4,68E-25 | - | At1g34200/F23M19.12 [Source:UniProtKB/TrEMBL;Acc:Q9ASQ9] |
| AT1G34300 | 0,68 | 1,34E-14 | - | G-type lectin S-receptor-like serine/threonine-protein kinase At1g34300 [Source:UniProtKB/Swiss-Prot;Acc:Q9XID3] |
| AT1G34315 | 0,85 | 1,22E-47 | - | unknown protein; FUNCTIONS IN: molecular_function unknown; INVOLVED IN: biological_process unknown; LOCATED IN: endomembrane system; BEST Arabidopsis thaliana protein match is: unknown protein (TAIR:AT3G31400.1); Ha. [Source:TAIR;Acc:AT1G34315] |
| AT1G34317 | 1,13 | 1,67E-14 | - | - |
| AT1G34340 | -0,88 | 2,20E-33 | - | Alpha/beta-Hydrolases superfamily protein [Source:UniProtKB/TrEMBL;Acc:Q9XID7] |
| AT1G34350 | -0,64 | 3,23E-33 | - | unknown protein; Ha. [Source:TAIR;Acc:AT1G34350] |
| AT1G34380 | 0,60 | 1,34E-04 | - | 5'-3' exonuclease family protein [Source:UniProtKB/TrEMBL;Acc:Q66GR7] |
| AT1G34430 | -1,19 | 1,34E-140 | EMB3003 | Dihydrolipoyllysine-residue acetyltransferase component 5 of pyruvate dehydrogenase complex, chloroplastic [Source:UniProtKB/Swiss-Prot;Acc:Q9C8P0] |
| AT1G34460 | 0,70 | 3,96E-03 | CYCB1;5 | CYCLIN B1;5 [Source:TAIR;Acc:AT1G34460] |
| AT1G34490 | -4,13 | 3,73E-30 | - | Putative long-chain-alcohol O-fatty-acyltransferase 10 [Source:UniProtKB/Swiss-Prot;Acc:Q3ED15] |
| AT1G34500 | -6,03 | 8,67E-18 | - | Probable long-chain-alcohol O-fatty-acyltransferase 9 [Source:UniProtKB/Swiss-Prot;Acc:Q4PT07] |
| AT1G34550 | -0,82 | 6,26E-69 | EMB2756 | Protein of unknown function (DUF616) [Source:TAIR;Acc:AT1G34550] |
| AT1G34640 | -0,68 | 1,81E-03 | - | At1g34640 [Source:UniProtKB/TrEMBL;Acc:Q9LNM6] |
| AT1G34760 | -4,27 | 2,34E-06 | GRF11 | general regulatory factor 11 [Source:TAIR;Acc:AT1G34760] |
| AT1G35140 | 4,57 | 3,92E-206 | EXL1 | Protein EXORDIUM-like 1 [Source:UniProtKB/Swiss-Prot;Acc:Q9C6E4] |
| AT1G35160 | -0,99 | 2,01E-155 | GF14 PHI | GF14 protein phi chain [Source:UniProtKB/TrEMBL;Acc:F4HWQ5] |
| AT1G35183 | -4,22 | 2,48E-02 | - | unknown protein; Ha. [Source:TAIR;Acc:AT1G35183] |
| AT1G35190 | 1,51 | 2,44E-41 | - | 2-oxoglutarate (2OG) and Fe(II)-dependent oxygenase superfamily protein [Source:UniProtKB/TrEMBL;Acc:Q9C6F0] |
| AT1G35250 | -5,20 | 2,24E-04 | ALT2 | Acyl-acyl carrier protein thioesterase ATL2, chloroplastic [Source:UniProtKB/Swiss-Prot;Acc:Q9C7I8] |
| AT1G35255 | -2,39 | 8,15E-03 | - | Transmembrane protein [Source:UniProtKB/TrEMBL;Acc:Q1G3E8] |
| AT1G35260 | -4,20 | 1,06E-23 | MLP165 | MLP-like protein 165 [Source:UniProtKB/Swiss-Prot;Acc:Q9C7I7] |
| AT1G35350 | -6,08 | 4,53E-08 | PHO1-H8 | Phosphate transporter PHO1 homolog 8 [Source:UniProtKB/Swiss-Prot;Acc:Q6R8G2] |
| AT1G35460 | 0,61 | 3,45E-05 | BHLH80 | Transcription factor bHLH80 [Source:UniProtKB/Swiss-Prot;Acc:Q9C8P8] |
| AT1G35515 | 1,13 | 1,25E-03 | MYB8 | Transcription factor MYB8 [Source:UniProtKB/Swiss-Prot;Acc:Q9SDS8] |
| AT1G35516 | 0,59 | 1,86E-28 | - | myb-like transcription factor family protein [Source:TAIR;Acc:AT1G35516] |
| AT1G35620 | -0,93 | 2,66E-89 | PDIL5-2 | Protein disulfide-isomerase 5-2 [Source:UniProtKB/Swiss-Prot;Acc:Q94F09] |
| AT1G35660 | 1,36 | 2,28E-61 | - | unknown protein; FUNCTIONS IN: molecular_function unknown; INVOLVED IN: biological_process unknown; LOCATED IN: plasma membrane; EXPRESSED IN: 19 plant structures; EXPRESSED DURING: 11 growth stages; Ha. [Source:TAIR;Acc:AT1G35660] |
| AT1G35670 | -0,94 | 8,11E-43 | CPK11 | Calcium-dependent protein kinase 11 [Source:UniProtKB/Swiss-Prot;Acc:Q39016] |
| AT1G35720 | -3,38 | 3,62E-100 | ANN1 | Annexin [Source:UniProtKB/TrEMBL;Acc:A0A178WGZ8] |
| AT1G35730 | 2,95 | 2,50E-02 | APUM9 | pumilio 9 [Source:TAIR;Acc:AT1G35730] |
| AT1G35880 | 4,38 | 9,07E-04 | - | Uncharacterized protein F10O5.5 [Source:UniProtKB/TrEMBL;Acc:Q9C8B6] |
| AT1G36070 | 0,60 | 3,48E-20 | - | At1g36070 [Source:UniProtKB/TrEMBL;Acc:Q0V7U5] |
| AT1G36180 | 4,03 | 0,00E+00 | ACC2 | Acetyl-CoA carboxylase 2 [Source:UniProtKB/Swiss-Prot;Acc:F4I1L3] |
| AT1G36320 | 0,81 | 1,79E-28 | - | At1g36320/F7F23_4 [Source:UniProtKB/TrEMBL;Acc:Q9C8X8] |
| AT1G36370 | 0,99 | 4,37E-24 | SHM7 | Serine hydroxymethyltransferase 7 [Source:UniProtKB/Swiss-Prot;Acc:Q84WV0] |
| AT1G36622 | 2,82 | 2,90E-17 | - | Transmembrane protein [Source:UniProtKB/TrEMBL;Acc:Q1G3E7] |
| AT1G36675 | -4,44 | 4,87E-03 | - | Glycine-rich protein [Source:UniProtKB/TrEMBL;Acc:F4I389] |
| AT1G36940 | 1,25 | 1,19E-08 | - | unknown protein; Ha. [Source:TAIR;Acc:AT1G36940] |
| AT1G37130 | 0,97 | 1,07E-123 | NIA2 | Nitrate reductase [NADH] 2 [Source:UniProtKB/Swiss-Prot;Acc:P11035] |
| AT1G37150 | 1,31 | 6,76E-20 | HCS2 | holocarboxylase synthetase 2 [Source:TAIR;Acc:AT1G37150] |
| AT1G41830 | -0,73 | 1,26E-38 | SKS6 | SKS6 [Source:UniProtKB/TrEMBL;Acc:A0A178W7S3] |
| AT1G41880 | -0,84 | 2,08E-82 | RPL35AB | 60S ribosomal protein L35a-2 [Source:UniProtKB/Swiss-Prot;Acc:Q9FZH0] |
| AT1G42430 | -0,63 | 1,59E-19 | - | unknown protein; BEST Arabidopsis thaliana protein match is: unknown protein (TAIR:AT3G55760.3); Ha. [Source:TAIR;Acc:AT1G42430] |
| AT1G42550 | 3,29 | 3,99E-82 | PMI1 | Protein PLASTID MOVEMENT IMPAIRED 1 [Source:UniProtKB/Swiss-Prot;Acc:Q9C8E6] |
| AT1G42980 | 3,34 | 7,97E-07 | FH12 | Formin-like protein 12 [Source:UniProtKB/Swiss-Prot;Acc:Q9C7S1] |
| AT1G43005 | 3,57 | 1,23E-02 | - | FUNCTIONS IN: molecular_function unknown; INVOLVED IN: biological_process unknown; LOCATED IN: cellular_component unknown; BEST Arabidopsis thaliana protein match is: F-box family protein (TAIR:AT1G61060.1); Ha. [Source:TAIR;Acc:AT1G43005] |
| AT1G43160 | 1,54 | 1,28E-15 | RAP2-6 | Ethylene-responsive transcription factor RAP2-6 [Source:UniProtKB/Swiss-Prot;Acc:Q7G1L2] |
| AT1G43245 | 1,02 | 3,72E-15 | - | SET domain-containing protein [Source:TAIR;Acc:AT1G43245] |
| AT1G43700 | 0,73 | 1,26E-17 | VIP1 | Transcription factor VIP1 [Source:UniProtKB/Swiss-Prot;Acc:Q9MA75] |
| AT1G43710 | -0,75 | 9,91E-70 | SDC | SDC1 [Source:UniProtKB/TrEMBL;Acc:A0A178WA97] |
| AT1G43765 | 1,26 | 1,02E-10 | - | other RNA [Source:TAIR;Acc:AT1G43765] |
| AT1G43770 | 0,85 | 4,56E-11 | - | RING/FYVE/PHD zinc finger superfamily protein [Source:UniProtKB/TrEMBL;Acc:B3H578] |
| AT1G43910 | 2,62 | 0,00E+00 | - | AAA-ATPase At1g43910 [Source:UniProtKB/Swiss-Prot;Acc:Q9LP11] |
| AT1G43980 | 0,95 | 3,56E-07 | PCMP-E58 | Pentatricopeptide repeat-containing protein At1g43980, mitochondrial [Source:UniProtKB/Swiss-Prot;Acc:Q9LP03] |
| AT1G44020 | -3,28 | 8,28E-15 | - | Cysteine/Histidine-rich C1 domain family protein [Source:UniProtKB/TrEMBL;Acc:F4IEE1] |
| AT1G44100 | 1,04 | 1,76E-37 | AAP5 | Amino acid permease 5 [Source:UniProtKB/Swiss-Prot;Acc:Q8GUM3] |
| AT1G44120 | -3,33 | 4,25E-02 | - | Armadillo/beta-catenin-like repeat ; C2 calcium/lipid-binding domain (CaLB) protein [Source:TAIR;Acc:AT1G44120] |
| AT1G44180 | -2,98 | 9,75E-03 | - | Aminoacylase, putative [Source:UniProtKB/TrEMBL;Acc:Q9C6Y8] |
| AT1G44318 | -1,68 | 1,86E-16 | HEMB2 | Probable delta-aminolevulinic acid dehydratase 2, chloroplastic [Source:UniProtKB/Swiss-Prot;Acc:Q94LA4] |
| AT1G44350 | 1,15 | 1,41E-25 | ILL6 | IAA-amino acid hydrolase [Source:UniProtKB/TrEMBL;Acc:Q0WNN8] |
| AT1G44414 | 3,92 | 5,81E-05 | - | At1g44414 [Source:UniProtKB/TrEMBL;Acc:Q94LA6] |
| AT1G44446 | 0,74 | 7,19E-09 | CAO | CH1 [Source:UniProtKB/TrEMBL;Acc:A0A178W6L1] |
| AT1G44542 | -2,74 | 2,03E-02 | CYCLASE3 | Cyclase-like protein 3 [Source:UniProtKB/Swiss-Prot;Acc:Q94LA9] |
| AT1G44740 | 0,65 | 1,06E-18 | - | unknown protein; FUNCTIONS IN: molecular_function unknown; INVOLVED IN: biological_process unknown; LOCATED IN: mitochondrion; EXPRESSED IN: embryo, leaf whorl, flower, seed; EXPRESSED DURING: F mature embryo stage, petal differentiation and expansi /.../ge, E expanded cotyledon stage, D bilateral stage; Ha. [Source:TAIR;Acc:AT1G44740] |
| AT1G44770 | 0,59 | 2,38E-16 | - | At1g44770 [Source:UniProtKB/TrEMBL;Acc:Q84WK2] |
| AT1G44800 | 2,42 | 1,16E-07 | - | WAT1-related protein At1g44800 [Source:UniProtKB/Swiss-Prot;Acc:Q9LPF1] |
| AT1G44830 | -1,17 | 1,65E-25 | ERF014 | Ethylene-responsive transcription factor ERF014 [Source:UniProtKB/Swiss-Prot;Acc:Q9LPE8] |
| AT1G44970 | 2,22 | 3,08E-13 | PER9 | Peroxidase 9 [Source:UniProtKB/Swiss-Prot;Acc:Q96512] |
| AT1G45000 | -0,77 | 8,43E-124 | RPT4B | 26S proteasome regulatory subunit S10B homolog B [Source:UniProtKB/Swiss-Prot;Acc:Q9MAK9] |
| AT1G45015 | 1,36 | 4,20E-07 | - | At1g45015 [Source:UniProtKB/TrEMBL;Acc:Q9MAK7] |
| AT1G45110 | 0,98 | 1,94E-30 | - | At1g45110 [Source:UniProtKB/TrEMBL;Acc:Q6NQA0] |
| AT1G45145 | -0,60 | 1,00E-12 | TRX5 | Thioredoxin H5 [Source:UniProtKB/Swiss-Prot;Acc:Q39241] |
| AT1G45150 | 0,59 | 1,70E-13 | - | Alpha-1,6-mannosyl-glycoprotein 2-beta-N-acetylglucosaminyltransferase [Source:UniProtKB/TrEMBL;Acc:Q7Y231] |
| AT1G45207 | -0,73 | 1,62E-18 | - | Remorin family protein [Source:UniProtKB/TrEMBL;Acc:F4HRB5] |
| AT1G45229 | 1,33 | 5,72E-19 | - | unknown protein; FUNCTIONS IN: molecular_function unknown; INVOLVED IN: biological_process unknown; LOCATED IN: cellular_component unknown; Ha. [Source:TAIR;Acc:AT1G45229] |
| AT1G45234 | 0,89 | 5,00E-04 | - | pre-tRNA [Source:TAIR;Acc:AT1G45234] |
| AT1G45238 | 1,78 | 2,58E-02 | - | pre-tRNA [Source:TAIR;Acc:AT1G45238] |
| AT1G45248 | 0,79 | 1,88E-09 | - | Nucleolar histone methyltransferase-related protein [Source:UniProtKB/TrEMBL;Acc:F4HRC7] |
| AT1G45474 | 4,08 | 3,23E-17 | LHCA5 | Photosystem I chlorophyll a/b-binding protein 5, chloroplastic [Source:UniProtKB/Swiss-Prot;Acc:Q9C639] |
| AT1G45976 | 0,98 | 9,84E-43 | SBP1 | SBP1 [Source:UniProtKB/TrEMBL;Acc:A0A178WK23] |
| AT1G46768 | 2,82 | 5,80E-13 | RAP2-1 | Ethylene-responsive transcription factor RAP2-1 [Source:UniProtKB/Swiss-Prot;Acc:Q8LC30] |
| AT1G47128 | 1,54 | 7,08E-180 | RD21A | RD21A [Source:UniProtKB/TrEMBL;Acc:A0A178WIH7] |
| AT1G47200 | -0,79 | 1,58E-69 | WPP2 | WPP domain-containing protein 2 [Source:UniProtKB/Swiss-Prot;Acc:Q9C500] |
| AT1G47240 | -0,68 | 9,44E-37 | NRAMP2 | Metal transporter Nramp2 [Source:UniProtKB/Swiss-Prot;Acc:Q9C6B2] |
| AT1G47250 | -0,83 | 8,45E-16 | PAF2 | Proteasome subunit alpha type-1-B [Source:UniProtKB/Swiss-Prot;Acc:O23712] |
| AT1G47270 | 0,76 | 9,00E-15 | TULP6 | Tubby-like F-box protein 6 [Source:UniProtKB/Swiss-Prot;Acc:Q0WPY0] |
| AT1G47271 | 0,68 | 4,96E-03 | - | Cystathionine beta-synthase (CBS) family protein [Source:UniProtKB/TrEMBL;Acc:B3H6Z2] |
| AT1G47290 | -0,73 | 4,24E-64 | 3BETAHSD/D1 | 3beta-hydroxysteroid-dehydrogenase/decarboxylase isoform 1 [Source:UniProtKB/Swiss-Prot;Acc:Q9FX01] |
| AT1G47395 | 5,51 | 2,14E-04 | - | At1g47390 [Source:UniProtKB/TrEMBL;Acc:Q8GUL3] |
| AT1G47400 | 4,02 | 2,87E-04 | - | unknown protein; BEST Arabidopsis thaliana protein match is: unknown protein (TAIR:AT1G47395.1); Ha. [Source:TAIR;Acc:AT1G47400] |
| AT1G47480 | -0,93 | 8,91E-09 | CXE2 | Probable carboxylesterase 2 [Source:UniProtKB/Swiss-Prot;Acc:Q9SX78] |
| AT1G47510 | 2,64 | 3,20E-237 | IP5P11 | Type IV inositol polyphosphate 5-phosphatase 11 [Source:UniProtKB/Swiss-Prot;Acc:Q5EAF2] |
| AT1G47530 | -0,92 | 2,36E-94 | DTX33 | Protein DETOXIFICATION 33 [Source:UniProtKB/Swiss-Prot;Acc:Q9SX83] |
| AT1G47600 | -0,82 | 3,43E-12 | TGG4 | Myrosinase 4 [Source:UniProtKB/Swiss-Prot;Acc:Q8GRX1] |
| AT1G47640 | -0,74 | 3,59E-106 | - | At1g47640/F16N3_6 [Source:UniProtKB/TrEMBL;Acc:Q9SX96] |
| AT1G47740 | -0,60 | 1,43E-30 | - | PPPDE putative thiol peptidase family protein [Source:UniProtKB/TrEMBL;Acc:Q8RXP3] |
| AT1G47830 | -0,70 | 2,30E-31 | AP17 | AP complex subunit sigma [Source:UniProtKB/TrEMBL;Acc:A0A178W398] |
| AT1G47845 | 3,07 | 3,86E-02 | - | Phosphotransferase [Source:UniProtKB/TrEMBL;Acc:A0A1P8ARU2] |
| AT1G47915 | 3,45 | 1,21E-05 | - | Putative F-box protein At1g47915 [Source:UniProtKB/Swiss-Prot;Acc:Q3E6X9] |
| AT1G47920 | 3,15 | 4,26E-11 | - | RNI-like superfamily protein [Source:UniProtKB/TrEMBL;Acc:F4HV53] |
| AT1G48040 | -0,64 | 1,24E-20 | - | Probable protein phosphatase 2C 13 [Source:UniProtKB/Swiss-Prot;Acc:Q9LNF4] |
| AT1G48070 | -1,73 | 7,66E-18 | - | At1g48070 [Source:UniProtKB/TrEMBL;Acc:Q6NM39] |
| AT1G48100 | 1,84 | 1,79E-10 | - | Polygalacturonase At1g48100 [Source:UniProtKB/Swiss-Prot;Acc:Q949Z1] |
| AT1G48110 | -1,06 | 4,93E-125 | ECT7 | evolutionarily conserved C-terminal region 7 [Source:TAIR;Acc:AT1G48110] |
| AT1G48140 | -0,59 | 5,83E-24 | DPMS3 | DPMS3 [Source:UniProtKB/TrEMBL;Acc:A0A178WDH1] |
| AT1G48210 | 1,46 | 1,37E-34 | - | Protein kinase superfamily protein [Source:UniProtKB/TrEMBL;Acc:F4HWU0] |
| AT1G48230 | -0,91 | 5,97E-82 | - | Probable sugar phosphate/phosphate translocator At1g48230 [Source:UniProtKB/Swiss-Prot;Acc:Q9LNH5] |
| AT1G48300 | 2,97 | 1,72E-39 | - | unknown protein; FUNCTIONS IN: molecular_function unknown; INVOLVED IN: biological_process unknown; LOCATED IN: endomembrane system; EXPRESSED IN: 24 plant structures; EXPRESSED DURING: 15 growth stages; Ha. [Source:TAIR;Acc:AT1G48300] |
| AT1G48350 | -0,72 | 1,69E-17 | RPL18 | Uncharacterized protein At1g48350 [Source:UniProtKB/TrEMBL;Acc:Q0WWC5] |
| AT1G48390 | 1,46 | 1,23E-13 | - | RNI-like superfamily protein [Source:UniProtKB/TrEMBL;Acc:F4HYE7] |
| AT1G48400 | 2,58 | 8,34E-05 | - | F-box/RNI-like/FBD-like domains-containing protein [Source:TAIR;Acc:AT1G48400] |
| AT1G48440 | -0,64 | 1,10E-37 | - | At1g48440 [Source:UniProtKB/TrEMBL;Acc:Q8LDS7] |
| AT1G48450 | 1,06 | 6,86E-41 | - | Alanine-tRNA ligase, putative (DUF760) [Source:UniProtKB/TrEMBL;Acc:Q9LP80] |
| AT1G48470 | -1,28 | 5,96E-13 | GLN1-5 | Glutamine synthetase cytosolic isozyme 1-5 [Source:UniProtKB/Swiss-Prot;Acc:Q8GXW5] |
| AT1G48560 | -0,66 | 4,27E-05 | - | unknown protein; Ha. [Source:TAIR;Acc:AT1G48560] |
| AT1G48605 | 0,98 | 3,05E-36 | HAL3B | Probable phosphopantothenoylcysteine decarboxylase [Source:UniProtKB/Swiss-Prot;Acc:P94063] |
| AT1G48610 | -0,96 | 1,64E-57 | - | Putative DNA-binding protein At1g48610 [Source:UniProtKB/Swiss-Prot;Acc:Q94AD1] |
| AT1G48630 | -1,06 | 3,42E-97 | RACK1B | RACK1B_AT [Source:UniProtKB/TrEMBL;Acc:A0A178W908] |
| AT1G48640 | -0,99 | 1,73E-03 | - | Lysine histidine transporter-like 1 [Source:UniProtKB/Swiss-Prot;Acc:Q9C733] |
| AT1G48670 | -1,38 | 2,51E-06 | - | Auxin-responsive GH3 family protein [Source:UniProtKB/TrEMBL;Acc:Q9C736] |
| AT1G48840 | 0,92 | 4,86E-15 | - | Heat-inducible transcription repressor (DUF639) [Source:UniProtKB/TrEMBL;Acc:Q8VXZ2] |
| AT1G48850 | -0,62 | 1,32E-61 | EMB1144 | Chorismate synthase, chloroplastic [Source:UniProtKB/Swiss-Prot;Acc:P57720] |
| AT1G48870 | 4,48 | 4,02E-10 | - | Transducin/WD40 repeat-like superfamily protein [Source:UniProtKB/TrEMBL;Acc:Q9FVP7] |
| AT1G48880 | -0,65 | 2,47E-13 | TBL7 | Protein trichome berefringence-like 7 [Source:UniProtKB/Swiss-Prot;Acc:F4I037] |
| AT1G48900 | -0,66 | 5,22E-50 | SRP-54C | Signal recognition particle 54 kDa protein 3 [Source:UniProtKB/Swiss-Prot;Acc:P49967] |
| AT1G48930 | -3,43 | 3,18E-15 | AtGH9C1 | Endoglucanase 5 [Source:UniProtKB/Swiss-Prot;Acc:Q9M995] |
| AT1G48960 | 0,84 | 5,35E-07 | - | Adenine nucleotide alpha hydrolases-like superfamily protein [Source:UniProtKB/TrEMBL;Acc:Q67Y32] |
| AT1G49010 | 0,70 | 8,39E-04 | - | At1g49010 [Source:UniProtKB/TrEMBL;Acc:Q9M9A3] |
| AT1G49030 | -3,46 | 4,47E-31 | PCR6 | Protein PLANT CADMIUM RESISTANCE 6 [Source:UniProtKB/Swiss-Prot;Acc:Q9M9A5] |
| AT1G49032 | 0,86 | 6,67E-03 | - | Putative uncharacterized protein [Source:UniProtKB/TrEMBL;Acc:Q8GX22] |
| AT1G49160 | 1,46 | 6,84E-52 | WNK7 | Protein kinase superfamily protein [Source:TAIR;Acc:AT1G49160] |
| AT1G49225 | -1,53 | 6,02E-04 | - | - |
| AT1G49330 | -4,87 | 1,97E-03 | - | F13F21.24 protein [Source:UniProtKB/TrEMBL;Acc:Q9XIA0] |
| AT1G49340 | -0,59 | 3,48E-37 | PI4KA1 | Phosphatidylinositol 4-kinase alpha 1 [Source:UniProtKB/Swiss-Prot;Acc:Q9SXA1] |
| AT1G49360 | 0,70 | 4,28E-07 | - | F-box protein At1g49360 [Source:UniProtKB/Swiss-Prot;Acc:Q9XIA2] |
| AT1G49390 | 0,87 | 3,41E-17 | - | 2-oxoglutarate (2OG) and Fe(II)-dependent oxygenase superfamily protein [Source:UniProtKB/TrEMBL;Acc:Q9XIA5] |
| AT1G49405 | 1,28 | 5,62E-05 | - | CASP-like protein 5C3 [Source:UniProtKB/Swiss-Prot;Acc:Q3ECT8] |
| AT1G49410 | -0,84 | 5,65E-57 | TOM6 | Mitochondrial import receptor subunit TOM6 homolog [Source:UniProtKB/Swiss-Prot;Acc:Q9XIA7] |
| AT1G49430 | -3,96 | 2,59E-02 | LACS2 | Long chain acyl-CoA synthetase 2 [Source:UniProtKB/Swiss-Prot;Acc:Q9XIA9] |
| AT1G49475 | 0,66 | 5,95E-06 | - | B3 domain-containing protein At1g49475 [Source:UniProtKB/Swiss-Prot;Acc:Q9XIB4] |
| AT1G49500 | -1,19 | 1,74E-04 | - | At1g49500/F13F21_6 [Source:UniProtKB/TrEMBL;Acc:Q9XIB7] |
| AT1G49510 | -0,66 | 3,88E-21 | emb1273 | At1g49510 [Source:UniProtKB/TrEMBL;Acc:Q9XIB8] |
| AT1G49580 | -0,71 | 5,11E-30 | CRK8 | CDPK-related kinase 8 [Source:UniProtKB/Swiss-Prot;Acc:Q9FX86] |
| AT1G49600 | -0,76 | 3,28E-134 | ATRBP47A | RNA-binding protein 47A [Source:TAIR;Acc:AT1G49600] |
| AT1G49610 | 1,73 | 2,03E-05 | - | F-box family protein [Source:TAIR;Acc:AT1G49610] |
| AT1G49650 | -0,64 | 1,42E-03 | CXE4 | Probable carboxylesterase 4, mitochondrial [Source:UniProtKB/Swiss-Prot;Acc:Q9FX93] |
| AT1G49660 | -0,61 | 1,49E-26 | CXE5 | Probable carboxylesterase 5 [Source:UniProtKB/Swiss-Prot;Acc:Q9FX94] |
| AT1G49670 | -0,83 | 3,17E-90 | NQR | ARP protein (REF) [Source:TAIR;Acc:AT1G49670] |
| AT1G49720 | 1,26 | 6,34E-64 | ABF1 | Abscisic acid responsive element-binding factor 1 [Source:UniProtKB/TrEMBL;Acc:F4I3C9] |
| AT1G49730 | -1,41 | 2,79E-52 | - | Protein kinase superfamily protein [Source:UniProtKB/TrEMBL;Acc:F4I3D1] |
| AT1G49740 | 1,50 | 1,11E-65 | - | F14J22.5 protein [Source:UniProtKB/TrEMBL;Acc:Q9FXA0] |
| AT1G49870 | -1,09 | 1,14E-26 | - | Myosin-2 heavy chain-like protein [Source:UniProtKB/TrEMBL;Acc:Q9C6C7] |
| AT1G49880 | 0,91 | 3,97E-11 | ERV1 | FAD-linked sulfhydryl oxidase ERV1 [Source:UniProtKB/Swiss-Prot;Acc:Q8GXX0] |
| AT1G49900 | 1,05 | 1,40E-02 | - | C2H2 type zinc finger transcription factor family [Source:UniProtKB/TrEMBL;Acc:Q9C538] |
| AT1G50010 | -0,83 | 8,83E-60 | TUBA4 | Tubulin alpha-2 chain [Source:UniProtKB/Swiss-Prot;Acc:B9DGT7] |
| AT1G50040 | 1,41 | 6,14E-11 | - | F2J10.8 protein [Source:UniProtKB/TrEMBL;Acc:Q9LPM5] |
| AT1G50055 | 0,60 | 4,05E-16 | TAS1B | TAS1B; other RNA [Source:TAIR;Acc:AT1G50055] |
| AT1G50060 | -1,52 | 2,17E-87 | - | CAP (Cysteine-rich secretory proteins, Antigen 5, and Pathogenesis-related 1 protein) superfamily protein [Source:UniProtKB/TrEMBL;Acc:Q9LPM7] |
| AT1G50090 | 1,12 | 3,40E-02 | BCAT7 | Putative branched-chain-amino-acid aminotransferase 7 [Source:UniProtKB/Swiss-Prot;Acc:Q9LPM8] |
| AT1G50110 | -0,83 | 3,81E-38 | BCAT6 | Branched-chain-amino-acid aminotransferase 6 [Source:UniProtKB/Swiss-Prot;Acc:Q9LPM9] |
| AT1G50180 | 1,12 | 7,33E-13 | - | Putative disease resistance protein At1g50180 [Source:UniProtKB/Swiss-Prot;Acc:Q9SX38] |
| AT1G50240 | -0,74 | 2,08E-23 | TIO | Serine/threonine-protein kinase TIO [Source:UniProtKB/Swiss-Prot;Acc:Q2QAV0] |
| AT1G50250 | 0,90 | 3,58E-33 | FTSH1 | ATP-dependent zinc metalloprotease FTSH 1, chloroplastic [Source:UniProtKB/Swiss-Prot;Acc:Q39102] |
| AT1G50400 | 3,56 | 0,00E+00 | TOM40-2 | Probable mitochondrial import receptor subunit TOM40-2 [Source:UniProtKB/Swiss-Prot;Acc:Q9SX55] |
| AT1G50420 | 0,72 | 5,63E-25 | SCL3 | Scarecrow-like protein 3 [Source:UniProtKB/Swiss-Prot;Acc:Q9LPR8] |
| AT1G50440 | -0,60 | 1,95E-15 | - | At1g50440 [Source:UniProtKB/TrEMBL;Acc:Q9LPR9] |
| AT1G50580 | -0,93 | 7,46E-24 | UGT79B5 | UDP-glycosyltransferase 79B5 [Source:UniProtKB/Swiss-Prot;Acc:Q9LPS8] |
| AT1G50640 | 0,77 | 8,95E-43 | ERF3 | Uncharacterized protein At1g50640 (Fragment) [Source:UniProtKB/TrEMBL;Acc:C0SV01] |
| AT1G50670 | -0,78 | 3,55E-49 | - | F11F12.1 protein [Source:UniProtKB/TrEMBL;Acc:Q9LPT6] |
| AT1G50750 | 1,00 | 6,78E-03 | - | Plant mobile domain protein family [Source:TAIR;Acc:AT1G50750] |
| AT1G50760 | -2,71 | 4,44E-03 | - | Aminotransferase-like, plant mobile domain family protein [Source:UniProtKB/TrEMBL;Acc:Q9C6K2] |
| AT1G50830 | 3,08 | 4,63E-02 | - | Aminotransferase-like, plant mobile domain family protein [Source:UniProtKB/TrEMBL;Acc:Q9C6J6] |
| AT1G50840 | 1,06 | 1,11E-59 | POLGAMMA2 | polymerase gamma 2 [Source:TAIR;Acc:AT1G50840] |
| AT1G50900 | -1,44 | 1,42E-08 | LTD | Protein LHCP TRANSLOCATION DEFECT [Source:UniProtKB/Swiss-Prot;Acc:Q8VY88] |
| AT1G50910 | 0,58 | 9,88E-11 | - | Uncharacterized protein At1g50910 [Source:UniProtKB/TrEMBL;Acc:Q56Y76] |
| AT1G50930 | -2,93 | 2,76E-02 | VUP2 | Vascular-related unknown protein 2 [Source:UniProtKB/Swiss-Prot;Acc:Q9C6I7] |
| AT1G50970 | 1,28 | 3,80E-34 | - | Vacuolar protein sorting-associated protein 53 B [Source:UniProtKB/Swiss-Prot;Acc:F4I7Y2] |
| AT1G51035 | 0,69 | 1,94E-03 | - | unknown protein. [Source:TAIR;Acc:AT1G51035] |
| AT1G51060 | -1,49 | 1,43E-150 | HTA10 | Probable histone H2A.1 [Source:UniProtKB/Swiss-Prot;Acc:Q9C681] |
| AT1G51090 | 3,10 | 1,15E-02 | - | Heavy metal transport/detoxification superfamily protein [Source:UniProtKB/TrEMBL;Acc:Q9C684] |
| AT1G51100 | 1,19 | 2,14E-07 | - | CRR41 [Source:UniProtKB/TrEMBL;Acc:A0A178W725] |
| AT1G51110 | 0,82 | 7,53E-04 | PAP12 | Probable plastid-lipid-associated protein 12, chloroplastic [Source:UniProtKB/Swiss-Prot;Acc:Q8LAP6] |
| AT1G51130 | 0,85 | 1,35E-14 | NSE4A | Non-structural maintenance of chromosomes element 4 homolog A [Source:UniProtKB/Swiss-Prot;Acc:Q9C689] |
| AT1G51200 | 0,90 | 1,16E-126 | SAP2 | Zinc finger A20 and AN1 domain-containing stress-associated protein 2 [Source:UniProtKB/Swiss-Prot;Acc:Q8H0X0] |
| AT1G51260 | -2,27 | 3,52E-56 | LPAT3 | 1-acyl-sn-glycerol-3-phosphate acyltransferase 3 [Source:UniProtKB/Swiss-Prot;Acc:Q9SYC8] |
| AT1G51270 | -1,58 | 6,81E-03 | - | Vesicle-associated protein 1-4 [Source:UniProtKB/TrEMBL;Acc:F4I818] |
| AT1G51310 | 0,65 | 3,16E-17 | - | tRNA (5-methylaminomethyl-2-thiouridylate)-methyltransferase [Source:UniProtKB/TrEMBL;Acc:F4I824] |
| AT1G51340 | 3,24 | 2,21E-23 | DTX42 | Protein DETOXIFICATION [Source:UniProtKB/TrEMBL;Acc:A0A178W9H8] |
| AT1G51355 | -1,71 | 4,10E-09 | SMR9 | Cyclin-dependent protein kinase inhibitor SMR9 [Source:UniProtKB/Swiss-Prot;Acc:Q3ECS5] |
| AT1G51430 | 0,83 | 7,45E-46 | - | unknown protein; BEST Arabidopsis thaliana protein match is: unknown protein (TAIR:AT3G28370.1); Ha. [Source:TAIR;Acc:AT1G51430] |
| AT1G51470 | -1,07 | 1,02E-16 | TGG5 | Myrosinase 5 [Source:UniProtKB/Swiss-Prot;Acc:Q3ECS3] |
| AT1G51480 | 2,36 | 1,65E-02 | - | Probable disease resistance protein At1g51480 [Source:UniProtKB/Swiss-Prot;Acc:Q9C8K0] |
| AT1G51500 | 3,24 | 6,49E-10 | ABCG12 | WBC12 [Source:UniProtKB/TrEMBL;Acc:A0A178W3S0] |
| AT1G51510 | -0,70 | 3,26E-50 | Y14 | Y14 [Source:UniProtKB/TrEMBL;Acc:A0A178WGD6] |
| AT1G51520 | 0,82 | 2,44E-07 | - | RNA-binding (RRM/RBD/RNP motifs) family protein [Source:UniProtKB/TrEMBL;Acc:F4I9J8] |
| AT1G51530 | 2,46 | 1,93E-02 | - | RNA-binding (RRM/RBD/RNP motifs) family protein [Source:UniProtKB/TrEMBL;Acc:F4I9K0] |
| AT1G51590 | -0,61 | 4,95E-29 | MNS1 | Mannosyl-oligosaccharide 1,2-alpha-mannosidase MNS1 [Source:UniProtKB/Swiss-Prot;Acc:Q9C512] |
| AT1G51660 | -0,71 | 3,39E-30 | MKK4 | MKK4 [Source:UniProtKB/TrEMBL;Acc:A0A178WCC0] |
| AT1G51670 | 1,23 | 1,23E-09 | HTT5 | Protein HEAT-INDUCED TAS1 TARGET 5 [Source:UniProtKB/Swiss-Prot;Acc:Q8RY97] |
| AT1G51680 | -2,45 | 4,79E-110 | 4CL1 | 4-coumarate--CoA ligase 1 [Source:UniProtKB/Swiss-Prot;Acc:Q42524] |
| AT1G51760 | 0,98 | 1,69E-93 | ILL4 | JR3 [Source:UniProtKB/TrEMBL;Acc:A0A178W8I8] |
| AT1G51770 | -4,41 | 8,98E-10 | - | Core-2/I-branching beta-1,6-N-acetylglucosaminyltransferase family protein [Source:UniProtKB/TrEMBL;Acc:Q1G300] |
| AT1G51790 | -0,87 | 7,46E-06 | - | Leucine-rich repeat protein kinase family protein [Source:TAIR;Acc:AT1G51790] |
| AT1G51800 | -1,22 | 1,41E-24 | IOS1 | LRR receptor-like serine/threonine-protein kinase IOS1 [Source:UniProtKB/Swiss-Prot;Acc:Q9C8I6] |
| AT1G51830 | 3,71 | 1,04E-36 | - | Leucine-rich repeat protein kinase family protein [Source:TAIR;Acc:AT1G51830] |
| AT1G51840 | 3,73 | 1,84E-65 | - | protein kinase-related [Source:TAIR;Acc:AT1G51840] |
| AT1G51960 | 1,51 | 4,60E-02 | IQD27 | Calmodulin-binding family protein [Source:UniProtKB/TrEMBL;Acc:Q9ZU28] |
| AT1G52040 | -5,35 | 8,26E-06 | MBP1 | Myrosinase-binding protein 1 [Source:UniProtKB/Swiss-Prot;Acc:Q9SAV0] |
| AT1G52070 | 0,82 | 7,77E-77 | JAL10 | Jacalin-related lectin 10 [Source:UniProtKB/Swiss-Prot;Acc:Q8GWI7] |
| AT1G52080 | 0,83 | 2,78E-33 | AR791 | Actin binding protein family [Source:UniProtKB/TrEMBL;Acc:Q9SAU9] |
| AT1G52100 | 1,52 | 9,07E-03 | - | Mannose-binding lectin superfamily protein [Source:TAIR;Acc:AT1G52100] |
| AT1G52155 | 0,76 | 1,31E-04 | - | unknown protein; Ha. [Source:TAIR;Acc:AT1G52155] |
| AT1G52190 | -1,56 | 5,46E-34 | NPF1.2 | Protein NRT1/ PTR FAMILY 1.2 [Source:UniProtKB/Swiss-Prot;Acc:Q9M817] |
| AT1G52200 | -1,05 | 1,05E-02 | PCR8 | Protein PLANT CADMIUM RESISTANCE 8 [Source:UniProtKB/Swiss-Prot;Acc:Q9M815] |
| AT1G52230 | -2,62 | 6,07E-03 | PSAH2 | Photosystem I reaction center subunit VI-2, chloroplastic [Source:UniProtKB/Swiss-Prot;Acc:Q9SUI6] |
| AT1G52240 | -8,04 | 2,67E-13 | ROPGEF11 | ROPGEF11 [Source:UniProtKB/TrEMBL;Acc:A0A178W8V0] |
| AT1G52280 | -2,46 | 6,39E-61 | RABG3D | Ras-related protein RABG3d [Source:UniProtKB/Swiss-Prot;Acc:Q9C820] |
| AT1G52290 | -1,43 | 7,12E-03 | PERK15 | Proline-rich receptor-like protein kinase PERK15 [Source:UniProtKB/Swiss-Prot;Acc:Q9C821] |
| AT1G52300 | -0,62 | 8,89E-78 | RPL37B | 60S ribosomal protein L37-2 [Source:UniProtKB/Swiss-Prot;Acc:Q43292] |
| AT1G52310 | 0,59 | 2,58E-28 | - | C-type lectin receptor-like tyrosine-protein kinase At1g52310 [Source:UniProtKB/Swiss-Prot;Acc:Q9C823] |
| AT1G52342 | 7,62 | 3,77E-12 | - | Putative uncharacterized protein [Source:UniProtKB/TrEMBL;Acc:Q1G3Y9] |
| AT1G52360 | -0,93 | 4,36E-81 | - | Coatomer subunit beta' [Source:UniProtKB/TrEMBL;Acc:F4ICX0] |
| AT1G52510 | 0,71 | 2,36E-07 | - | Alpha/beta-Hydrolases superfamily protein [Source:UniProtKB/TrEMBL;Acc:Q8VZ57] |
| AT1G52530 | -1,18 | 1,03E-05 | - | Hus1 protein [Source:UniProtKB/TrEMBL;Acc:Q709F6] |
| AT1G52565 | -0,87 | 2,92E-03 | - | At1g52565 [Source:UniProtKB/TrEMBL;Acc:A0JPU2] |
| AT1G52570 | 1,25 | 5,97E-08 | PLDALPHA2 | Phospholipase D alpha 2 [Source:UniProtKB/Swiss-Prot;Acc:Q9SSQ9] |
| AT1G52590 | 1,01 | 1,35E-05 | - | DCC family protein At1g52590, chloroplastic [Source:UniProtKB/Swiss-Prot;Acc:Q9SSR1] |
| AT1G52600 | -0,72 | 7,57E-44 | - | Signal peptidase I [Source:UniProtKB/TrEMBL;Acc:Q9SSR2] |
| AT1G52615 | -3,18 | 2,87E-02 | - | unknown gene [Source:TAIR;Acc:AT1G52615] |
| AT1G52660 | -7,44 | 3,97E-11 | - | P-loop containing nucleoside triphosphate hydrolases superfamily protein [Source:TAIR;Acc:AT1G52660] |
| AT1G52670 | -0,68 | 1,91E-51 | - | Is a member of the PF [Source:UniProtKB/TrEMBL;Acc:Q8VZ68] |
| AT1G52700 | -4,53 | 1,15E-08 | - | Alpha/beta-Hydrolases superfamily protein [Source:UniProtKB/TrEMBL;Acc:A0A1P8AQT5] |
| AT1G52820 | 1,40 | 1,80E-06 | - | 2-oxoglutarate (2OG) and Fe(II)-dependent oxygenase superfamily protein [Source:UniProtKB/TrEMBL;Acc:Q9C936] |
| AT1G52870 | 1,43 | 1,23E-10 | - | At1g52870/F14G24_14 [Source:UniProtKB/TrEMBL;Acc:Q9C933] |
| AT1G52880 | 1,35 | 1,82E-03 | NAC018 | NARS2 [Source:UniProtKB/TrEMBL;Acc:A0A178W6K4] |
| AT1G52920 | 0,82 | 1,04E-04 | GCR2 | LanC-like protein GCR2 [Source:UniProtKB/Swiss-Prot;Acc:F4IEM5] |
| AT1G53000 | -0,70 | 5,62E-65 | KDSB | Nucleotide-diphospho-sugar transferases superfamily protein [Source:TAIR;Acc:AT1G53000] |
| AT1G53030 | 0,63 | 1,37E-11 | COX17-2 | Cytochrome c oxidase copper chaperone 2 [Source:UniProtKB/Swiss-Prot;Acc:Q94FT1] |
| AT1G53035 | -0,62 | 4,83E-06 | - | unknown protein; FUNCTIONS IN: molecular_function unknown; INVOLVED IN: biological_process unknown; LOCATED IN: endomembrane system; EXPRESSED IN: 23 plant structures; EXPRESSED DURING: 13 growth stages; BEST Arabidopsis thaliana protein match is: u /.../ protein (TAIR:AT3G15358.1); Ha. [Source:TAIR;Acc:AT1G53035] |
| AT1G53040 | -1,43 | 1,79E-81 | - | Putative tRNA (Met) cytidine acetyltransferase [Source:UniProtKB/TrEMBL;Acc:Q0WNW2] |
| AT1G53080 | -2,54 | 1,48E-03 | - | Lectin-like protein At1g53080 [Source:UniProtKB/Swiss-Prot;Acc:Q9LNN3] |
| AT1G53090 | 1,03 | 1,22E-15 | SPA4 | SPA4 [Source:UniProtKB/TrEMBL;Acc:A0A178WBA0] |
| AT1G53110 | 0,65 | 5,63E-08 | PPI4 | Proton pump-interactor 4 [Source:UniProtKB/Swiss-Prot;Acc:Q8VZN4] |
| AT1G53140 | -0,70 | 2,23E-17 | DRP5A | DRP5A [Source:UniProtKB/TrEMBL;Acc:A0A178WBH9] |
| AT1G53200 | 0,80 | 8,99E-33 | - | unknown protein; Ha. [Source:TAIR;Acc:AT1G53200] |
| AT1G53240 | -0,83 | 2,99E-113 | mMDH1 | Malate dehydrogenase 1, mitochondrial [Source:UniProtKB/Swiss-Prot;Acc:Q9ZP06] |
| AT1G53260 | 2,22 | 1,56E-02 | - | Putative uncharacterized protein [Source:UniProtKB/TrEMBL;Acc:Q58G44] |
| AT1G53270 | -0,81 | 4,93E-02 | ABCG10 | ABCG10 [Source:UniProtKB/TrEMBL;Acc:A0A178WBC4] |
| AT1G53290 | -0,93 | 5,07E-51 | B3GALT14 | Hexosyltransferase (Fragment) [Source:UniProtKB/TrEMBL;Acc:W8Q798] |
| AT1G53310 | -1,20 | 2,21E-218 | PPC1 | Phosphoenolpyruvate carboxylase 1 [Source:UniProtKB/Swiss-Prot;Acc:Q9MAH0] |
| AT1G53320 | 0,86 | 2,49E-86 | TULP7 | Tubby-like F-box protein [Source:UniProtKB/TrEMBL;Acc:A0A178WNG9] |
| AT1G53330 | 1,09 | 3,23E-11 | - | Putative pentatricopeptide repeat-containing protein At1g53330 [Source:UniProtKB/Swiss-Prot;Acc:Q9MAG8] |
| AT1G53340 | -2,42 | 1,67E-36 | - | Cysteine/Histidine-rich C1 domain family protein [Source:TAIR;Acc:AT1G53340] |
| AT1G53420 | 1,18 | 1,02E-04 | - | Probable LRR receptor-like serine/threonine-protein kinase At1g53420 [Source:UniProtKB/Swiss-Prot;Acc:C0LGG7] |
| AT1G53460 | 1,52 | 9,48E-126 | - | At1g53460/T3F20_21 [Source:UniProtKB/TrEMBL;Acc:Q9LPG2] |
| AT1G53500 | -1,20 | 6,56E-68 | RHM2 | RHM2 [Source:UniProtKB/TrEMBL;Acc:A0A178W591] |
| AT1G53540 | 3,27 | 4,63E-39 | HSP17.6C | 17.6 kDa class I heat shock protein 3 [Source:UniProtKB/Swiss-Prot;Acc:P13853] |
| AT1G53542 | -0,94 | 6,86E-03 | - | At1g53545 [Source:UniProtKB/TrEMBL;Acc:Q84WL5] |
| AT1G53560 | 1,51 | 5,50E-48 | - | At1g53560 [Source:UniProtKB/TrEMBL;Acc:Q9LPH1] |
| AT1G53600 | 1,01 | 5,91E-12 | PCMP-E63 | Pentatricopeptide repeat-containing protein At1g53600, mitochondrial [Source:UniProtKB/Swiss-Prot;Acc:Q9C8L6] |
| AT1G53620 | 3,07 | 9,43E-04 | - | Transmembrane protein [Source:UniProtKB/TrEMBL;Acc:Q9C8L8] |
| AT1G53633 | 1,63 | 1,14E-04 | - | unknown protein; FUNCTIONS IN: molecular_function unknown; INVOLVED IN: biological_process unknown; LOCATED IN: cellular_component unknown; Ha. [Source:TAIR;Acc:AT1G53633] |
| AT1G53660 | -2,30 | 4,98E-67 | - | nodulin MtN21 /EamA-like transporter family protein [Source:TAIR;Acc:AT1G53660] |
| AT1G53670 | 1,10 | 1,81E-20 | MSRB1 | Peptide methionine sulfoxide reductase B1, chloroplastic [Source:UniProtKB/Swiss-Prot;Acc:Q9C8M2] |
| AT1G53680 | -1,00 | 5,38E-08 | GSTU28 | Glutathione S-transferase U28 [Source:UniProtKB/Swiss-Prot;Acc:Q9C8M3] |
| AT1G53700 | -0,64 | 9,47E-15 | WAG1 | Serine/threonine-protein kinase WAG1 [Source:UniProtKB/Swiss-Prot;Acc:Q9C8M5] |
| AT1G53780 | 0,97 | 7,73E-70 | - | 26S proteasome regulatory complex ATPase [Source:UniProtKB/TrEMBL;Acc:F4HTC3] |
| AT1G53790 | 1,12 | 1,56E-23 | - | F-box and associated interaction domains-containing protein [Source:UniProtKB/TrEMBL;Acc:B3H7F2] |
| AT1G53830 | -1,39 | 2,98E-11 | PME2 | Pectinesterase 2 [Source:UniProtKB/Swiss-Prot;Acc:Q42534] |
| AT1G53840 | -0,60 | 1,24E-50 | PME1 | Pectinesterase [Source:UniProtKB/TrEMBL;Acc:Q0WWC7] |
| AT1G53950 | 1,43 | 4,70E-02 | - | F15I1.4 protein [Source:UniProtKB/TrEMBL;Acc:Q9SYF2] |
| AT1G54000 | -1,70 | 5,90E-65 | GLL22 | GDSL esterase/lipase 22 [Source:UniProtKB/Swiss-Prot;Acc:Q1H583] |
| AT1G54010 | -0,76 | 6,33E-46 | GLL23 | Inactive GDSL esterase/lipase-like protein 23 [Source:UniProtKB/Swiss-Prot;Acc:Q8W4H8] |
| AT1G54020 | 1,61 | 4,89E-21 | - | GDSL esterase/lipase At1g54020 [Source:UniProtKB/Swiss-Prot;Acc:Q9C5N8] |
| AT1G54050 | 1,75 | 1,02E-56 | HSP17.4B | 17.4 kDa class III heat shock protein [Source:UniProtKB/Swiss-Prot;Acc:Q9SYG1] |
| AT1G54095 | -1,44 | 5,78E-07 | - | At1g54095 [Source:UniProtKB/TrEMBL;Acc:Q6NNH0] |
| AT1G54200 | 0,62 | 2,80E-02 | - | Protein BIG GRAIN 1-like B [Source:UniProtKB/Swiss-Prot;Acc:Q9SLL2] |
| AT1G54210 | 0,93 | 1,55E-09 | ATG12A | Ubiquitin-like protein ATG12 [Source:UniProtKB/TrEMBL;Acc:A0A178VZU7] |
| AT1G54220 | -0,89 | 8,32E-55 | - | Acetyltransferase component of pyruvate dehydrogenase complex [Source:UniProtKB/TrEMBL;Acc:A0A178W5Y1] |
| AT1G54280 | 2,82 | 4,46E-10 | ALA6 | Phospholipid-transporting ATPase 6 [Source:UniProtKB/Swiss-Prot;Acc:Q9SLK6] |
| AT1G54290 | 0,68 | 2,93E-06 | - | Protein translation factor SUI1 homolog 2 [Source:UniProtKB/Swiss-Prot;Acc:Q94JV4] |
| AT1G54310 | 0,72 | 2,98E-21 | - | S-adenosyl-L-methionine-dependent methyltransferases superfamily protein [Source:UniProtKB/TrEMBL;Acc:F4HVA0] |
| AT1G54340 | 0,73 | 2,70E-35 | ICDH | Peroxisomal isocitrate dehydrogenase [NADP] [Source:UniProtKB/Swiss-Prot;Acc:Q9SLK0] |
| AT1G54380 | 0,66 | 8,11E-31 | - | Spliceosome protein-like protein [Source:UniProtKB/TrEMBL;Acc:Q9C5M7] |
| AT1G54410 | -1,46 | 6,46E-172 | HIRD11 | HIRD11 [Source:UniProtKB/TrEMBL;Acc:A0A178WR56] |
| AT1G54450 | -0,59 | 6,93E-18 | B''DELTA | Probable serine/threonine protein phosphatase 2A regulatory subunit B''delta [Source:UniProtKB/Swiss-Prot;Acc:Q9SLI8] |
| AT1G54500 | -0,93 | 4,07E-06 | - | At1g54500/F20D21_31 [Source:UniProtKB/TrEMBL;Acc:Q9SLI4] |
| AT1G54530 | -1,54 | 1,14E-17 | - | Calcium-binding EF hand family protein [Source:TAIR;Acc:AT1G54530] |
| AT1G54570 | 0,97 | 1,38E-53 | - | Acyltransferase-like protein At1g54570, chloroplastic [Source:UniProtKB/Swiss-Prot;Acc:Q9ZVN2] |
| AT1G54575 | 3,60 | 2,03E-20 | - | unknown protein; Ha. [Source:TAIR;Acc:AT1G54575] |
| AT1G54580 | -0,61 | 2,70E-69 | ACP2 | Acyl carrier protein 2, chloroplastic [Source:UniProtKB/Swiss-Prot;Acc:P25701] |
| AT1G54630 | -0,83 | 3,04E-111 | ACP3 | Acyl carrier protein 3, chloroplastic [Source:UniProtKB/Swiss-Prot;Acc:P25702] |
| AT1G54680 | 1,38 | 5,57E-09 | - | T22H22.11 protein [Source:UniProtKB/TrEMBL;Acc:Q9ZVM3] |
| AT1G54740 | 1,00 | 1,35E-06 | - | Protein of unknown function (DUF3049) [Source:TAIR;Acc:AT1G54740] |
| AT1G54773 | 1,84 | 3,98E-45 | - | - |
| AT1G54780 | -2,87 | 4,99E-12 | TLP18.3 | TLP18.3 [Source:UniProtKB/TrEMBL;Acc:A0A178WHT4] |
| AT1G54820 | -4,06 | 6,11E-07 | - | Protein kinase superfamily protein [Source:UniProtKB/TrEMBL;Acc:F4HYK7] |
| AT1G54840 | 0,79 | 2,39E-04 | IDM2 | Increased DNA methylation 2 [Source:UniProtKB/Swiss-Prot;Acc:Q8RWL4] |
| AT1G54870 | -2,01 | 8,98E-06 | - | NAD(P)-binding Rossmann-fold superfamily protein [Source:TAIR;Acc:AT1G54870] |
| AT1G54890 | -0,81 | 1,44E-39 | - | Late embryogenesis abundant (LEA) protein-like protein [Source:UniProtKB/TrEMBL;Acc:Q9FZ41] |
| AT1G54940 | -4,09 | 2,96E-02 | GUX4 | Putative UDP-glucuronate:xylan alpha-glucuronosyltransferase 4 [Source:UniProtKB/Swiss-Prot;Acc:Q9FZ37] |
| AT1G54970 | -5,08 | 5,78E-04 | PRP1 | Proline-rich protein 1 [Source:UniProtKB/Swiss-Prot;Acc:Q9FZ35] |
| AT1G55000 | 1,13 | 5,56E-21 | - | F-box protein At1g55000 [Source:UniProtKB/Swiss-Prot;Acc:Q9FZ32] |
| AT1G55020 | 0,74 | 1,26E-16 | LOX1 | Linoleate 9S-lipoxygenase 1 [Source:UniProtKB/Swiss-Prot;Acc:Q06327] |
| AT1G55030 | 0,89 | 2,54E-03 | - | RNI-like superfamily protein [Source:TAIR;Acc:AT1G55030] |
| AT1G55090 | 0,67 | 5,42E-54 | - | Glutamine-dependent NAD(+) synthetase [Source:UniProtKB/Swiss-Prot;Acc:Q9C723] |
| AT1G55120 | -4,12 | 9,38E-42 | CWINV3 | Beta-fructofuranosidase, insoluble isoenzyme CWINV3 [Source:UniProtKB/Swiss-Prot;Acc:Q67XZ3] |
| AT1G55205 | -0,66 | 5,84E-19 | - | unknown protein; FUNCTIONS IN: molecular_function unknown; INVOLVED IN: biological_process unknown; LOCATED IN: chloroplast; BEST Arabidopsis thaliana protein match is: unknown protein (TAIR:AT3G13674.1); Ha. [Source:TAIR;Acc:AT1G55205] |
| AT1G55230 | 3,26 | 1,88E-02 | - | Proteinase inhibitor I4, serpin (DUF716) [Source:UniProtKB/TrEMBL;Acc:Q9C893] |
| AT1G55290 | -2,64 | 2,07E-02 | F6'H2 | Feruloyl CoA ortho-hydroxylase 2 [Source:UniProtKB/Swiss-Prot;Acc:Q9C899] |
| AT1G55310 | 1,18 | 3,40E-182 | SR33 | SC35-like splicing factor 33 [Source:TAIR;Acc:AT1G55310] |
| AT1G55320 | 1,14 | 5,40E-52 | AAE18 | AAE18 [Source:UniProtKB/TrEMBL;Acc:A0A178WMT3] |
| AT1G55380 | -1,01 | 7,00E-22 | - | Cysteine/Histidine-rich C1 domain family protein [Source:UniProtKB/TrEMBL;Acc:Q9C6G7] |
| AT1G55430 | -1,51 | 1,88E-29 | - | Cysteine/Histidine-rich C1 domain family protein [Source:UniProtKB/TrEMBL;Acc:Q4TU44] |
| AT1G55450 | -1,54 | 1,08E-10 | - | S-adenosyl-L-methionine-dependent methyltransferases superfamily protein [Source:UniProtKB/TrEMBL;Acc:F4I0B5] |
| AT1G55510 | 1,50 | 3,40E-47 | BCDH BETA1 | 2-oxoisovalerate dehydrogenase subunit beta 1, mitochondrial [Source:UniProtKB/Swiss-Prot;Acc:Q9SAV3] |
| AT1G55530 | 1,29 | 2,75E-47 | - | At1g55530/T5A14_7 [Source:UniProtKB/TrEMBL;Acc:Q9ZVU8] |
| AT1G55591 | 3,61 | 2,34E-06 | MIR158B | MIR158b; miRNA [Source:TAIR;Acc:AT1G55591] |
| AT1G55600 | 1,53 | 9,75E-04 | WRKY10 | Probable WRKY transcription factor 10 [Source:UniProtKB/Swiss-Prot;Acc:Q9LG05] |
| AT1G55610 | 0,73 | 3,10E-03 | BRL1 | Serine/threonine-protein kinase BRI1-like 1 [Source:UniProtKB/Swiss-Prot;Acc:Q9ZWC8] |
| AT1G55670 | -4,23 | 2,19E-02 | PSAG | Photosystem I reaction center subunit V, chloroplastic [Source:UniProtKB/Swiss-Prot;Acc:Q9S7N7] |
| AT1G55740 | 1,39 | 1,20E-12 | RFS1 | Probable galactinol--sucrose galactosyltransferase 1 [Source:UniProtKB/Swiss-Prot;Acc:Q84VX0] |
| AT1G55850 | 0,86 | 1,16E-103 | CSLE1 | Cellulose synthase-like protein E1 [Source:UniProtKB/Swiss-Prot;Acc:Q8VZK9] |
| AT1G55880 | 0,69 | 2,65E-22 | - | At1g55880 [Source:UniProtKB/TrEMBL;Acc:Q6NKY5] |
| AT1G55910 | 0,71 | 1,18E-03 | ZIP11 | Zinc transporter 11 [Source:UniProtKB/Swiss-Prot;Acc:Q94EG9] |
| AT1G55980 | 0,75 | 3,56E-02 | - | FAD/NAD(P)-binding oxidoreductase family protein [Source:TAIR;Acc:AT1G55980] |
| AT1G56010 | 0,97 | 4,77E-07 | NAC021 | NAC domain-containing protein 21/22 [Source:UniProtKB/Swiss-Prot;Acc:Q84TE6] |
| AT1G56045 | -0,88 | 6,38E-31 | RPL41G | 60S ribosomal protein L41 [Source:UniProtKB/Swiss-Prot;Acc:P62120] |
| AT1G56070 | -0,58 | 2,10E-86 | LOS1 | Elongation factor 2 [Source:UniProtKB/Swiss-Prot;Acc:Q9ASR1] |
| AT1G56120 | 0,70 | 4,49E-08 | - | Leucine-rich repeat transmembrane protein kinase [Source:UniProtKB/TrEMBL;Acc:F4I3K0] |
| AT1G56150 | 5,84 | 2,53E-07 | SAUR71 | Auxin-responsive protein SAUR71 [Source:UniProtKB/Swiss-Prot;Acc:Q9SGU2] |
| AT1G56165 | 4,33 | 6,56E-24 | - | other RNA [Source:TAIR;Acc:AT1G56165] |
| AT1G56200 | 1,98 | 3,87E-211 | emb1303 | EMB1303 [Source:UniProtKB/TrEMBL;Acc:A0A178WKB3] |
| AT1G56210 | 0,73 | 1,60E-11 | HIPP35 | Heavy metal-associated isoprenylated plant protein 35 [Source:UniProtKB/Swiss-Prot;Acc:Q9C7J6] |
| AT1G56280 | 1,79 | 0,00E+00 | ATDI19 | drought-induced 19 [Source:TAIR;Acc:AT1G56280] |
| AT1G56300 | 3,56 | 0,00E+00 | - | At1g56300 [Source:UniProtKB/TrEMBL;Acc:Q8L7R1] |
| AT1G56320 | -1,24 | 1,83E-02 | - | At1g56320 [Source:UniProtKB/TrEMBL;Acc:Q9C7K7] |
| AT1G56330 | -1,30 | 1,84E-225 | SAR1B | SAR1B [Source:UniProtKB/TrEMBL;Acc:A0A178WM97] |
| AT1G56423 | -0,64 | 1,82E-22 | - | unknown protein; FUNCTIONS IN: molecular_function unknown; INVOLVED IN: biological_process unknown; LOCATED IN: endomembrane system; Ha. [Source:TAIR;Acc:AT1G56423] |
| AT1G56440 | 1,10 | 1,69E-39 | TPR5 | TPR5 [Source:UniProtKB/TrEMBL;Acc:A0A178WG02] |
| AT1G56450 | -0,68 | 7,06E-70 | PBG1 | Proteasome subunit beta type-4 [Source:UniProtKB/Swiss-Prot;Acc:Q7DLR9] |
| AT1G56500 | 0,59 | 3,29E-07 | SOQ1 | Protein SUPPRESSOR OF QUENCHING 1, chloroplastic [Source:UniProtKB/Swiss-Prot;Acc:Q8VZ10] |
| AT1G56540 | -2,88 | 4,37E-03 | - | Disease resistance protein (TIR-NBS-LRR class) family [Source:UniProtKB/TrEMBL;Acc:Q9FXA6] |
| AT1G56550 | -1,80 | 5,91E-49 | RGXT3 | UDP-D-xylose:L-fucose alpha-1,3-D-xylosyltransferase 3 [Source:UniProtKB/Swiss-Prot;Acc:Q9FXA7] |
| AT1G56610 | 0,68 | 1,86E-09 | - | F-box protein At1g56610 [Source:UniProtKB/Swiss-Prot;Acc:Q8GYP8] |
| AT1G56612 | 0,94 | 4,28E-15 | - | other RNA [Source:TAIR;Acc:AT1G56612] |
| AT1G56630 | -1,65 | 1,29E-15 | - | alpha/beta-Hydrolases superfamily protein [Source:TAIR;Acc:AT1G56630] |
| AT1G56660 | -1,99 | 4,63E-40 | - | F25P12.91 protein [Source:UniProtKB/TrEMBL;Acc:Q9FXB5] |
| AT1G56670 | -0,87 | 1,25E-18 | LIP4 | GDSL esterase/lipase LIP-4 [Source:UniProtKB/Swiss-Prot;Acc:Q9FXB6] |
| AT1G56680 | -0,76 | 6,97E-26 | - | Chitinase family protein [Source:TAIR;Acc:AT1G56680] |
| AT1G57560 | -2,26 | 2,31E-19 | AtMYB50 | At1g57560 [Source:UniProtKB/TrEMBL;Acc:Q9C695] |
| AT1G57570 | -1,27 | 9,86E-05 | JAL14 | Jacalin-related lectin 14 [Source:UniProtKB/Swiss-Prot;Acc:F4I837] |
| AT1G57590 | -3,25 | 4,12E-20 | PAE2 | Pectin acetylesterase 2 [Source:UniProtKB/Swiss-Prot;Acc:F4I839] |
| AT1G57600 | -0,72 | 1,68E-30 | - | MBOAT (Membrane bound O-acyl transferase) family protein [Source:UniProtKB/TrEMBL;Acc:Q8RY80] |
| AT1G57610 | -0,66 | 1,77E-34 | - | Calcium uniporter protein 5, mitochondrial [Source:UniProtKB/Swiss-Prot;Acc:Q8VYR0] |
| AT1G57620 | -1,22 | 2,89E-125 | CYB | Transmembrane emp24 domain-containing protein p24delta4 [Source:UniProtKB/Swiss-Prot;Acc:Q9FVU0] |
| AT1G57670 | 2,86 | 2,35E-02 | - | Disease resistance protein RPS4, putative [Source:UniProtKB/TrEMBL;Acc:Q9FVT6] |
| AT1G57770 | 1,81 | 6,62E-11 | - | FAD/NAD(P)-binding oxidoreductase family protein [Source:UniProtKB/TrEMBL;Acc:Q9FVR9] |
| AT1G57790 | 1,55 | 5,21E-05 | - | F-box/kelch-repeat protein At1g57790 [Source:UniProtKB/Swiss-Prot;Acc:Q9FVS1] |
| AT1G57820 | 0,80 | 3,80E-58 | ORTH2 | E3 ubiquitin-protein ligase ORTHRUS 2 [Source:UniProtKB/Swiss-Prot;Acc:Q8VYZ0] |
| AT1G57860 | -0,76 | 5,76E-117 | RPL21E | 60S ribosomal protein L21-2 [Source:UniProtKB/Swiss-Prot;Acc:Q9FDZ9] |
| AT1G58030 | 0,93 | 8,41E-43 | CAT2 | Cationic amino acid transporter 2, vacuolar [Source:UniProtKB/Swiss-Prot;Acc:Q9ASS7] |
| AT1G58037 | -1,38 | 2,57E-16 | - | Cysteine/Histidine-rich C1 domain family protein [Source:UniProtKB/TrEMBL;Acc:Q2V4G4] |
| AT1G58080 | -1,09 | 2,02E-102 | HISN1A | HISN1A [Source:UniProtKB/TrEMBL;Acc:A0A178WM39] |
| AT1G58120 | 4,51 | 2,70E-03 | - | Uncharacterized protein At1g58120/68103_m00121 [Source:UniProtKB/TrEMBL;Acc:Q9C6F6] |
| AT1G58150 | 1,72 | 1,72E-26 | - | Phosphoglycerate kinase, putative [Source:UniProtKB/TrEMBL;Acc:Q9C6R4] |
| AT1G58170 | -1,30 | 1,87E-15 | DIR19 | Dirigent protein 19 [Source:UniProtKB/Swiss-Prot;Acc:Q9C523] |
| AT1G58180 | 0,80 | 4,68E-10 | BCA6 | Beta carbonic anhydrase 6, mitochondrial [Source:UniProtKB/Swiss-Prot;Acc:Q9C6F5] |
| AT1G58215 | 1,84 | 5,52E-13 | NET2A | Protein NETWORKED 2A [Source:UniProtKB/Swiss-Prot;Acc:P0DMS1] |
| AT1G58220 | 0,64 | 7,31E-12 | - | At1g58220 [Source:UniProtKB/TrEMBL;Acc:Q8GZ96] |
| AT1G58270 | -4,98 | 4,33E-177 | ZW9 | At1g58270/F19C14_8 [Source:UniProtKB/TrEMBL;Acc:Q9SLV3] |
| AT1G58340 | 2,12 | 4,10E-22 | DTX48 | Protein DETOXIFICATION 48 [Source:UniProtKB/Swiss-Prot;Acc:Q9SLV0] |
| AT1G58400 | 0,97 | 3,12E-38 | - | Putative disease resistance protein At1g58400 [Source:UniProtKB/Swiss-Prot;Acc:Q8W3K3] |
| AT1G58410 | 1,14 | 1,43E-24 | RXW24L | Probable disease resistance protein RXW24L [Source:UniProtKB/Swiss-Prot;Acc:Q9C646] |
| AT1G58470 | 1,05 | 8,84E-14 | RBP1 | RNA-binding protein 1 [Source:UniProtKB/Swiss-Prot;Acc:Q9C652] |
| AT1G58520 | -1,45 | 8,29E-29 | RXW8 | CSC1-like protein RXW8 [Source:UniProtKB/Swiss-Prot;Acc:F4IBD7] |
| AT1G58983 | -0,58 | 9,60E-03 | RPS2B | 40S ribosomal protein S2-2 [Source:UniProtKB/Swiss-Prot;Acc:Q93VB8] |
| AT1G59359 | -1,11 | 1,16E-08 | RPS2B | 40S ribosomal protein S2-2 [Source:UniProtKB/Swiss-Prot;Acc:Q93VB8] |
| AT1G59530 | 2,09 | 5,66E-14 | BZIP4 | Basic leucine zipper 4 [Source:UniProtKB/Swiss-Prot;Acc:Q9LQ65] |
| AT1G59590 | 1,42 | 1,56E-20 | ZCF37 | At1g59590 [Source:UniProtKB/TrEMBL;Acc:Q9SLT9] |
| AT1G59600 | -0,78 | 8,52E-39 | ZCW7 | ZCW7 protein [Source:UniProtKB/TrEMBL;Acc:Q9SLT8] |
| AT1G59700 | 1,06 | 1,44E-23 | GSTU16 | Glutathione S-transferase U16 [Source:UniProtKB/Swiss-Prot;Acc:Q9XIF8] |
| AT1G59740 | 1,80 | 2,10E-07 | NPF4.3 | Protein NRT1/ PTR FAMILY 4.3 [Source:UniProtKB/Swiss-Prot;Acc:Q93VV5] |
| AT1G59860 | 3,43 | 6,57E-105 | HSP17.6A | 17.6 kDa class I heat shock protein 1 [Source:UniProtKB/Swiss-Prot;Acc:Q9XIE3] |
| AT1G59865 | 3,66 | 6,67E-52 | - | unknown protein; Ha. [Source:TAIR;Acc:AT1G59865] |
| AT1G59940 | -0,73 | 2,54E-23 | ARR3 | response regulator 3 [Source:TAIR;Acc:AT1G59940] |
| AT1G59970 | -0,98 | 1,13E-17 | 5MMP | Metalloendoproteinase 5-MMP [Source:UniProtKB/Swiss-Prot;Acc:Q9ZUJ5] |
| AT1G59980 | 0,80 | 1,66E-27 | ATJ39 | Chaperone protein dnaJ 39 [Source:UniProtKB/Swiss-Prot;Acc:Q6XL73] |
| AT1G60000 | 0,95 | 1,56E-05 | - | AT1G60000 protein [Source:UniProtKB/TrEMBL;Acc:Q9ZUJ3] |
| AT1G60030 | 0,91 | 1,30E-04 | NAT7 | Nucleobase-ascorbate transporter 7 [Source:UniProtKB/Swiss-Prot;Acc:Q0WPE9] |
| AT1G60110 | 1,33 | 4,88E-02 | - | Mannose-binding lectin superfamily protein [Source:TAIR;Acc:AT1G60110] |
| AT1G60130 | -0,63 | 1,29E-02 | JAL18 | Jacalin-related lectin 18 [Source:UniProtKB/Swiss-Prot;Acc:O80737] |
| AT1G60160 | -0,65 | 1,67E-05 | POT12 | Putative potassium transporter 12 [Source:UniProtKB/Swiss-Prot;Acc:O80739] |
| AT1G60190 | -2,49 | 2,29E-19 | PUB19 | U-box domain-containing protein 19 [Source:UniProtKB/Swiss-Prot;Acc:O80742] |
| AT1G60270 | 1,31 | 1,03E-03 | BGLU6 | Putative beta-glucosidase 6 [Source:UniProtKB/Swiss-Prot;Acc:Q682B4] |
| AT1G60505 | 0,75 | 2,10E-19 | - | other RNA [Source:TAIR;Acc:AT1G60505] |
| AT1G60525 | 0,69 | 1,33E-04 | - | other RNA [Source:TAIR;Acc:AT1G60525] |
| AT1G60610 | 1,96 | 1,22E-134 | - | At1g60610 [Source:UniProtKB/TrEMBL;Acc:Q4TU35] |
| AT1G60730 | 0,97 | 1,32E-56 | - | NAD(P)-linked oxidoreductase superfamily protein [Source:TAIR;Acc:AT1G60730] |
| AT1G60740 | 1,25 | 7,42E-17 | PRXIID | At1g60740 [Source:UniProtKB/TrEMBL;Acc:B4G289] |
| AT1G60750 | 3,15 | 6,25E-60 | - | Probable aldo-keto reductase 6 [Source:UniProtKB/Swiss-Prot;Acc:F4HPY8] |
| AT1G60780 | -0,84 | 2,98E-62 | AP1M2 | AP-1 complex subunit mu-2 [Source:UniProtKB/Swiss-Prot;Acc:O22715] |
| AT1G60787 | -3,14 | 8,08E-41 | - | Cysteine/histidine-rich C1 domain protein [Source:UniProtKB/TrEMBL;Acc:A0A1P8ATJ8] |
| AT1G60940 | 1,19 | 7,18E-46 | SRK2B | Serine/threonine-protein kinase SRK2B [Source:UniProtKB/Swiss-Prot;Acc:Q9C958] |
| AT1G60960 | 0,71 | 2,53E-17 | IRT3 | IRT3 [Source:UniProtKB/TrEMBL;Acc:A0A178WHL0] |
| AT1G60970 | 3,65 | 2,55E-24 | - | Coatomer subunit zeta-1 [Source:UniProtKB/Swiss-Prot;Acc:Q940S5] |
| AT1G60980 | 1,90 | 9,88E-03 | GA20OX4 | Gibberellin 20 oxidase 4 [Source:UniProtKB/Swiss-Prot;Acc:Q9C955] |
| AT1G61065 | 1,21 | 7,72E-11 | - | 1,3-beta-glucan synthase component (DUF1218) [Source:UniProtKB/TrEMBL;Acc:Q8W576] |
| AT1G61100 | -0,63 | 6,41E-35 | - | CSL1 [Source:UniProtKB/TrEMBL;Acc:F4HRR0] |
| AT1G61140 | 0,58 | 5,64E-25 | EDA16 | SNF2 domain-containing protein / helicase domain-containing protein / zinc finger protein-like protein [Source:UniProtKB/TrEMBL;Acc:F4HTG1] |
| AT1G61170 | -1,57 | 4,75E-45 | - | At1g61170 [Source:UniProtKB/TrEMBL;Acc:O22729] |
| AT1G61180 | 0,98 | 1,03E-36 | - | LRR and NB-ARC domains-containing disease resistance protein [Source:UniProtKB/TrEMBL;Acc:Q2V4G0] |
| AT1G61190 | 0,88 | 8,60E-07 | - | Probable disease resistance protein At1g61190 [Source:UniProtKB/Swiss-Prot;Acc:O22727] |
| AT1G61215 | 1,94 | 1,55E-19 | BRD4 | At1g61215 [Source:UniProtKB/TrEMBL;Acc:Q6AWX2] |
| AT1G61230 | -2,84 | 7,47E-24 | - | Mannose-binding lectin superfamily protein [Source:UniProtKB/TrEMBL;Acc:F4HTI1] |
| AT1G61275 | 2,76 | 7,13E-28 | U12 | U12; snRNA [Source:TAIR;Acc:AT1G61275] |
| AT1G61300 | -1,73 | 2,04E-03 | - | Probable disease resistance protein At1g61300 [Source:UniProtKB/Swiss-Prot;Acc:O64790] |
| AT1G61360 | 0,69 | 1,14E-11 | - | Serine/threonine-protein kinase [Source:UniProtKB/TrEMBL;Acc:A0A178W9H1] |
| AT1G61390 | 0,62 | 3,01E-07 | - | G-type lectin S-receptor-like serine/threonine-protein kinase At1g61390 [Source:UniProtKB/Swiss-Prot;Acc:O64781] |
| AT1G61400 | 1,26 | 3,62E-56 | - | S-locus lectin protein kinase family protein [Source:TAIR;Acc:AT1G61400] |
| AT1G61410 | 1,08 | 7,27E-09 | - | At1g61410 [Source:UniProtKB/TrEMBL;Acc:Q058G5] |
| AT1G61415 | 2,73 | 1,27E-25 | - | unknown protein; FUNCTIONS IN: molecular_function unknown; INVOLVED IN: biological_process unknown; LOCATED IN: cellular_component unknown; BEST Arabidopsis thaliana protein match is: unknown protein (TAIR:AT1G61450.1); Ha. [Source:TAIR;Acc:AT1G61415] |
| AT1G61420 | 0,62 | 4,29E-05 | - | S-locus lectin protein kinase family protein [Source:TAIR;Acc:AT1G61420] |
| AT1G61440 | 2,40 | 7,34E-69 | - | S-locus lectin protein kinase family protein [Source:TAIR;Acc:AT1G61440] |
| AT1G61470 | 1,89 | 1,99E-05 | CAF1-5 | Probable CCR4-associated factor 1 homolog 5 [Source:UniProtKB/Swiss-Prot;Acc:O64773] |
| AT1G61500 | 0,69 | 4,54E-16 | - | S-locus lectin protein kinase family protein [Source:TAIR;Acc:AT1G61500] |
| AT1G61550 | 0,98 | 4,30E-02 | - | G-type lectin S-receptor-like serine/threonine-protein kinase At1g61550 [Source:UniProtKB/Swiss-Prot;Acc:Q9SY95] |
| AT1G61560 | 3,18 | 3,03E-14 | MLO6 | MLO-like protein 6 [Source:UniProtKB/Swiss-Prot;Acc:Q94KB7] |
| AT1G61580 | -0,60 | 1,84E-35 | ARP2 | 60S ribosomal protein L3-2 [Source:UniProtKB/Swiss-Prot;Acc:P22738] |
| AT1G61620 | 0,89 | 9,35E-63 | CSU1 | Nitric oxide synthase-interacting protein homolog [Source:UniProtKB/TrEMBL;Acc:A0A178WN49] |
| AT1G61740 | -0,60 | 2,35E-12 | - | Sulfite exporter TauE/SafE family protein 2 [Source:UniProtKB/Swiss-Prot;Acc:Q9SYB0] |
| AT1G61770 | -1,00 | 3,74E-91 | C50 | Chaperone protein dnaJ 50 [Source:UniProtKB/Swiss-Prot;Acc:Q8GUN6] |
| AT1G61800 | 4,53 | 7,80E-209 | GPT2 | glucose-6-phosphate/phosphate translocator 2 [Source:TAIR;Acc:AT1G61800] |
| AT1G61840 | -2,72 | 3,41E-54 | - | Cysteine/Histidine-rich C1 domain family protein [Source:UniProtKB/TrEMBL;Acc:O80692] |
| AT1G61890 | -1,08 | 3,25E-84 | DTX37 | Protein DETOXIFICATION 37 [Source:UniProtKB/Swiss-Prot;Acc:O80695] |
| AT1G61920 | -3,00 | 1,27E-04 | - | Putative uncharacterized protein [Source:UniProtKB/TrEMBL;Acc:Q5XVH7] |
| AT1G61930 | -1,83 | 6,04E-10 | - | At1g61930 [Source:UniProtKB/TrEMBL;Acc:O80698] |
| AT1G61970 | 0,67 | 2,39E-13 | - | At1g61970 [Source:UniProtKB/TrEMBL;Acc:O80702] |
| AT1G62000 | 1,82 | 3,98E-02 | - | UPF0540 protein At1g62000 [Source:UniProtKB/Swiss-Prot;Acc:Q39168] |
| AT1G62020 | -0,76 | 1,24E-61 | - | Coatomer subunit alpha [Source:UniProtKB/TrEMBL;Acc:A0A178WAY4] |
| AT1G62180 | 2,25 | 2,09E-174 | 37347 | 5'-adenylylsulfate reductase 2, chloroplastic [Source:UniProtKB/Swiss-Prot;Acc:P92981] |
| AT1G62290 | 1,39 | 7,30E-07 | APA2 | Aspartic proteinase A2 [Source:UniProtKB/Swiss-Prot;Acc:Q8VYL3] |
| AT1G62320 | -1,16 | 2,12E-10 | - | CSC1-like protein At1g62320 [Source:UniProtKB/Swiss-Prot;Acc:F4HYR3] |
| AT1G62330 | -0,58 | 4,72E-42 | OFUT15 | O-fucosyltransferase 15 [Source:UniProtKB/Swiss-Prot;Acc:F4HYR4] |
| AT1G62370 | 1,72 | 8,98E-16 | - | F2401.10 [Source:UniProtKB/TrEMBL;Acc:O48801] |
| AT1G62422 | 1,29 | 1,72E-11 | - | F2401.15 [Source:UniProtKB/TrEMBL;Acc:Q9MAV1] |
| AT1G62440 | -0,95 | 2,86E-43 | LRX2 | Leucine-rich repeat extensin-like protein 2 [Source:UniProtKB/Swiss-Prot;Acc:O48809] |
| AT1G62450 | -2,84 | 1,96E-03 | - | Immunoglobulin E-set superfamily protein [Source:UniProtKB/TrEMBL;Acc:F4HYS9] |
| AT1G62480 | -1,20 | 8,26E-25 | - | At1g62480/T3P18_4 [Source:UniProtKB/TrEMBL;Acc:Q9SXE9] |
| AT1G62500 | 1,78 | 2,31E-73 | - | Bifunctional inhibitor/lipid-transfer protein/seed storage 2S albumin superfamily protein [Source:UniProtKB/TrEMBL;Acc:Q9SXE7] |
| AT1G62570 | 0,62 | 5,00E-43 | FMOGS-OX4 | Flavin-containing monooxygenase FMO GS-OX4 [Source:UniProtKB/Swiss-Prot;Acc:Q93Y23] |
| AT1G62580 | -0,88 | 3,49E-17 | - | Flavin-binding monooxygenase family protein [Source:TAIR;Acc:AT1G62580] |
| AT1G62590 | 0,65 | 1,24E-08 | - | Pentatricopeptide repeat-containing protein At1g62590 [Source:UniProtKB/Swiss-Prot;Acc:Q9SXD8] |
| AT1G62600 | -0,62 | 2,69E-15 | - | Flavin-containing monooxygenase FMO GS-OX-like 4 [Source:UniProtKB/Swiss-Prot;Acc:Q94BV5] |
| AT1G62640 | -1,00 | 9,48E-96 | KAS III | 3-oxoacyl-[acyl-carrier-protein] synthase III, chloroplastic [Source:UniProtKB/Swiss-Prot;Acc:P49243] |
| AT1G62660 | -1,79 | 3,07E-210 | BFRUCT3 | Acid beta-fructofuranosidase 3, vacuolar [Source:UniProtKB/Swiss-Prot;Acc:Q43348] |
| AT1G62720 | 1,82 | 3,81E-13 | - | Pentatricopeptide repeat-containing protein At1g62720 [Source:UniProtKB/Swiss-Prot;Acc:Q9SI78] |
| AT1G62760 | 1,53 | 2,79E-02 | PMEI10 | Pectinesterase inhibitor 10 [Source:UniProtKB/Swiss-Prot;Acc:Q9SI74] |
| AT1G62770 | -0,90 | 1,75E-06 | PMEI9 | Pectinesterase inhibitor 9 [Source:UniProtKB/Swiss-Prot;Acc:Q9SI72] |
| AT1G62790 | -0,68 | 2,03E-48 | - | At1g62790 [Source:UniProtKB/TrEMBL;Acc:Q6NLF7] |
| AT1G62810 | -1,53 | 1,24E-159 | - | Primary amine oxidase [Source:UniProtKB/Swiss-Prot;Acc:Q8H1H9] |
| AT1G62900 | 2,63 | 5,38E-03 | - | F16P17.4 protein [Source:UniProtKB/TrEMBL;Acc:Q9LQ17] |
| AT1G62935 | -0,80 | 2,27E-03 | - | unknown protein; FUNCTIONS IN: molecular_function unknown; INVOLVED IN: biological_process unknown; LOCATED IN: endomembrane system; Ha. [Source:TAIR;Acc:AT1G62935] |
| AT1G62940 | -0,91 | 7,08E-03 | 4CLL1 | 4-coumarate--CoA ligase-like 1 [Source:UniProtKB/Swiss-Prot;Acc:Q9LQ12] |
| AT1G62975 | -2,14 | 2,53E-05 | BHLH125 | Transcription factor bHLH125 [Source:UniProtKB/Swiss-Prot;Acc:Q9LQ08] |
| AT1G62980 | -5,38 | 1,57E-04 | EXPA18 | Expansin-A18 [Source:UniProtKB/Swiss-Prot;Acc:Q9LQ07] |
| AT1G62990 | -0,67 | 4,88E-02 | KNAT7 | Homeobox protein knotted-1-like 7 [Source:UniProtKB/Swiss-Prot;Acc:Q9FPQ8] |
| AT1G63000 | -1,75 | 6,74E-172 | NRS/ER | Bifunctional dTDP-4-dehydrorhamnose 3,5-epimerase/dTDP-4-dehydrorhamnose reductase [Source:UniProtKB/Swiss-Prot;Acc:Q9LQ04] |
| AT1G63010 | -0,59 | 4,14E-52 | - | Major Facilitator Superfamily with SPX (SYG1/Pho81/XPR1) domain-containing protein [Source:UniProtKB/TrEMBL;Acc:F4I0G7] |
| AT1G63020 | 0,92 | 2,44E-25 | NRPD1 | DNA-directed RNA polymerase IV subunit 1 [Source:UniProtKB/Swiss-Prot;Acc:Q9LQ02] |
| AT1G63050 | -1,08 | 1,58E-73 | LPLAT2 | LPLAT2 [Source:UniProtKB/TrEMBL;Acc:A0A178WEX3] |
| AT1G63080 | 0,70 | 2,00E-08 | - | Pentatricopeptide repeat-containing protein At1g63080, mitochondrial [Source:UniProtKB/Swiss-Prot;Acc:Q9CAN5] |
| AT1G63100 | -1,07 | 7,97E-40 | SCL28 | Scarecrow-like protein 28 [Source:UniProtKB/Swiss-Prot;Acc:Q9CAN3] |
| AT1G63120 | 1,14 | 8,91E-10 | RBL2 | RHOMBOID-like protein 2 [Source:UniProtKB/Swiss-Prot;Acc:Q9CAN1] |
| AT1G63150 | 1,05 | 2,79E-27 | - | Pentatricopeptide repeat-containing protein At1g63150 [Source:UniProtKB/Swiss-Prot;Acc:Q9CAM8] |
| AT1G63220 | -2,04 | 3,08E-46 | - | C2 domain-containing protein At1g63220 [Source:UniProtKB/Swiss-Prot;Acc:Q9C8S6] |
| AT1G63240 | 2,24 | 5,05E-56 | - | Uncharacterized protein At1g63240 [Source:UniProtKB/TrEMBL;Acc:Q949Y1] |
| AT1G63250 | 0,63 | 4,60E-44 | RH48 | Probable DEAD-box ATP-dependent RNA helicase 48 [Source:UniProtKB/Swiss-Prot;Acc:Q9C8S9] |
| AT1G63310 | 0,74 | 3,72E-05 | - | Uncharacterized protein At1g63310/F9N12_7 [Source:UniProtKB/TrEMBL;Acc:Q9C8T5] |
| AT1G63440 | 1,20 | 9,19E-32 | HMA5 | Probable copper-transporting ATPase HMA5 [Source:UniProtKB/Swiss-Prot;Acc:Q9SH30] |
| AT1G63450 | -3,77 | 3,58E-42 | GT16 | Xyloglucan-specific galacturonosyltransferase 1 [Source:UniProtKB/Swiss-Prot;Acc:Q9SH31] |
| AT1G63470 | -0,67 | 2,66E-32 | AHL5 | AT-hook motif nuclear-localized protein 5 [Source:UniProtKB/Swiss-Prot;Acc:Q8GXB3] |
| AT1G63530 | 1,02 | 4,96E-02 | - | BEST Arabidopsis thaliana protein match is: hydroxyproline-rich glycoprotein family protein (TAIR:AT1G63540.1); Ha. [Source:TAIR;Acc:AT1G63530] |
| AT1G63600 | -4,72 | 2,17E-03 | - | Receptor-like protein kinase-related family protein [Source:TAIR;Acc:AT1G63600] |
| AT1G63650 | -1,40 | 8,34E-65 | BHLH2 | EGL3 [Source:UniProtKB/TrEMBL;Acc:A0A384LNI2] |
| AT1G63720 | 2,07 | 7,28E-16 | - | At1g63720/F24D7_9 [Source:UniProtKB/TrEMBL;Acc:Q93W23] |
| AT1G63730 | 0,64 | 9,61E-09 | - | Disease resistance protein (TIR-NBS-LRR class) family [Source:UniProtKB/TrEMBL;Acc:Q9CAD8] |
| AT1G63750 | 0,84 | 4,96E-02 | - | Disease resistance protein (TIR-NBS-LRR class) family [Source:UniProtKB/TrEMBL;Acc:Q9CAE0] |
| AT1G63800 | 1,21 | 3,76E-22 | UBC5 | UBC5 [Source:UniProtKB/TrEMBL;Acc:A0A178WDU0] |
| AT1G63820 | 1,68 | 9,25E-06 | - | CCT motif family protein [Source:UniProtKB/TrEMBL;Acc:Q67ZF8] |
| AT1G63830 | 0,99 | 2,30E-42 | - | PLAC8 family protein [Source:UniProtKB/TrEMBL;Acc:Q9CAJ7] |
| AT1G63840 | 0,81 | 5,50E-28 | - | At1g63840/T12P18_14 [Source:UniProtKB/TrEMBL;Acc:Q9CAJ8] |
| AT1G63860 | -0,89 | 4,09E-04 | - | Disease resistance protein (TIR-NBS-LRR class) family [Source:UniProtKB/TrEMBL;Acc:F4I3S8] |
| AT1G63870 | 4,00 | 1,32E-04 | - | Disease resistance protein (TIR-NBS-LRR class) family [Source:UniProtKB/TrEMBL;Acc:Q9CAK0] |
| AT1G63930 | -2,99 | 6,31E-49 | ROH1 | At1g63930 [Source:UniProtKB/TrEMBL;Acc:Q9CAK4] |
| AT1G63940 | -0,60 | 1,12E-47 | MDAR5 | MDAR6 [Source:UniProtKB/TrEMBL;Acc:A0A178WFH3] |
| AT1G63990 | -1,62 | 1,67E-04 | SPO11-2 | Meiotic recombination protein SPO11-2 [Source:UniProtKB/Swiss-Prot;Acc:Q9M4A1] |
| AT1G64040 | 0,71 | 3,82E-61 | TOPP3 | Serine/threonine-protein phosphatase [Source:UniProtKB/TrEMBL;Acc:A0A178WMA0] |
| AT1G64050 | 0,59 | 4,71E-35 | - | F22C12.19 [Source:UniProtKB/TrEMBL;Acc:Q9SH55] |
| AT1G64090 | -1,14 | 2,10E-157 | RTNLB3 | Reticulon-like protein [Source:UniProtKB/TrEMBL;Acc:F4I596] |
| AT1G64100 | 1,50 | 8,84E-03 | - | Pentatricopeptide (PPR) repeat-containing protein [Source:UniProtKB/TrEMBL;Acc:A8MRJ8] |
| AT1G64110 | 0,60 | 1,21E-06 | - | p-loop containing nucleoside triphosphate hydrolases superfamily protein [Source:UniProtKB/TrEMBL;Acc:F4I5A3] |
| AT1G64170 | 2,31 | 9,60E-12 | CHX16 | Cation/H(+) antiporter 16 [Source:UniProtKB/Swiss-Prot;Acc:Q1HDT3] |
| AT1G64180 | 1,15 | 2,60E-07 | - | Intracellular protein transport protein USO1-like protein [Source:UniProtKB/TrEMBL;Acc:F4I5B1] |
| AT1G64210 | 2,32 | 6,63E-31 | - | Putative inactive receptor-like protein kinase At1g64210 [Source:UniProtKB/Swiss-Prot;Acc:Q9SH71] |
| AT1G64220 | 1,08 | 1,37E-56 | TOM7-2 | Mitochondrial import receptor subunit TOM7-2 [Source:UniProtKB/Swiss-Prot;Acc:Q3ECI7] |
| AT1G64230 | 1,15 | 1,88E-202 | UBC28 | Ubiquitin-conjugating enzyme 28 [Source:UniProtKB/TrEMBL;Acc:F4I5B8] |
| AT1G64253 | 0,90 | 9,51E-06 | - | DEK carboxy-terminal domain protein [Source:UniProtKB/TrEMBL;Acc:A0A1P8AUN6] |
| AT1G64260 | 1,77 | 3,20E-04 | - | MuDR family transposase [Source:UniProtKB/TrEMBL;Acc:Q9C7V1] |
| AT1G64295 | 4,59 | 1,09E-02 | - | F-box associated ubiquitination effector family protein [Source:UniProtKB/TrEMBL;Acc:F4I6P4] |
| AT1G64310 | 0,96 | 3,02E-06 | PCMP-E65 | Putative pentatricopeptide repeat-containing protein At1g64310 [Source:UniProtKB/Swiss-Prot;Acc:Q9C7V5] |
| AT1G64320 | 0,79 | 4,02E-09 | - | myosin heavy chain-related [Source:TAIR;Acc:AT1G64320] |
| AT1G64380 | 0,70 | 1,39E-06 | ERF061 | Ethylene-responsive transcription factor ERF061 [Source:UniProtKB/Swiss-Prot;Acc:Q9C7W2] |
| AT1G64390 | -2,00 | 3,94E-207 | AtGH9C2 | Endoglucanase 6 [Source:UniProtKB/Swiss-Prot;Acc:Q42059] |
| AT1G64440 | -0,93 | 4,14E-45 | UGE4 | UDP-glucose 4-epimerase 4 [Source:UniProtKB/Swiss-Prot;Acc:Q9C7W7] |
| AT1G64500 | 1,70 | 6,94E-03 | - | F1N19.7 [Source:UniProtKB/TrEMBL;Acc:Q9SGW5] |
| AT1G64520 | -0,60 | 1,13E-77 | RPN12A | RPN12a [Source:UniProtKB/TrEMBL;Acc:A0A178WH62] |
| AT1G64530 | -0,96 | 6,31E-74 | NLP6 | Protein NLP6 [Source:UniProtKB/Swiss-Prot;Acc:Q8RWY4] |
| AT1G64540 | 1,40 | 2,77E-03 | - | Putative F-box protein At1g64540 [Source:UniProtKB/Swiss-Prot;Acc:P0C2F8] |
| AT1G64563 | 1,28 | 5,14E-06 | - | other RNA [Source:TAIR;Acc:AT1G64563] |
| AT1G64600 | 0,87 | 9,32E-83 | - | At1g64600 [Source:UniProtKB/TrEMBL;Acc:Q8GW63] |
| AT1G64610 | 1,49 | 1,28E-19 | - | Transducin/WD40 repeat-like superfamily protein [Source:UniProtKB/TrEMBL;Acc:Q8VZK1] |
| AT1G64620 | 0,84 | 1,35E-10 | DOF1.8 | Dof zinc finger protein DOF1.8 [Source:UniProtKB/Swiss-Prot;Acc:Q84JQ8] |
| AT1G64640 | -1,16 | 2,04E-114 | ENODL8 | ENODL8 [Source:UniProtKB/TrEMBL;Acc:A0A178WAZ0] |
| AT1G64650 | -1,58 | 4,18E-182 | - | At1g64650 [Source:UniProtKB/TrEMBL;Acc:Q6NLR2] |
| AT1G64660 | 1,63 | 3,13E-179 | MGL | Methionine gamma-lyase [Source:UniProtKB/Swiss-Prot;Acc:Q9SGU9] |
| AT1G64690 | -0,88 | 1,28E-06 | BLT | branchless trichome [Source:TAIR;Acc:AT1G64690] |
| AT1G64720 | 0,95 | 1,78E-34 | CP5 | Polyketide cyclase/dehydrase and lipid transport superfamily protein [Source:UniProtKB/TrEMBL;Acc:Q9XIR9] |
| AT1G64750 | -0,65 | 3,85E-29 | ATDSS1(I) | Deletion of SUV3 suppressor 1(I) [Source:UniProtKB/TrEMBL;Acc:F4I886] |
| AT1G64820 | 1,31 | 4,33E-03 | DTX7 | Protein DETOXIFICATION 7 [Source:UniProtKB/Swiss-Prot;Acc:Q1PFG9] |
| AT1G64850 | -0,73 | 6,35E-30 | - | At1g64850/F13O11_15 [Source:UniProtKB/TrEMBL;Acc:Q9XIR0] |
| AT1G64900 | 2,47 | 0,00E+00 | CYP89A2 | Cytochrome P450 89A2 [Source:UniProtKB/Swiss-Prot;Acc:Q42602] |
| AT1G64910 | -3,24 | 4,41E-70 | UGT79B10 | UDP-glycosyltransferase 79B10 [Source:UniProtKB/Swiss-Prot;Acc:Q9XIQ5] |
| AT1G64920 | -2,78 | 1,83E-171 | UGT79B11 | UDP-glycosyltransferase 79B11 [Source:UniProtKB/Swiss-Prot;Acc:Q9XIQ4] |
| AT1G64930 | 2,27 | 9,83E-287 | CYP89A7 | CYP89A7 [Source:UniProtKB/TrEMBL;Acc:A0A178VZZ4] |
| AT1G64970 | 0,87 | 1,58E-40 | VTE4 | Tocopherol O-methyltransferase, chloroplastic [Source:UniProtKB/Swiss-Prot;Acc:Q9ZSK1] |
| AT1G65032 | 0,62 | 1,27E-07 | - | unknown protein; FUNCTIONS IN: molecular_function unknown; INVOLVED IN: biological_process unknown; LOCATED IN: cellular_component unknown; Ha. [Source:TAIR;Acc:AT1G65032] |
| AT1G65080 | 1,21 | 5,63E-24 | ALB3L2 | ALBINO3-like protein 2, chloroplastic [Source:UniProtKB/Swiss-Prot;Acc:Q8L718] |
| AT1G65120 | 0,66 | 1,11E-09 | - | Ubiquitin carboxyl-terminal hydrolase-related protein [Source:UniProtKB/TrEMBL;Acc:F4I9U7] |
| AT1G65130 | -0,79 | 9,98E-03 | - | Ubiquitin carboxyl-terminal hydrolase-related protein [Source:UniProtKB/TrEMBL;Acc:F4I9U9] |
| AT1G65150 | 2,61 | 4,09E-10 | - | T23K8.6 [Source:UniProtKB/TrEMBL;Acc:Q9S9J5] |
| AT1G65180 | -1,27 | 6,24E-13 | - | Cysteine/Histidine-rich C1 domain family protein [Source:UniProtKB/TrEMBL;Acc:Q9S9J8] |
| AT1G65190 | -5,54 | 3,16E-05 | - | Protein kinase superfamily protein [Source:UniProtKB/TrEMBL;Acc:Q9S9J9] |
| AT1G65230 | 0,84 | 1,74E-03 | - | Transmembrane protein, putative (DUF2358) [Source:UniProtKB/TrEMBL;Acc:Q8L604] |
| AT1G65250 | -0,81 | 3,95E-04 | - | Probable inactive receptor-like protein kinase At1g65250 [Source:UniProtKB/Swiss-Prot;Acc:O80795] |
| AT1G65260 | 0,64 | 1,94E-35 | VIPP1 | Membrane-associated protein VIPP1, chloroplastic [Source:UniProtKB/Swiss-Prot;Acc:O80796] |
| AT1G65270 | -0,77 | 2,83E-85 | - | AT1G65270 protein [Source:UniProtKB/TrEMBL;Acc:O80798] |
| AT1G65290 | -0,88 | 1,68E-120 | MTACP2 | Acyl carrier protein 2, mitochondrial [Source:UniProtKB/Swiss-Prot;Acc:O80800] |
| AT1G65295 | -0,74 | 5,01E-18 | - | Ubiquitin carboxyl-terminal hydrolase [Source:UniProtKB/TrEMBL;Acc:Q8H130] |
| AT1G65310 | -0,98 | 3,07E-05 | ATXTH17 | xyloglucan endotransglucosylase/hydrolase 17 [Source:TAIR;Acc:AT1G65310] |
| AT1G65330 | -5,26 | 1,54E-04 | PHE1 | MADS-box transcription factor PHERES 1 [Source:UniProtKB/Swiss-Prot;Acc:O80805] |
| AT1G65370 | 1,48 | 8,45E-97 | - | At1g65370/T8F5_15 [Source:UniProtKB/TrEMBL;Acc:Q940M1] |
| AT1G65450 | 2,87 | 2,90E-02 | - | HXXXD-type acyl-transferase family protein [Source:UniProtKB/TrEMBL;Acc:O80816] |
| AT1G65470 | -1,06 | 5,49E-33 | FAS1 | Chromatin assembly factor 1 subunit FAS1 [Source:UniProtKB/Swiss-Prot;Acc:Q9SXY0] |
| AT1G65481 | 2,02 | 9,39E-20 | - | unknown protein; FUNCTIONS IN: molecular_function unknown; INVOLVED IN: biological_process unknown; LOCATED IN: endomembrane system. [Source:TAIR;Acc:AT1G65481] |
| AT1G65540 | -1,06 | 2,02E-65 | - | LETM1-like protein [Source:UniProtKB/TrEMBL;Acc:F4IBH5] |
| AT1G65560 | 1,40 | 6,05E-44 | - | Zinc-binding dehydrogenase family protein [Source:UniProtKB/TrEMBL;Acc:F4IBH8] |
| AT1G65590 | 1,11 | 1,04E-146 | HEXO3 | Beta-hexosaminidase 3 [Source:UniProtKB/Swiss-Prot;Acc:Q8L7S6] |
| AT1G65660 | 0,73 | 1,37E-31 | SMP1 | SMP1 [Source:UniProtKB/TrEMBL;Acc:A0A178W8X4] |
| AT1G65730 | 0,74 | 4,17E-27 | YSL7 | Probable metal-nicotianamine transporter YSL7 [Source:UniProtKB/Swiss-Prot;Acc:Q9SHY2] |
| AT1G65900 | -2,00 | 1,21E-61 | - | At1g65900/F12P19_7 [Source:UniProtKB/TrEMBL;Acc:Q94A98] |
| AT1G65930 | -0,64 | 1,56E-67 | CICDH | Isocitrate dehydrogenase [NADP] [Source:UniProtKB/TrEMBL;Acc:A0A178W7K0] |
| AT1G65960 | -0,64 | 1,09E-14 | GAD2 | Glutamate decarboxylase 2 [Source:UniProtKB/Swiss-Prot;Acc:Q42472] |
| AT1G65970 | 3,67 | 1,32E-07 | PRXIIC | TPX2 [Source:UniProtKB/TrEMBL;Acc:A0A178WKG0] |
| AT1G65980 | 0,96 | 1,00E-201 | PRXIIB | Peroxiredoxin-2B [Source:UniProtKB/Swiss-Prot;Acc:Q9XEX2] |
| AT1G66030 | -0,71 | 2,11E-03 | CYP96A14P | Cytochrome P450-related [Source:UniProtKB/TrEMBL;Acc:Q9C8E2] |
| AT1G66070 | -0,64 | 1,03E-44 | - | Eukaryotic translation initiation factor 3 subunit J [Source:UniProtKB/TrEMBL;Acc:A0A178WCY1] |
| AT1G66140 | -1,31 | 1,84E-07 | ZFP4 | Zinc finger protein 4 [Source:UniProtKB/Swiss-Prot;Acc:Q39263] |
| AT1G66160 | -1,72 | 2,08E-10 | PUB20 | U-box domain-containing protein 20 [Source:UniProtKB/Swiss-Prot;Acc:Q9C8D1] |
| AT1G66180 | 1,47 | 5,67E-13 | - | Eukaryotic aspartyl protease family protein [Source:UniProtKB/TrEMBL;Acc:Q9C8C9] |
| AT1G66190 | 1,40 | 2,28E-11 | - | Uncharacterized protein F15E12.13 [Source:UniProtKB/TrEMBL;Acc:Q9C8C8] |
| AT1G66200 | 2,36 | 2,57E-79 | ATGSR2 | Glutamine synthetase [Source:UniProtKB/TrEMBL;Acc:F4ID91] |
| AT1G66235 | 3,10 | 8,24E-05 | - | unknown protein; BEST Arabidopsis thaliana protein match is: unknown protein (TAIR:AT1G24380.1); Ha. [Source:TAIR;Acc:AT1G66235] |
| AT1G66270 | -4,09 | 3,98E-159 | BGLU21 | BGLU21 [Source:UniProtKB/TrEMBL;Acc:A0A178WA93] |
| AT1G66280 | -1,38 | 2,82E-208 | BGLU22 | Beta-glucosidase 22 [Source:UniProtKB/Swiss-Prot;Acc:Q9C8Y9] |
| AT1G66320 | 4,06 | 4,18E-30 | - | FBD-associated F-box protein At1g66320 [Source:UniProtKB/Swiss-Prot;Acc:Q9C8Y5] |
| AT1G66350 | -0,62 | 2,40E-12 | RGL1 | DELLA protein RGL1 [Source:UniProtKB/Swiss-Prot;Acc:Q9C8Y3] |
| AT1G66390 | 3,22 | 1,02E-02 | MYB90 | Transcription factor MYB90 [Source:UniProtKB/Swiss-Prot;Acc:Q9ZTC3] |
| AT1G66410 | -0,79 | 1,21E-56 | CAM4 | Calmodulin 4 [Source:UniProtKB/TrEMBL;Acc:F4IEU4] |
| AT1G66430 | -0,95 | 1,96E-48 | - | Probable fructokinase-6, chloroplastic [Source:UniProtKB/Swiss-Prot;Acc:Q9C524] |
| AT1G66460 | -2,95 | 1,67E-04 | - | Protein kinase superfamily protein [Source:TAIR;Acc:AT1G66460] |
| AT1G66470 | -1,19 | 7,05E-47 | BHLH83 | RHD6 [Source:UniProtKB/TrEMBL;Acc:A0A178W430] |
| AT1G66480 | 1,03 | 4,29E-15 | - | Uncharacterized protein At1g66480 [Source:UniProtKB/Swiss-Prot;Acc:Q6NLC8] |
| AT1G66500 | 0,79 | 1,21E-05 | PCFS1 | Polyadenylation and cleavage factor homolog 1 [Source:UniProtKB/Swiss-Prot;Acc:Q9C710] |
| AT1G66540 | 2,01 | 5,52E-62 | - | At1g66540 [Source:UniProtKB/TrEMBL;Acc:A2RVN3] |
| AT1G66590 | 0,62 | 2,05E-11 | ATCOX19-1 | Cytochrome c oxidase 19-1 [Source:UniProtKB/TrEMBL;Acc:F4IEX1] |
| AT1G66650 | 0,64 | 2,20E-03 | - | E3 ubiquitin-protein ligase SINA-like 4 [Source:UniProtKB/Swiss-Prot;Acc:Q9C9M0] |
| AT1G66660 | 0,71 | 1,24E-15 | - | E3 ubiquitin-protein ligase [Source:UniProtKB/TrEMBL;Acc:F4IEX9] |
| AT1G66700 | -6,65 | 1,09E-07 | PXMT1 | Paraxanthine methyltransferase 1 [Source:UniProtKB/Swiss-Prot;Acc:Q9C9M3] |
| AT1G66725 | 1,70 | 3,80E-43 | MIR163 | MIR163; miRNA [Source:TAIR;Acc:AT1G66725] |
| AT1G66800 | -4,87 | 2,92E-21 | - | NAD(P)-binding Rossmann-fold superfamily protein [Source:UniProtKB/TrEMBL;Acc:F4HQ07] |
| AT1G66810 | -2,53 | 2,94E-28 | - | Zinc finger CCCH domain-containing protein 14 [Source:UniProtKB/Swiss-Prot;Acc:Q9C9N3] |
| AT1G66880 | 0,58 | 2,71E-14 | - | Protein kinase superfamily protein [Source:TAIR;Acc:AT1G66880] |
| AT1G66890 | 0,82 | 6,68E-05 | - | 50S ribosomal-like protein [Source:UniProtKB/TrEMBL;Acc:Q9C618] |
| AT1G66930 | 1,28 | 8,12E-07 | LRK10L-2.7 | Protein kinase superfamily protein [Source:TAIR;Acc:AT1G66930] |
| AT1G67020 | 1,51 | 4,49E-25 | - | F1O19.7 protein [Source:UniProtKB/TrEMBL;Acc:Q9FZH9] |
| AT1G67025 | 2,73 | 6,91E-03 | - | BEST Arabidopsis thaliana protein match is: Protein kinase superfamily protein (TAIR:AT1G18390.1); Ha. [Source:TAIR;Acc:AT1G67025] |
| AT1G67035 | -1,22 | 2,15E-11 | - | Homeobox Hox-B3-like protein [Source:UniProtKB/TrEMBL;Acc:Q5BPW2] |
| AT1G67050 | -1,16 | 2,45E-08 | - | F1O19.11 protein [Source:UniProtKB/TrEMBL;Acc:Q9FZH6] |
| AT1G67090 | -4,34 | 1,57E-02 | RBCS-1A | Ribulose bisphosphate carboxylase small chain [Source:UniProtKB/TrEMBL;Acc:A0A178WD57] |
| AT1G67120 | 1,00 | 4,04E-179 | MDN1 | ATPases;nucleotide binding;ATP binding;nucleoside-triphosphatases;transcription factor binding [Source:TAIR;Acc:AT1G67120] |
| AT1G67170 | -0,77 | 3,95E-24 | FLXL2 | Protein FLX-like 2 [Source:UniProtKB/Swiss-Prot;Acc:Q84TD8] |
| AT1G67180 | -1,13 | 4,56E-22 | - | F5A8.9 protein [Source:UniProtKB/TrEMBL;Acc:Q9ZW89] |
| AT1G67220 | 5,93 | 1,04E-06 | HAC2 | Histone acetyltransferase HAC2 [Source:UniProtKB/Swiss-Prot;Acc:Q9FYH1] |
| AT1G67270 | -2,08 | 1,20E-08 | - | Zinc-finger domain of monoamine-oxidase A repressor R1 protein [Source:TAIR;Acc:AT1G67270] |
| AT1G67340 | 0,72 | 6,11E-09 | - | F-box protein At1g67340 [Source:UniProtKB/Swiss-Prot;Acc:Q9FYF9] |
| AT1G67365 | 1,16 | 6,92E-10 | - | other RNA [Source:TAIR;Acc:AT1G67365] |
| AT1G67370 | 0,96 | 1,96E-16 | ASY1 | Meiosis-specific protein ASY1 [Source:UniProtKB/Swiss-Prot;Acc:F4HRV8] |
| AT1G67410 | -0,84 | 3,96E-33 | - | At1g67410 [Source:UniProtKB/TrEMBL;Acc:Q08AA5] |
| AT1G67430 | -1,13 | 7,05E-178 | RPL17B | 60S ribosomal protein L17-2 [Source:UniProtKB/Swiss-Prot;Acc:P51413] |
| AT1G67440 | 1,11 | 1,88E-15 | emb1688 | Minichromosome maintenance (MCM2/3/5) family protein [Source:TAIR;Acc:AT1G67440] |
| AT1G67460 | 2,03 | 3,89E-07 | - | - |
| AT1G67470 | 1,85 | 4,88E-04 | - | Inactive serine/threonine-protein kinase At1g67470 [Source:UniProtKB/Swiss-Prot;Acc:O64798] |
| AT1G67490 | -0,64 | 2,89E-60 | GCS1 | Mannosyl-oligosaccharide glucosidase GCS1 [Source:UniProtKB/Swiss-Prot;Acc:F4HTM3] |
| AT1G67510 | -0,61 | 1,39E-12 | - | Leucine-rich repeat protein kinase family protein [Source:UniProtKB/TrEMBL;Acc:O64794] |
| AT1G67570 | -0,70 | 6,99E-25 | - | Protein of unknown function (DUF3537) [Source:TAIR;Acc:AT1G67570] |
| AT1G67600 | 1,75 | 3,26E-12 | - | Acid phosphatase/vanadium-dependent haloperoxidase-related protein [Source:UniProtKB/TrEMBL;Acc:Q9FXC5] |
| AT1G67650 | 2,63 | 3,60E-96 | - | Signal recognition particle subunit SRP72 [Source:UniProtKB/TrEMBL;Acc:Q9FXD1] |
| AT1G67680 | -0,65 | 2,66E-64 | - | Signal recognition particle subunit SRP72 [Source:UniProtKB/TrEMBL;Acc:Q9FXD4] |
| AT1G67730 | -0,65 | 2,85E-40 | KCR1 | Very-long-chain 3-oxoacyl-CoA reductase 1 [Source:UniProtKB/Swiss-Prot;Acc:Q8L9C4] |
| AT1G67740 | -1,21 | 6,21E-12 | PSBY | Putative uncharacterized protein [Source:UniProtKB/TrEMBL;Acc:Q549N8] |
| AT1G67750 | -3,47 | 1,86E-61 | - | Probable pectate lyase 5 [Source:UniProtKB/Swiss-Prot;Acc:Q9FXD8] |
| AT1G67760 | -2,86 | 1,59E-82 | - | TCP-1/cpn60 chaperonin family protein [Source:UniProtKB/TrEMBL;Acc:Q67ZE4] |
| AT1G67780 | -0,71 | 9,02E-15 | - | Zinc-finger domain of monoamine-oxidase A repressor R1 protein [Source:UniProtKB/TrEMBL;Acc:F4HTR3] |
| AT1G67785 | -0,90 | 1,12E-86 | - | Putative uncharacterized protein [Source:UniProtKB/TrEMBL;Acc:Q8VZ65] |
| AT1G67810 | 0,97 | 2,74E-35 | SUFE2 | SufE-like protein 2, chloroplastic [Source:UniProtKB/Swiss-Prot;Acc:Q9FXE3] |
| AT1G67820 | 0,87 | 7,32E-11 | - | PP2C-type phosphatase AP2C4 [Source:UniProtKB/TrEMBL;Acc:F6LPR4] |
| AT1G67830 | -1,12 | 3,80E-05 | FXG1 | Alpha-L-fucosidase 3 [Source:UniProtKB/Swiss-Prot;Acc:Q9FXE5] |
| AT1G67920 | 1,82 | 3,55E-02 | - | At1g67920 [Source:UniProtKB/TrEMBL;Acc:Q9C9V8] |
| AT1G67930 | -0,76 | 8,60E-67 | - | Golgi transport complex protein-like protein [Source:UniProtKB/TrEMBL;Acc:Q9C9V9] |
| AT1G67940 | -0,93 | 1,88E-18 | ABCI17 | Sensitive to aluminum rhizotoxicity 1 [Source:UniProtKB/TrEMBL;Acc:D6RVY5] |
| AT1G67970 | 1,71 | 1,17E-24 | HSFA8 | Heat stress transcription factor A-8 [Source:UniProtKB/Swiss-Prot;Acc:Q9S7U5] |
| AT1G68000 | -0,83 | 5,50E-45 | PIS1 | CDP-diacylglycerol--inositol 3-phosphatidyltransferase 1 [Source:UniProtKB/Swiss-Prot;Acc:Q8LBA6] |
| AT1G68050 | 3,09 | 6,04E-48 | ADO3 | FKF1 [Source:UniProtKB/TrEMBL;Acc:A0A178W8F6] |
| AT1G68060 | -0,79 | 4,24E-68 | MAP70.1 | Microtubule-associated protein 70-1 [Source:UniProtKB/Swiss-Prot;Acc:Q9C9X0] |
| AT1G68100 | -0,96 | 4,03E-75 | IAR1 | IAA-alanine resistance protein 1 [Source:UniProtKB/Swiss-Prot;Acc:Q9M647] |
| AT1G68130 | -0,60 | 3,07E-17 | IDD14 | Protein indeterminate-domain 14 [Source:UniProtKB/Swiss-Prot;Acc:Q9C9X7] |
| AT1G68200 | -1,93 | 1,30E-03 | - | Zinc finger CCCH domain-containing protein 15 [Source:UniProtKB/Swiss-Prot;Acc:Q9C9F5] |
| AT1G68240 | 3,46 | 5,04E-18 | BHLH109 | Transcription factor bHLH109 [Source:UniProtKB/Swiss-Prot;Acc:Q5XVH0] |
| AT1G68250 | 5,80 | 3,30E-06 | - | At1g68250 [Source:UniProtKB/TrEMBL;Acc:Q9C9G0] |
| AT1G68300 | 0,73 | 2,50E-24 | - | Adenine nucleotide alpha hydrolases-like superfamily protein [Source:UniProtKB/TrEMBL;Acc:Q9C9G5] |
| AT1G68330 | -0,87 | 4,72E-14 | - | At1g68330 [Source:UniProtKB/TrEMBL;Acc:Q8RWC8] |
| AT1G68350 | -3,12 | 6,73E-06 | - | At1g68350 [Source:UniProtKB/TrEMBL;Acc:Q9C9H0] |
| AT1G68390 | 1,17 | 2,83E-16 | - | Core-2/I-branching beta-1,6-N-acetylglucosaminyltransferase family protein [Source:UniProtKB/TrEMBL;Acc:Q9M9C4] |
| AT1G68410 | 0,70 | 6,53E-31 | - | Probable protein phosphatase 2C 15 [Source:UniProtKB/Swiss-Prot;Acc:Q9M9C6] |
| AT1G68440 | 0,70 | 3,38E-30 | - | At1g68440/T2E12_1 [Source:UniProtKB/TrEMBL;Acc:Q9M9C9] |
| AT1G68470 | -0,92 | 5,15E-04 | GT17 | Probable xyloglucan galactosyltransferase GT17 [Source:UniProtKB/Swiss-Prot;Acc:Q9CA34] |
| AT1G68500 | 2,02 | 1,31E-08 | - | Uncharacterized protein At1g68500 [Source:UniProtKB/TrEMBL;Acc:Q84K42] |
| AT1G68540 | -1,57 | 5,50E-10 | TKPR2 | TKPR2 [Source:UniProtKB/TrEMBL;Acc:A0A178W2A9] |
| AT1G68550 | -0,69 | 8,95E-39 | ERF118 | Ethylene-responsive transcription factor ERF118 [Source:UniProtKB/Swiss-Prot;Acc:Q9CA27] |
| AT1G68560 | -1,04 | 2,90E-22 | XYL1 | Alpha-xylosidase 1 [Source:UniProtKB/Swiss-Prot;Acc:Q9S7Y7] |
| AT1G68570 | -1,42 | 2,13E-06 | NPF3.1 | Protein NRT1/ PTR FAMILY 3.1 [Source:UniProtKB/Swiss-Prot;Acc:Q9SX20] |
| AT1G68585 | 2,64 | 1,07E-04 | - | At1g68585 [Source:UniProtKB/TrEMBL;Acc:Q8LGG7] |
| AT1G68590 | -2,92 | 4,27E-26 | - | 30S ribosomal protein 3-1, chloroplastic [Source:UniProtKB/Swiss-Prot;Acc:Q9SX22] |
| AT1G68620 | 5,50 | 2,02E-09 | CXE6 | Probable carboxylesterase 6 [Source:UniProtKB/Swiss-Prot;Acc:Q9SX25] |
| AT1G68650 | -0,64 | 6,02E-30 | - | GDT1-like protein 5 [Source:UniProtKB/Swiss-Prot;Acc:Q9SX28] |
| AT1G68680 | -0,75 | 8,54E-42 | - | At1g68680 [Source:UniProtKB/TrEMBL;Acc:Q8L9R6] |
| AT1G68710 | 0,73 | 1,28E-36 | - | ATPase E1-E2 type family protein / haloacid dehalogenase-like hydrolase family protein [Source:TAIR;Acc:AT1G68710] |
| AT1G68740 | 2,96 | 7,56E-03 | PHO1-H1 | Phosphate transporter PHO1 homolog 1 [Source:UniProtKB/Swiss-Prot;Acc:Q93ZF5] |
| AT1G68760 | 0,98 | 1,98E-30 | NUDT1 | Nudix hydrolase 1 [Source:UniProtKB/Swiss-Prot;Acc:Q9CA40] |
| AT1G68795 | 1,67 | 3,24E-29 | CLE12 | CLAVATA3/ESR (CLE)-related protein 12 [Source:UniProtKB/Swiss-Prot;Acc:Q29PU4] |
| AT1G68820 | 1,37 | 1,78E-79 | - | Transmembrane Fragile-X-F-associated protein [Source:TAIR;Acc:AT1G68820] |
| AT1G68840 | 0,70 | 7,13E-26 | RAV2 | TEM2 [Source:UniProtKB/TrEMBL;Acc:A0A178WNP0] |
| AT1G68862 | 1,01 | 4,39E-20 | - | unknown protein; FUNCTIONS IN: molecular_function unknown; INVOLVED IN: biological_process unknown; LOCATED IN: cellular_component unknown; Ha. [Source:TAIR;Acc:AT1G68862] |
| AT1G68870 | 1,01 | 1,50E-08 | ATSOFL2 | At1g68870 [Source:UniProtKB/TrEMBL;Acc:Q9CA45] |
| AT1G68872 | 0,78 | 4,97E-24 | - | Unknown gene [Source:TAIR;Acc:AT1G68872] |
| AT1G68890 | -1,60 | 4,86E-40 | PHYLLO | Protein PHYLLO, chloroplastic [Source:UniProtKB/Swiss-Prot;Acc:Q15KI9] |
| AT1G69030 | -0,59 | 4,50E-42 | - | BSD domain-containing protein [Source:UniProtKB/TrEMBL;Acc:F4I0I6] |
| AT1G69080 | -0,60 | 8,40E-03 | - | Adenine nucleotide alpha hydrolases-like superfamily protein [Source:UniProtKB/TrEMBL;Acc:Q9LQB2] |
| AT1G69150 | -4,89 | 9,31E-04 | - | Cysteine/Histidine-rich C1 domain family protein [Source:UniProtKB/TrEMBL;Acc:F4I0J7] |
| AT1G69160 | 1,55 | 7,76E-09 | - | Protein BIG GRAIN 1-like E [Source:UniProtKB/Swiss-Prot;Acc:Q93Z37] |
| AT1G69240 | -8,93 | 1,03E-16 | MES15 | RHS9 [Source:UniProtKB/TrEMBL;Acc:A0A178W505] |
| AT1G69260 | -1,66 | 4,80E-46 | AFP1 | Ninja-family protein AFP1 [Source:UniProtKB/Swiss-Prot;Acc:Q9LQ98] |
| AT1G69280 | 0,93 | 4,26E-15 | - | Uncharacterized protein At1g69280 [Source:UniProtKB/TrEMBL;Acc:Q0WP94] |
| AT1G69360 | 0,70 | 6,95E-17 | - | Plant protein of unknown function (DUF863) [Source:TAIR;Acc:AT1G69360] |
| AT1G69420 | -0,59 | 1,21E-40 | PAT22 | S-acyltransferase [Source:UniProtKB/TrEMBL;Acc:A0A178WF77] |
| AT1G69430 | 1,03 | 6,69E-04 | - | Son of sevenless protein [Source:UniProtKB/TrEMBL;Acc:Q9C794] |
| AT1G69485 | 0,87 | 2,02E-05 | - | Ribosomal L32p protein family [Source:UniProtKB/TrEMBL;Acc:Q5Q0D8] |
| AT1G69490 | 0,93 | 2,11E-03 | NAC029 | NAP [Source:UniProtKB/TrEMBL;Acc:A0A178W8K0] |
| AT1G69520 | 0,66 | 3,69E-13 | - | S-adenosyl-L-methionine-dependent methyltransferases superfamily protein [Source:TAIR;Acc:AT1G69520] |
| AT1G69523 | 0,65 | 4,30E-02 | - | At1g69520/F10D13_17 [Source:UniProtKB/TrEMBL;Acc:Q941B1] |
| AT1G69526 | 1,07 | 1,75E-37 | - | S-adenosyl-L-methionine-dependent methyltransferases superfamily protein [Source:UniProtKB/TrEMBL;Acc:F4I261] |
| AT1G69530 | -1,08 | 3,40E-70 | ATEXPA1 | Expansin [Source:UniProtKB/TrEMBL;Acc:C0Z241] |
| AT1G69540 | 1,85 | 1,46E-04 | AGL94 | AGAMOUS-like 94 [Source:TAIR;Acc:AT1G69540] |
| AT1G69587 | 0,77 | 1,81E-18 | - | other RNA [Source:TAIR;Acc:AT1G69587] |
| AT1G69600 | 0,82 | 1,03E-33 | ZHD11 | Zinc-finger homeodomain protein 11 [Source:UniProtKB/Swiss-Prot;Acc:Q9SEZ1] |
| AT1G69610 | 0,95 | 1,74E-02 | - | Protein of unknown function (DUF1666) [Source:TAIR;Acc:AT1G69610] |
| AT1G69710 | 0,82 | 1,27E-16 | - | Regulator of chromosome condensation (RCC1) family with FYVE zinc finger domain-containing protein [Source:UniProtKB/TrEMBL;Acc:F4I287] |
| AT1G69750 | 0,62 | 2,26E-47 | COX19-2 | At1g66590 [Source:UniProtKB/TrEMBL;Acc:Q9C9L6] |
| AT1G69760 | -0,85 | 9,41E-27 | - | At1g69760 [Source:UniProtKB/TrEMBL;Acc:Q9C9L7] |
| AT1G69780 | -0,95 | 7,20E-67 | ATHB-13 | Homeobox-leucine zipper protein ATHB-13 [Source:UniProtKB/Swiss-Prot;Acc:Q8LC03] |
| AT1G69790 | 4,80 | 3,90E-31 | PBL18 | Probable serine/threonine-protein kinase PBL18 [Source:UniProtKB/Swiss-Prot;Acc:Q5XF79] |
| AT1G69840 | 0,66 | 1,29E-09 | HIR2 | Hypersensitive-induced response protein 2 [Source:UniProtKB/Swiss-Prot;Acc:Q9CAR7] |
| AT1G69850 | 2,55 | 7,22E-126 | NPF4.6 | Protein NRT1/ PTR FAMILY 4.6 [Source:UniProtKB/Swiss-Prot;Acc:Q8H157] |
| AT1G69870 | 7,01 | 2,50E-09 | NPF2.13 | Protein NRT1/ PTR FAMILY 2.13 [Source:UniProtKB/Swiss-Prot;Acc:Q8RX77] |
| AT1G69920 | -2,94 | 9,74E-03 | GSTU12 | Glutathione S-transferase U12 [Source:UniProtKB/Swiss-Prot;Acc:Q6NMS0] |
| AT1G69935 | 0,92 | 1,05E-07 | SHW1 | Protein SHORT HYPOCOTYL IN WHITE LIGHT 1 [Source:UniProtKB/Swiss-Prot;Acc:F4I3V6] |
| AT1G70000 | 1,56 | 5,92E-64 | - | At1g70000 [Source:UniProtKB/TrEMBL;Acc:O04544] |
| AT1G70080 | -0,71 | 1,27E-04 | TPS06 | Dolabella-3,7-dien-18-ol synthase TPS06 [Source:UniProtKB/Swiss-Prot;Acc:Q84UU9] |
| AT1G70090 | 0,58 | 1,28E-25 | GATL9 | Probable galacturonosyltransferase-like 9 [Source:UniProtKB/Swiss-Prot;Acc:O04536] |
| AT1G70140 | 2,53 | 8,59E-45 | FH8 | Formin-like protein 8 [Source:UniProtKB/Swiss-Prot;Acc:O04532] |
| AT1G70185 | -2,11 | 8,03E-04 | - | other RNA [Source:TAIR;Acc:AT1G70185] |
| AT1G70210 | -0,62 | 3,88E-03 | CYCD1-1 | Cyclin-D1-1 [Source:UniProtKB/Swiss-Prot;Acc:P42751] |
| AT1G70230 | -1,08 | 9,19E-30 | AXY4 | TBL27 [Source:UniProtKB/TrEMBL;Acc:A0A178WG99] |
| AT1G70290 | 1,08 | 8,47E-11 | TPS8 | Probable alpha,alpha-trehalose-phosphate synthase [UDP-forming] 8 [Source:UniProtKB/Swiss-Prot;Acc:Q0WUI9] |
| AT1G70310 | 0,67 | 2,11E-70 | SPDSYN2 | Spermidine synthase 2 [Source:UniProtKB/Swiss-Prot;Acc:O48661] |
| AT1G70330 | -0,67 | 1,26E-22 | ENT1 | Equilibrative nucleotide transporter 1 [Source:UniProtKB/Swiss-Prot;Acc:Q8VXY7] |
| AT1G70360 | 0,60 | 2,75E-08 | - | F-box family protein [Source:TAIR;Acc:AT1G70360] |
| AT1G70370 | -1,50 | 1,19E-129 | PGL3 | Polygalacturonase 1 beta-like protein 3 [Source:UniProtKB/Swiss-Prot;Acc:P92990] |
| AT1G70390 | 4,43 | 4,14E-03 | - | Putative F-box protein At1g70390 [Source:UniProtKB/Swiss-Prot;Acc:O64597] |
| AT1G70420 | 1,39 | 1,70E-06 | - | At1g70420/F17O7_4 [Source:UniProtKB/TrEMBL;Acc:O64594] |
| AT1G70440 | 6,65 | 3,28E-46 | SRO3 | Probable inactive poly [ADP-ribose] polymerase SRO3 [Source:UniProtKB/Swiss-Prot;Acc:O64592] |
| AT1G70450 | 0,91 | 2,74E-02 | - | Protein kinase superfamily protein [Source:UniProtKB/TrEMBL;Acc:Q9CAL9] |
| AT1G70490 | -1,13 | 9,87E-91 | ARF2-A | ADP-ribosylation factor 2-B [Source:UniProtKB/Swiss-Prot;Acc:P0DH91] |
| AT1G70510 | 0,60 | 1,05E-02 | KNAT2 | KNOTTED-like from Arabidopsis thaliana 2 [Source:TAIR;Acc:AT1G70510] |
| AT1G70520 | 1,16 | 9,77E-04 | CRK2 | Cysteine-rich receptor-like protein kinase 2 [Source:UniProtKB/Swiss-Prot;Acc:Q9CAL3] |
| AT1G70570 | -0,71 | 1,09E-50 | - | Anthranilate phosphoribosyltransferase [Source:UniProtKB/TrEMBL;Acc:F4I5H7] |
| AT1G70600 | -1,39 | 1,66E-186 | RPL27AC | 60S ribosomal protein L27a-3 [Source:UniProtKB/Swiss-Prot;Acc:P49637] |
| AT1G70640 | 1,24 | 1,02E-02 | - | At1g70640 [Source:UniProtKB/TrEMBL;Acc:Q9S843] |
| AT1G70650 | 1,12 | 1,39E-15 | - | Ran BP2/NZF zinc finger-like superfamily protein [Source:UniProtKB/TrEMBL;Acc:F4I6V2] |
| AT1G70660 | 0,94 | 8,32E-11 | UEV1B | Ubiquitin-conjugating enzyme E2 variant 1B [Source:UniProtKB/Swiss-Prot;Acc:Q9CAB6] |
| AT1G70680 | 1,89 | 3,72E-02 | PXG5 | Probable peroxygenase 5 [Source:UniProtKB/Swiss-Prot;Acc:Q9CAB8] |
| AT1G70720 | 3,94 | 1,93E-02 | - | Plant invertase/pectin methylesterase inhibitor superfamily protein [Source:TAIR;Acc:AT1G70720] |
| AT1G70730 | -0,71 | 1,01E-61 | PGM2 | Phosphoglucomutase/phosphomannomutase family protein [Source:UniProtKB/TrEMBL;Acc:F4I6W4] |
| AT1G70770 | -0,62 | 2,52E-93 | - | AT1G70770 protein [Source:UniProtKB/TrEMBL;Acc:Q9S791] |
| AT1G70850 | -1,39 | 3,06E-16 | MLP34 | MLP34 [Source:UniProtKB/TrEMBL;Acc:A0A384KEJ8] |
| AT1G70940 | -0,93 | 5,16E-64 | PIN3 | Auxin efflux carrier component 3 [Source:UniProtKB/Swiss-Prot;Acc:Q9S7Z8] |
| AT1G70980 | 0,91 | 3,28E-102 | SYNC3 | Asparagine--tRNA ligase, cytoplasmic 3 [Source:UniProtKB/Swiss-Prot;Acc:Q9SSK1] |
| AT1G70990 | 1,06 | 1,11E-14 | - | At1g70990 [Source:UniProtKB/TrEMBL;Acc:A0JQ68] |
| AT1G71000 | 6,19 | 3,06E-07 | - | Chaperone DnaJ-domain superfamily protein [Source:UniProtKB/TrEMBL;Acc:F4I8D6] |
| AT1G71002 | 1,88 | 9,39E-03 | MIR858A | MIR858a; miRNA [Source:TAIR;Acc:AT1G71002] |
| AT1G71015 | 1,05 | 6,28E-16 | - | At1g71015 [Source:UniProtKB/TrEMBL;Acc:Q9SSJ7] |
| AT1G71040 | 1,73 | 8,63E-32 | LPR2 | Multicopper oxidase LPR2 [Source:UniProtKB/Swiss-Prot;Acc:Q949X9] |
| AT1G71050 | -1,84 | 1,86E-02 | HIPP20 | HIPP20 [Source:UniProtKB/TrEMBL;Acc:A0A178W5Y0] |
| AT1G71070 | -0,70 | 1,06E-57 | - | At1g71070/F23N20_6 [Source:UniProtKB/TrEMBL;Acc:Q9C9A1] |
| AT1G71110 | 0,80 | 3,16E-55 | - | unknown protein; FUNCTIONS IN: molecular_function unknown; INVOLVED IN: biological_process unknown; LOCATED IN: endomembrane system; BEST Arabidopsis thaliana protein match is: unknown protein (TAIR:AT2G12400.1); Ha. [Source:TAIR;Acc:AT1G71110] |
| AT1G71150 | 1,56 | 3,59E-04 | - | Cyclin-D1-binding protein [Source:UniProtKB/TrEMBL;Acc:Q9C993] |
| AT1G71270 | -0,74 | 5,61E-60 | VPS52 | Vacuolar protein sorting-associated protein 52 A [Source:UniProtKB/Swiss-Prot;Acc:Q94KD3] |
| AT1G71280 | 3,07 | 1,71E-90 | RH55 | DEAD-box ATP-dependent RNA helicase 55 [Source:UniProtKB/Swiss-Prot;Acc:Q9FVV4] |
| AT1G71330 | 0,64 | 2,77E-05 | NAP5 | Probable non-intrinsic ABC protein 5 [Source:UniProtKB/Swiss-Prot;Acc:Q9FVV9] |
| AT1G71360 | -0,92 | 1,49E-27 | - | Galactose-binding protein [Source:TAIR;Acc:AT1G71360] |
| AT1G71370 | 1,83 | 2,07E-02 | RH49 | DEAD-box ATP-dependent RNA helicase 49 [Source:UniProtKB/Swiss-Prot;Acc:Q8GXD6] |
| AT1G71380 | -0,67 | 6,88E-07 | CEL3 | Endoglucanase [Source:UniProtKB/TrEMBL;Acc:Q0V7W1] |
| AT1G71420 | 0,65 | 8,37E-04 | PCMP-H70 | Pentatricopeptide repeat-containing protein At1g71420 [Source:UniProtKB/Swiss-Prot;Acc:Q9C9H9] |
| AT1G71440 | -0,80 | 5,50E-49 | TFCE | Tubulin-folding cofactor E [Source:UniProtKB/Swiss-Prot;Acc:Q8GRL7] |
| AT1G71460 | 1,19 | 9,21E-24 | PCMP-A3 | Pentatricopeptide repeat-containing protein At1g71460, chloroplastic [Source:UniProtKB/Swiss-Prot;Acc:Q9C9I3] |
| AT1G71480 | 0,72 | 7,27E-04 | - | AT1G71480 protein [Source:UniProtKB/TrEMBL;Acc:Q9C9I5] |
| AT1G71490 | 0,62 | 4,68E-07 | PCMP-E67 | Pentatricopeptide repeat-containing protein At1g71490 [Source:UniProtKB/Swiss-Prot;Acc:Q9C9I6] |
| AT1G71692 | -1,45 | 2,47E-25 | AGL12 | Agamous-like MADS-box protein AGL12 [Source:UniProtKB/Swiss-Prot;Acc:Q38841] |
| AT1G71750 | -0,59 | 1,41E-04 | HGPT | Hypoxanthine phosphoribosyltransferase [Source:UniProtKB/TrEMBL;Acc:F4IA25] |
| AT1G71760 | -1,10 | 2,87E-11 | - | Putative uncharacterized protein [Source:UniProtKB/TrEMBL;Acc:Q5BPV4] |
| AT1G71780 | -1,11 | 6,66E-91 | - | unknown protein; FUNCTIONS IN: molecular_function unknown; INVOLVED IN: biological_process unknown; LOCATED IN: endoplasmic reticulum; EXPRESSED IN: 25 plant structures; EXPRESSED DURING: 15 growth stages; Ha. [Source:TAIR;Acc:AT1G71780] |
| AT1G71790 | 0,66 | 6,30E-42 | - | Probable F-actin-capping protein subunit beta [Source:UniProtKB/Swiss-Prot;Acc:Q9M9G7] |
| AT1G71880 | 0,71 | 4,88E-09 | SUC1 | Sucrose transport protein SUC1 [Source:UniProtKB/Swiss-Prot;Acc:Q39232] |
| AT1G71900 | -0,95 | 5,19E-44 | - | Protein of unknown function (DUF803) [Source:TAIR;Acc:AT1G71900] |
| AT1G71930 | -1,28 | 9,84E-03 | NAC030 | NAC domain-containing protein 30 [Source:UniProtKB/Swiss-Prot;Acc:Q9C8W9] |
| AT1G71940 | -0,92 | 8,10E-62 | - | SNARE associated Golgi protein family [Source:UniProtKB/TrEMBL;Acc:F4IBM1] |
| AT1G71970 | -0,73 | 6,37E-06 | - | At1g71970/F17M19_12 [Source:UniProtKB/TrEMBL;Acc:Q9C8W5] |
| AT1G71990 | 1,06 | 5,71E-33 | FUT13 | At1g71990 [Source:UniProtKB/TrEMBL;Acc:Q0V7Y9] |
| AT1G72070 | 2,23 | 2,46E-05 | - | Chaperone DnaJ-domain superfamily protein [Source:UniProtKB/TrEMBL;Acc:F4IBN6] |
| AT1G72090 | 0,59 | 2,85E-39 | - | At1g72090/F28P5_4 [Source:UniProtKB/TrEMBL;Acc:Q9C7H2] |
| AT1G72100 | 1,93 | 2,50E-11 | - | Late embryogenesis abundant domain-containing protein / LEA domain-containing protein [Source:UniProtKB/TrEMBL;Acc:Q9C7H3] |
| AT1G72120 | 2,99 | 1,44E-113 | NPF5.14 | Protein NRT1/ PTR FAMILY 5.14 [Source:UniProtKB/Swiss-Prot;Acc:Q8VZE2] |
| AT1G72140 | -1,97 | 1,24E-10 | NPF5.12 | Protein NRT1/ PTR FAMILY 5.12 [Source:UniProtKB/Swiss-Prot;Acc:Q9C7U1] |
| AT1G72170 | -0,76 | 6,54E-50 | - | MICOS complex subunit, putative (DUF543) [Source:UniProtKB/TrEMBL;Acc:Q8GWC7] |
| AT1G72175 | 0,95 | 2,05E-30 | - | E3 ubiquitin-protein ligase RNF170-like protein (DUF 1232) [Source:UniProtKB/TrEMBL;Acc:Q93WJ5] |
| AT1G72200 | -1,56 | 3,19E-11 | ATL11 | RING-H2 finger protein ATL11 [Source:UniProtKB/Swiss-Prot;Acc:Q84W40] |
| AT1G72220 | -1,35 | 5,72E-03 | ATL54 | RING-H2 finger protein ATL54 [Source:UniProtKB/Swiss-Prot;Acc:Q8LFY8] |
| AT1G72230 | -1,57 | 3,75E-13 | - | At1g72230/T9N14_17 [Source:UniProtKB/TrEMBL;Acc:Q9C7T2] |
| AT1G72270 | 1,10 | 5,79E-07 | - | CONTAINS InterPro DOMAIN/s: Ribosome 60S biogenesis N-terminal (InterPro:IPR021714); BEST Arabidopsis thaliana protein match is: unknown protein (TAIR:AT4G27010.1); Ha. [Source:TAIR;Acc:AT1G72270] |
| AT1G72330 | 3,37 | 0,00E+00 | ALAAT2 | alanine aminotransferase 2 [Source:TAIR;Acc:AT1G72330] |
| AT1G72360 | 1,42 | 1,23E-06 | HRE1 | Integrase-type DNA-binding superfamily protein [Source:UniProtKB/TrEMBL;Acc:F4IDA7] |
| AT1G72370 | -0,84 | 2,72E-118 | RPSAA | 40S ribosomal protein SA [Source:UniProtKB/TrEMBL;Acc:A0A178WNA1] |
| AT1G72416 | 1,49 | 4,47E-23 | - | Chaperone DnaJ-domain superfamily protein [Source:UniProtKB/TrEMBL;Acc:B3H5X6] |
| AT1G72430 | 1,34 | 2,58E-11 | SAUR78 | Auxin-responsive protein SAUR78 [Source:UniProtKB/Swiss-Prot;Acc:Q9C9E1] |
| AT1G72450 | 2,80 | 9,80E-12 | TIFY11B | TIFY11B [Source:UniProtKB/TrEMBL;Acc:A0A178W2J3] |
| AT1G72460 | 4,44 | 3,41E-08 | - | Leucine-rich repeat protein kinase family protein [Source:UniProtKB/TrEMBL;Acc:Q9C9E4] |
| AT1G72490 | 1,25 | 7,40E-51 | - | unknown protein; BEST Arabidopsis thaliana protein match is: unknown protein (TAIR:AT1G17400.1); Ha. [Source:TAIR;Acc:AT1G72490] |
| AT1G72500 | 0,64 | 4,04E-24 | - | LOCATED IN: plasma membrane; EXPRESSED IN: 23 plant structures; EXPRESSED DURING: 13 growth stages; CONTAINS InterPro DOMAIN/s: von Willebrand factor, type A (InterPro:IPR002035); BEST Arabidopsis thaliana protein match is: inter-alpha-trypsin inhib /.../eavy chain-related (TAIR:AT1G19110.1); Ha. [Source:TAIR;Acc:AT1G72500] |
| AT1G72520 | 0,75 | 4,47E-02 | LOX4 | Lipoxygenase 4, chloroplastic [Source:UniProtKB/Swiss-Prot;Acc:Q9FNX8] |
| AT1G72530 | 1,12 | 3,01E-13 | - | Plastid developmental protein DAG [Source:UniProtKB/TrEMBL;Acc:F4IDD2] |
| AT1G72540 | 1,47 | 1,83E-14 | - | Putative receptor-like protein kinase At1g72540 [Source:UniProtKB/Swiss-Prot;Acc:Q9CAH1] |
| AT1G72590 | -1,57 | 6,11E-12 | PPRD1 | Polyprenol reductase 1 [Source:UniProtKB/Swiss-Prot;Acc:Q9CAH5] |
| AT1G72600 | -1,90 | 1,04E-06 | - | At1g72600 [Source:UniProtKB/TrEMBL;Acc:Q9CAH6] |
| AT1G72620 | -2,79 | 4,94E-04 | - | Alpha/beta-Hydrolases superfamily protein [Source:UniProtKB/TrEMBL;Acc:F4IDE6] |
| AT1G72630 | -0,94 | 1,35E-23 | EFL2 | Protein ELF4-LIKE 2 [Source:UniProtKB/Swiss-Prot;Acc:Q94BS8] |
| AT1G72660 | 5,71 | 1,58E-254 | DRG2 | Developmentally-regulated G-protein 2 [Source:UniProtKB/Swiss-Prot;Acc:Q9CAI1] |
| AT1G72730 | -1,29 | 2,35E-160 | TIF4A-3 | Eukaryotic initiation factor 4A-3 [Source:UniProtKB/Swiss-Prot;Acc:Q9CAI7] |
| AT1G72740 | 1,24 | 7,39E-84 | - | Telomere repeat-binding factor 5 [Source:UniProtKB/Swiss-Prot;Acc:F4IEY4] |
| AT1G72760 | 1,51 | 6,34E-05 | - | Protein kinase superfamily protein [Source:TAIR;Acc:AT1G72760] |
| AT1G72810 | -0,80 | 3,30E-12 | TS2 | Threonine synthase 2, chloroplastic [Source:UniProtKB/Swiss-Prot;Acc:Q9SSP5] |
| AT1G72820 | 0,75 | 7,58E-19 | - | At1g72820/F3N23_2 [Source:UniProtKB/TrEMBL;Acc:Q9SSP4] |
| AT1G72830 | -0,75 | 2,34E-12 | HAP2C | Nuclear factor Y, subunit A3 [Source:UniProtKB/TrEMBL;Acc:F4IEZ6] |
| AT1G72850 | 0,75 | 3,67E-03 | - | Disease resistance protein (TIR-NBS class) [Source:UniProtKB/TrEMBL;Acc:Q9SSP1] |
| AT1G72852 | 1,87 | 1,52E-02 | - | Potential natural antisense gene, locus overlaps with AT1G72850 [Source:TAIR;Acc:AT1G72852] |
| AT1G72860 | -1,12 | 1,65E-03 | - | Disease resistance protein (TIR-NBS-LRR class) family [Source:TAIR;Acc:AT1G72860] |
| AT1G72870 | -1,80 | 9,20E-15 | - | Disease resistance protein (TIR-NBS class) [Source:UniProtKB/TrEMBL;Acc:Q9SSN9] |
| AT1G72900 | 2,96 | 5,69E-128 | - | Similar to part of disease resistance protein [Source:UniProtKB/TrEMBL;Acc:Q9SSN6] |
| AT1G72920 | 0,75 | 4,71E-04 | - | Similar to part of disease resistance protein [Source:UniProtKB/TrEMBL;Acc:Q9SSN4] |
| AT1G72930 | 1,82 | 1,96E-19 | TIR | Toll/interleukin-1 receptor-like protein [Source:UniProtKB/Swiss-Prot;Acc:Q9SSN3] |
| AT1G72970 | -0,79 | 1,03E-16 | HTH | HTH [Source:UniProtKB/TrEMBL;Acc:A0A178WG21] |
| AT1G73066 | 1,21 | 2,10E-07 | - | Highly similar to receptor-like protein kinase [Source:UniProtKB/TrEMBL;Acc:Q9SSM0] |
| AT1G73130 | 1,11 | 6,76E-13 | - | F3N23.33 protein [Source:UniProtKB/TrEMBL;Acc:Q9S781] |
| AT1G73230 | -0,79 | 5,23E-107 | - | Nascent polypeptide-associated complex subunit beta [Source:UniProtKB/Swiss-Prot;Acc:Q9CAT7] |
| AT1G73250 | -0,60 | 9,64E-28 | GER1 | GDP-L-fucose synthase 1 [Source:UniProtKB/Swiss-Prot;Acc:O49213] |
| AT1G73260 | 4,17 | 3,28E-46 | KTI1 | Kunitz trypsin inhibitor 1 [Source:UniProtKB/Swiss-Prot;Acc:Q8RXD5] |
| AT1G73270 | 0,73 | 3,82E-02 | SCPL6 | Serine carboxypeptidase-like 6 [Source:UniProtKB/Swiss-Prot;Acc:Q9CAU0] |
| AT1G73340 | 1,06 | 3,52E-53 | - | Cytochrome P450 superfamily protein [Source:TAIR;Acc:AT1G73340] |
| AT1G73360 | 0,60 | 4,50E-07 | HDG11 | Homeobox-leucine zipper protein HDG11 [Source:UniProtKB/Swiss-Prot;Acc:Q9FX31] |
| AT1G73430 | -0,60 | 4,17E-46 | - | Sec34-like family protein [Source:UniProtKB/TrEMBL;Acc:F4HQ84] |
| AT1G73480 | 1,16 | 2,99E-25 | - | Alpha/beta-Hydrolases superfamily protein [Source:UniProtKB/TrEMBL;Acc:Q94AM5] |
| AT1G73500 | 1,17 | 3,39E-37 | MKK9 | Mitogen-activated protein kinase kinase 9 [Source:UniProtKB/Swiss-Prot;Acc:Q9FX43] |
| AT1G73530 | 1,01 | 6,39E-15 | ORRM6 | Organelle RRM domain-containing protein 6, chloroplastic [Source:UniProtKB/Swiss-Prot;Acc:Q9FX45] |
| AT1G73580 | -3,78 | 1,15E-09 | CAR3 | Protein C2-DOMAIN ABA-RELATED 3 [Source:UniProtKB/Swiss-Prot;Acc:Q9C6B7] |
| AT1G73620 | -1,25 | 7,49E-87 | - | Pathogenesis-related thaumatin superfamily protein [Source:UniProtKB/TrEMBL;Acc:Q9C9U9] |
| AT1G73640 | -1,38 | 7,81E-07 | RABA6A | Ras-related protein RABA6a [Source:UniProtKB/Swiss-Prot;Acc:Q9C9U7] |
| AT1G73655 | 1,54 | 2,58E-18 | FKBP17-3 | Peptidylprolyl isomerase [Source:UniProtKB/TrEMBL;Acc:A0A178WAL2] |
| AT1G73660 | 1,10 | 1,35E-19 | SIS8 | Probable serine/threonine-protein kinase SIS8 [Source:UniProtKB/Swiss-Prot;Acc:Q9C9U5] |
| AT1G73680 | -1,70 | 4,66E-06 | ALPHA DOX2 | alpha dioxygenase [Source:TAIR;Acc:AT1G73680] |
| AT1G73700 | 1,05 | 9,13E-07 | DTX17 | Protein DETOXIFICATION 17 [Source:UniProtKB/Swiss-Prot;Acc:Q9C9U1] |
| AT1G73780 | -2,58 | 1,14E-54 | - | Bifunctional inhibitor/lipid-transfer protein/seed storage 2S albumin superfamily protein [Source:UniProtKB/TrEMBL;Acc:Q9C9T4] |
| AT1G73860 | -2,02 | 8,12E-10 | KIN14P | Kinesin-like protein KIN-14P [Source:UniProtKB/Swiss-Prot;Acc:Q0WN69] |
| AT1G73885 | -1,12 | 1,25E-14 | - | unknown protein; FUNCTIONS IN: molecular_function unknown; INVOLVED IN: biological_process unknown; LOCATED IN: chloroplast thylakoid membrane, chloroplast; Ha. [Source:TAIR;Acc:AT1G73885] |
| AT1G73950 | 0,69 | 1,44E-14 | - | Transmembrane Fragile-X-F-associated protein [Source:UniProtKB/TrEMBL;Acc:F4HS21] |
| AT1G73965 | 1,00 | 6,06E-13 | CLE13 | CLAVATA3/ESR (CLE)-related protein 13 [Source:UniProtKB/Swiss-Prot;Acc:Q6NMF0] |
| AT1G74030 | -0,85 | 7,88E-113 | ENO1 | Enolase 1, chloroplastic [Source:UniProtKB/Swiss-Prot;Acc:Q9C9C4] |
| AT1G74088 | 0,61 | 1,81E-05 | - | FUNCTIONS IN: molecular_function unknown; INVOLVED IN: biological_process unknown; LOCATED IN: endomembrane system; BEST Arabidopsis thaliana protein match is: galacturonosyltransferase 11 (TAIR:AT1G18580.1). [Source:TAIR;Acc:AT1G74088] |
| AT1G74090 | -1,30 | 1,81E-19 | SOT18 | Sulfotransferase [Source:UniProtKB/TrEMBL;Acc:T1P4U2] |
| AT1G74100 | -1,41 | 3,51E-143 | SOT16 | Sulfotransferase [Source:UniProtKB/TrEMBL;Acc:M1EU36] |
| AT1G74130 | -4,09 | 3,08E-02 | RBL16 | Rhomboid-like protein 16, chloroplastic [Source:UniProtKB/Swiss-Prot;Acc:Q84WG3] |
| AT1G74150 | 1,23 | 9,44E-12 | - | Galactose oxidase/kelch repeat superfamily protein [Source:UniProtKB/TrEMBL;Acc:F4HTV1] |
| AT1G74190 | 3,57 | 3,86E-02 | AtRLP15 | Receptor-like protein 15 [Source:UniProtKB/Swiss-Prot;Acc:Q9C6A8] |
| AT1G74210 | -0,91 | 5,22E-56 | GDPD5 | GDPD5 [Source:UniProtKB/TrEMBL;Acc:A0A178W3R5] |
| AT1G74240 | 1,33 | 1,11E-90 | - | Mitochondrial substrate carrier family protein [Source:UniProtKB/TrEMBL;Acc:Q8GYH1] |
| AT1G74270 | -0,69 | 7,37E-82 | RPL35AC | 60S ribosomal protein L35a-3 [Source:UniProtKB/Swiss-Prot;Acc:Q9C912] |
| AT1G74290 | 2,60 | 2,24E-03 | - | Alpha/beta-Hydrolases superfamily protein [Source:UniProtKB/TrEMBL;Acc:Q9C914] |
| AT1G74300 | 0,59 | 2,77E-09 | - | Alpha/beta-Hydrolases superfamily protein [Source:UniProtKB/TrEMBL;Acc:Q9C915] |
| AT1G74310 | 2,19 | 2,15E-177 | CLPB1 | Chaperone protein ClpB1 [Source:UniProtKB/Swiss-Prot;Acc:P42730] |
| AT1G74330 | 0,84 | 8,24E-17 | - | Protein kinase superfamily protein [Source:UniProtKB/TrEMBL;Acc:F4HTX0] |
| AT1G74350 | -0,67 | 2,22E-04 | - | - |
| AT1G74380 | -1,06 | 4,30E-76 | XXT5 | Probable xyloglucan 6-xylosyltransferase 5 [Source:UniProtKB/Swiss-Prot;Acc:Q9CA75] |
| AT1G74410 | 0,86 | 1,16E-12 | ATL24 | NEP1-interacting protein-like 2 [Source:UniProtKB/Swiss-Prot;Acc:Q8LBA0] |
| AT1G74458 | -3,67 | 3,35E-05 | - | Transmembrane protein [Source:UniProtKB/TrEMBL;Acc:Q56YK7] |
| AT1G74470 | -0,88 | 1,29E-32 | CHLP | Geranylgeranyl diphosphate reductase, chloroplastic [Source:UniProtKB/Swiss-Prot;Acc:Q9CA67] |
| AT1G74500 | -0,81 | 1,80E-71 | PRE3 | Transcription factor PRE3 [Source:UniProtKB/Swiss-Prot;Acc:Q9CA64] |
| AT1G74550 | 2,12 | 2,46E-05 | CYP98A9 | Cytochrome P450 98A9 [Source:UniProtKB/Swiss-Prot;Acc:Q9CA60] |
| AT1G74560 | 0,64 | 6,85E-58 | NRP1 | NAP1-related protein 1 [Source:TAIR;Acc:AT1G74560] |
| AT1G74590 | 0,84 | 3,75E-10 | GSTU10 | Glutathione S-transferase U10 [Source:UniProtKB/Swiss-Prot;Acc:Q9CA57] |
| AT1G74680 | 0,76 | 7,89E-13 | - | At1g74680/F1M20_36 [Source:UniProtKB/TrEMBL;Acc:Q93ZD5] |
| AT1G74710 | -0,90 | 1,76E-17 | EDS16 | ADC synthase superfamily protein [Source:TAIR;Acc:AT1G74710] |
| AT1G74730 | -0,63 | 1,19E-03 | - | Transmembrane protein, putative (DUF1118) [Source:UniProtKB/TrEMBL;Acc:Q94F10] |
| AT1G74750 | 0,70 | 3,63E-26 | - | Pentatricopeptide repeat-containing protein At1g74750 [Source:UniProtKB/Swiss-Prot;Acc:Q9SSF9] |
| AT1G74770 | -1,86 | 4,70E-46 | - | Zinc finger protein BRUTUS-like At1g74770 [Source:UniProtKB/Swiss-Prot;Acc:F4HVS0] |
| AT1G74800 | -0,95 | 4,13E-47 | GALT5 | Hydroxyproline O-galactosyltransferase GALT5 [Source:UniProtKB/Swiss-Prot;Acc:Q8RX55] |
| AT1G74810 | 2,28 | 4,45E-216 | BOR5 | Putative boron transporter 5 [Source:UniProtKB/Swiss-Prot;Acc:Q9SSG5] |
| AT1G74830 | -3,16 | 6,34E-05 | MYOB6 | Probable myosin-binding protein 6 [Source:UniProtKB/Swiss-Prot;Acc:F4HVS6] |
| AT1G74850 | -0,68 | 8,91E-19 | PTAC2 | PTAC2 [Source:UniProtKB/TrEMBL;Acc:A0A178WNJ2] |
| AT1G74860 | 0,60 | 2,23E-07 | - | At1g74860 [Source:UniProtKB/TrEMBL;Acc:Q9SSG6] |
| AT1G74870 | 1,50 | 1,38E-19 | - | At1g74870 [Source:UniProtKB/TrEMBL;Acc:Q9S7I7] |
| AT1G74875 | -3,33 | 3,43E-02 | - | FUNCTIONS IN: molecular_function unknown; INVOLVED IN: biological_process unknown; LOCATED IN: cellular_component unknown; BEST Arabidopsis thaliana protein match is: F-box family protein (TAIR:AT1G67623.1); Ha. [Source:TAIR;Acc:AT1G74875] |
| AT1G74880 | -2,26 | 6,76E-08 | ndhO | NAD(P)H-quinone oxidoreductase subunit O, chloroplastic [Source:UniProtKB/Swiss-Prot;Acc:Q9S829] |
| AT1G74890 | -1,99 | 2,86E-10 | ARR15 | Two-component response regulator ARR15 [Source:UniProtKB/Swiss-Prot;Acc:Q7G8V2] |
| AT1G74900 | 1,03 | 2,05E-06 | OTP43 | Pentatricopeptide repeat (PPR) superfamily protein [Source:TAIR;Acc:AT1G74900] |
| AT1G74910 | -0,94 | 3,86E-115 | - | ADP-glucose pyrophosphorylase family protein [Source:UniProtKB/TrEMBL;Acc:Q9C9P3] |
| AT1G74940 | -2,31 | 1,47E-17 | FLZ13 | FCS-Like Zinc finger 13 [Source:UniProtKB/Swiss-Prot;Acc:Q8GRN0] |
| AT1G74960 | -0,70 | 5,73E-35 | KAS2 | 3-oxoacyl-[acyl-carrier-protein] synthase II, chloroplastic [Source:UniProtKB/Swiss-Prot;Acc:Q9C9P4] |
| AT1G75030 | -2,51 | 2,01E-02 | ATLP-3 | At1g75030 [Source:UniProtKB/TrEMBL;Acc:Q9C9P9] |
| AT1G75060 | 1,26 | 3,53E-12 | - | Histone deacetylase complex subunit [Source:UniProtKB/TrEMBL;Acc:Q9C9Q1] |
| AT1G75100 | 0,82 | 1,47E-06 | JAC1 | JAC1 [Source:UniProtKB/TrEMBL;Acc:A0A178WHU1] |
| AT1G75120 | 0,98 | 1,53E-19 | RRA1 | Arabinosyltransferase RRA1 [Source:UniProtKB/Swiss-Prot;Acc:Q9C9Q6] |
| AT1G75125 | 0,69 | 1,71E-05 | - | - |
| AT1G75150 | -1,16 | 8,60E-50 | - | unknown protein; Ha. [Source:TAIR;Acc:AT1G75150] |
| AT1G75190 | 1,25 | 1,35E-03 | - | AT1G75190 protein [Source:UniProtKB/TrEMBL;Acc:Q9FRL0] |
| AT1G75240 | -4,81 | 3,40E-04 | ZHD5 | ZHD5 [Source:UniProtKB/TrEMBL;Acc:A0A178WFZ4] |
| AT1G75280 | 1,61 | 5,93E-62 | - | Isoflavone reductase homolog P3 [Source:UniProtKB/Swiss-Prot;Acc:P52577] |
| AT1G75290 | 1,88 | 2,19E-22 | - | NAD(P)-binding Rossmann-fold superfamily protein [Source:TAIR;Acc:AT1G75290] |
| AT1G75370 | 0,80 | 9,04E-38 | - | Sec14p-like phosphatidylinositol transfer family protein [Source:UniProtKB/TrEMBL;Acc:F4HZ25] |
| AT1G75380 | 1,28 | 8,73E-19 | BBD1 | Bifunctional nuclease 1 [Source:UniProtKB/Swiss-Prot;Acc:Q9FWS6] |
| AT1G75410 | 0,75 | 8,69E-03 | BLH3 | BEL1-like homeodomain protein 3 [Source:UniProtKB/Swiss-Prot;Acc:Q9FWS9] |
| AT1G75540 | -0,71 | 2,83E-54 | BBX21 | B-box zinc finger protein 21 [Source:UniProtKB/Swiss-Prot;Acc:Q9LQZ7] |
| AT1G75550 | -0,72 | 3,78E-04 | - | glycine-rich protein [Source:TAIR;Acc:AT1G75550] |
| AT1G75580 | 0,67 | 1,72E-04 | - | At1g75580 [Source:UniProtKB/TrEMBL;Acc:Q9LR00] |
| AT1G75590 | -0,72 | 2,51E-05 | - | SAUR-like auxin-responsive protein family [Source:UniProtKB/TrEMBL;Acc:F4HZ54] |
| AT1G75620 | -1,30 | 5,27E-07 | - | F10A5.18 [Source:UniProtKB/TrEMBL;Acc:Q9LR03] |
| AT1G75630 | -0,69 | 1,87E-57 | AVA-P4 | V-type proton ATPase proteolipid subunit [Source:UniProtKB/TrEMBL;Acc:F4HZ57] |
| AT1G75640 | -1,24 | 1,50E-37 | - | F10A5.16 [Source:UniProtKB/TrEMBL;Acc:Q9LR04] |
| AT1G75700 | 4,34 | 8,46E-03 | HVA22G | HVA22-like protein G [Source:TAIR;Acc:AT1G75700] |
| AT1G75717 | -3,65 | 1,15E-03 | - | F10A5.9 [Source:UniProtKB/TrEMBL;Acc:Q9LR11] |
| AT1G75750 | 1,44 | 2,81E-09 | GASA1 | GASA1 [Source:UniProtKB/TrEMBL;Acc:A0A178W4S5] |
| AT1G75770 | 0,81 | 1,49E-02 | - | At1g75770/F10A5_10 [Source:UniProtKB/TrEMBL;Acc:Q94A92] |
| AT1G75780 | -1,95 | 4,75E-33 | TUBB1 | Tubulin beta-1 chain [Source:UniProtKB/Swiss-Prot;Acc:P12411] |
| AT1G75810 | 1,30 | 1,21E-14 | - | At1g75810 [Source:UniProtKB/TrEMBL;Acc:Q9LQT3] |
| AT1G75860 | 0,86 | 3,51E-56 | - | unknown protein; BEST Arabidopsis thaliana protein match is: unknown protein (TAIR:AT1G20100.1); Ha. [Source:TAIR;Acc:AT1G75860] |
| AT1G75960 | 1,69 | 9,18E-05 | AAE8 | Probable acyl-activating enzyme 8 [Source:UniProtKB/Swiss-Prot;Acc:Q9LQS1] |
| AT1G75990 | -0,92 | 1,10E-52 | RPN3B | 26S proteasome non-ATPase regulatory subunit 3 homolog B [Source:UniProtKB/Swiss-Prot;Acc:Q9LQR8] |
| AT1G76020 | -0,67 | 1,84E-02 | - | At1g76020 [Source:UniProtKB/TrEMBL;Acc:A0JQ12] |
| AT1G76070 | 1,26 | 1,51E-12 | - | Uncharacterized protein At1g76070 [Source:UniProtKB/Swiss-Prot;Acc:Q9SGS5] |
| AT1G76080 | 3,81 | 4,99E-131 | CDSP32 | CDSP32 [Source:UniProtKB/TrEMBL;Acc:A0A178WHK1] |
| AT1G76100 | 2,75 | 3,32E-54 | PETE1 | plastocyanin 1 [Source:TAIR;Acc:AT1G76100] |
| AT1G76130 | 1,05 | 4,77E-09 | AMY2 | Probable alpha-amylase 2 [Source:UniProtKB/Swiss-Prot;Acc:Q8LFG1] |
| AT1G76150 | 0,72 | 3,22E-37 | ECH2 | Enoyl-CoA hydratase 2, peroxisomal [Source:UniProtKB/Swiss-Prot;Acc:Q8VYI3] |
| AT1G76190 | -3,66 | 1,08E-02 | - | SAUR-like auxin-responsive protein family [Source:UniProtKB/TrEMBL;Acc:Q9SGR4] |
| AT1G76200 | -1,37 | 6,16E-121 | - | NADH dehydrogenase [ubiquinone] 1 beta subcomplex subunit 2 [Source:UniProtKB/Swiss-Prot;Acc:Q8LDK3] |
| AT1G76210 | -2,47 | 4,40E-04 | - | At1g76210 [Source:UniProtKB/TrEMBL;Acc:Q9SGR3] |
| AT1G76240 | -3,24 | 6,39E-32 | - | At1g76240 [Source:UniProtKB/TrEMBL;Acc:Q501A3] |
| AT1G76260 | -0,70 | 1,66E-30 | DWA2 | WD repeat-containing protein DWA2 [Source:UniProtKB/Swiss-Prot;Acc:Q6NPN9] |
| AT1G76360 | 1,51 | 5,75E-08 | - | At1g76360 [Source:UniProtKB/TrEMBL;Acc:A4FVS9] |
| AT1G76400 | -0,85 | 6,42E-64 | OST1A | Dolichyl-diphosphooligosaccharide--protein glycosyltransferase subunit 1A [Source:UniProtKB/Swiss-Prot;Acc:Q9SFX3] |
| AT1G76450 | -0,81 | 3,08E-06 | PPD3 | PsbP domain-containing protein 3, chloroplastic [Source:UniProtKB/Swiss-Prot;Acc:Q9S720] |
| AT1G76480 | -1,15 | 4,70E-04 | - | unknown protein; BEST Arabidopsis thaliana protein match is: unknown protein (TAIR:AT1G20890.1); Ha. [Source:TAIR;Acc:AT1G76480] |
| AT1G76520 | 0,86 | 1,02E-53 | PILS3 | Protein PIN-LIKES 3 [Source:UniProtKB/Swiss-Prot;Acc:Q9C9K5] |
| AT1G76560 | 1,01 | 1,65E-17 | CP12-3 | CP12-3 [Source:UniProtKB/TrEMBL;Acc:A0A178WDK7] |
| AT1G76590 | 1,61 | 5,59E-17 | - | At1g76590 [Source:UniProtKB/TrEMBL;Acc:Q2HIW3] |
| AT1G76600 | 1,97 | 1,47E-108 | - | Poly polymerase [Source:UniProtKB/TrEMBL;Acc:Q9C9J8] |
| AT1G76620 | -0,94 | 7,58E-11 | - | At1g76620/F14G6_22 [Source:UniProtKB/TrEMBL;Acc:Q8W4R7] |
| AT1G76640 | -2,42 | 1,48E-09 | CML39 | Calcium-binding protein CML39 [Source:UniProtKB/Swiss-Prot;Acc:Q9SRE7] |
| AT1G76720 | 1,82 | 6,92E-95 | - | Eukaryotic translation initiation factor 2 (eIF-2) family protein [Source:UniProtKB/TrEMBL;Acc:Q9SRE1] |
| AT1G76770 | -1,64 | 4,16E-02 | - | HSP20-like chaperones superfamily protein [Source:UniProtKB/TrEMBL;Acc:Q9SRD6] |
| AT1G76780 | 0,64 | 5,59E-04 | - | HSP20-like chaperones superfamily protein [Source:TAIR;Acc:AT1G76780] |
| AT1G76800 | -6,01 | 7,78E-07 | - | Vacuolar iron transporter homolog 2 [Source:UniProtKB/Swiss-Prot;Acc:Q9SRD3] |
| AT1G76820 | 1,13 | 3,05E-09 | - | eukaryotic translation initiation factor 2 (eIF-2) family protein [Source:TAIR;Acc:AT1G76820] |
| AT1G76850 | -0,66 | 4,66E-55 | SEC5A | Exocyst complex component SEC5A [Source:UniProtKB/Swiss-Prot;Acc:Q8S3U9] |
| AT1G76870 | 1,05 | 1,58E-08 | - | Transcription factor [Source:UniProtKB/TrEMBL;Acc:Q9C6K4] |
| AT1G76878 | -0,83 | 1,61E-05 | - | other RNA [Source:TAIR;Acc:AT1G76878] |
| AT1G76890 | -1,25 | 3,05E-19 | GT-2 | Trihelix transcription factor GT-2 [Source:UniProtKB/Swiss-Prot;Acc:Q39117] |
| AT1G76910 | -2,73 | 2,15E-03 | - | unknown protein; Ha. [Source:TAIR;Acc:AT1G76910] |
| AT1G76970 | 0,75 | 3,52E-17 | TOL4 | TOM1-like protein 4 [Source:UniProtKB/Swiss-Prot;Acc:Q6NQK0] |
| AT1G77000 | 0,89 | 3,21E-14 | SKP2B | F-box protein SKP2B [Source:UniProtKB/Swiss-Prot;Acc:O49286] |
| AT1G77010 | 0,90 | 5,26E-07 | PCMP-E5 | Putative pentatricopeptide repeat-containing protein At1g77010, mitochondrial [Source:UniProtKB/Swiss-Prot;Acc:O49287] |
| AT1G77090 | 3,00 | 2,57E-02 | PPD4 | PsbP domain-containing protein 4, chloroplastic [Source:UniProtKB/Swiss-Prot;Acc:O49292] |
| AT1G77120 | 4,92 | 9,86E-92 | ADH1 | Alcohol dehydrogenase class-P [Source:UniProtKB/Swiss-Prot;Acc:P06525] |
| AT1G77200 | -1,79 | 3,78E-43 | ERF037 | Ethylene-responsive transcription factor ERF037 [Source:UniProtKB/Swiss-Prot;Acc:O80654] |
| AT1G77230 | 0,89 | 3,53E-20 | - | At1g77230 [Source:UniProtKB/TrEMBL;Acc:Q8VZL0] |
| AT1G77260 | -0,74 | 1,62E-53 | - | Probable methyltransferase PMT10 [Source:UniProtKB/Swiss-Prot;Acc:Q94KE1] |
| AT1G77270 | -1,77 | 3,12E-08 | - | Uncharacterized protein At1g77270 [Source:UniProtKB/TrEMBL;Acc:Q8VY02] |
| AT1G77280 | -0,87 | 6,12E-17 | - | Protein kinase protein with adenine nucleotide alpha hydrolases-like domain [Source:TAIR;Acc:AT1G77280] |
| AT1G77330 | -1,46 | 1,38E-111 | - | 1-aminocyclopropane-1-carboxylate oxidase 5 [Source:UniProtKB/Swiss-Prot;Acc:Q0WPW4] |
| AT1G77350 | -0,65 | 6,54E-52 | - | At1g77350/F2P24_6 [Source:UniProtKB/TrEMBL;Acc:Q9FVX3] |
| AT1G77360 | 0,86 | 1,06E-11 | - | Pentatricopeptide repeat-containing protein At1g77360, mitochondrial [Source:UniProtKB/Swiss-Prot;Acc:Q9FVX2] |
| AT1G77370 | 0,72 | 1,05E-23 | - | Glutaredoxin family protein [Source:TAIR;Acc:AT1G77370] |
| AT1G77380 | 2,23 | 6,14E-04 | AAP3 | ATAAP3 [Source:UniProtKB/TrEMBL;Acc:A0A178W3U1] |
| AT1G77450 | 0,60 | 1,77E-31 | NAC032 | NAC032 [Source:UniProtKB/TrEMBL;Acc:A0A178WMH1] |
| AT1G77500 | -0,69 | 1,80E-24 | - | DUF630 family protein, putative (DUF630 and DUF632) [Source:UniProtKB/TrEMBL;Acc:Q9CAQ5] |
| AT1G77510 | -0,88 | 3,19E-165 | PDIL1-2 | Protein disulfide isomerase-like 1-2 [Source:UniProtKB/Swiss-Prot;Acc:Q9SRG3] |
| AT1G77530 | 0,87 | 4,27E-05 | - | O-methyltransferase family protein [Source:UniProtKB/TrEMBL;Acc:Q9CAQ3] |
| AT1G77580 | -0,62 | 1,65E-21 | FPP1 | Filament-like plant protein 1 [Source:UniProtKB/Swiss-Prot;Acc:Q9CAP9] |
| AT1G77655 | 5,75 | 5,44E-13 | - | At1g77655 [Source:UniProtKB/TrEMBL;Acc:Q3ECB7] |
| AT1G77680 | -0,81 | 7,45E-55 | SOV | Inactive exonuclease DIS3L2 [Source:UniProtKB/Swiss-Prot;Acc:Q0WPN0] |
| AT1G77760 | 1,10 | 2,47E-10 | NIA1 | Nitrate reductase [Source:UniProtKB/TrEMBL;Acc:A0A178WBR8] |
| AT1G77770 | -0,61 | 3,00E-33 | - | At1g77770 [Source:UniProtKB/TrEMBL;Acc:Q84K51] |
| AT1G77790 | -5,17 | 1,89E-05 | - | Glycosyl hydrolase superfamily protein [Source:UniProtKB/TrEMBL;Acc:Q9CA15] |
| AT1G77830 | 7,02 | 4,53E-10 | - | F28K19.4 [Source:UniProtKB/TrEMBL;Acc:Q9SH17] |
| AT1G77840 | 0,97 | 6,17E-144 | - | Probable eukaryotic translation initiation factor 5-2 [Source:UniProtKB/Swiss-Prot;Acc:Q9S825] |
| AT1G77880 | 2,51 | 1,07E-27 | - | Putative F-box protein At1g77880 [Source:UniProtKB/Swiss-Prot;Acc:Q9SH13] |
| AT1G77885 | 1,06 | 1,07E-03 | - | Putative uncharacterized protein [Source:UniProtKB/TrEMBL;Acc:Q3E7C8] |
| AT1G77890 | 0,60 | 1,75E-16 | - | At1g77890 [Source:UniProtKB/TrEMBL;Acc:Q147F2] |
| AT1G77920 | 1,04 | 2,59E-59 | TGA7 | Transcription factor TGA7 [Source:UniProtKB/Swiss-Prot;Acc:Q93ZE2] |
| AT1G77940 | -1,01 | 3,45E-120 | RPL30B | 60S ribosomal protein L30-2 [Source:UniProtKB/Swiss-Prot;Acc:Q8VZ19] |
| AT1G77950 | 1,71 | 5,06E-04 | AGL67 | AGAMOUS-like 67 [Source:UniProtKB/TrEMBL;Acc:F4I8L6] |
| AT1G78000 | 1,55 | 3,37E-17 | SULTR1;2 | Sulfate transporter 1.2 [Source:UniProtKB/Swiss-Prot;Acc:Q9MAX3] |
| AT1G78010 | 0,72 | 5,12E-21 | - | At1g78010 [Source:UniProtKB/TrEMBL;Acc:Q66GQ1] |
| AT1G78030 | -1,29 | 3,95E-02 | - | unknown protein; BEST Arabidopsis thaliana protein match is: Protein of unknown function (duplicated DUF1399) (TAIR:AT4G37900.1); Ha. [Source:TAIR;Acc:AT1G78030] |
| AT1G78060 | -0,59 | 8,23E-18 | BXL7 | Probable beta-D-xylosidase 7 [Source:UniProtKB/Swiss-Prot;Acc:Q9SGZ5] |
| AT1G78070 | 1,68 | 2,78E-23 | - | At1g78070/F28K19_28 [Source:UniProtKB/TrEMBL;Acc:Q94JT6] |
| AT1G78080 | 1,86 | 6,97E-61 | RAP2-4 | Ethylene-responsive transcription factor RAP2-4 [Source:UniProtKB/Swiss-Prot;Acc:Q8H1E4] |
| AT1G78090 | -5,90 | 1,48E-22 | TPPB | Trehalose-phosphate phosphatase B [Source:UniProtKB/Swiss-Prot;Acc:Q9C9S4] |
| AT1G78120 | 0,72 | 6,67E-13 | TPR12 | TPR12 [Source:UniProtKB/TrEMBL;Acc:A0A178W863] |
| AT1G78140 | 0,84 | 7,34E-15 | - | Uncharacterized methyltransferase At1g78140, chloroplastic [Source:UniProtKB/Swiss-Prot;Acc:Q8LBV4] |
| AT1G78172 | -1,40 | 1,27E-06 | - | unknown protein; FUNCTIONS IN: molecular_function unknown; INVOLVED IN: biological_process unknown; LOCATED IN: cellular_component unknown; Ha. [Source:TAIR;Acc:AT1G78172] |
| AT1G78190 | 1,12 | 2,46E-41 | TRM112B | Multifunctional methyltransferase subunit TRM112 homolog B [Source:UniProtKB/Swiss-Prot;Acc:Q9C9R3] |
| AT1G78230 | -3,84 | 1,69E-04 | - | Outer arm dynein light chain 1 protein [Source:UniProtKB/TrEMBL;Acc:F4IA60] |
| AT1G78300 | -0,99 | 1,96E-100 | GRF2 | 14-3-3-like protein GF14 omega [Source:UniProtKB/Swiss-Prot;Acc:Q01525] |
| AT1G78370 | -4,55 | 3,63E-93 | GSTU20 | Glutathione S-transferase U20 [Source:UniProtKB/Swiss-Prot;Acc:Q8L7C9] |
| AT1G78420 | 1,17 | 3,17E-88 | DA2 | E3 ubiquitin-protein ligase DA2 [Source:UniProtKB/Swiss-Prot;Acc:Q93YV5] |
| AT1G78530 | -1,81 | 5,17E-06 | - | Receptor-like serine/threonine-protein kinase At1g78530 [Source:UniProtKB/Swiss-Prot;Acc:Q9SYM9] |
| AT1G78580 | -0,92 | 1,60E-57 | TPS1 | TPS1 [Source:UniProtKB/TrEMBL;Acc:A0A384L3B9] |
| AT1G78600 | 1,40 | 7,49E-181 | LZF1 | Light-regulated zinc finger protein 1 [Source:UniProtKB/TrEMBL;Acc:F4IBS4] |
| AT1G78630 | -0,91 | 3,41E-50 | RPL13 | 50S ribosomal protein L13, chloroplastic [Source:UniProtKB/Swiss-Prot;Acc:Q9SYL9] |
| AT1G78660 | -0,65 | 3,62E-34 | GGH1 | Gamma-glutamyl hydrolase 1 [Source:UniProtKB/Swiss-Prot;Acc:Q9SYL6] |
| AT1G78670 | -0,96 | 1,38E-34 | GGH3 | Probable gamma-glutamyl hydrolase 3 [Source:UniProtKB/Swiss-Prot;Acc:Q9ZV85] |
| AT1G78750 | 3,74 | 6,10E-46 | - | F-box/FBD/LRR-repeat protein At1g78750 [Source:UniProtKB/Swiss-Prot;Acc:Q9ZV93] |
| AT1G78780 | 1,40 | 4,00E-07 | - | pathogenesis-related family protein [Source:TAIR;Acc:AT1G78780] |
| AT1G78810 | -0,58 | 1,41E-13 | - | Uncharacterized protein At1g78810; F9K20.15 [Source:UniProtKB/TrEMBL;Acc:Q8W461] |
| AT1G78820 | -3,17 | 3,78E-50 | - | EP1-like glycoprotein 1 [Source:UniProtKB/Swiss-Prot;Acc:Q9ZVA1] |
| AT1G78830 | -0,91 | 5,33E-22 | - | EP1-like glycoprotein 2 [Source:UniProtKB/Swiss-Prot;Acc:Q9ZVA2] |
| AT1G78850 | -1,92 | 1,80E-58 | - | EP1-like glycoprotein 3 [Source:UniProtKB/Swiss-Prot;Acc:Q9ZVA4] |
| AT1G78860 | -2,98 | 3,53E-36 | - | EP1-like glycoprotein 4 [Source:UniProtKB/Swiss-Prot;Acc:Q9ZVA5] |
| AT1G78870 | -0,67 | 1,29E-34 | UBC35 | UBC35 [Source:UniProtKB/TrEMBL;Acc:A0A178WFI9] |
| AT1G78900 | -0,71 | 3,32E-89 | VHA-A | VHA-A [Source:UniProtKB/TrEMBL;Acc:A0A384LM33] |
| AT1G78915 | 1,46 | 8,31E-36 | - | Tetratricopeptide repeat (TPR)-like superfamily protein [Source:UniProtKB/TrEMBL;Acc:F4IBX5] |
| AT1G78930 | 1,47 | 2,02E-36 | - | Mitochondrial transcription termination factor family protein [Source:UniProtKB/TrEMBL;Acc:Q0WRV2] |
| AT1G79060 | -0,68 | 4,18E-21 | - | At1g79060 [Source:UniProtKB/TrEMBL;Acc:O64545] |
| AT1G79075 | -0,68 | 1,33E-07 | - | other RNA [Source:TAIR;Acc:AT1G79075] |
| AT1G79160 | 2,57 | 8,67E-08 | - | Filamentous hemagglutinin transporter [Source:UniProtKB/TrEMBL;Acc:Q84JL6] |
| AT1G79180 | -1,54 | 2,70E-15 | ATMYB63 | myb domain protein 63 [Source:TAIR;Acc:AT1G79180] |
| AT1G79220 | 1,02 | 1,43E-14 | - | Mitochondrial transcription termination factor family protein [Source:UniProtKB/TrEMBL;Acc:O64531] |
| AT1G79270 | 1,13 | 1,23E-139 | ECT8 | Evolutionarily conserved C-terminal region 8 [Source:UniProtKB/TrEMBL;Acc:Q9FPE7] |
| AT1G79310 | -3,00 | 1,72E-10 | AMC7 | Metacaspase-7 [Source:UniProtKB/Swiss-Prot;Acc:Q6XPT5] |
| AT1G79320 | -3,51 | 3,20E-03 | AMC6 | MCP2c [Source:UniProtKB/TrEMBL;Acc:A0A178W108] |
| AT1G79330 | -0,75 | 2,35E-06 | AMC5 | Metacaspase-5 [Source:UniProtKB/Swiss-Prot;Acc:O64518] |
| AT1G79340 | -1,46 | 2,07E-185 | AMC4 | Metacaspase-4 [Source:UniProtKB/Swiss-Prot;Acc:O64517] |
| AT1G79410 | -0,92 | 3,36E-33 | 38626 | Organic cation/carnitine transporter 5 [Source:UniProtKB/Swiss-Prot;Acc:Q9SAK7] |
| AT1G79430 | 0,82 | 3,53E-12 | APL | Myb family transcription factor APL [Source:UniProtKB/Swiss-Prot;Acc:Q9SAK5] |
| AT1G79490 | 0,87 | 2,34E-38 | EMB2217 | Pentatricopeptide repeat-containing protein At1g79490, mitochondrial [Source:UniProtKB/Swiss-Prot;Acc:Q9SAK0] |
| AT1G79500 | -0,77 | 1,19E-45 | KDSA1 | 2-dehydro-3-deoxyphosphooctonate aldolase 1 [Source:UniProtKB/Swiss-Prot;Acc:Q9AV97] |
| AT1G79600 | 1,04 | 2,75E-54 | ABC1K3 | ABC1K3 [Source:UniProtKB/TrEMBL;Acc:A0A178WNJ7] |
| AT1G79620 | 0,84 | 1,35E-08 | - | Leucine-rich repeat protein kinase family protein [Source:TAIR;Acc:AT1G79620] |
| AT1G79640 | -0,67 | 4,87E-13 | - | Protein kinase superfamily protein [Source:TAIR;Acc:AT1G79640] |
| AT1G79650 | -0,81 | 1,68E-90 | RAD23 | Rad23 UV excision repair protein family [Source:UniProtKB/TrEMBL;Acc:F4IF85] |
| AT1G79720 | 0,95 | 1,48E-42 | - | At1g79720/F19K16_30 [Source:UniProtKB/TrEMBL;Acc:Q8RX60] |
| AT1G79780 | 0,67 | 2,73E-03 | - | CASP-like protein 3A2 [Source:UniProtKB/Swiss-Prot;Acc:Q1PFB8] |
| AT1G79860 | -3,77 | 7,39E-03 | ROPGEF12 | Rop guanine nucleotide exchange factor 12 [Source:UniProtKB/Swiss-Prot;Acc:Q9CA89] |
| AT1G79890 | 0,66 | 6,79E-13 | - | Putative helicase; 55525-51977 [Source:UniProtKB/TrEMBL;Acc:Q9CA92] |
| AT1G79930 | -0,94 | 4,40E-98 | HSP70-14 | Heat shock 70 kDa protein 14 [Source:UniProtKB/Swiss-Prot;Acc:Q9S7C0] |
| AT1G79990 | -1,02 | 1,53E-114 | - | Coatomer subunit beta' [Source:UniProtKB/TrEMBL;Acc:A0A178WGE9] |
| AT1G80050 | 2,24 | 6,10E-04 | APT2 | PHT1.1 [Source:UniProtKB/TrEMBL;Acc:A0A178W1K2] |
| AT1G80060 | 0,87 | 2,07E-11 | - | At1g80060 [Source:UniProtKB/TrEMBL;Acc:Q5XEN2] |
| AT1G80110 | -0,82 | 3,26E-05 | PP2B11 | F-box protein PP2-B11 [Source:UniProtKB/Swiss-Prot;Acc:Q949S5] |
| AT1G80150 | 0,78 | 7,16E-31 | - | Pentatricopeptide repeat-containing protein At1g80150, mitochondrial [Source:UniProtKB/Swiss-Prot;Acc:Q8GW57] |
| AT1G80170 | -0,64 | 7,30E-20 | - | Probable polygalacturonase At1g80170 [Source:UniProtKB/Swiss-Prot;Acc:Q94AJ5] |
| AT1G80190 | -0,74 | 4,11E-16 | PSF1 | partner of SLD five 1 [Source:TAIR;Acc:AT1G80190] |
| AT1G80230 | -0,71 | 3,97E-96 | COX5B-2 | Cytochrome c oxidase subunit 5b-2, mitochondrial [Source:UniProtKB/Swiss-Prot;Acc:Q9SSB8] |
| AT1G80245 | 1,18 | 6,98E-50 | - | At1g80245 [Source:UniProtKB/TrEMBL;Acc:Q0V7R8] |
| AT1G80280 | -1,11 | 1,93E-78 | - | Alpha/beta-Hydrolases superfamily protein [Source:UniProtKB/TrEMBL;Acc:Q9C976] |
| AT1G80340 | -4,32 | 1,36E-27 | GA3OX2 | Gibberellin 3-beta-dioxygenase 2 [Source:UniProtKB/Swiss-Prot;Acc:Q9ZT84] |
| AT1G80400 | 0,79 | 9,11E-18 | - | AT1G80400 protein [Source:UniProtKB/TrEMBL;Acc:Q9C965] |
| AT1G80460 | -0,77 | 6,00E-67 | GLPK | Glycerol kinase [Source:UniProtKB/Swiss-Prot;Acc:Q9M8L4] |
| AT1G80510 | -0,67 | 4,73E-18 | AVT6E | Amino acid transporter AVT6E [Source:UniProtKB/Swiss-Prot;Acc:Q9M8L9] |
| AT1G80520 | -0,95 | 2,44E-16 | - | Sterile alpha motif (SAM) domain-containing protein [Source:UniProtKB/TrEMBL;Acc:Q9M8M0] |
| AT1G80570 | 0,99 | 1,49E-08 | FBL14 | F-box/LRR-repeat protein 14 [Source:UniProtKB/Swiss-Prot;Acc:Q3EC97] |
| AT1G80610 | 0,79 | 6,03E-27 | - | At1g80610/T21F11_6 [Source:UniProtKB/TrEMBL;Acc:Q8VYI7] |
| AT1G80650 | -0,81 | 3,32E-02 | RTL1 | Ribonuclease 3-like protein 1 [Source:UniProtKB/Swiss-Prot;Acc:Q9M8N2] |
| AT1G80660 | 2,24 | 4,28E-02 | AHA9 | ATPase 9, plasma membrane-type [Source:UniProtKB/Swiss-Prot;Acc:Q42556] |
| AT1G80760 | -3,19 | 2,91E-03 | NIP6-1 | Aquaporin NIP6-1 [Source:UniProtKB/Swiss-Prot;Acc:Q9SAI4] |
| AT1G80840 | 0,78 | 9,39E-06 | WRKY40 | Probable WRKY transcription factor 40 [Source:UniProtKB/Swiss-Prot;Acc:Q9SAH7] |
| AT1G80890 | 0,70 | 1,99E-11 | - | At1g80890 [Source:UniProtKB/TrEMBL;Acc:Q9SAH1] |
| AT1G80920 | 0,72 | 1,98E-07 | ATJ8 | Chaperone protein dnaJ 8, chloroplastic [Source:UniProtKB/Swiss-Prot;Acc:Q9SAG8] |
| AT1G80960 | 0,82 | 1,00E-03 | - | F-box protein At1g80960 [Source:UniProtKB/Swiss-Prot;Acc:Q9SAG4] |
| AT2G01008 | 1,05 | 1,25E-03 | - | FUNCTIONS IN: molecular_function unknown; INVOLVED IN: biological_process unknown; LOCATED IN: cellular_component unknown; BEST Arabidopsis thaliana protein match is: maternal effect embryo arrest 38 (TAIR:AT3G43160.1). [Source:TAIR;Acc:AT2G01008] |
| AT2G01010 | 0,83 | 6,06E-17 | - | rRNA [Source:TAIR;Acc:AT2G01010] |
| AT2G01020 | 1,80 | 1,36E-92 | - | rRNA [Source:TAIR;Acc:AT2G01020] |
| AT2G01021 | 1,13 | 5,75E-05 | - | unknown protein; Ha. [Source:TAIR;Acc:AT2G01021] |
| AT2G01023 | 1,32 | 1,76E-03 | - | unknown protein; Ha. [Source:TAIR;Acc:AT2G01023] |
| AT2G01175 | 1,15 | 6,15E-05 | - | At2g01175 [Source:UniProtKB/TrEMBL;Acc:Q3EC92] |
| AT2G01250 | -0,85 | 6,51E-171 | RPL7B | 60S ribosomal protein L7-2 [Source:UniProtKB/Swiss-Prot;Acc:P60040] |
| AT2G01275 | 0,60 | 6,25E-07 | - | RING/FYVE/PHD zinc finger superfamily protein [Source:TAIR;Acc:AT2G01275] |
| AT2G01280 | 4,79 | 1,67E-04 | MEE65 | Cyclin/Brf1-like TBP-binding protein [Source:UniProtKB/TrEMBL;Acc:F4IN86] |
| AT2G01340 | 1,96 | 3,43E-22 | At17.1 | At2g01340 [Source:UniProtKB/TrEMBL;Acc:Q52K81] |
| AT2G01430 | 1,22 | 1,36E-05 | ATHB-17 | Homeobox-leucine zipper protein ATHB-17 [Source:UniProtKB/Swiss-Prot;Acc:Q8S9N6] |
| AT2G01540 | -0,86 | 1,34E-13 | CAR10 | Protein C2-DOMAIN ABA-RELATED 10 [Source:UniProtKB/Swiss-Prot;Acc:Q9ZVF1] |
| AT2G01660 | 1,02 | 4,12E-02 | CRRSP12 | Cysteine-rich repeat secretory protein 12 [Source:UniProtKB/Swiss-Prot;Acc:Q9ZU94] |
| AT2G01670 | -1,59 | 3,61E-10 | NUDT17 | Nudix hydrolase 17, mitochondrial [Source:UniProtKB/Swiss-Prot;Acc:Q9ZU95] |
| AT2G01720 | -0,74 | 4,26E-106 | OST1B | Dolichyl-diphosphooligosaccharide--protein glycosyltransferase subunit 1B [Source:UniProtKB/Swiss-Prot;Acc:Q9ZUA0] |
| AT2G01730 | 0,96 | 6,69E-19 | CPSF73-II | Cleavage and polyadenylation specificity factor subunit 3-II [Source:UniProtKB/Swiss-Prot;Acc:Q8GUU3] |
| AT2G01740 | 0,81 | 5,38E-09 | - | Pentatricopeptide repeat-containing protein At2g01740 [Source:UniProtKB/Swiss-Prot;Acc:Q9ZUA2] |
| AT2G01760 | 1,22 | 1,26E-09 | ARR14 | response regulator 14 [Source:TAIR;Acc:AT2G01760] |
| AT2G01770 | -2,43 | 2,52E-07 | VIT1 | Vacuolar iron transporter 1 [Source:UniProtKB/Swiss-Prot;Acc:Q9ZUA5] |
| AT2G01818 | -0,82 | 1,81E-10 | - | PLATZ transcription factor family protein [Source:UniProtKB/TrEMBL;Acc:A8MQN6] |
| AT2G01850 | 0,61 | 6,66E-32 | XTH27 | Probable xyloglucan endotransglucosylase/hydrolase protein 27 [Source:UniProtKB/Swiss-Prot;Acc:Q8LDS2] |
| AT2G01860 | 0,91 | 1,91E-03 | EMB975 | Pentatricopeptide repeat-containing protein At2g01860 [Source:UniProtKB/Swiss-Prot;Acc:Q5XET4] |
| AT2G01870 | 1,91 | 3,19E-02 | - | At2g01870 [Source:UniProtKB/TrEMBL;Acc:Q9SIS7] |
| AT2G01880 | -0,69 | 1,32E-07 | PAP7 | Purple acid phosphatase 7 [Source:UniProtKB/Swiss-Prot;Acc:Q8S341] |
| AT2G01970 | -0,83 | 6,81E-142 | TMN3 | Transmembrane 9 superfamily member 3 [Source:UniProtKB/Swiss-Prot;Acc:Q9ZPS7] |
| AT2G02000 | -1,79 | 1,34E-12 | GAD3 | Glutamate decarboxylase 3 [Source:UniProtKB/Swiss-Prot;Acc:Q9ZPS4] |
| AT2G02100 | 2,29 | 2,72E-22 | PDF2.2 | PDF2.2 [Source:UniProtKB/TrEMBL;Acc:A0A178VVN9] |
| AT2G02130 | -1,69 | 2,32E-36 | PDF2.3 | Defensin-like protein 1 [Source:UniProtKB/Swiss-Prot;Acc:Q9ZUL7] |
| AT2G02160 | 1,03 | 1,31E-185 | - | Zinc finger CCCH domain-containing protein 17 [Source:UniProtKB/Swiss-Prot;Acc:Q9ZUM0] |
| AT2G02220 | 0,77 | 5,04E-59 | PSKR1 | Phytosulfokine receptor 1 [Source:UniProtKB/Swiss-Prot;Acc:Q9ZVR7] |
| AT2G02360 | 0,93 | 1,38E-26 | PP2B10 | F-box protein PP2-B10 [Source:UniProtKB/Swiss-Prot;Acc:Q9ZVQ6] |
| AT2G02370 | 1,06 | 3,69E-25 | - | At2g02370/T16F16.16 [Source:UniProtKB/TrEMBL;Acc:Q9ZVQ5] |
| AT2G02380 | 1,59 | 5,68E-05 | GSTZ2 | Glutathione S-transferase Z2 [Source:UniProtKB/Swiss-Prot;Acc:Q9ZVQ4] |
| AT2G02390 | 0,63 | 4,15E-30 | ATGSTZ1 | glutathione S-transferase zeta 1 [Source:TAIR;Acc:AT2G02390] |
| AT2G02400 | -0,73 | 1,82E-16 | - | NAD(P)-binding Rossmann-fold superfamily protein [Source:UniProtKB/TrEMBL;Acc:Q9ZVQ2] |
| AT2G02498 | 3,94 | 2,35E-02 | - | Putative uncharacterized protein [Source:UniProtKB/TrEMBL;Acc:Q1G3S6] |
| AT2G02630 | 0,69 | 2,75E-22 | - | Cysteine/Histidine-rich C1 domain family protein [Source:UniProtKB/TrEMBL;Acc:F4IR90] |
| AT2G02710 | 0,87 | 1,07E-21 | TLP1 | Protein TWIN LOV 1 [Source:UniProtKB/Swiss-Prot;Acc:O64511] |
| AT2G02750 | 1,12 | 5,74E-09 | PCMP-E22 | Pentatricopeptide repeat-containing protein At2g02750 [Source:UniProtKB/Swiss-Prot;Acc:Q1PFA6] |
| AT2G02760 | 0,64 | 1,16E-40 | UBC2 | UBC2 [Source:UniProtKB/TrEMBL;Acc:A0A178W0U6] |
| AT2G02765 | 0,85 | 1,70E-25 | - | L-aminoadipate-semialdehyde dehydrogenase-phosphopantetheinyl transferase [Source:UniProtKB/TrEMBL;Acc:A0A1P8B2C9] |
| AT2G02780 | 0,59 | 1,22E-27 | - | Probable LRR receptor-like serine/threonine-protein kinase At2g02780 [Source:UniProtKB/Swiss-Prot;Acc:C0LGJ9] |
| AT2G02800 | 0,77 | 2,84E-57 | PBL3 | Probable serine/threonine-protein kinase PBL3 [Source:UniProtKB/Swiss-Prot;Acc:O49840] |
| AT2G02950 | -2,22 | 1,54E-88 | PKS1 | Protein PHYTOCHROME KINASE SUBSTRATE 1 [Source:UniProtKB/Swiss-Prot;Acc:Q9SWI1] |
| AT2G02955 | 0,91 | 2,74E-43 | MEE12 | TATA box-binding protein-associated factor RNA polymerase I subunit B [Source:UniProtKB/Swiss-Prot;Acc:Q5XVF0] |
| AT2G02990 | 2,82 | 2,61E-98 | RNS1 | Ribonuclease 1 [Source:UniProtKB/Swiss-Prot;Acc:P42813] |
| AT2G03020 | 6,71 | 8,62E-110 | - | Heat shock protein HSP20/alpha crystallin family [Source:UniProtKB/TrEMBL;Acc:Q84X24] |
| AT2G03090 | -1,57 | 8,79E-126 | EXPA15 | Expansin-A15 [Source:UniProtKB/Swiss-Prot;Acc:O80622] |
| AT2G03120 | -0,84 | 2,11E-141 | SPP | Signal peptide peptidase [Source:UniProtKB/Swiss-Prot;Acc:O81062] |
| AT2G03130 | 8,06 | 2,11E-104 | - | 50S ribosomal protein L7/L12 [Source:UniProtKB/TrEMBL;Acc:O81061] |
| AT2G03220 | 0,60 | 2,27E-40 | FUT1 | Glycosyltransferase (Fragment) [Source:UniProtKB/TrEMBL;Acc:W8PV36] |
| AT2G03260 | 0,67 | 2,59E-06 | PHO1-H2 | Phosphate transporter PHO1 homolog 2 [Source:UniProtKB/Swiss-Prot;Acc:Q6R8G8] |
| AT2G03350 | -0,82 | 3,72E-39 | - | At2g03350 [Source:UniProtKB/TrEMBL;Acc:Q9ZQ71] |
| AT2G03360 | 2,88 | 1,89E-18 | - | Glycosyltransferase family 61 protein [Source:UniProtKB/TrEMBL;Acc:F4ISB4] |
| AT2G03370 | 1,16 | 1,43E-07 | - | Glycosyltransferase (Fragment) [Source:UniProtKB/TrEMBL;Acc:W8Q3I2] |
| AT2G03380 | 0,82 | 6,12E-06 | PCMP-E47 | Pentatricopeptide repeat-containing protein At2g03380, mitochondrial [Source:UniProtKB/Swiss-Prot;Acc:Q9ZQ74] |
| AT2G03430 | -0,62 | 1,67E-15 | - | Ankyrin repeat family protein [Source:UniProtKB/TrEMBL;Acc:Q8GZ22] |
| AT2G03440 | -1,08 | 6,74E-22 | NRP1 | Nodulin-related protein 1 [Source:UniProtKB/Swiss-Prot;Acc:Q9ZQ80] |
| AT2G03480 | -1,03 | 1,70E-55 | QUL2 | Probable methyltransferase PMT5 [Source:UniProtKB/Swiss-Prot;Acc:Q3EC77] |
| AT2G03600 | -2,42 | 1,25E-12 | ATUPS3 | Ureide permease 3 [Source:UniProtKB/TrEMBL;Acc:F4IT87] |
| AT2G03620 | -0,59 | 2,48E-07 | MRS2-5 | Magnesium transporter MRS2-5 [Source:UniProtKB/Swiss-Prot;Acc:Q9ZPR4] |
| AT2G03720 | -6,18 | 6,08E-07 | MRH6 | MRH6 [Source:UniProtKB/TrEMBL;Acc:A0A384KF39] |
| AT2G03730 | 0,91 | 1,02E-26 | ACR5 | ACT domain-containing protein ACR5 [Source:UniProtKB/Swiss-Prot;Acc:Q9ZPQ8] |
| AT2G03750 | 4,80 | 3,45E-04 | SOT11 | Sulfotransferase [Source:UniProtKB/TrEMBL;Acc:A0A178VL58] |
| AT2G03760 | 3,67 | 0,00E+00 | SOT12 | Cytosolic sulfotransferase 12 [Source:UniProtKB/Swiss-Prot;Acc:P52839] |
| AT2G03770 | 3,14 | 1,04E-19 | SOT13 | Cytosolic sulfotransferase 13 [Source:UniProtKB/Swiss-Prot;Acc:Q9ZPQ5] |
| AT2G03870 | -0,62 | 4,37E-32 | LSM7 | LSM7 [Source:UniProtKB/TrEMBL;Acc:A0A178VPR3] |
| AT2G03980 | -1,88 | 6,84E-11 | - | GDSL esterase/lipase At2g03980 [Source:UniProtKB/Swiss-Prot;Acc:Q9SIF5] |
| AT2G04030 | 0,67 | 2,24E-114 | HSP90-5 | HSP90.5 [Source:UniProtKB/TrEMBL;Acc:A0A178VPV7] |
| AT2G04032 | 4,40 | 1,18E-03 | ZIP7 | Zinc transporter 7 [Source:UniProtKB/Swiss-Prot;Acc:Q8W246] |
| AT2G04039 | 4,40 | 3,12E-04 | - | unknown protein; FUNCTIONS IN: molecular_function unknown; LOCATED IN: chloroplast; EXPRESSED IN: 21 plant structures; EXPRESSED DURING: 13 growth stages; CONTAINS InterPro DOMAIN/s: Protein of unknown function DUF2996 (InterPro:IPR021374); Has 159 /.../hits to 159 proteins in 52 species: Archae - 0; Bacteria - 76; Metazoa - 0; Fungi - 0; Plants - 38; Viruses - 0; Other Eukaryotes - 45 (source: NCBI BLink). [Source:TAIR;Acc:AT2G04039] |
| AT2G04040 | 1,30 | 2,08E-90 | DTX1 | Protein DETOXIFICATION [Source:UniProtKB/TrEMBL;Acc:A0A178VPK7] |
| AT2G04050 | 8,53 | 6,80E-122 | DTX3 | Protein DETOXIFICATION 3 [Source:UniProtKB/Swiss-Prot;Acc:Q9SIA4] |
| AT2G04070 | 4,85 | 6,89E-04 | DTX4 | Protein DETOXIFICATION 4 [Source:UniProtKB/Swiss-Prot;Acc:Q9SIA3] |
| AT2G04100 | -0,69 | 2,00E-27 | DTX6 | Protein DETOXIFICATION 6 [Source:UniProtKB/Swiss-Prot;Acc:Q8RWF5] |
| AT2G04170 | -1,91 | 3,81E-162 | - | TRAF-like family protein [Source:UniProtKB/TrEMBL;Acc:Q6RF45] |
| AT2G04190 | 3,85 | 7,57E-03 | - | TRAF-like family protein [Source:UniProtKB/TrEMBL;Acc:F4IV59] |
| AT2G04230 | 0,91 | 4,32E-03 | - | F-box/FBD/LRR-repeat protein At2g04230 [Source:UniProtKB/Swiss-Prot;Acc:Q6NKX3] |
| AT2G04350 | 0,64 | 1,30E-19 | LACS8 | Long chain acyl-CoA synthetase 8 [Source:UniProtKB/Swiss-Prot;Acc:Q9SJD4] |
| AT2G04390 | -1,01 | 7,75E-88 | RPS17A | 40S ribosomal protein S17-1 [Source:UniProtKB/Swiss-Prot;Acc:P49205] |
| AT2G04400 | -0,91 | 2,35E-69 | IGPS | Indole-3-glycerol phosphate synthase, chloroplastic [Source:UniProtKB/Swiss-Prot;Acc:P49572] |
| AT2G04500 | -1,59 | 2,51E-40 | - | Cysteine/Histidine-rich C1 domain family protein [Source:UniProtKB/TrEMBL;Acc:Q6DR61] |
| AT2G04520 | -0,71 | 2,19E-57 | - | Nucleic acid-binding, OB-fold-like protein [Source:UniProtKB/TrEMBL;Acc:Q9SJB9] |
| AT2G04530 | 1,16 | 4,70E-38 | TRZ2 | tRNase Z TRZ2, chloroplastic [Source:UniProtKB/Swiss-Prot;Acc:Q8L633] |
| AT2G04650 | -0,95 | 1,82E-44 | - | ADP-glucose pyrophosphorylase family protein [Source:UniProtKB/TrEMBL;Acc:F4IFA4] |
| AT2G04680 | -0,61 | 7,00E-04 | - | Cysteine/Histidine-rich C1 domain family protein [Source:UniProtKB/TrEMBL;Acc:Q9SJ91] |
| AT2G04690 | 0,80 | 9,09E-11 | - | Pyridoxamine 5'-phosphate oxidase family protein [Source:TAIR;Acc:AT2G04690] |
| AT2G04780 | -1,39 | 2,05E-118 | FLA7 | Fasciclin-like arabinogalactan protein 7 [Source:UniProtKB/Swiss-Prot;Acc:Q9SJ81] |
| AT2G04800 | -3,52 | 9,33E-57 | - | At2g04800 [Source:UniProtKB/TrEMBL;Acc:Q9SJ79] |
| AT2G04852 | 1,02 | 2,28E-06 | - | Potential natural antisense gene, locus overlaps with AT2G04850 [Source:TAIR;Acc:AT2G04852] |
| AT2G04860 | 0,74 | 1,42E-08 | PCMP-E74 | Pentatricopeptide repeat-containing protein At2g04860 [Source:UniProtKB/Swiss-Prot;Acc:Q9SJ73] |
| AT2G05035 | 4,59 | 1,76E-42 | - | - |
| AT2G05045 | 4,26 | 1,32E-97 | - | - |
| AT2G05100 | -5,63 | 1,33E-05 | LHCB2.1 | photosystem II light harvesting complex gene 2.1 [Source:TAIR;Acc:AT2G05100] |
| AT2G05160 | -3,59 | 2,98E-14 | - | Zinc finger CCCH domain-containing protein 18 [Source:UniProtKB/Swiss-Prot;Acc:Q9SJ41] |
| AT2G05185 | 0,68 | 5,02E-04 | - | At2g05185 [Source:UniProtKB/TrEMBL;Acc:Q3EC64] |
| AT2G05220 | -0,65 | 2,26E-59 | RPS17B | 40S ribosomal protein S17-2 [Source:UniProtKB/Swiss-Prot;Acc:Q9SJ36] |
| AT2G05330 | 1,12 | 1,15E-04 | - | Putative BTB/POZ domain-containing protein At2g05330 [Source:UniProtKB/Swiss-Prot;Acc:Q9SJ29] |
| AT2G05350 | 0,82 | 1,34E-02 | - | Uncharacterized protein At2g05350 [Source:UniProtKB/TrEMBL;Acc:Q9SHT9] |
| AT2G05365 | 4,19 | 1,26E-03 | - | - |
| AT2G05380 | -3,27 | 2,00E-10 | GRP3S | glycine-rich protein 3 short isoform [Source:TAIR;Acc:AT2G05380] |
| AT2G05410 | 4,15 | 1,70E-02 | - | MATH domain and coiled-coil domain-containing protein At2g05410 [Source:UniProtKB/Swiss-Prot;Acc:Q9SHT3] |
| AT2G05420 | 9,40 | 8,35E-19 | - | MATH domain and coiled-coil domain-containing protein At2g05420 [Source:UniProtKB/Swiss-Prot;Acc:Q9SHT2] |
| AT2G05505 | 3,46 | 1,78E-10 | - | - |
| AT2G05515 | 3,54 | 9,05E-05 | - | - |
| AT2G05518 | 2,11 | 4,52E-03 | - | Unknown gene [Source:TAIR;Acc:AT2G05518] |
| AT2G05555 | 6,39 | 5,14E-08 | - | - |
| AT2G05620 | 0,71 | 1,42E-04 | PGR5 | Protein PROTON GRADIENT REGULATION 5, chloroplastic [Source:UniProtKB/Swiss-Prot;Acc:Q9SL05] |
| AT2G05630 | 1,34 | 1,37E-45 | ATG8D | Autophagy-related protein [Source:UniProtKB/TrEMBL;Acc:F4IHC1] |
| AT2G05632 | 0,97 | 4,15E-18 | - | Putative uncharacterized protein [Source:UniProtKB/TrEMBL;Acc:Q0WU16] |
| AT2G05753 | -2,69 | 4,51E-02 | - | unknown protein; FUNCTIONS IN: molecular_function unknown; INVOLVED IN: biological_process unknown; LOCATED IN: cellular_component unknown; Ha. [Source:TAIR;Acc:AT2G05753] |
| AT2G05810 | -0,75 | 5,67E-03 | - | ARM repeat superfamily protein [Source:UniProtKB/TrEMBL;Acc:Q8S8G1] |
| AT2G05825 | -1,61 | 1,53E-02 | - | - |
| AT2G05940 | 2,44 | 8,26E-32 | RIPK | Serine/threonine-protein kinase RIPK [Source:UniProtKB/Swiss-Prot;Acc:Q9ZUF4] |
| AT2G05990 | -0,72 | 1,08E-52 | MOD1 | Enoyl-[acyl-carrier-protein] reductase [NADH], chloroplastic [Source:UniProtKB/Swiss-Prot;Acc:Q9SLA8] |
| AT2G06040 | 1,00 | 3,10E-43 | - | CONTAINS InterPro DOMAIN/s: Leucine-rich repeat, cysteine-containing subtype (InterPro:IPR006553); BEST Arabidopsis thaliana protein match is: RNI-like superfamily protein (TAIR:AT5G21900.1); Ha. [Source:TAIR;Acc:AT2G06040] |
| AT2G06200 | -1,41 | 1,01E-35 | AtGRF6 | growth-regulating factor 6 [Source:TAIR;Acc:AT2G06200] |
| AT2G06255 | 1,13 | 1,53E-11 | EFL3 | Protein ELF4-LIKE 3 [Source:UniProtKB/Swiss-Prot;Acc:Q8S8F5] |
| AT2G06345 | 1,76 | 7,71E-06 | - | - |
| AT2G06520 | -0,61 | 6,22E-10 | PSBX | PSBX [Source:UniProtKB/TrEMBL;Acc:A0A178W0Y5] |
| AT2G06925 | 0,85 | 1,73E-13 | PLA2-ALPHA | PLA2-ALPHA [Source:UniProtKB/TrEMBL;Acc:A0A178VVH8] |
| AT2G07042 | 2,14 | 1,73E-06 | - | other RNA [Source:TAIR;Acc:AT2G07042] |
| AT2G07045 | 4,18 | 1,06E-02 | - | - |
| AT2G07050 | -1,22 | 2,97E-89 | CAS1 | Cycloartenol synthase [Source:UniProtKB/Swiss-Prot;Acc:P38605] |
| AT2G07180 | 0,86 | 2,35E-31 | PBL17 | Probable serine/threonine-protein kinase PBL17 [Source:UniProtKB/Swiss-Prot;Acc:Q8H1E3] |
| AT2G07340 | -0,65 | 1,61E-23 | PFD1 | At2g07350/T13E11.12 [Source:UniProtKB/TrEMBL;Acc:Q94AF7] |
| AT2G07605 | -0,76 | 8,03E-03 | - | - |
| AT2G07629 | 2,05 | 1,32E-03 | - | - |
| AT2G07638 | 3,33 | 2,58E-02 | - | - |
| AT2G07652 | 4,95 | 4,87E-04 | - | - |
| AT2G07655 | 1,96 | 4,69E-02 | - | - |
| AT2G07662 | 2,27 | 1,60E-04 | - | - |
| AT2G07665 | 2,77 | 3,55E-02 | - | - |
| AT2G07667 | 4,75 | 1,43E-03 | - | unknown protein; FUNCTIONS IN: molecular_function unknown; INVOLVED IN: biological_process unknown; LOCATED IN: mitochondrion; BEST Arabidopsis thaliana protein match is: unknown protein (TAIR:ATMG01180.1); Ha. [Source:TAIR;Acc:AT2G07667] |
| AT2G07695 | 4,41 | 4,50E-03 | - | Cytochrome C oxidase subunit II-like, transmembrane domain [Source:TAIR;Acc:AT2G07695] |
| AT2G07698 | 2,20 | 2,29E-08 | - | ATPase, F1 complex, alpha subunit protein [Source:UniProtKB/TrEMBL;Acc:F4IMB5] |
| AT2G07722 | 4,42 | 1,02E-02 | - | unknown protein; BEST Arabidopsis thaliana protein match is: unknown protein (TAIR:ATMG00620.1); Ha. [Source:TAIR;Acc:AT2G07722] |
| AT2G07725 | 4,77 | 7,30E-04 | - | At2g07725 [Source:UniProtKB/TrEMBL;Acc:Q6NMS1] |
| AT2G07739 | 3,74 | 6,53E-03 | - | Ycf1 protein [Source:TAIR;Acc:AT2G07739] |
| AT2G07798 | 3,06 | 3,57E-02 | - | unknown protein; FUNCTIONS IN: molecular_function unknown; INVOLVED IN: biological_process unknown; LOCATED IN: cellular_component unknown; BEST Arabidopsis thaliana protein match is: unknown protein (TAIR:ATMG01220.1); Ha. [Source:TAIR;Acc:AT2G07798] |
| AT2G08035 | 3,00 | 1,32E-04 | - | - |
| AT2G08095 | 9,16 | 8,07E-18 | - | - |
| AT2G08350 | 1,04 | 1,22E-03 | - | - |
| AT2G08440 | 1,88 | 4,09E-02 | - | - |
| AT2G08450 | 1,90 | 1,69E-02 | - | - |
| AT2G08530 | -3,10 | 4,20E-02 | - | - |
| AT2G08585 | 1,27 | 1,67E-02 | - | - |
| AT2G08610 | -1,75 | 2,36E-02 | - | - |
| AT2G08650 | 1,04 | 3,51E-12 | - | - |
| AT2G08755 | 1,08 | 2,38E-08 | - | - |
| AT2G08760 | 1,10 | 2,41E-35 | - | - |
| AT2G08865 | 0,69 | 6,74E-03 | - | - |
| AT2G09225 | -1,51 | 3,47E-06 | - | - |
| AT2G09495 | 1,04 | 2,46E-02 | - | - |
| AT2G09525 | 4,74 | 1,12E-06 | - | - |
| AT2G09710 | -1,38 | 3,02E-07 | - | - |
| AT2G09925 | -3,94 | 2,99E-03 | - | - |
| AT2G10440 | 1,66 | 1,72E-07 | MED15C | Probable mediator of RNA polymerase II transcription subunit 15c [Source:UniProtKB/Swiss-Prot;Acc:Q9SHV7] |
| AT2G10450 | 1,58 | 3,28E-02 | - | 14-3-3 family protein [Source:UniProtKB/TrEMBL;Acc:Q9SHV6] |
| AT2G10537 | -2,63 | 1,98E-02 | - | other RNA [Source:TAIR;Acc:AT2G10537] |
| AT2G10606 | -3,17 | 3,51E-02 | MIR396A | MIR396A; miRNA [Source:TAIR;Acc:AT2G10606] |
| AT2G10930 | 1,46 | 9,45E-12 | - | Transmembrane protein [Source:UniProtKB/TrEMBL;Acc:Q9SHW4] |
| AT2G10940 | -5,94 | 8,13E-06 | - | At2g10940/F15K19.1 [Source:UniProtKB/TrEMBL;Acc:Q9SKI0] |
| AT2G11270 | 1,40 | 8,52E-04 | - | Citrate synthase-like protein [Source:UniProtKB/TrEMBL;Acc:Q8RV72] |
| AT2G11520 | 0,66 | 1,66E-24 | CRCK3 | Calmodulin-binding receptor-like cytoplasmic kinase 3 [Source:UniProtKB/Swiss-Prot;Acc:Q9ASQ5] |
| AT2G12170 | 1,25 | 1,37E-05 | - | BEST Arabidopsis thaliana protein match is: Transcriptional factor B3 family protein (TAIR:AT2G24645.1); Ha. [Source:TAIR;Acc:AT2G12170] |
| AT2G12462 | -0,98 | 1,39E-02 | - | Sterile alpha motif (SAM) domain protein [Source:UniProtKB/TrEMBL;Acc:Q1G3Q5] |
| AT2G13290 | 0,76 | 1,70E-12 | - | Beta-1,4-N-acetylglucosaminyltransferase family protein [Source:UniProtKB/TrEMBL;Acc:Q9SKF0] |
| AT2G13360 | -1,23 | 6,31E-04 | AGT1 | Serine--glyoxylate aminotransferase [Source:UniProtKB/Swiss-Prot;Acc:Q56YA5] |
| AT2G13610 | 1,27 | 1,61E-14 | ABCG5 | ABC transporter G family member 5 [Source:UniProtKB/Swiss-Prot;Acc:Q9SIT6] |
| AT2G13650 | -0,72 | 5,06E-22 | GONST1 | GDP-mannose transporter (Fragment) [Source:UniProtKB/TrEMBL;Acc:A0A1B0VP09] |
| AT2G13800 | 0,59 | 6,11E-09 | SERK5 | Somatic embryogenesis receptor kinase 5 [Source:UniProtKB/Swiss-Prot;Acc:Q8LPS5] |
| AT2G13820 | -0,71 | 1,02E-23 | - | Bifunctional inhibitor/lipid-transfer protein/seed storage 2S albumin superfamily protein [Source:TAIR;Acc:AT2G13820] |
| AT2G13840 | 0,63 | 2,27E-14 | - | Expressed protein [Source:UniProtKB/TrEMBL;Acc:Q9ZQI9] |
| AT2G13900 | -2,10 | 4,58E-06 | - | Cysteine/Histidine-rich C1 domain family protein [Source:UniProtKB/TrEMBL;Acc:Q8S8J8] |
| AT2G13960 | 1,14 | 4,02E-09 | - | At2g13960 [Source:UniProtKB/TrEMBL;Acc:Q29PZ8] |
| AT2G14070 | 8,53 | 2,61E-15 | - | Wound-responsive protein-like protein [Source:UniProtKB/TrEMBL;Acc:Q9ZPT2] |
| AT2G14260 | 0,84 | 1,40E-33 | PIP | Proline iminopeptidase [Source:UniProtKB/Swiss-Prot;Acc:P93732] |
| AT2G14460 | -1,01 | 3,88E-38 | - | At2g14460 [Source:UniProtKB/TrEMBL;Acc:Q9ZQQ9] |
| AT2G14520 | 0,79 | 5,04E-25 | CBSDUF3 | DUF21 domain-containing protein At2g14520 [Source:UniProtKB/Swiss-Prot;Acc:Q9ZQR4] |
| AT2G14775 | 0,98 | 3,57E-05 | - | - |
| AT2G14800 | 1,17 | 5,00E-05 | - | unknown protein; BEST Arabidopsis thaliana protein match is: unknown protein (TAIR:AT3G44713.1); Ha. [Source:TAIR;Acc:AT2G14800] |
| AT2G14820 | -1,07 | 3,41E-50 | NPY2 | BTB/POZ domain-containing protein NPY2 [Source:UniProtKB/Swiss-Prot;Acc:O80970] |
| AT2G14835 | -0,61 | 7,22E-53 | - | Expressed protein [Source:UniProtKB/TrEMBL;Acc:Q944Q6] |
| AT2G14878 | 1,76 | 7,68E-182 | - | - |
| AT2G14880 | 0,98 | 4,55E-25 | - | Expressed protein [Source:UniProtKB/TrEMBL;Acc:O82326] |
| AT2G14890 | -1,14 | 4,52E-117 | AGP9 | AGP9 [Source:UniProtKB/TrEMBL;Acc:A0A178VSX2] |
| AT2G14910 | 1,76 | 2,68E-194 | - | MAR-binding filament-like protein [Source:UniProtKB/TrEMBL;Acc:O82329] |
| AT2G14920 | -1,56 | 5,93E-13 | SOT10 | Cytosolic sulfotransferase 10 [Source:UniProtKB/Swiss-Prot;Acc:O82330] |
| AT2G14960 | -0,90 | 8,90E-07 | GH3.1 | Probable indole-3-acetic acid-amido synthetase GH3.1 [Source:UniProtKB/Swiss-Prot;Acc:O82333] |
| AT2G15020 | -4,83 | 1,31E-04 | - | At2g15020 [Source:UniProtKB/TrEMBL;Acc:Q9ZUK9] |
| AT2G15029 | 2,68 | 1,48E-05 | - | unknown protein; Ha. [Source:TAIR;Acc:AT2G15029] |
| AT2G15128 | 0,75 | 1,80E-13 | - | other RNA [Source:TAIR;Acc:AT2G15128] |
| AT2G15130 | 1,29 | 1,40E-08 | - | Plant basic secretory protein (BSP) family protein [Source:UniProtKB/TrEMBL;Acc:Q9ZUJ8] |
| AT2G15280 | -0,68 | 1,98E-23 | RTNLB10 | Reticulon-like protein B10 [Source:UniProtKB/Swiss-Prot;Acc:Q6NPD8] |
| AT2G15300 | 0,77 | 1,56E-14 | - | Leucine-rich repeat protein kinase family protein [Source:UniProtKB/TrEMBL;Acc:Q9SHU6] |
| AT2G15310 | -0,60 | 3,90E-02 | ATARFB1A | Probable ADP-ribosylation factor At2g15310 [Source:UniProtKB/Swiss-Prot;Acc:Q9SHU5] |
| AT2G15350 | -4,69 | 1,14E-05 | FUT10 | Putative fucosyltransferase 10 [Source:UniProtKB/Swiss-Prot;Acc:Q9SJP6] |
| AT2G15370 | -4,29 | 6,42E-43 | FUT5 | Fucosyltransferase 5 (Fragment) [Source:UniProtKB/TrEMBL;Acc:A0A1W6AK11] |
| AT2G15390 | -1,42 | 1,30E-13 | FUT4 | Probable fucosyltransferase 4 [Source:UniProtKB/Swiss-Prot;Acc:Q9SJP2] |
| AT2G15400 | 0,66 | 3,33E-06 | NRPD3B | DNA-directed RNA polymerase II, third largest subunit [Source:UniProtKB/TrEMBL;Acc:Q0WRA4] |
| AT2G15580 | 1,07 | 3,45E-33 | - | AT2G15580 protein [Source:UniProtKB/TrEMBL;Acc:Q9ZQF5] |
| AT2G15630 | 0,68 | 7,05E-09 | - | Pentatricopeptide repeat-containing protein At2g15630, mitochondrial [Source:UniProtKB/Swiss-Prot;Acc:Q9ZQF1] |
| AT2G15760 | -1,27 | 9,62E-03 | - | At2g15760 [Source:UniProtKB/TrEMBL;Acc:Q9ZQD8] |
| AT2G15880 | 2,39 | 1,19E-13 | PEX3 | Pollen-specific leucine-rich repeat extensin-like protein 3 [Source:UniProtKB/Swiss-Prot;Acc:Q9XIL9] |
| AT2G15890 | 1,13 | 1,18E-09 | MEE14 | CCG-binding protein 1 [Source:UniProtKB/Swiss-Prot;Acc:Q9XIM0] |
| AT2G15970 | -1,12 | 4,05E-48 | COR413PM1 | WCOR413-like protein [Source:UniProtKB/TrEMBL;Acc:A0A178VY40] |
| AT2G16060 | 1,33 | 9,41E-64 | AHB1 | NSHB1 [Source:UniProtKB/TrEMBL;Acc:A0A384KL50] |
| AT2G16070 | 0,71 | 5,59E-11 | PDV2 | Plastid division protein PDV2 [Source:UniProtKB/Swiss-Prot;Acc:Q9XII1] |
| AT2G16230 | -1,58 | 1,53E-70 | - | O-Glycosyl hydrolases family 17 protein [Source:UniProtKB/TrEMBL;Acc:F4IKB3] |
| AT2G16270 | -0,89 | 5,10E-17 | - | Transmembrane protein [Source:UniProtKB/TrEMBL;Acc:Q9SIX2] |
| AT2G16280 | -0,63 | 5,49E-39 | KCS9 | 3-ketoacyl-CoA synthase 9 [Source:UniProtKB/Swiss-Prot;Acc:Q9SIX1] |
| AT2G16365 | 0,87 | 3,57E-49 | - | F-box protein At2g16365 [Source:UniProtKB/Swiss-Prot;Acc:Q84V03] |
| AT2G16450 | 3,72 | 3,46E-02 | - | F-box protein At2g16450 [Source:UniProtKB/Swiss-Prot;Acc:Q9SIV7] |
| AT2G16460 | -0,66 | 1,20E-25 | - | Coiled-coil 90B-like protein (DUF1640) [Source:UniProtKB/TrEMBL;Acc:Q9SIV6] |
| AT2G16500 | -0,92 | 1,73E-164 | ADC1 | Arginine decarboxylase 1 [Source:UniProtKB/Swiss-Prot;Acc:Q9SI64] |
| AT2G16510 | -1,39 | 3,46E-199 | VHA-c3 | V-type proton ATPase subunit c1 [Source:UniProtKB/Swiss-Prot;Acc:P0DH92] |
| AT2G16530 | -1,03 | 1,15E-23 | - | 3-oxo-5-alpha-steroid 4-dehydrogenase family protein [Source:TAIR;Acc:AT2G16530] |
| AT2G16570 | 0,73 | 1,05E-60 | ASE1 | Amidophosphoribosyltransferase 1, chloroplastic [Source:UniProtKB/Swiss-Prot;Acc:Q9SI61] |
| AT2G16575 | 0,89 | 2,77E-03 | - | unknown protein; BEST Arabidopsis thaliana protein match is: unknown protein (TAIR:AT1G17780.2); Ha. [Source:TAIR;Acc:AT2G16575] |
| AT2G16580 | -0,75 | 4,06E-20 | - | Putative auxin-induced protein [Source:UniProtKB/TrEMBL;Acc:Q9SI60] |
| AT2G16586 | 1,65 | 1,50E-04 | - | Transmembrane protein [Source:UniProtKB/TrEMBL;Acc:Q8GXM1] |
| AT2G16600 | -0,84 | 2,15E-72 | CYP19-1 | Peptidyl-prolyl cis-trans isomerase [Source:UniProtKB/TrEMBL;Acc:A0A178VVJ7] |
| AT2G16650 | 1,14 | 1,23E-156 | PRORP2 | Proteinaceous RNase P 2 [Source:UniProtKB/Swiss-Prot;Acc:Q680B9] |
| AT2G16700 | 0,61 | 3,19E-03 | ADF5 | ATADF5 [Source:UniProtKB/TrEMBL;Acc:A0A178VPY9] |
| AT2G16770 | 0,78 | 4,46E-19 | BZIP23 | Basic leucine zipper 23 [Source:UniProtKB/Swiss-Prot;Acc:Q8GTS2] |
| AT2G16780 | 0,65 | 1,97E-45 | MSI2 | WD-40 repeat-containing protein MSI2 [Source:UniProtKB/Swiss-Prot;Acc:O22468] |
| AT2G16870 | 0,62 | 2,49E-07 | - | Disease resistance protein (TIR-NBS-LRR class) family [Source:UniProtKB/TrEMBL;Acc:Q9ZVX6] |
| AT2G16890 | 1,92 | 1,82E-10 | UGT90A1 | UDP-glycosyltransferase 90A1 [Source:UniProtKB/Swiss-Prot;Acc:Q9ZVX4] |
| AT2G16900 | 0,97 | 7,56E-43 | - | Expressed protein [Source:UniProtKB/TrEMBL;Acc:Q9ZVX3] |
| AT2G16910 | 2,06 | 2,03E-03 | AMS | Transcription factor ABORTED MICROSPORES [Source:UniProtKB/Swiss-Prot;Acc:Q9ZVX2] |
| AT2G16980 | -2,30 | 1,81E-19 | - | Major facilitator superfamily protein [Source:TAIR;Acc:AT2G16980] |
| AT2G16990 | 1,88 | 5,59E-07 | - | Major facilitator superfamily protein [Source:UniProtKB/TrEMBL;Acc:F4IMD9] |
| AT2G17036 | 1,43 | 4,56E-06 | - | F-box protein At2g17036 [Source:UniProtKB/Swiss-Prot;Acc:Q6DR20] |
| AT2G17110 | 0,67 | 2,83E-15 | - | Protein of unknown function (DUF630 and DUF632) [Source:TAIR;Acc:AT2G17110] |
| AT2G17130 | -0,74 | 1,20E-44 | IDH2 | Isocitrate dehydrogenase [NAD] regulatory subunit 2, mitochondrial [Source:UniProtKB/Swiss-Prot;Acc:P93032] |
| AT2G17220 | 0,98 | 1,60E-05 | PIX13 | Probable serine/threonine-protein kinase PIX13 [Source:UniProtKB/Swiss-Prot;Acc:Q9SII6] |
| AT2G17260 | 0,58 | 8,03E-05 | GLR2 | glutamate receptor 2 [Source:TAIR;Acc:AT2G17260] |
| AT2G17290 | -0,69 | 2,47E-49 | CPK6 | Calcium-dependent protein kinase 6 [Source:UniProtKB/Swiss-Prot;Acc:Q38872] |
| AT2G17430 | 1,34 | 2,10E-12 | MLO7 | MLO-like protein 7 [Source:UniProtKB/Swiss-Prot;Acc:O22752] |
| AT2G17450 | -0,59 | 1,53E-12 | ATL44 | Probable E3 ubiquitin-protein ligase ATL44 [Source:UniProtKB/Swiss-Prot;Acc:O22755] |
| AT2G17520 | 0,89 | 1,06E-50 | IRE1A | Serine/threonine-protein kinase/endoribonuclease IRE1a [Source:UniProtKB/Swiss-Prot;Acc:Q9C5S2] |
| AT2G17525 | 0,66 | 5,10E-17 | - | Pentatricopeptide repeat-containing protein At2g17525, mitochondrial [Source:UniProtKB/Swiss-Prot;Acc:Q84VG6] |
| AT2G17560 | -1,11 | 2,61E-145 | HMGB4 | High mobility group B protein 4 [Source:UniProtKB/Swiss-Prot;Acc:Q42344] |
| AT2G17590 | -2,62 | 1,39E-21 | - | Cysteine/Histidine-rich C1 domain family protein [Source:UniProtKB/TrEMBL;Acc:Q84X54] |
| AT2G17600 | -3,69 | 2,98E-04 | - | Cysteine/Histidine-rich C1 domain family protein [Source:UniProtKB/TrEMBL;Acc:Q9SHK9] |
| AT2G17620 | -0,73 | 5,52E-09 | CYCB2-1 | Cyclin-B2-1 [Source:UniProtKB/Swiss-Prot;Acc:Q39068] |
| AT2G17630 | -1,48 | 6,13E-189 | PSAT2 | Phosphoserine aminotransferase 2, chloroplastic [Source:UniProtKB/Swiss-Prot;Acc:Q9SHP0] |
| AT2G17640 | 2,33 | 9,31E-48 | SAT2 | Serine acetyltransferase 2 [Source:UniProtKB/Swiss-Prot;Acc:Q8S895] |
| AT2G17660 | 1,67 | 1,98E-02 | - | At2g17660 [Source:UniProtKB/TrEMBL;Acc:Q9SEY4] |
| AT2G17695 | 1,13 | 9,79E-05 | - | FUNCTIONS IN: molecular_function unknown; INVOLVED IN: biological_process unknown; LOCATED IN: chloroplast; CONTAINS InterPro DOMAIN/s: Domain of unknown function DUF1990 (InterPro:IPR018960); Ha. [Source:TAIR;Acc:AT2G17695] |
| AT2G17705 | 0,78 | 6,95E-07 | - | Methionine-S-oxide reductase [Source:UniProtKB/TrEMBL;Acc:Q6RF46] |
| AT2G17710 | 0,82 | 8,94E-04 | - | At2g17710/T17A5.17 [Source:UniProtKB/TrEMBL;Acc:Q8W1E8] |
| AT2G17720 | -1,19 | 4,75E-102 | P4H5 | Prolyl 4-hydroxylase 5 [Source:UniProtKB/Swiss-Prot;Acc:Q24JN5] |
| AT2G17787 | 1,07 | 5,90E-18 | - | At2g17787 [Source:UniProtKB/TrEMBL;Acc:Q0WS99] |
| AT2G17830 | 1,10 | 2,70E-15 | - | At2g17830 [Source:UniProtKB/TrEMBL;Acc:Q058M6] |
| AT2G17890 | -2,32 | 3,74E-05 | CPK16 | Calcium-dependent protein kinase 16 [Source:UniProtKB/Swiss-Prot;Acc:Q7XJR9] |
| AT2G17940 | 4,84 | 9,82E-04 | - | WEB family protein At2g17940 [Source:UniProtKB/Swiss-Prot;Acc:O48822] |
| AT2G17980 | -0,68 | 1,38E-54 | SLY1 | SEC1 family transport protein SLY1 [Source:UniProtKB/Swiss-Prot;Acc:Q9SL48] |
| AT2G18020 | -0,71 | 8,43E-100 | RPL8A | EMB2296 [Source:UniProtKB/TrEMBL;Acc:A0A178VSS0] |
| AT2G18025 | 5,74 | 3,47E-06 | - | - |
| AT2G18040 | -0,69 | 2,35E-76 | PIN1 | Peptidyl-prolyl cis-trans isomerase Pin1 [Source:UniProtKB/Swiss-Prot;Acc:Q9SL42] |
| AT2G18050 | 5,11 | 7,51E-08 | HIS1-3 | HIS1-3 [Source:UniProtKB/TrEMBL;Acc:A0A178VX64] |
| AT2G18060 | 1,14 | 1,52E-08 | NAC037 | VND1 [Source:UniProtKB/TrEMBL;Acc:A0A178VM15] |
| AT2G18090 | 0,68 | 3,51E-58 | - | PHD finger family protein / SWIB complex BAF60b domain-containing protein / GYF domain-containing protein [Source:UniProtKB/TrEMBL;Acc:Q94JX1] |
| AT2G18160 | -0,70 | 1,62E-89 | BZIP2 | bZIP transcription factor 2 [Source:UniProtKB/Swiss-Prot;Acc:Q9SI15] |
| AT2G18170 | 0,64 | 1,13E-19 | MPK7 | Mitogen-activated protein kinase 7 [Source:UniProtKB/Swiss-Prot;Acc:Q39027] |
| AT2G18180 | 5,64 | 6,68E-06 | SFH10 | Phosphatidylinositol/phosphatidylcholine transfer protein SFH10 [Source:UniProtKB/Swiss-Prot;Acc:Q9SI13] |
| AT2G18190 | 6,66 | 2,20E-09 | - | AAA-ATPase At2g18190 [Source:UniProtKB/Swiss-Prot;Acc:F4IQG2] |
| AT2G18193 | 2,86 | 0,00E+00 | - | AAA-ATPase At2g18193 [Source:UniProtKB/Swiss-Prot;Acc:Q8GW96] |
| AT2G18280 | -1,51 | 6,99E-71 | TULP2 | Tubby-like F-box protein [Source:UniProtKB/TrEMBL;Acc:B9DGB6] |
| AT2G18290 | 0,89 | 4,39E-25 | APC10 | Anaphase-promoting complex subunit 10 [Source:UniProtKB/Swiss-Prot;Acc:Q9ZPW2] |
| AT2G18350 | -0,87 | 1,25E-03 | ZHD6 | Zinc-finger homeodomain protein 6 [Source:UniProtKB/Swiss-Prot;Acc:Q9ZPW7] |
| AT2G18390 | 1,00 | 1,43E-97 | ARL2 | TTN5 [Source:UniProtKB/TrEMBL;Acc:A0A178VQD7] |
| AT2G18400 | -0,68 | 2,09E-31 | - | Putative ribosomal protein L6 [Source:UniProtKB/TrEMBL;Acc:Q9ZPX2] |
| AT2G18440 | 1,98 | 4,19E-242 | GUT15 | GUT15 (GENE WITH UNSTABLE TRANSCRIPT 15); other RNA [Source:TAIR;Acc:AT2G18440] |
| AT2G18450 | -1,73 | 3,91E-60 | SDH1-2 | Succinate dehydrogenase [ubiquinone] flavoprotein subunit, mitochondrial [Source:UniProtKB/TrEMBL;Acc:A0A178VRF6] |
| AT2G18460 | -5,45 | 4,43E-05 | LCV3 | Protein LIKE COV 3 [Source:UniProtKB/Swiss-Prot;Acc:F4IQJ6] |
| AT2G18480 | 5,30 | 5,49E-05 | PLT3 | Probable polyol transporter 3 [Source:UniProtKB/Swiss-Prot;Acc:Q9ZNS0] |
| AT2G18500 | 1,85 | 3,91E-09 | OFP7 | Transcription repressor OFP7 [Source:UniProtKB/Swiss-Prot;Acc:Q9ZU65] |
| AT2G18600 | 0,90 | 1,10E-02 | RCE2 | Probable NEDD8-conjugating enzyme Ubc12-like [Source:UniProtKB/Swiss-Prot;Acc:Q9ZU75] |
| AT2G18620 | -3,00 | 2,94E-20 | - | Geranylgeranyl pyrophosphate synthase 7, chloroplastic [Source:UniProtKB/Swiss-Prot;Acc:Q9ZU77] |
| AT2G18670 | 2,14 | 1,58E-13 | ATL56 | RING-H2 finger protein ATL56 [Source:UniProtKB/Swiss-Prot;Acc:Q9ZV51] |
| AT2G18680 | 3,74 | 4,47E-05 | - | unknown protein; FUNCTIONS IN: molecular_function unknown; INVOLVED IN: biological_process unknown; LOCATED IN: endomembrane system; EXPRESSED IN: male gametophyte, pollen tube; EXPRESSED DURING: L mature pollen stage, M germinated pollen stage; BES /.../idopsis thaliana protein match is: unknown protein (TAIR:AT2G18690.1); Ha. [Source:TAIR;Acc:AT2G18680] |
| AT2G18700 | 0,80 | 1,97E-14 | TPS11 | Probable alpha,alpha-trehalose-phosphate synthase [UDP-forming] 11 [Source:UniProtKB/Swiss-Prot;Acc:Q9ZV48] |
| AT2G18720 | 3,53 | 7,58E-28 | - | Translation elongation factor EF1A/initiation factor IF2gamma family protein [Source:UniProtKB/TrEMBL;Acc:F4IRF5] |
| AT2G18735 | -0,61 | 1,82E-04 | - | other RNA [Source:TAIR;Acc:AT2G18735] |
| AT2G18740 | -0,68 | 3,01E-50 | - | Putative small nuclear ribonucleoprotein E [Source:UniProtKB/TrEMBL;Acc:Q9ZV45] |
| AT2G18800 | -1,31 | 4,20E-26 | XTH21 | Xyloglucan endotransglucosylase/hydrolase [Source:UniProtKB/TrEMBL;Acc:A0A178VQ29] |
| AT2G18840 | -0,95 | 1,25E-53 | - | Protein YIPF [Source:UniProtKB/TrEMBL;Acc:O64614] |
| AT2G18950 | 0,69 | 1,38E-10 | HPT1 | Homogentisate phytyltransferase 1, chloroplastic [Source:UniProtKB/Swiss-Prot;Acc:Q8VWJ1] |
| AT2G18970 | 2,02 | 1,41E-02 | - | At2g18970 [Source:UniProtKB/TrEMBL;Acc:O64627] |
| AT2G18990 | -0,65 | 3,62E-33 | TXND9 | Thioredoxin domain-containing protein 9 homolog [Source:UniProtKB/Swiss-Prot;Acc:O64628] |
| AT2G19050 | -1,58 | 2,44E-02 | - | GDSL esterase/lipase At2g19050 [Source:UniProtKB/Swiss-Prot;Acc:O64468] |
| AT2G19060 | -4,71 | 3,17E-03 | - | GDSL esterase/lipase At2g19060 [Source:UniProtKB/Swiss-Prot;Acc:O64469] |
| AT2G19130 | 3,18 | 4,13E-19 | - | G-type lectin S-receptor-like serine/threonine-protein kinase At2g19130 [Source:UniProtKB/Swiss-Prot;Acc:O64477] |
| AT2G19160 | -0,66 | 1,56E-28 | - | At2g19160 [Source:UniProtKB/TrEMBL;Acc:Q8W460] |
| AT2G19170 | -0,74 | 1,46E-29 | SBT2.5 | Subtilisin-like protease SBT2.5 [Source:UniProtKB/Swiss-Prot;Acc:O64481] |
| AT2G19180 | 0,59 | 8,36E-20 | - | Expressed protein [Source:UniProtKB/TrEMBL;Acc:O64482] |
| AT2G19190 | 4,08 | 3,59E-13 | SIRK | Senescence-induced receptor-like serine/threonine-protein kinase [Source:UniProtKB/Swiss-Prot;Acc:O64483] |
| AT2G19240 | 1,63 | 1,78E-19 | - | Ypt/Rab-GAP domain of gyp1p superfamily protein [Source:TAIR;Acc:AT2G19240] |
| AT2G19310 | 2,94 | 3,72E-160 | HSP18.5 | 18.5 kDa class IV heat shock protein [Source:UniProtKB/Swiss-Prot;Acc:O64564] |
| AT2G19330 | 1,84 | 3,81E-04 | PIRL6 | Plant intracellular Ras-group-related LRR protein 6 [Source:UniProtKB/Swiss-Prot;Acc:O64566] |
| AT2G19340 | 1,77 | 6,60E-04 | - | At2g19340 [Source:UniProtKB/TrEMBL;Acc:Q8VYT4] |
| AT2G19450 | 0,71 | 1,91E-45 | DGAT1 | Diacylglycerol O-acyltransferase 1 [Source:UniProtKB/Swiss-Prot;Acc:Q9SLD2] |
| AT2G19460 | 1,40 | 1,59E-124 | - | Protein of unknown function (DUF3511) [Source:TAIR;Acc:AT2G19460] |
| AT2G19530 | -0,93 | 1,59E-14 | - | Transmembrane protein [Source:UniProtKB/TrEMBL;Acc:Q9ZUN9] |
| AT2G19580 | -0,83 | 4,28E-04 | TET2 | Tetraspanin-2 [Source:UniProtKB/Swiss-Prot;Acc:Q9ZUN5] |
| AT2G19610 | 5,18 | 7,71E-08 | - | RBR-type E3 ubiquitin transferase [Source:UniProtKB/TrEMBL;Acc:Q84RJ8] |
| AT2G19640 | -0,80 | 1,96E-28 | ASHR2 | Histone-lysine N-methyltransferase ASHR2 [Source:UniProtKB/Swiss-Prot;Acc:Q9ZUM9] |
| AT2G19650 | 1,97 | 3,42E-02 | - | Cysteine/Histidine-rich C1 domain family protein [Source:UniProtKB/TrEMBL;Acc:Q9ZUM8] |
| AT2G19690 | -0,92 | 3,22E-15 | PLA2-BETA | Phospholipase A2-beta [Source:UniProtKB/TrEMBL;Acc:F4ITF6] |
| AT2G19730 | -0,85 | 1,51E-169 | RPL28A | 60S ribosomal protein L28-1 [Source:UniProtKB/Swiss-Prot;Acc:O82204] |
| AT2G19740 | -0,66 | 1,27E-68 | RPL31A | 60S ribosomal protein L31-1 [Source:UniProtKB/Swiss-Prot;Acc:Q9SLL7] |
| AT2G19750 | -0,58 | 5,16E-37 | RPS30A | 40S ribosomal protein S30 [Source:UniProtKB/Swiss-Prot;Acc:P49689] |
| AT2G19760 | -0,86 | 4,72E-42 | PRO1 | Profilin-1 [Source:UniProtKB/Swiss-Prot;Acc:Q42449] |
| AT2G19780 | -1,13 | 3,93E-05 | - | Leucine-rich repeat (LRR) family protein [Source:UniProtKB/TrEMBL;Acc:O82202] |
| AT2G19800 | 2,77 | 1,07E-188 | MIOX2 | At2g19800 [Source:UniProtKB/TrEMBL;Acc:B4F7Q2] |
| AT2G19810 | 0,72 | 5,95E-04 | - | TZF2 [Source:UniProtKB/TrEMBL;Acc:A0A178VX61] |
| AT2G19880 | -0,65 | 5,03E-38 | - | Nucleotide-diphospho-sugar transferases superfamily protein [Source:TAIR;Acc:AT2G19880] |
| AT2G19890 | 4,60 | 1,14E-10 | - | BEST Arabidopsis thaliana protein match is: Putative endonuclease or glycosyl hydrolase (TAIR:AT3G62210.1); Ha. [Source:TAIR;Acc:AT2G19890] |
| AT2G19900 | 4,33 | 2,65E-28 | NADP-ME1 | NADP-dependent malic enzyme 1 [Source:UniProtKB/Swiss-Prot;Acc:O82191] |
| AT2G19910 | 1,58 | 1,27E-04 | RDR3 | Probable RNA-dependent RNA polymerase 3 [Source:UniProtKB/Swiss-Prot;Acc:O82190] |
| AT2G19920 | -1,42 | 5,07E-04 | - | RNA-dependent RNA polymerase family protein [Source:TAIR;Acc:AT2G19920] |
| AT2G19950 | -0,59 | 8,16E-29 | GC1 | GC1 [Source:UniProtKB/TrEMBL;Acc:A0A178VUM2] |
| AT2G19970 | -2,78 | 2,14E-02 | - | At2g19970 [Source:UniProtKB/TrEMBL;Acc:Q9SL83] |
| AT2G20080 | -0,94 | 9,76E-10 | SPEAR1 | Protein SPEAR1 [Source:UniProtKB/Swiss-Prot;Acc:Q84X40] |
| AT2G20100 | -1,11 | 3,50E-12 | - | basic helix-loop-helix (bHLH) DNA-binding superfamily protein [Source:TAIR;Acc:AT2G20100] |
| AT2G20140 | -0,76 | 1,71E-71 | RPT2B | RPT2b [Source:UniProtKB/TrEMBL;Acc:A0A178VM16] |
| AT2G20142 | 2,25 | 2,85E-07 | - | Toll-Interleukin-Resistance (TIR) domain family protein [Source:UniProtKB/TrEMBL;Acc:F4IUF0] |
| AT2G20180 | 0,80 | 1,37E-03 | PIF1 | Transcription factor PIF1 [Source:UniProtKB/Swiss-Prot;Acc:Q8GZM7] |
| AT2G20250 | -1,20 | 5,85E-03 | - | unknown protein; Ha. [Source:TAIR;Acc:AT2G20250] |
| AT2G20260 | -1,82 | 6,71E-06 | PSAE2 | Photosystem I reaction center subunit IV B, chloroplastic [Source:UniProtKB/Swiss-Prot;Acc:Q9S714] |
| AT2G20290 | -0,78 | 1,53E-31 | XI-G | Myosin-13 [Source:UniProtKB/Swiss-Prot;Acc:F4IUG9] |
| AT2G20400 | 0,91 | 3,84E-09 | - | myb-like HTH transcriptional regulator family protein [Source:TAIR;Acc:AT2G20400] |
| AT2G20420 | -0,82 | 5,53E-142 | - | Succinate--CoA ligase [ADP-forming] subunit beta, mitochondrial [Source:UniProtKB/Swiss-Prot;Acc:O82662] |
| AT2G20450 | -0,90 | 2,59E-94 | RPL14A | 60S ribosomal protein L14-1 [Source:UniProtKB/Swiss-Prot;Acc:Q9SIM4] |
| AT2G20495 | 0,82 | 2,24E-16 | - | CONTAINS InterPro DOMAIN/s: Serine-threonine protein kinase 19 (InterPro:IPR018865); Ha. [Source:TAIR;Acc:AT2G20495] |
| AT2G20550 | 1,15 | 5,53E-04 | - | At2g20550 [Source:UniProtKB/TrEMBL;Acc:Q9SIL4] |
| AT2G20560 | 2,99 | 2,21E-203 | - | At2g20560/T13C7.15 [Source:UniProtKB/TrEMBL;Acc:Q9SIL3] |
| AT2G20570 | -2,01 | 2,05E-02 | GPRI1 | GBF's pro-rich region-interacting factor 1 [Source:UniProtKB/TrEMBL;Acc:F4IVF9] |
| AT2G20580 | -0,67 | 4,96E-74 | RPN1A | 26S proteasome non-ATPase regulatory subunit 2 homolog [Source:UniProtKB/TrEMBL;Acc:A0A178VZC8] |
| AT2G20590 | -0,90 | 1,69E-12 | RTNLB17 | Reticulon-like protein B17 [Source:UniProtKB/Swiss-Prot;Acc:Q6DR04] |
| AT2G20610 | -1,78 | 3,59E-157 | SUR1 | S-alkyl-thiohydroximate lyase SUR1 [Source:UniProtKB/Swiss-Prot;Acc:Q9SIV0] |
| AT2G20630 | -0,63 | 7,58E-19 | PPC3-1.2 | Probable protein phosphatase 2C 20 [Source:UniProtKB/Swiss-Prot;Acc:Q9SIU8] |
| AT2G20670 | -0,79 | 5,56E-06 | - | Expressed protein [Source:UniProtKB/TrEMBL;Acc:Q9SIU5] |
| AT2G20680 | -1,13 | 5,08E-92 | MAN2 | Mannan endo-1,4-beta-mannosidase 2 [Source:UniProtKB/Swiss-Prot;Acc:Q7Y223] |
| AT2G20720 | 4,60 | 4,01E-03 | - | At2g20720 [Source:UniProtKB/TrEMBL;Acc:Q8L705] |
| AT2G20750 | -0,76 | 5,77E-38 | EXPB1 | Expansin-B1 [Source:UniProtKB/Swiss-Prot;Acc:Q9SKU2] |
| AT2G20760 | -0,91 | 5,80E-80 | - | Clathrin light chain 1 [Source:UniProtKB/Swiss-Prot;Acc:Q9SKU1] |
| AT2G20770 | -0,67 | 4,67E-09 | GCL2 | LanC-like protein GCL2 [Source:UniProtKB/Swiss-Prot;Acc:Q8VZQ6] |
| AT2G20800 | 6,96 | 0,00E+00 | NDB4 | External alternative NAD(P)H-ubiquinone oxidoreductase B4, mitochondrial [Source:UniProtKB/Swiss-Prot;Acc:Q9SKT7] |
| AT2G20825 | 3,96 | 9,14E-18 | ULT2 | Protein ULTRAPETALA 2 [Source:UniProtKB/Swiss-Prot;Acc:Q8S8I2] |
| AT2G20840 | -1,17 | 8,69E-106 | SCAMP1 | Secretory carrier-associated membrane protein 1 [Source:UniProtKB/Swiss-Prot;Acc:Q9SKT3] |
| AT2G20890 | -0,71 | 7,40E-38 | THF1 | THF1 [Source:UniProtKB/TrEMBL;Acc:A0A178VVV5] |
| AT2G20920 | 1,08 | 8,88E-19 | - | At2g20920/F5H14.11 [Source:UniProtKB/TrEMBL;Acc:Q9SKS8] |
| AT2G20950 | 0,76 | 2,59E-10 | - | Arabidopsis phospholipase-like protein (PEARLI 4) family [Source:TAIR;Acc:AT2G20950] |
| AT2G20960 | 0,87 | 3,94E-28 | pEARLI4 | PEARLI 4 protein [Source:UniProtKB/TrEMBL;Acc:Q9SKR5] |
| AT2G20980 | -0,89 | 5,16E-39 | MCM10 | Minichromosome maintenance 10 [Source:UniProtKB/TrEMBL;Acc:Q5XVE2] |
| AT2G21040 | 2,08 | 1,65E-04 | - | Calcium-dependent lipid-binding (CaLB domain) family protein [Source:UniProtKB/TrEMBL;Acc:Q9SKQ7] |
| AT2G21045 | 1,15 | 1,74E-67 | HAC1 | Protein HIGH ARSENIC CONTENT 1, mitochondrial [Source:UniProtKB/Swiss-Prot;Acc:Q8RUD6] |
| AT2G21080 | 1,42 | 4,11E-19 | - | At2g21080 [Source:UniProtKB/TrEMBL;Acc:Q8L726] |
| AT2G21130 | 2,33 | 1,80E-165 | CYP19-2 | Peptidyl-prolyl cis-trans isomerase CYP19-2 [Source:UniProtKB/Swiss-Prot;Acc:Q9SKQ0] |
| AT2G21140 | -5,32 | 1,97E-04 | PRP2 | Proline-rich protein 2 [Source:UniProtKB/Swiss-Prot;Acc:Q9SKP9] |
| AT2G21160 | -1,45 | 4,81E-165 | - | Translocon-associated protein subunit alpha [Source:UniProtKB/Swiss-Prot;Acc:P45434] |
| AT2G21180 | 2,63 | 3,04E-89 | - | At2g21180/F26H11.6 [Source:UniProtKB/TrEMBL;Acc:Q9SKP5] |
| AT2G21187 | 1,50 | 3,37E-04 | - | other RNA [Source:TAIR;Acc:AT2G21187] |
| AT2G21188 | 1,02 | 3,18E-13 | - | other RNA [Source:TAIR;Acc:AT2G21188] |
| AT2G21250 | -0,67 | 8,72E-72 | - | NAD(P)-linked oxidoreductase superfamily protein [Source:UniProtKB/TrEMBL;Acc:Q9SJV2] |
| AT2G21260 | 1,29 | 4,01E-02 | - | At2g21260 [Source:UniProtKB/TrEMBL;Acc:Q9SJV1] |
| AT2G21290 | -0,64 | 2,11E-27 | - | 30S ribosomal protein S31, mitochondrial [Source:UniProtKB/Swiss-Prot;Acc:Q9SJU8] |
| AT2G21320 | 1,41 | 9,66E-07 | BBX18 | B-box zinc finger protein 18 [Source:UniProtKB/Swiss-Prot;Acc:Q9SJU5] |
| AT2G21330 | 4,83 | 5,48E-07 | FBA1 | Fructose-bisphosphate aldolase 1, chloroplastic [Source:UniProtKB/Swiss-Prot;Acc:Q9SJU4] |
| AT2G21350 | 1,13 | 1,63E-16 | - | RNA-binding CRS1 / YhbY (CRM) domain protein [Source:UniProtKB/TrEMBL;Acc:F4IGM0] |
| AT2G21385 | 0,80 | 1,11E-18 | - | Expressed protein [Source:UniProtKB/TrEMBL;Acc:Q94AU3] |
| AT2G21390 | -0,75 | 1,36E-67 | - | Coatomer subunit alpha-2 [Source:UniProtKB/Swiss-Prot;Acc:Q9SJT9] |
| AT2G21430 | 3,05 | 4,76E-64 | RD19B | Probable cysteine protease RD19B [Source:UniProtKB/Swiss-Prot;Acc:P43295] |
| AT2G21470 | -0,67 | 2,87E-44 | SAE2 | SUMO-activating enzyme subunit 2 [Source:UniProtKB/Swiss-Prot;Acc:Q9SJT1] |
| AT2G21500 | 0,77 | 2,59E-26 | - | At2g21560 [Source:UniProtKB/TrEMBL;Acc:Q8RWF9] |
| AT2G21510 | -3,35 | 7,25E-40 | - | DNAJ heat shock N-terminal domain-containing protein [Source:TAIR;Acc:AT2G21510] |
| AT2G21580 | -1,22 | 8,65E-190 | RPS25B | 40S ribosomal protein S25-2 [Source:UniProtKB/Swiss-Prot;Acc:Q9SIK2] |
| AT2G21640 | 7,09 | 0,00E+00 | - | Marker for oxidative stress response protein [Source:UniProtKB/TrEMBL;Acc:Q9SIJ6] |
| AT2G21650 | 2,49 | 2,15E-02 | RL2 | RSM1 [Source:UniProtKB/TrEMBL;Acc:A0A178VZA8] |
| AT2G21710 | 1,25 | 8,01E-30 | MTERF2 | Transcription termination factor MTERF2, chloroplastic [Source:UniProtKB/Swiss-Prot;Acc:F4IHL3] |
| AT2G21770 | -0,65 | 1,38E-02 | CESA9 | Probable cellulose synthase A catalytic subunit 9 [UDP-forming] [Source:UniProtKB/Swiss-Prot;Acc:Q9SJ22] |
| AT2G21780 | 1,81 | 3,22E-03 | - | At2g21780 [Source:UniProtKB/TrEMBL;Acc:Q9SJ21] |
| AT2G21830 | -1,84 | 3,36E-74 | - | Cysteine/Histidine-rich C1 domain family protein [Source:UniProtKB/TrEMBL;Acc:Q9SJ16] |
| AT2G21880 | -1,10 | 8,28E-08 | RABG2 | RAB7A [Source:UniProtKB/TrEMBL;Acc:A0A178VZI8] |
| AT2G21940 | 0,90 | 3,89E-22 | SK1 | Shikimate kinase 1 [Source:UniProtKB/TrEMBL;Acc:F4IIJ2] |
| AT2G22060 | 3,65 | 7,55E-11 | - | Galactose oxidase/kelch repeat protein [Source:UniProtKB/TrEMBL;Acc:Q9SHZ9] |
| AT2G22140 | -0,82 | 2,10E-09 | EME1B | Crossover junction endonuclease EME1B [Source:UniProtKB/Swiss-Prot;Acc:C5H8J1] |
| AT2G22230 | -0,66 | 6,27E-38 | - | At2g22230/T26C19.11 [Source:UniProtKB/TrEMBL;Acc:Q9SIE3] |
| AT2G22300 | 0,85 | 6,29E-45 | CAMTA3 | Calmodulin-binding transcription activator 3 [Source:UniProtKB/Swiss-Prot;Acc:Q8GSA7] |
| AT2G22330 | -2,23 | 1,57E-09 | CYP79B3 | cytochrome P450, family 79, subfamily B, polypeptide 3 [Source:TAIR;Acc:AT2G22330] |
| AT2G22420 | 0,73 | 3,84E-05 | PER17 | Peroxidase 17 [Source:UniProtKB/Swiss-Prot;Acc:Q9SJZ2] |
| AT2G22425 | -0,58 | 8,75E-24 | - | Probable signal peptidase complex subunit 1 [Source:UniProtKB/Swiss-Prot;Acc:Q944J0] |
| AT2G22430 | 0,78 | 7,18E-22 | ATHB-6 | Homeobox-leucine zipper protein ATHB-6 [Source:UniProtKB/Swiss-Prot;Acc:P46668] |
| AT2G22470 | 0,82 | 3,31E-12 | AGP2 | Classical arabinogalactan protein 2 [Source:UniProtKB/Swiss-Prot;Acc:Q9SJY7] |
| AT2G22475 | -0,61 | 1,54E-33 | GEM | GLABRA2 expression modulator [Source:UniProtKB/Swiss-Prot;Acc:Q8S8F8] |
| AT2G22500 | -0,59 | 3,49E-19 | PUMP5 | Mitochondrial uncoupling protein 5 [Source:UniProtKB/Swiss-Prot;Acc:Q9SJY5] |
| AT2G22560 | -1,21 | 1,53E-56 | NET2D | NET2D [Source:UniProtKB/TrEMBL;Acc:A0A178VLF4] |
| AT2G22610 | -0,81 | 8,14E-20 | - | Di-glucose binding protein with Kinesin motor domain [Source:TAIR;Acc:AT2G22610] |
| AT2G22620 | 0,69 | 3,23E-02 | - | Rhamnogalacturonate lyase family protein [Source:UniProtKB/TrEMBL;Acc:Q9ZQ51] |
| AT2G22630 | -0,80 | 1,09E-20 | AGL17 | AGL17 [Source:UniProtKB/TrEMBL;Acc:A0A384L577] |
| AT2G22730 | 0,71 | 1,81E-08 | - | Major facilitator superfamily protein [Source:TAIR;Acc:AT2G22730] |
| AT2G22790 | 0,58 | 2,29E-02 | - | Uncharacterized protein At2g22790 [Source:UniProtKB/TrEMBL;Acc:O82400] |
| AT2G22840 | -0,96 | 1,22E-78 | GRF1 | Growth-regulating factor 1 [Source:UniProtKB/Swiss-Prot;Acc:O81001] |
| AT2G22860 | 2,46 | 6,70E-15 | PSK2 | Phytosulfokines 2 [Source:UniProtKB/Swiss-Prot;Acc:O81003] |
| AT2G22870 | 0,87 | 3,43E-24 | EMB2001 | GTP-binding protein At2g22870 [Source:UniProtKB/Swiss-Prot;Acc:O81004] |
| AT2G22890 | -1,47 | 3,04E-03 | FAD4L2 | Fatty acid desaturase 4-like 2, chloroplastic [Source:UniProtKB/Swiss-Prot;Acc:O81006] |
| AT2G22930 | -3,15 | 1,74E-30 | UGT79B8 | UDP-glycosyltransferase 79B8 [Source:UniProtKB/Swiss-Prot;Acc:O81010] |
| AT2G23010 | 2,03 | 7,59E-38 | SCPL9 | SCPL9 [Source:UniProtKB/TrEMBL;Acc:A0A178W1Q9] |
| AT2G23030 | 3,05 | 1,16E-03 | SRK2J | SNRK2.9 [Source:UniProtKB/TrEMBL;Acc:A0A178VSC8] |
| AT2G23050 | -0,72 | 5,48E-48 | NPY4 | BTB/POZ domain-containing protein NPY4 [Source:UniProtKB/Swiss-Prot;Acc:O64814] |
| AT2G23093 | -0,66 | 4,93E-15 | - | Major facilitator superfamily protein [Source:UniProtKB/TrEMBL;Acc:Q8GXH4] |
| AT2G23100 | 1,08 | 8,00E-04 | - | Cysteine/Histidine-rich C1 domain family protein [Source:UniProtKB/TrEMBL;Acc:F4ILF9] |
| AT2G23110 | 2,17 | 2,78E-04 | - | At2g23110 [Source:UniProtKB/TrEMBL;Acc:O64820] |
| AT2G23120 | 0,66 | 8,37E-40 | - | Expressed protein [Source:UniProtKB/TrEMBL;Acc:Q8S8R1] |
| AT2G23130 | 2,68 | 4,37E-60 | AGP17 | Lysine-rich arabinogalactan protein 17 [Source:UniProtKB/Swiss-Prot;Acc:O22194] |
| AT2G23170 | 3,33 | 1,30E-04 | GH3.3 | Indole-3-acetic acid-amido synthetase GH3.3 [Source:UniProtKB/Swiss-Prot;Acc:O22190] |
| AT2G23180 | 1,55 | 4,42E-08 | CYP96A1 | Cytochrome P450, family 96, subfamily A, polypeptide 1 [Source:UniProtKB/TrEMBL;Acc:O22189] |
| AT2G23200 | 0,89 | 8,65E-04 | - | Probable receptor-like protein kinase At2g23200 [Source:UniProtKB/Swiss-Prot;Acc:O22187] |
| AT2G23290 | -0,95 | 1,45E-10 | AtMYB70 | At2g23280 [Source:UniProtKB/TrEMBL;Acc:O22179] |
| AT2G23310 | -0,65 | 1,04E-46 | RER1C | Protein RER1C [Source:UniProtKB/Swiss-Prot;Acc:Q9ZWI7] |
| AT2G23320 | 2,07 | 5,36E-186 | WRKY15 | Probable WRKY transcription factor 15 [Source:UniProtKB/Swiss-Prot;Acc:O22176] |
| AT2G23340 | 0,59 | 2,42E-05 | ERF008 | Ethylene-responsive transcription factor ERF008 [Source:UniProtKB/Swiss-Prot;Acc:O22174] |
| AT2G23347 | 1,52 | 5,04E-04 | MIR844A | MIR844a; miRNA [Source:TAIR;Acc:AT2G23347] |
| AT2G23348 | 2,08 | 3,99E-11 | - | unknown protein; FUNCTIONS IN: molecular_function unknown; INVOLVED IN: biological_process unknown; LOCATED IN: endomembrane system; Ha. [Source:TAIR;Acc:AT2G23348] |
| AT2G23360 | -0,64 | 7,71E-19 | FPP7 | Filament-like plant protein 7 [Source:UniProtKB/Swiss-Prot;Acc:Q9SLN1] |
| AT2G23390 | 0,62 | 3,02E-14 | - | Acyl-CoA [Source:UniProtKB/TrEMBL;Acc:O80456] |
| AT2G23400 | -2,73 | 1,24E-10 | - | Undecaprenyl pyrophosphate synthetase family protein [Source:TAIR;Acc:AT2G23400] |
| AT2G23410 | -2,72 | 3,83E-30 | DPS | Dehydrodolichyl diphosphate synthase 1 [Source:UniProtKB/Swiss-Prot;Acc:O80458] |
| AT2G23430 | 1,09 | 2,93E-02 | KRP1 | Cyclin-dependent kinase inhibitor 1 [Source:UniProtKB/Swiss-Prot;Acc:Q67Y93] |
| AT2G23450 | 1,54 | 3,99E-48 | WAKL14 | Wall-associated receptor kinase-like 14 [Source:UniProtKB/Swiss-Prot;Acc:Q8RY67] |
| AT2G23530 | -0,71 | 4,08E-08 | - | Zinc-finger domain of monoamine-oxidase A repressor R1 [Source:UniProtKB/TrEMBL;Acc:Q1PF11] |
| AT2G23550 | 1,19 | 4,68E-10 | ATMES6 | methyl esterase 6 [Source:TAIR;Acc:AT2G23550] |
| AT2G23630 | -1,19 | 3,35E-57 | sks16 | SKU5 similar 16 [Source:TAIR;Acc:AT2G23630] |
| AT2G23680 | 0,65 | 2,96E-02 | - | Cold-regulated 413 plasma membrane protein 3 [Source:UniProtKB/Swiss-Prot;Acc:O64834] |
| AT2G23760 | 4,37 | 1,78E-02 | BLH4 | BEL1-like homeodomain 4 [Source:TAIR;Acc:AT2G23760] |
| AT2G23770 | 0,95 | 1,04E-04 | LYK4 | LysM domain receptor-like kinase 4 [Source:UniProtKB/Swiss-Prot;Acc:O64825] |
| AT2G23810 | 1,69 | 5,21E-73 | TET8 | TET8 [Source:UniProtKB/TrEMBL;Acc:A0A178VYI5] |
| AT2G23910 | 1,58 | 7,71E-18 | - | At2g23910 [Source:UniProtKB/TrEMBL;Acc:O82219] |
| AT2G23940 | -0,87 | 3,89E-45 | - | Expressed protein [Source:UniProtKB/TrEMBL;Acc:O82222] |
| AT2G23945 | -2,00 | 1,69E-02 | - | Eukaryotic aspartyl protease family protein [Source:UniProtKB/TrEMBL;Acc:Q8S8N7] |
| AT2G23960 | -0,90 | 8,53E-05 | GGP4 | Gamma-glutamyl peptidase 4 [Source:UniProtKB/Swiss-Prot;Acc:F4INN2] |
| AT2G24050 | -0,84 | 7,81E-95 | EIF(ISO)4G2 | Eukaryotic translation initiation factor isoform 4G-2 [Source:UniProtKB/Swiss-Prot;Acc:O82233] |
| AT2G24070 | -0,93 | 2,77E-17 | QWRF4 | QWRF motif-containing protein 4 [Source:UniProtKB/Swiss-Prot;Acc:F4INP9] |
| AT2G24100 | 0,70 | 3,66E-15 | - | ATP-dependent DNA helicase [Source:UniProtKB/TrEMBL;Acc:Q9ZUI1] |
| AT2G24120 | 0,66 | 7,10E-30 | RPOT3 | DNA-directed RNA polymerase 3, chloroplastic [Source:UniProtKB/Swiss-Prot;Acc:O24600] |
| AT2G24170 | -0,94 | 1,80E-82 | TMN10 | Transmembrane 9 superfamily member 10 [Source:UniProtKB/Swiss-Prot;Acc:Q8RWW1] |
| AT2G24250 | 1,52 | 1,15E-28 | - | F-box/kelch-repeat protein At2g24250 [Source:UniProtKB/Swiss-Prot;Acc:Q9ZUH0] |
| AT2G24255 | 3,59 | 2,19E-03 | - | LOW protein: F-box/kelch-repeat protein [Source:UniProtKB/TrEMBL;Acc:Q8S8L1] |
| AT2G24280 | 0,78 | 5,19E-49 | - | Alpha/beta-Hydrolases superfamily protein [Source:UniProtKB/TrEMBL;Acc:Q93Z34] |
| AT2G24285 | 2,76 | 4,06E-02 | - | BEST Arabidopsis thaliana protein match is: Zinc finger, C3HC4 type (RING finger) family protein (TAIR:AT2G24480.1); Ha. [Source:TAIR;Acc:AT2G24285] |
| AT2G24290 | -0,77 | 4,45E-32 | - | Expressed protein [Source:UniProtKB/TrEMBL;Acc:Q9ZQ38] |
| AT2G24300 | -1,03 | 2,80E-74 | - | Calmodulin-binding protein [Source:TAIR;Acc:AT2G24300] |
| AT2G24350 | 0,65 | 2,03E-05 | - | At2g24350 [Source:UniProtKB/TrEMBL;Acc:Q0WPW8] |
| AT2G24400 | -2,50 | 3,46E-72 | - | At2g24400 [Source:UniProtKB/TrEMBL;Acc:Q9ZQ28] |
| AT2G24510 | 6,33 | 1,19E-07 | - | F-box associated ubiquitination effector family protein [Source:UniProtKB/TrEMBL;Acc:F4IPQ0] |
| AT2G24545 | 1,23 | 4,91E-02 | - | other RNA [Source:TAIR;Acc:AT2G24545] |
| AT2G24560 | 2,88 | 1,60E-27 | - | GDSL esterase/lipase At2g24560 [Source:UniProtKB/Swiss-Prot;Acc:Q9SJA9] |
| AT2G24570 | 0,93 | 1,94E-51 | WRKY17 | WRKY transcription factor 17 [Source:UniProtKB/TrEMBL;Acc:Q0WTF3] |
| AT2G24580 | 2,29 | 1,17E-109 | - | Probable sarcosine oxidase [Source:UniProtKB/Swiss-Prot;Acc:Q9SJA7] |
| AT2G24590 | -0,81 | 3,18E-56 | RSZ22A | Serine/arginine-rich splicing factor RSZ22A [Source:UniProtKB/Swiss-Prot;Acc:Q9SJA6] |
| AT2G24600 | 0,84 | 1,10E-02 | - | Ankyrin repeat family protein [Source:UniProtKB/TrEMBL;Acc:F4IPR3] |
| AT2G24680 | 1,24 | 3,81E-38 | - | transcriptional factor B3 family protein [Source:TAIR;Acc:AT2G24680] |
| AT2G24762 | -1,87 | 9,40E-03 | GDU4 | Protein GLUTAMINE DUMPER 4 [Source:UniProtKB/Swiss-Prot;Acc:Q8S8A0] |
| AT2G24765 | -0,69 | 2,75E-45 | ARF3 | ADP-ribosylation factor 3 [Source:UniProtKB/Swiss-Prot;Acc:P40940] |
| AT2G24800 | -5,93 | 3,97E-62 | PER18 | Peroxidase 18 [Source:UniProtKB/Swiss-Prot;Acc:Q9SK52] |
| AT2G24820 | 1,50 | 1,62E-28 | TIC55 | Protein TIC 55, chloroplastic [Source:UniProtKB/Swiss-Prot;Acc:Q9SK50] |
| AT2G24830 | 0,72 | 2,34E-31 | - | Zinc finger CCCH domain-containing protein 22 [Source:UniProtKB/Swiss-Prot;Acc:Q9SK49] |
| AT2G24850 | -0,74 | 2,62E-03 | TAT3 | Probable aminotransferase TAT3 [Source:UniProtKB/Swiss-Prot;Acc:Q9SK47] |
| AT2G24940 | 1,08 | 1,89E-68 | MP3 | MAPR2 [Source:UniProtKB/TrEMBL;Acc:A0A178VV33] |
| AT2G24945 | 1,19 | 2,13E-37 | - | unknown protein; BEST Arabidopsis thaliana protein match is: unknown protein (TAIR:AT4G36925.1); Ha. [Source:TAIR;Acc:AT2G24945] |
| AT2G24970 | -0,74 | 1,38E-03 | - | At2g24970 [Source:UniProtKB/TrEMBL;Acc:Q9SK36] |
| AT2G24980 | -4,96 | 9,78E-04 | - | Proline-rich extensin-like family protein [Source:TAIR;Acc:AT2G24980] |
| AT2G25000 | 0,59 | 3,25E-02 | WRKY60 | Probable WRKY transcription factor 60 [Source:UniProtKB/Swiss-Prot;Acc:Q9SK33] |
| AT2G25010 | 0,98 | 6,82E-37 | MAIL1 | Protein MAIN-LIKE 1 [Source:UniProtKB/Swiss-Prot;Acc:Q9SK32] |
| AT2G25060 | -0,82 | 7,92E-35 | ENODL14 | ENODL14 [Source:UniProtKB/TrEMBL;Acc:A0A178VWH0] |
| AT2G25070 | -0,69 | 1,74E-39 | PPC4-2 | Probable protein phosphatase 2C 21 [Source:UniProtKB/Swiss-Prot;Acc:O81716] |
| AT2G25130 | -2,14 | 1,30E-33 | - | ARM repeat superfamily protein [Source:UniProtKB/TrEMBL;Acc:Q8H1N2] |
| AT2G25140 | 2,25 | 0,00E+00 | CLPB4 | Chaperone protein ClpB4, mitochondrial [Source:UniProtKB/Swiss-Prot;Acc:Q8VYJ7] |
| AT2G25160 | -2,66 | 1,45E-37 | CYP82F1 | At2g25160 [Source:UniProtKB/TrEMBL;Acc:Q7Y222] |
| AT2G25210 | -0,98 | 1,49E-52 | - | Ribosomal protein L39 family protein [Source:TAIR;Acc:AT2G25210] |
| AT2G25220 | -1,27 | 2,68E-34 | - | Protein kinase superfamily protein [Source:UniProtKB/TrEMBL;Acc:F4IRL7] |
| AT2G25260 | -1,70 | 2,49E-42 | HPAT2 | Hydroxyproline O-arabinosyltransferase 2 [Source:UniProtKB/Swiss-Prot;Acc:Q494Q2] |
| AT2G25330 | 3,05 | 5,01E-03 | - | TRAF-like family protein [Source:UniProtKB/TrEMBL;Acc:Q9SIR0] |
| AT2G25440 | 0,61 | 1,53E-03 | AtRLP20 | Receptor-like protein 20 [Source:UniProtKB/Swiss-Prot;Acc:Q9SKK5] |
| AT2G25450 | 1,70 | 1,88E-98 | GSL-OH | Probable 2-oxoacid dependent dioxygenase [Source:UniProtKB/Swiss-Prot;Acc:Q9SKK4] |
| AT2G25460 | 1,52 | 1,52E-35 | - | CONTAINS InterPro DOMAIN/s: C2 calcium-dependent membrane targeting (InterPro:IPR000008); BEST Arabidopsis thaliana protein match is: unknown protein (TAIR:AT5G04860.1); Ha. [Source:TAIR;Acc:AT2G25460] |
| AT2G25520 | -0,63 | 8,47E-63 | - | Probable sugar phosphate/phosphate translocator At2g25520 [Source:UniProtKB/Swiss-Prot;Acc:Q9SKJ7] |
| AT2G25590 | 0,84 | 1,83E-29 | - | Plant Tudor-like protein [Source:UniProtKB/TrEMBL;Acc:Q9SLA4] |
| AT2G25605 | 0,63 | 5,22E-05 | - | At2g25605 [Source:UniProtKB/TrEMBL;Acc:Q6NM03] |
| AT2G25610 | -0,98 | 2,26E-72 | - | ATPase, F0/V0 complex, subunit C protein [Source:TAIR;Acc:AT2G25610] |
| AT2G25650 | 0,66 | 5,88E-13 | GPL1 | GLABROUS1 enhancer-binding protein-like 1 [Source:UniProtKB/Swiss-Prot;Acc:Q8VYD2] |
| AT2G25690 | -0,67 | 2,05E-10 | FLZ11 | FCS-Like Zinc finger 11 [Source:UniProtKB/Swiss-Prot;Acc:Q9SL94] |
| AT2G25770 | 1,60 | 1,07E-27 | - | At2g25770 [Source:UniProtKB/TrEMBL;Acc:O82320] |
| AT2G25790 | 0,65 | 2,78E-09 | - | Probably inactive leucine-rich repeat receptor-like protein kinase At2g25790 [Source:UniProtKB/Swiss-Prot;Acc:O82318] |
| AT2G25810 | -0,65 | 1,36E-23 | TIP4-1 | Aquaporin TIP4-1 [Source:UniProtKB/Swiss-Prot;Acc:O82316] |
| AT2G25880 | -1,25 | 1,77E-32 | AtAUR2 | ataurora2 [Source:TAIR;Acc:AT2G25880] |
| AT2G25920 | 0,60 | 2,80E-13 | - | At2g25920/F17H15.5 [Source:UniProtKB/TrEMBL;Acc:O82305] |
| AT2G25940 | 0,73 | 4,52E-02 | ALPHA-VPE | Vacuolar-processing enzyme alpha-isozyme [Source:UniProtKB/Swiss-Prot;Acc:P49047] |
| AT2G25950 | 1,09 | 1,96E-65 | - | At2g25950 [Source:UniProtKB/TrEMBL;Acc:O82808] |
| AT2G25980 | -1,15 | 2,75E-162 | JAL20 | Jacalin-related lectin 20 [Source:UniProtKB/Swiss-Prot;Acc:O80998] |
| AT2G25990 | -4,33 | 1,18E-03 | - | Putative uncharacterized protein At2g25990 [Source:UniProtKB/TrEMBL;Acc:O80997] |
| AT2G26000 | 0,73 | 2,80E-18 | BRIZ2 | At2g26000 [Source:UniProtKB/TrEMBL;Acc:O80996] |
| AT2G26030 | 0,76 | 2,40E-12 | - | F-box/RNI-like/FBD-like domains-containing protein [Source:TAIR;Acc:AT2G26030] |
| AT2G26070 | 0,90 | 3,56E-18 | RTE1 | Protein of unknown function (DUF778) [Source:TAIR;Acc:AT2G26070] |
| AT2G26080 | -0,66 | 1,24E-68 | GLDP2 | Glycine dehydrogenase (decarboxylating) 2, mitochondrial [Source:UniProtKB/Swiss-Prot;Acc:O80988] |
| AT2G26110 | -0,81 | 2,32E-28 | - | At2g26110 [Source:UniProtKB/TrEMBL;Acc:O80986] |
| AT2G26150 | 2,07 | 5,88E-53 | HSFA2 | Heat stress transcription factor A-2 [Source:UniProtKB/Swiss-Prot;Acc:O80982] |
| AT2G26170 | 0,85 | 1,64E-10 | CYP711A1 | Cytochrome P450 711A1 [Source:UniProtKB/Swiss-Prot;Acc:B9DFU2] |
| AT2G26250 | -3,83 | 1,02E-09 | FDH | 3-ketoacyl-CoA synthase [Source:UniProtKB/TrEMBL;Acc:A0A178VYA3] |
| AT2G26290 | 4,88 | 4,77E-31 | PBL12 | Probable serine/threonine-protein kinase PBL12 [Source:UniProtKB/Swiss-Prot;Acc:O64842] |
| AT2G26340 | 0,83 | 5,14E-11 | - | unknown protein; FUNCTIONS IN: molecular_function unknown; INVOLVED IN: biological_process unknown; LOCATED IN: chloroplast thylakoid membrane, chloroplast thylakoid lumen, chloroplast; EXPRESSED IN: 22 plant structures; EXPRESSED DURING: 13 growth /.../; Ha. [Source:TAIR;Acc:AT2G26340] |
| AT2G26355 | 0,65 | 6,00E-13 | - | other RNA [Source:TAIR;Acc:AT2G26355] |
| AT2G26360 | -1,04 | 1,22E-31 | - | Mitochondrial substrate carrier family protein [Source:UniProtKB/TrEMBL;Acc:F4IUJ2] |
| AT2G26370 | -0,85 | 4,07E-02 | - | At2g26370 [Source:UniProtKB/TrEMBL;Acc:O48704] |
| AT2G26390 | 1,01 | 2,36E-15 | - | Serpin-Z3 [Source:UniProtKB/Swiss-Prot;Acc:O48706] |
| AT2G26420 | 0,80 | 3,43E-16 | PIP5K3 | 1-phosphatidylinositol-4-phosphate 5-kinase 3 [Source:TAIR;Acc:AT2G26420] |
| AT2G26440 | 2,07 | 2,87E-85 | PME12 | Probable pectinesterase/pectinesterase inhibitor 12 [Source:UniProtKB/Swiss-Prot;Acc:O48711] |
| AT2G26450 | 6,42 | 3,69E-08 | PME13 | Probable pectinesterase/pectinesterase inhibitor 13 [Source:UniProtKB/Swiss-Prot;Acc:Q7Y201] |
| AT2G26480 | -2,96 | 1,43E-07 | UGT76D1 | UDP-glycosyltransferase 76D1 [Source:UniProtKB/Swiss-Prot;Acc:O48715] |
| AT2G26500 | -1,48 | 5,08E-05 | - | At2g26500/T9J22.17 [Source:UniProtKB/TrEMBL;Acc:O48717] |
| AT2G26520 | -1,80 | 4,14E-19 | - | Expressed protein [Source:UniProtKB/TrEMBL;Acc:O48719] |
| AT2G26530 | -1,99 | 1,96E-03 | AR781 | AR781 [Source:UniProtKB/TrEMBL;Acc:A0A178VPT1] |
| AT2G26560 | 1,06 | 4,21E-05 | PLP2 | Patatin-like protein 2 [Source:UniProtKB/Swiss-Prot;Acc:O48723] |
| AT2G26640 | -0,63 | 9,16E-25 | KCS11 | 3-ketoacyl-CoA synthase 11 [Source:UniProtKB/Swiss-Prot;Acc:O48780] |
| AT2G26660 | -0,58 | 5,56E-13 | SPX2 | SPX domain-containing protein 2 [Source:UniProtKB/Swiss-Prot;Acc:O48781] |
| AT2G26680 | -0,94 | 1,35E-43 | - | Expressed protein [Source:UniProtKB/TrEMBL;Acc:O48783] |
| AT2G26690 | -1,59 | 1,58E-25 | NPF6.2 | Protein NRT1/ PTR FAMILY 6.2 [Source:UniProtKB/Swiss-Prot;Acc:Q9SZY4] |
| AT2G26695 | 4,97 | 3,81E-04 | - | At2g26695 [Source:UniProtKB/TrEMBL;Acc:Q6ID73] |
| AT2G26700 | 0,63 | 1,16E-08 | PID2 | Protein kinase PINOID 2 [Source:UniProtKB/Swiss-Prot;Acc:Q64FQ2] |
| AT2G26710 | 0,86 | 9,42E-22 | CYP734A1 | CYP734A1 [Source:UniProtKB/TrEMBL;Acc:A0A178VRF1] |
| AT2G26740 | 2,99 | 4,95E-03 | ATSEH | At2g26740/F18A8.11 [Source:UniProtKB/TrEMBL;Acc:Q42566] |
| AT2G26760 | -1,29 | 3,59E-42 | CYCB1-4 | CYCB1 [Source:UniProtKB/TrEMBL;Acc:A0A178VPU5] |
| AT2G26830 | -0,67 | 1,08E-27 | EMB1187 | Probable ethanolamine kinase [Source:UniProtKB/Swiss-Prot;Acc:O81024] |
| AT2G26860 | 0,96 | 1,96E-42 | - | FBD-associated F-box protein At2g26860 [Source:UniProtKB/Swiss-Prot;Acc:Q84X02] |
| AT2G26870 | 0,67 | 2,04E-03 | NPC2 | Non-specific phospholipase C2 [Source:UniProtKB/Swiss-Prot;Acc:O81020] |
| AT2G26910 | -1,36 | 4,10E-21 | ABCG32 | PEC1 [Source:UniProtKB/TrEMBL;Acc:A0A178VNA5] |
| AT2G26920 | 0,72 | 7,78E-35 | - | At2g26920 [Source:UniProtKB/TrEMBL;Acc:O81015] |
| AT2G27030 | -0,77 | 1,05E-70 | CAM5 | Calmodulin 5 [Source:UniProtKB/TrEMBL;Acc:F4IVN6] |
| AT2G27035 | 3,72 | 4,33E-02 | ENODL20 | Early nodulin-like protein 20 [Source:UniProtKB/TrEMBL;Acc:F4IVN9] |
| AT2G27060 | 1,44 | 2,46E-49 | - | Leucine-rich repeat protein kinase family protein [Source:UniProtKB/TrEMBL;Acc:F4IVP3] |
| AT2G27120 | -2,51 | 2,09E-04 | POL2B | DNA polymerase epsilon catalytic subunit [Source:TAIR;Acc:AT2G27120] |
| AT2G27150 | -0,70 | 1,61E-29 | AAO3 | Abscisic-aldehyde oxidase [Source:UniProtKB/Swiss-Prot;Acc:Q7G9P4] |
| AT2G27250 | -3,82 | 1,38E-02 | CLV3 | Protein CLAVATA 3 [Source:UniProtKB/Swiss-Prot;Acc:Q9XF04] |
| AT2G27290 | 1,63 | 8,38E-13 | - | At2g27290 [Source:UniProtKB/TrEMBL;Acc:Q9XIN6] |
| AT2G27310 | 1,87 | 6,36E-30 | - | F-box protein At2g27310 [Source:UniProtKB/Swiss-Prot;Acc:Q9XIN8] |
| AT2G27330 | -0,74 | 2,78E-28 | - | At2g27330 [Source:UniProtKB/TrEMBL;Acc:Q1ECN0] |
| AT2G27360 | -0,79 | 2,35E-08 | - | GDSL esterase/lipase At2g27360 [Source:UniProtKB/Swiss-Prot;Acc:Q9ZQI3] |
| AT2G27389 | 1,50 | 5,61E-04 | - | unknown protein; LOCATED IN: endomembrane system. [Source:TAIR;Acc:AT2G27389] |
| AT2G27430 | -1,68 | 1,53E-42 | - | ARM repeat superfamily protein [Source:UniProtKB/TrEMBL;Acc:Q8GUS7] |
| AT2G27450 | 0,62 | 1,53E-43 | CPA | N-carbamoylputrescine amidase [Source:UniProtKB/Swiss-Prot;Acc:Q8VYF5] |
| AT2G27460 | -0,70 | 2,21E-22 | - | At2g27460 [Source:UniProtKB/TrEMBL;Acc:Q9ZQH3] |
| AT2G27480 | 0,99 | 2,41E-10 | CML48 | Probable calcium-binding protein CML48 [Source:UniProtKB/Swiss-Prot;Acc:Q9ZQH1] |
| AT2G27500 | 0,97 | 4,10E-32 | - | Glucan endo-1,3-beta-glucosidase 14 [Source:UniProtKB/Swiss-Prot;Acc:Q9ZQG9] |
| AT2G27530 | -0,77 | 2,01E-67 | RPL10AB | 60S ribosomal protein L10a-2 [Source:UniProtKB/Swiss-Prot;Acc:P59230] |
| AT2G27550 | -2,13 | 1,54E-08 | CEN | Protein CENTRORADIALIS-like [Source:UniProtKB/Swiss-Prot;Acc:Q9ZNV5] |
| AT2G27580 | 0,60 | 5,31E-29 | SAP3 | Zinc finger A20 and AN1 domain-containing stress-associated protein 3 [Source:UniProtKB/Swiss-Prot;Acc:Q9ZNU9] |
| AT2G27660 | 1,02 | 4,58E-30 | - | Cysteine/Histidine-rich C1 domain family protein [Source:UniProtKB/TrEMBL;Acc:Q9ZUW8] |
| AT2G27680 | 0,71 | 8,57E-09 | - | At2g27680/F15K20.22 [Source:UniProtKB/TrEMBL;Acc:Q9ZUX0] |
| AT2G27710 | -1,00 | 9,11E-155 | RPP2B | AT2G27710 protein [Source:UniProtKB/TrEMBL;Acc:B9DGN3] |
| AT2G27720 | -0,66 | 5,37E-112 | - | 60S acidic ribosomal protein family [Source:UniProtKB/TrEMBL;Acc:F4IGR4] |
| AT2G27730 | -0,85 | 1,03E-70 | - | Uncharacterized protein At2g27730, mitochondrial [Source:UniProtKB/Swiss-Prot;Acc:Q9ZUX4] |
| AT2G27740 | -0,73 | 3,34E-03 | - | At2g27740 [Source:UniProtKB/TrEMBL;Acc:Q9ZUX5] |
| AT2G27830 | 3,81 | 7,12E-11 | - | Expressed protein [Source:UniProtKB/TrEMBL;Acc:Q9ZUY4] |
| AT2G27840 | -0,99 | 2,52E-102 | HDT4 | Histone deacetylase HDT4 [Source:UniProtKB/Swiss-Prot;Acc:Q9M4T3] |
| AT2G27920 | -1,81 | 1,75E-02 | SCPL51 | Serine carboxypeptidase-like 51 [Source:UniProtKB/Swiss-Prot;Acc:Q67Y83] |
| AT2G27930 | -0,58 | 4,46E-03 | - | PLATZ transcription factor family protein [Source:TAIR;Acc:AT2G27930] |
| AT2G27970 | -0,75 | 1,04E-36 | CKS2 | Cyclin-dependent kinases regulatory subunit 2 [Source:UniProtKB/Swiss-Prot;Acc:Q9SJJ5] |
| AT2G28040 | 1,15 | 5,37E-04 | - | At2g28040 [Source:UniProtKB/TrEMBL;Acc:Q84WH0] |
| AT2G28100 | -1,77 | 3,53E-43 | FUC1 | Alpha-L-fucosidase 1 [Source:UniProtKB/Swiss-Prot;Acc:Q8GW72] |
| AT2G28105 | 0,88 | 1,07E-04 | - | Replication factor-A carboxy-terminal domain protein [Source:UniProtKB/TrEMBL;Acc:Q5XVD2] |
| AT2G28120 | 2,11 | 9,40E-15 | - | At2g28120/F24D13.9 [Source:UniProtKB/TrEMBL;Acc:Q9ZUV4] |
| AT2G28310 | -0,64 | 1,87E-24 | - | Trimethylguanosine synthase (DUF707) [Source:UniProtKB/TrEMBL;Acc:Q56Y17] |
| AT2G28360 | -0,65 | 2,80E-25 | - | SIT4 phosphatase-associated family protein [Source:UniProtKB/TrEMBL;Acc:F4IIN7] |
| AT2G28410 | -0,70 | 3,87E-07 | - | Expressed protein [Source:UniProtKB/TrEMBL;Acc:Q9SKM9] |
| AT2G28440 | 0,59 | 1,49E-05 | - | En/Spm-like transposon protein [Source:UniProtKB/TrEMBL;Acc:Q9SKM6] |
| AT2G28450 | 0,62 | 1,93E-40 | - | Zinc finger CCCH domain-containing protein 24 [Source:UniProtKB/Swiss-Prot;Acc:Q8L7S3] |
| AT2G28470 | 0,74 | 3,30E-23 | BGAL8 | Beta-galactosidase 8 [Source:UniProtKB/Swiss-Prot;Acc:Q9SCV4] |
| AT2G28520 | -0,80 | 4,06E-94 | VHA-A1 | V-type proton ATPase subunit a1 [Source:UniProtKB/Swiss-Prot;Acc:Q8RWZ7] |
| AT2G28570 | -0,74 | 5,07E-03 | - | At2g28570 [Source:UniProtKB/TrEMBL;Acc:Q9SK01] |
| AT2G28590 | -1,25 | 3,94E-13 | PBL6 | Probable serine/threonine-protein kinase PBL6 [Source:UniProtKB/Swiss-Prot;Acc:Q9SIB6] |
| AT2G28620 | -0,93 | 1,44E-52 | - | P-loop containing nucleoside triphosphate hydrolases superfamily protein [Source:TAIR;Acc:AT2G28620] |
| AT2G28650 | 1,40 | 1,64E-09 | ATEXO70H8 | Exocyst subunit Exo70 family protein [Source:UniProtKB/TrEMBL;Acc:Q9SIB0] |
| AT2G28671 | 1,38 | 2,16E-02 | - | unknown protein; FUNCTIONS IN: molecular_function unknown; INVOLVED IN: biological_process unknown; LOCATED IN: cellular_component unknown; Ha. [Source:TAIR;Acc:AT2G28671] |
| AT2G28690 | 1,52 | 4,69E-02 | - | TOX high mobility group box protein, putative (DUF1635) [Source:UniProtKB/TrEMBL;Acc:Q8L8B6] |
| AT2G28700 | 4,89 | 9,54E-05 | AGL46 | AGAMOUS-like 46 [Source:UniProtKB/TrEMBL;Acc:F4IIT6] |
| AT2G28720 | -1,90 | 1,90E-44 | - | Histone H2B.3 [Source:UniProtKB/Swiss-Prot;Acc:Q9SI96] |
| AT2G28740 | -1,60 | 6,57E-129 | HIS4 | Histone H4 [Source:UniProtKB/Swiss-Prot;Acc:P59259] |
| AT2G28815 | 6,78 | 0,00E+00 | - | 60S ribosomal protein L16-like, mitochondrial [Source:UniProtKB/Swiss-Prot;Acc:Q84WZ8] |
| AT2G28910 | 1,37 | 1,10E-192 | CXIP4 | CXIP4 [Source:UniProtKB/TrEMBL;Acc:A0A178VV16] |
| AT2G28960 | -2,21 | 4,75E-16 | - | Probable LRR receptor-like serine/threonine-protein kinase At2g28960 [Source:UniProtKB/Swiss-Prot;Acc:C0LGL4] |
| AT2G28970 | -1,04 | 8,38E-05 | - | Leucine-rich repeat protein kinase family protein [Source:UniProtKB/TrEMBL;Acc:O81067] |
| AT2G29060 | 1,37 | 8,22E-13 | SCL33 | Scarecrow-like protein 33 [Source:UniProtKB/Swiss-Prot;Acc:P0C883] |
| AT2G29110 | 3,33 | 1,61E-05 | ATGLR2.8 | Glutamate receptor [Source:UniProtKB/TrEMBL;Acc:A0A178VW76] |
| AT2G29125 | -0,75 | 8,28E-07 | RTFL2 | RTFL2 [Source:UniProtKB/TrEMBL;Acc:A0A178VPZ1] |
| AT2G29140 | 0,75 | 1,22E-86 | APUM3 | Pumilio homolog 3 [Source:UniProtKB/Swiss-Prot;Acc:Q9ZW02] |
| AT2G29263 | 4,16 | 1,32E-02 | - | unknown protein; LOCATED IN: mitochondrion; Ha. [Source:TAIR;Acc:AT2G29263] |
| AT2G29330 | 3,36 | 4,09E-141 | TRI | Tropinone reductase homolog At2g29330 [Source:UniProtKB/Swiss-Prot;Acc:Q9ZW16] |
| AT2G29340 | 6,05 | 3,97E-14 | - | Tropinone reductase homolog At2g29340 [Source:UniProtKB/Swiss-Prot;Acc:F4IKM1] |
| AT2G29360 | -3,00 | 7,37E-07 | SDR | Tropinone reductase homolog At2g29360 [Source:UniProtKB/Swiss-Prot;Acc:Q9ZW19] |
| AT2G29380 | -0,79 | 1,54E-03 | HAI3 | HAI3 [Source:UniProtKB/TrEMBL;Acc:A0A178VWK9] |
| AT2G29400 | 1,20 | 0,00E+00 | TOPP1 | Serine/threonine-protein phosphatase [Source:UniProtKB/TrEMBL;Acc:A0A178VXL8] |
| AT2G29440 | -0,90 | 2,44E-49 | GSTU6 | GSTU6 [Source:UniProtKB/TrEMBL;Acc:A0A178VUQ0] |
| AT2G29450 | 1,80 | 3,56E-94 | GSTU5 | GSTU5 [Source:UniProtKB/TrEMBL;Acc:A0A178VPP0] |
| AT2G29500 | 4,56 | 1,22E-260 | HSP17.6B | 17.6 kDa class I heat shock protein 2 [Source:UniProtKB/Swiss-Prot;Acc:Q9ZW31] |
| AT2G29560 | -0,66 | 4,30E-38 | ENO3 | Cytosolic enolase 3 [Source:UniProtKB/Swiss-Prot;Acc:Q9ZW34] |
| AT2G29620 | -6,58 | 1,55E-08 | - | Dentin sialophosphoprotein [Source:UniProtKB/TrEMBL;Acc:O82393] |
| AT2G29670 | 2,08 | 5,66E-111 | - | At2g29670/T27A16.23 [Source:UniProtKB/TrEMBL;Acc:O82388] |
| AT2G29680 | -0,80 | 5,82E-25 | CDC6 | Cell division control protein 6 homolog [Source:UniProtKB/Swiss-Prot;Acc:O82387] |
| AT2G29720 | 2,18 | 4,17E-28 | CTF2B | FAD/NAD(P)-binding oxidoreductase family protein [Source:UniProtKB/TrEMBL;Acc:O82384] |
| AT2G29730 | -1,36 | 4,51E-37 | UGT71D1 | Glycosyltransferase (Fragment) [Source:UniProtKB/TrEMBL;Acc:W8PVN2] |
| AT2G29940 | 2,08 | 4,50E-03 | ABCG31 | ABC transporter G family member 31 [Source:UniProtKB/Swiss-Prot;Acc:Q7PC88] |
| AT2G29960 | -0,82 | 1,06E-58 | CYP19-4 | Peptidyl-prolyl cis-trans isomerase CYP19-4 [Source:UniProtKB/Swiss-Prot;Acc:Q8LDP4] |
| AT2G29970 | 0,65 | 2,68E-11 | SMXL7 | Protein SMAX1-LIKE 7 [Source:UniProtKB/Swiss-Prot;Acc:O80875] |
| AT2G29980 | 0,72 | 8,40E-100 | FAD3 | FAD3 [Source:UniProtKB/TrEMBL;Acc:A0A178VX43] |
| AT2G29995 | 1,42 | 2,39E-37 | PSY3 | Protein PSY3 [Source:UniProtKB/Swiss-Prot;Acc:Q8S8P7] |
| AT2G30040 | 0,98 | 1,25E-14 | MAPKKK14 | Mitogen-activated protein kinase kinase kinase 14 [Source:UniProtKB/TrEMBL;Acc:O64741] |
| AT2G30050 | -0,85 | 1,13E-85 | SEC13B | Protein transport protein SEC13 homolog B [Source:UniProtKB/Swiss-Prot;Acc:O64740] |
| AT2G30060 | -1,30 | 1,00E-103 | RANBP1B | Ran-binding protein 1 homolog b [Source:UniProtKB/Swiss-Prot;Acc:Q8RWG8] |
| AT2G30080 | -1,85 | 2,27E-02 | ZIP6 | ZIP6 [Source:UniProtKB/TrEMBL;Acc:A0A178VN05] |
| AT2G30100 | 0,89 | 1,18E-03 | - | Pentatricopeptide repeat-containing protein At2g30100, chloroplastic [Source:UniProtKB/Swiss-Prot;Acc:Q0WNN7] |
| AT2G30140 | 1,71 | 4,21E-195 | UGT87A2 | UDP-glycosyltransferase 87A2 [Source:UniProtKB/Swiss-Prot;Acc:O64733] |
| AT2G30170 | 0,95 | 1,60E-11 | - | Probable protein phosphatase 2C 26 [Source:UniProtKB/Swiss-Prot;Acc:O64730] |
| AT2G30200 | -0,87 | 5,67E-137 | - | EMBRYO DEFECTIVE 3147 [Source:UniProtKB/TrEMBL;Acc:Q8RU07] |
| AT2G30230 | -1,63 | 2,53E-43 | - | 6,7-dimethyl-8-ribityllumazine synthase [Source:UniProtKB/TrEMBL;Acc:O22919] |
| AT2G30250 | 3,41 | 3,41E-115 | WRKY25 | Probable WRKY transcription factor 25 [Source:UniProtKB/Swiss-Prot;Acc:O22921] |
| AT2G30350 | -0,64 | 3,69E-06 | - | Structure-specific endonuclease subunit SLX1 homolog [Source:UniProtKB/TrEMBL;Acc:A0A178VUD7] |
| AT2G30370 | -1,07 | 3,32E-12 | CHAL | EPFL6 [Source:UniProtKB/TrEMBL;Acc:A0A178VW02] |
| AT2G30380 | -0,90 | 4,94E-10 | - | Plant protein of unknown function (DUF641) [Source:TAIR;Acc:AT2G30380] |
| AT2G30395 | 2,42 | 1,53E-03 | OFP17 | Transcription repressor OFP17 [Source:UniProtKB/Swiss-Prot;Acc:Q84RF2] |
| AT2G30410 | -0,81 | 3,15E-58 | TFCA | Tubulin-folding cofactor A [Source:UniProtKB/Swiss-Prot;Acc:O04350] |
| AT2G30460 | -0,81 | 2,45E-38 | UXT2 | UDP-xylose transporter 2 [Source:UniProtKB/Swiss-Prot;Acc:Q8GUJ1] |
| AT2G30480 | 0,67 | 6,09E-12 | - | unknown protein; Ha. [Source:TAIR;Acc:AT2G30480] |
| AT2G30500 | 0,92 | 1,20E-60 | NET4B | Protein NETWORKED 4B [Source:UniProtKB/Swiss-Prot;Acc:Q84VY2] |
| AT2G30550 | 1,51 | 1,62E-92 | - | Phospholipase A1-Igamma2, chloroplastic [Source:UniProtKB/Swiss-Prot;Acc:Q3EBR6] |
| AT2G30570 | -2,07 | 3,46E-06 | PSBW | PSBW [Source:UniProtKB/TrEMBL;Acc:A0A178VQ32] |
| AT2G30600 | 0,61 | 2,52E-16 | - | BTB/POZ domain-containing protein [Source:UniProtKB/TrEMBL;Acc:F4INV5] |
| AT2G30620 | -0,91 | 1,03E-111 | - | Histone H1.2 [Source:UniProtKB/Swiss-Prot;Acc:P26569] |
| AT2G30660 | -2,94 | 1,34E-03 | - | Probable 3-hydroxyisobutyryl-CoA hydrolase 3 [Source:UniProtKB/Swiss-Prot;Acc:Q6NMB0] |
| AT2G30670 | 1,85 | 4,08E-03 | - | Tropinone reductase homolog At2g30670 [Source:UniProtKB/Swiss-Prot;Acc:O49332] |
| AT2G30680 | 2,29 | 3,49E-13 | - | BEST Arabidopsis thaliana protein match is: glucan synthase-like 3 (TAIR:AT2G31960.2); Ha. [Source:TAIR;Acc:AT2G30680] |
| AT2G30710 | -0,63 | 1,01E-38 | - | Expressed protein [Source:UniProtKB/TrEMBL;Acc:O49336] |
| AT2G30730 | -4,13 | 1,48E-03 | - | Protein kinase superfamily protein [Source:TAIR;Acc:AT2G30730] |
| AT2G30760 | 4,76 | 1,37E-03 | - | Uncharacterized protein At2g30760 [Source:UniProtKB/TrEMBL;Acc:O49341] |
| AT2G30820 | -3,59 | 1,07E-08 | - | Aspartyl/glutamyl-tRNA(Asn/Gln) amidotransferase subunit [Source:UniProtKB/TrEMBL;Acc:Q6NMH7] |
| AT2G30860 | -2,66 | 4,64E-113 | GSTF9 | GSTF9 [Source:UniProtKB/TrEMBL;Acc:A0A178VP05] |
| AT2G30870 | -1,09 | 4,57E-118 | GSTF10 | Glutathione S-transferase F10 [Source:UniProtKB/Swiss-Prot;Acc:P42761] |
| AT2G30890 | -2,05 | 1,31E-27 | - | Cytochrome b561 domain-containing protein At2g30890 [Source:UniProtKB/Swiss-Prot;Acc:O80854] |
| AT2G30942 | -1,11 | 1,01E-09 | - | Protein of unknown function (DUF3317) [Source:TAIR;Acc:AT2G30942] |
| AT2G31081 | -5,26 | 1,72E-04 | CLE4 | CLAVATA3/ESR (CLE)-related protein 4 [Source:UniProtKB/Swiss-Prot;Acc:Q8S8N0] |
| AT2G31083 | -2,49 | 5,92E-05 | CLE5 | CLAVATA3/ESR-RELATED 5 [Source:TAIR;Acc:AT2G31083] |
| AT2G31085 | -3,23 | 1,73E-40 | CLE6 | CLE6 [Source:UniProtKB/TrEMBL;Acc:A0A178VSR3] |
| AT2G31110 | -0,72 | 3,85E-02 | TBL40 | TBL40 [Source:UniProtKB/TrEMBL;Acc:A0A178VPV0] |
| AT2G31150 | 0,67 | 2,16E-13 | - | ATP binding / ATPase [Source:UniProtKB/TrEMBL;Acc:F4IPY0] |
| AT2G31160 | 0,87 | 2,49E-04 | LSH3 | Protein LIGHT-DEPENDENT SHORT HYPOCOTYLS 3 [Source:UniProtKB/Swiss-Prot;Acc:O82268] |
| AT2G31220 | -2,95 | 1,39E-06 | BHLH10 | Transcription factor bHLH10 [Source:UniProtKB/Swiss-Prot;Acc:Q84TK1] |
| AT2G31250 | -1,87 | 7,07E-34 | HEMA3 | Probable glutamyl-tRNA reductase 3, chloroplastic [Source:UniProtKB/Swiss-Prot;Acc:Q9SJX1] |
| AT2G31260 | 0,62 | 2,64E-38 | ATG9 | Autophagy-related protein 9 [Source:UniProtKB/Swiss-Prot;Acc:Q8RUS5] |
| AT2G31270 | -1,22 | 7,46E-63 | CDT1A | CDT1-like protein a, chloroplastic [Source:UniProtKB/Swiss-Prot;Acc:Q9SJW9] |
| AT2G31310 | -4,79 | 6,44E-35 | LBD14 | LOB domain-containing protein 14 [Source:UniProtKB/Swiss-Prot;Acc:Q9SJW5] |
| AT2G31350 | 2,03 | 1,70E-105 | GLX2-5 | Hydroxyacylglutathione hydrolase 2, mitochondrial [Source:UniProtKB/Swiss-Prot;Acc:Q9SID3] |
| AT2G31420 | 2,56 | 6,91E-05 | - | B3 domain-containing protein At2g31420 [Source:UniProtKB/Swiss-Prot;Acc:Q9SIC7] |
| AT2G31425 | 4,34 | 8,81E-04 | - | Plant invertase/pectin methylesterase inhibitor superfamily protein [Source:UniProtKB/TrEMBL;Acc:Q2V441] |
| AT2G31480 | 1,84 | 1,87E-02 | - | Putative uncharacterized protein [Source:UniProtKB/TrEMBL;Acc:Q4PSS8] |
| AT2G31490 | -0,70 | 8,59E-65 | - | AT2G31490 protein [Source:UniProtKB/TrEMBL;Acc:Q9SIQ8] |
| AT2G31540 | -2,51 | 1,23E-09 | - | GDSL esterase/lipase At2g31540 [Source:UniProtKB/Swiss-Prot;Acc:Q9SIQ3] |
| AT2G31560 | 0,71 | 1,18E-06 | - | AT2G31560 protein [Source:UniProtKB/TrEMBL;Acc:Q8VZ71] |
| AT2G31570 | 1,76 | 3,77E-294 | GPX2 | Probable glutathione peroxidase 2 [Source:UniProtKB/Swiss-Prot;Acc:O04922] |
| AT2G31585 | 0,59 | 2,19E-03 | - | other RNA [Source:TAIR;Acc:AT2G31585] |
| AT2G31590 | 2,97 | 1,34E-03 | - | Uncharacterized protein At2g31590 [Source:UniProtKB/TrEMBL;Acc:Q9SIP9] |
| AT2G31600 | 0,92 | 7,32E-16 | - | INO80 complex subunit D-like protein [Source:UniProtKB/TrEMBL;Acc:Q84LR3] |
| AT2G31610 | -0,95 | 3,54E-108 | RPS3A | 40S ribosomal protein S3-1 [Source:UniProtKB/Swiss-Prot;Acc:Q9SIP7] |
| AT2G31670 | -0,71 | 1,60E-78 | UP3 | Stress-response A/B barrel domain-containing protein UP3 [Source:UniProtKB/Swiss-Prot;Acc:Q9SIP1] |
| AT2G31680 | -1,22 | 4,39E-83 | RABA5D | Ras-related protein RABA5d [Source:UniProtKB/Swiss-Prot;Acc:Q9SIP0] |
| AT2G31710 | -0,79 | 7,54E-22 | - | Vacuolar ATPase assembly integral membrane protein VMA21 homolog [Source:UniProtKB/TrEMBL;Acc:Q9SIN7] |
| AT2G31730 | 2,39 | 4,57E-06 | BHLH154 | Transcription factor bHLH154 [Source:UniProtKB/Swiss-Prot;Acc:Q7XJU1] |
| AT2G31830 | 0,99 | 8,84E-11 | - | Endonuclease/exonuclease/phosphatase family protein [Source:UniProtKB/TrEMBL;Acc:F4IRT2] |
| AT2G31840 | 1,20 | 1,03E-33 | MRL7L | Thioredoxin-like fold domain-containing protein MRL7L, chloroplastic [Source:UniProtKB/Swiss-Prot;Acc:Q9SKB6] |
| AT2G31862 | 4,27 | 2,11E-02 | - | B3 domain-containing protein At2g31862 [Source:UniProtKB/Swiss-Prot;Acc:Q6DSS2] |
| AT2G31865 | 1,80 | 2,99E-21 | PARG2 | poly(ADP-ribose) glycohydrolase 2 [Source:TAIR;Acc:AT2G31865] |
| AT2G31880 | -0,87 | 1,86E-18 | SOBIR1 | Leucine-rich repeat receptor-like serine/threonine/tyrosine-protein kinase SOBIR1 [Source:UniProtKB/Swiss-Prot;Acc:Q9SKB2] |
| AT2G31890 | 1,15 | 1,78E-31 | RAP | RAP domain-containing protein, chloroplastic [Source:UniProtKB/Swiss-Prot;Acc:Q8VZE7] |
| AT2G31910 | 2,45 | 4,77E-25 | CHX21 | Cation/H(+) antiporter 21 [Source:UniProtKB/Swiss-Prot;Acc:Q9SKA9] |
| AT2G31940 | -0,89 | 6,27E-05 | - | Oxidoreductase/transition metal ion-binding protein [Source:UniProtKB/TrEMBL;Acc:Q9SKA6] |
| AT2G31970 | 0,86 | 9,14E-105 | RAD50 | DNA repair protein RAD50 [Source:UniProtKB/Swiss-Prot;Acc:Q9SL02] |
| AT2G31990 | 2,35 | 1,37E-07 | - | Exostosin family protein [Source:TAIR;Acc:AT2G31990] |
| AT2G32020 | 3,37 | 0,00E+00 | - | Acyl-CoA N-acyltransferases (NAT) superfamily protein [Source:UniProtKB/TrEMBL;Acc:Q9SKZ7] |
| AT2G32030 | 1,65 | 7,87E-56 | - | Acyl-CoA N-acyltransferases (NAT) superfamily protein [Source:UniProtKB/TrEMBL;Acc:Q9SKZ6] |
| AT2G32060 | -1,01 | 1,76E-125 | RPS12C | 40S ribosomal protein S12 [Source:UniProtKB/TrEMBL;Acc:A0A178VU31] |
| AT2G32090 | 1,25 | 1,51E-19 | - | At2g32090/F22D22.16 [Source:UniProtKB/TrEMBL;Acc:Q9SKZ0] |
| AT2G32120 | 2,67 | 1,88E-55 | HSP70-8 | Heat shock 70 kDa protein 8 [Source:UniProtKB/Swiss-Prot;Acc:Q9SKY8] |
| AT2G32179 | 2,13 | 1,03E-02 | - | other RNA [Source:TAIR;Acc:AT2G32179] |
| AT2G32190 | 0,74 | 1,60E-05 | - | Cysteine-rich/transmembrane domain A-like protein [Source:UniProtKB/TrEMBL;Acc:Q9SKY1] |
| AT2G32240 | -0,69 | 1,98E-55 | - | FUNCTIONS IN: molecular_function unknown; INVOLVED IN: response to cadmium ion; LOCATED IN: plasma membrane; EXPRESSED IN: 25 plant structures; EXPRESSED DURING: 13 growth stages; CONTAINS InterPro DOMAIN/s: Prefoldin (InterPro:IPR009053); BEST Arab /.../s thaliana protein match is: unknown protein (TAIR:AT1G05320.3); Ha. [Source:TAIR;Acc:AT2G32240] |
| AT2G32275 | -0,87 | 3,07E-02 | - | Expressed protein [Source:TAIR;Acc:AT2G32275] |
| AT2G32280 | 0,63 | 2,80E-19 | - | At2g32280 [Source:UniProtKB/TrEMBL;Acc:Q9ZV57] |
| AT2G32300 | -1,82 | 9,16E-04 | UCC1 | uclacyanin 1 [Source:TAIR;Acc:AT2G32300] |
| AT2G32340 | 2,47 | 1,02E-38 | - | TraB family protein [Source:TAIR;Acc:AT2G32340] |
| AT2G32380 | -1,39 | 2,70E-140 | - | At2g32380 [Source:UniProtKB/TrEMBL;Acc:Q9ZV66] |
| AT2G32460 | 2,41 | 7,20E-04 | MYB101 | Transcription factor MYB101 [Source:UniProtKB/Swiss-Prot;Acc:O80883] |
| AT2G32487 | 2,65 | 5,15E-07 | - | unknown protein; FUNCTIONS IN: molecular_function unknown; INVOLVED IN: biological_process unknown; LOCATED IN: endomembrane system; Ha. [Source:TAIR;Acc:AT2G32487] |
| AT2G32500 | -0,64 | 5,80E-05 | - | At2g32500 [Source:UniProtKB/TrEMBL;Acc:Q67XD6] |
| AT2G32510 | 2,32 | 1,34E-03 | MAPKKK17 | Mitogen-activated protein kinase kinase kinase 17 [Source:UniProtKB/Swiss-Prot;Acc:O80888] |
| AT2G32560 | 0,76 | 1,71E-13 | - | F-box protein At2g32560 [Source:UniProtKB/Swiss-Prot;Acc:Q8RY82] |
| AT2G32590 | -0,84 | 4,11E-31 | CAPH | Condensin complex subunit 2 [Source:UniProtKB/Swiss-Prot;Acc:Q564K3] |
| AT2G32610 | -1,67 | 5,73E-67 | CSLB1 | Cellulose synthase-like protein B1 [Source:UniProtKB/Swiss-Prot;Acc:O80898] |
| AT2G32620 | -1,82 | 9,40E-28 | CSLB2 | Cellulose synthase-like protein B2 [Source:UniProtKB/Swiss-Prot;Acc:O80899] |
| AT2G32650 | 0,64 | 4,81E-03 | - | At2g32650 [Source:UniProtKB/TrEMBL;Acc:O48852] |
| AT2G32670 | 1,09 | 2,70E-04 | VAMP725 | Vesicle-associated membrane protein 725 [Source:UniProtKB/Swiss-Prot;Acc:O48850] |
| AT2G32680 | 1,89 | 5,62E-04 | AtRLP23 | Receptor like protein 23 [Source:UniProtKB/Swiss-Prot;Acc:O48849] |
| AT2G32720 | -0,97 | 8,25E-75 | CYTB5-B | CYTB5-D [Source:UniProtKB/TrEMBL;Acc:A0A178VXM5] |
| AT2G32760 | -0,86 | 4,03E-30 | - | At2g32760/F24L7.10 [Source:UniProtKB/TrEMBL;Acc:Q8S9J3] |
| AT2G32780 | 4,05 | 3,14E-02 | UBP1 | Ubiquitin carboxyl-terminal hydrolase 1 [Source:UniProtKB/Swiss-Prot;Acc:Q9FPT5] |
| AT2G32795 | 2,12 | 8,72E-08 | - | other RNA [Source:TAIR;Acc:AT2G32795] |
| AT2G32930 | 0,71 | 2,08E-18 | ZFN2 | zinc finger nuclease 2 [Source:TAIR;Acc:AT2G32930] |
| AT2G32960 | 0,61 | 2,19E-03 | DSP2 | Tyrosine-protein phosphatase DSP2 [Source:UniProtKB/Swiss-Prot;Acc:Q84MD6] |
| AT2G32980 | -0,69 | 1,28E-22 | 37469 | AUGMIN subunit 2 [Source:UniProtKB/Swiss-Prot;Acc:O48767] |
| AT2G32990 | -0,75 | 7,36E-15 | AtGH9B8 | Endoglucanase 11 [Source:UniProtKB/Swiss-Prot;Acc:O48766] |
| AT2G33000 | 1,03 | 8,34E-06 | - | Ubiquitin-associated (UBA)/TS-N domain-containing protein-like protein [Source:UniProtKB/TrEMBL;Acc:F4IUT4] |
| AT2G33051 | 0,86 | 1,06E-02 | - | other RNA [Source:TAIR;Acc:AT2G33051] |
| AT2G33060 | 0,76 | 8,00E-09 | AtRLP27 | Receptor like protein 27 [Source:UniProtKB/Swiss-Prot;Acc:F4IUU1] |
| AT2G33100 | 1,24 | 3,19E-11 | CSLD1 | Cellulose synthase-like protein D1 [Source:UniProtKB/Swiss-Prot;Acc:O49323] |
| AT2G33120 | -0,59 | 1,02E-43 | SAR1 | AT2G33120 protein [Source:UniProtKB/TrEMBL;Acc:B9DH97] |
| AT2G33205 | 1,14 | 1,26E-03 | - | Serinc-domain containing serine and sphingolipid biosynthesis protein [Source:TAIR;Acc:AT2G33205] |
| AT2G33220 | -0,65 | 7,09E-43 | - | GRIM-19 protein [Source:TAIR;Acc:AT2G33220] |
| AT2G33250 | 2,03 | 1,15E-140 | - | At2g33250/F25I18.1 [Source:UniProtKB/TrEMBL;Acc:O22777] |
| AT2G33260 | -1,10 | 1,27E-07 | - | Tryptophan/tyrosine permease [Source:TAIR;Acc:AT2G33260] |
| AT2G33310 | 0,75 | 3,19E-16 | IAA13 | Auxin-responsive protein IAA13 [Source:UniProtKB/Swiss-Prot;Acc:Q38831] |
| AT2G33330 | 0,75 | 5,18E-12 | CRRSP11 | Cysteine-rich repeat secretory protein 11 [Source:UniProtKB/Swiss-Prot;Acc:O22784] |
| AT2G33340 | 0,88 | 2,38E-131 | PRP19B | Pre-mRNA-processing factor 19 homolog 2 [Source:UniProtKB/Swiss-Prot;Acc:O22785] |
| AT2G33470 | -0,61 | 2,90E-55 | GLTP1 | Glycolipid transfer protein 1 [Source:UniProtKB/Swiss-Prot;Acc:O22797] |
| AT2G33480 | 1,16 | 1,49E-04 | NAC041 | NAC domain-containing protein 41 [Source:UniProtKB/Swiss-Prot;Acc:O22798] |
| AT2G33530 | -3,28 | 1,12E-108 | SCPL46 | Serine carboxypeptidase-like 46 [Source:UniProtKB/Swiss-Prot;Acc:Q8VY01] |
| AT2G33540 | -0,69 | 3,08E-16 | CPL3 | RNA polymerase II C-terminal domain phosphatase-like 3 [Source:UniProtKB/Swiss-Prot;Acc:Q8LL04] |
| AT2G33550 | 0,89 | 6,08E-19 | ASR3 | Trihelix transcription factor ASR3 [Source:UniProtKB/Swiss-Prot;Acc:Q8VZ20] |
| AT2G33560 | -0,71 | 7,71E-11 | BUBR1 | BUBR1 [Source:UniProtKB/TrEMBL;Acc:A0A178VU51] |
| AT2G33580 | 0,95 | 1,50E-20 | LYK5 | Protein LYK5 [Source:UniProtKB/Swiss-Prot;Acc:O22808] |
| AT2G33590 | 1,05 | 2,99E-96 | - | CRL1 [Source:UniProtKB/TrEMBL;Acc:A0A178VRE7] |
| AT2G33610 | 0,71 | 1,46E-28 | SWI3B | SWI/SNF complex subunit SWI3B [Source:UniProtKB/Swiss-Prot;Acc:Q84JG2] |
| AT2G33710 | -0,84 | 1,15E-21 | - | Integrase-type DNA-binding superfamily protein [Source:UniProtKB/TrEMBL;Acc:F4IFX1] |
| AT2G33735 | 0,76 | 1,79E-05 | - | Chaperone DnaJ-domain superfamily protein [Source:UniProtKB/TrEMBL;Acc:Q8RYC5] |
| AT2G33740 | 1,25 | 4,58E-83 | CUTA | Protein CutA, chloroplastic [Source:UniProtKB/Swiss-Prot;Acc:P93009] |
| AT2G33760 | 0,72 | 3,48E-06 | PCMP-H6 | Pentatricopeptide repeat-containing protein At2g33760 [Source:UniProtKB/Swiss-Prot;Acc:P93011] |
| AT2G33790 | -3,41 | 1,45E-18 | AGP30 | Non-classical arabinogalactan protein 30 [Source:UniProtKB/Swiss-Prot;Acc:P93013] |
| AT2G33793 | -0,79 | 1,28E-09 | - | unknown protein; Ha. [Source:TAIR;Acc:AT2G33793] |
| AT2G33860 | -2,15 | 8,11E-70 | ARF3 | Auxin response factor 3 [Source:UniProtKB/Swiss-Prot;Acc:O23661] |
| AT2G33880 | -6,24 | 2,53E-07 | WOX9 | WOX9A [Source:UniProtKB/TrEMBL;Acc:A0A178VQA8] |
| AT2G33900 | 2,19 | 1,08E-02 | - | pre-tRNA [Source:TAIR;Acc:AT2G33900] |
| AT2G33910 | 2,58 | 6,01E-03 | - | pre-tRNA [Source:TAIR;Acc:AT2G33910] |
| AT2G33980 | 0,77 | 1,86E-10 | atnudt22 | nudix hydrolase homolog 22 [Source:TAIR;Acc:AT2G33980] |
| AT2G34010 | -1,51 | 2,59E-02 | SPEAR2 | Protein SPEAR2 [Source:UniProtKB/Swiss-Prot;Acc:F4IGU3] |
| AT2G34050 | -0,75 | 2,11E-30 | - | ATP synthase F1 complex assembly factor [Source:UniProtKB/TrEMBL;Acc:O22958] |
| AT2G34080 | 1,52 | 1,53E-83 | - | Cysteine proteinases superfamily protein [Source:UniProtKB/TrEMBL;Acc:O22961] |
| AT2G34150 | -1,18 | 1,43E-90 | SCAR1 | Protein SCAR1 [Source:UniProtKB/Swiss-Prot;Acc:Q6AWX6] |
| AT2G34170 | -0,66 | 2,55E-26 | - | Uncharacterized protein At2g34170 [Source:UniProtKB/TrEMBL;Acc:Q8RYE0] |
| AT2G34180 | -0,71 | 6,14E-04 | CIPK13 | CBL-interacting serine/threonine-protein kinase 13 [Source:UniProtKB/Swiss-Prot;Acc:O22971] |
| AT2G34185 | -0,69 | 1,08E-02 | - | unknown protein; Ha. [Source:TAIR;Acc:AT2G34185] |
| AT2G34190 | -1,31 | 6,69E-70 | NAT2 | Nucleobase-ascorbate transporter 2 [Source:UniProtKB/Swiss-Prot;Acc:Q94C70] |
| AT2G34220 | 0,99 | 4,28E-07 | MEE20 | Protein with domains of unknown function (DUF627 and DUF629) [Source:TAIR;Acc:AT2G34220] |
| AT2G34224 | 0,96 | 2,77E-02 | - | unknown protein; LOCATED IN: endomembrane system; Ha. [Source:TAIR;Acc:AT2G34224] |
| AT2G34230 | 1,20 | 2,61E-08 | - | Ubiquitin carboxyl-terminal hydrolase-like protein, putative (DUF627 and DUF629) [Source:UniProtKB/TrEMBL;Acc:O80772] |
| AT2G34240 | 0,61 | 4,45E-05 | - | Protein with domains of unknown function DUF627 and DUF632 [Source:TAIR;Acc:AT2G34240] |
| AT2G34250 | -1,12 | 1,78E-263 | - | AT2G34250 protein [Source:UniProtKB/TrEMBL;Acc:O80774] |
| AT2G34300 | -0,78 | 1,46E-42 | - | Probable methyltransferase PMT25 [Source:UniProtKB/Swiss-Prot;Acc:Q0WT31] |
| AT2G34325 | 0,67 | 3,20E-04 | - | - |
| AT2G34340 | 0,99 | 1,10E-20 | - | At2g34340 [Source:UniProtKB/TrEMBL;Acc:O80783] |
| AT2G34355 | -1,39 | 2,56E-33 | - | At2g34355 [Source:UniProtKB/TrEMBL;Acc:Q501E0] |
| AT2G34360 | 3,34 | 9,06E-06 | DTX15 | Protein DETOXIFICATION 15 [Source:UniProtKB/Swiss-Prot;Acc:F4IHU9] |
| AT2G34370 | 1,41 | 1,08E-03 | PCMP-H25 | Pentatricopeptide repeat-containing protein At2g34370, mitochondrial [Source:UniProtKB/Swiss-Prot;Acc:Q8S8Q7] |
| AT2G34390 | 1,85 | 2,53E-07 | NIP2-1 | Aquaporin NIP2-1 [Source:UniProtKB/Swiss-Prot;Acc:Q8W037] |
| AT2G34400 | 0,65 | 4,92E-05 | PCMP-E23 | Pentatricopeptide repeat-containing protein At2g34400 [Source:UniProtKB/Swiss-Prot;Acc:O64705] |
| AT2G34410 | -0,71 | 5,21E-29 | RWA3 | Protein REDUCED WALL ACETYLATION 3 [Source:UniProtKB/Swiss-Prot;Acc:Q66GQ5] |
| AT2G34450 | 0,72 | 4,89E-10 | - | HMG-box (high mobility group) DNA-binding family protein [Source:TAIR;Acc:AT2G34450] |
| AT2G34460 | 1,82 | 3,01E-68 | - | Uncharacterized protein At2g34460, chloroplastic [Source:UniProtKB/Swiss-Prot;Acc:Q8H124] |
| AT2G34480 | -0,79 | 2,28E-166 | - | Ribosomal protein L18ae/LX family protein [Source:TAIR;Acc:AT2G34480] |
| AT2G34500 | 2,52 | 4,95E-33 | CYP710A1 | CYP710A1 [Source:UniProtKB/TrEMBL;Acc:A0A178VRK5] |
| AT2G34510 | 1,66 | 3,44E-03 | - | Uncharacterized protein At2g34510 [Source:UniProtKB/TrEMBL;Acc:O64696] |
| AT2G34580 | -1,64 | 1,85E-03 | - | CONTAINS InterPro DOMAIN/s: Herpesvirus UL139, cytomegalovirus (InterPro:IPR021042); Ha. [Source:TAIR;Acc:AT2G34580] |
| AT2G34585 | -0,69 | 1,44E-21 | - | At2g34585 [Source:UniProtKB/TrEMBL;Acc:Q8S8R9] |
| AT2G34690 | 0,81 | 3,94E-68 | ACD11 | Accelerated cell death 11 [Source:UniProtKB/Swiss-Prot;Acc:O64587] |
| AT2G34790 | -0,64 | 5,40E-05 | MEE23 | Berberine bridge enzyme-like 15 [Source:UniProtKB/Swiss-Prot;Acc:O64743] |
| AT2G34860 | 1,05 | 2,62E-28 | PSA2 | Protein PHOTOSYSTEM I ASSEMBLY 2, chloroplastic [Source:UniProtKB/Swiss-Prot;Acc:O64750] |
| AT2G34900 | 0,89 | 2,87E-89 | GTE1 | Transcription factor GTE1 [Source:UniProtKB/Swiss-Prot;Acc:Q84XV2] |
| AT2G34910 | -3,09 | 1,85E-09 | - | At2g34910 [Source:UniProtKB/TrEMBL;Acc:O64755] |
| AT2G34920 | -1,43 | 4,16E-54 | EDA18 | RING/U-box superfamily protein [Source:TAIR;Acc:AT2G34920] |
| AT2G34940 | -1,88 | 4,61E-10 | VSR5 | VSR5 [Source:UniProtKB/TrEMBL;Acc:A0A384KPH5] |
| AT2G34960 | 1,30 | 3,15E-02 | CAT5 | CAT5 [Source:UniProtKB/TrEMBL;Acc:A0A178VPZ9] |
| AT2G35020 | -0,82 | 9,33E-43 | GLCNAC1PUT2 | UDP-N-acetylglucosamine diphosphorylase 2 [Source:UniProtKB/Swiss-Prot;Acc:O64765] |
| AT2G35120 | -0,74 | 2,48E-108 | GDH2 | Glycine cleavage system H protein 2, mitochondrial [Source:UniProtKB/Swiss-Prot;Acc:O82179] |
| AT2G35140 | 1,11 | 1,06E-27 | - | DCD (Development and Cell Death) domain protein [Source:TAIR;Acc:AT2G35140] |
| AT2G35190 | -0,92 | 1,69E-53 | NPSN11 | Novel plant SNARE 11 [Source:UniProtKB/Swiss-Prot;Acc:Q944A9] |
| AT2G35200 | 1,11 | 1,13E-11 | - | DUF740 family protein [Source:UniProtKB/TrEMBL;Acc:O82172] |
| AT2G35260 | -2,96 | 5,17E-11 | - | At2g35260/T4C15.7 [Source:UniProtKB/TrEMBL;Acc:O82167] |
| AT2G35300 | 4,27 | 6,90E-04 | LEA18 | Late embryogenesis abundant protein 18 [Source:UniProtKB/Swiss-Prot;Acc:Q96273] |
| AT2G35310 | -1,25 | 4,44E-04 | - | B3 domain-containing protein At2g35310 [Source:UniProtKB/Swiss-Prot;Acc:Q5PNU4] |
| AT2G35343 | 1,03 | 9,32E-04 | - | - |
| AT2G35345 | 1,02 | 2,68E-04 | - | Putative uncharacterized protein [Source:UniProtKB/TrEMBL;Acc:Q1G3S2] |
| AT2G35370 | -2,08 | 1,29E-23 | GDH1 | Glycine cleavage system H protein [Source:UniProtKB/TrEMBL;Acc:A0A178VV72] |
| AT2G35410 | 0,73 | 1,18E-11 | - | Putative chloroplast RNA binding protein [Source:UniProtKB/TrEMBL;Acc:O82299] |
| AT2G35430 | 0,67 | 3,83E-07 | - | Zinc finger CCCH domain-containing protein 28 [Source:UniProtKB/Swiss-Prot;Acc:Q5PP65] |
| AT2G35480 | 1,66 | 4,72E-183 | - | Envelope glycoprotein [Source:UniProtKB/TrEMBL;Acc:Q8VZ11] |
| AT2G35540 | 0,81 | 3,77E-11 | - | DNAJ heat shock N-terminal domain-containing protein [Source:UniProtKB/TrEMBL;Acc:F4IKR5] |
| AT2G35550 | 2,27 | 7,96E-03 | BPC7 | BPC7 [Source:UniProtKB/TrEMBL;Acc:A0A178W000] |
| AT2G35610 | -0,86 | 1,36E-139 | XEG113 | XEG113 [Source:UniProtKB/TrEMBL;Acc:A0A178W347] |
| AT2G35650 | -0,79 | 2,96E-15 | CSLA7 | Glucomannan 4-beta-mannosyltransferase 7 [Source:UniProtKB/Swiss-Prot;Acc:Q9ZQN8] |
| AT2G35680 | 0,60 | 1,35E-18 | DSP8 | Putative dual specificity protein phosphatase DSP8 [Source:UniProtKB/Swiss-Prot;Acc:Q9ZQP1] |
| AT2G35700 | 2,22 | 5,33E-24 | ERF038 | ERF38 [Source:UniProtKB/TrEMBL;Acc:A0A178VWJ3] |
| AT2G35730 | 0,65 | 3,40E-02 | - | At2g35730 [Source:UniProtKB/TrEMBL;Acc:Q9ZQP5] |
| AT2G35736 | 1,81 | 1,90E-53 | - | Uncharacterized protein At2g35736 [Source:UniProtKB/TrEMBL;Acc:Q8S8K5] |
| AT2G35742 | 0,96 | 2,01E-22 | - | snoRNA [Source:TAIR;Acc:AT2G35742] |
| AT2G35800 | -0,88 | 2,36E-55 | - | Mitochondrial substrate carrier family protein [Source:UniProtKB/TrEMBL;Acc:Q8VZP7] |
| AT2G35820 | 2,39 | 4,45E-05 | - | ureidoglycolate hydrolases [Source:TAIR;Acc:AT2G35820] |
| AT2G35830 | 2,99 | 4,35E-02 | - | At2g35830/F11F19.26 [Source:UniProtKB/TrEMBL;Acc:Q9SJ67] |
| AT2G35900 | 1,62 | 5,73E-143 | - | Mal d 1-associated protein [Source:UniProtKB/TrEMBL;Acc:Q9SJ60] |
| AT2G35910 | 0,93 | 1,84E-13 | ATL70 | RING-H2 finger protein ATL70 [Source:UniProtKB/Swiss-Prot;Acc:Q8RX29] |
| AT2G35940 | 4,37 | 5,18E-07 | BLH1 | BEL1-like homeodomain protein 1 [Source:UniProtKB/Swiss-Prot;Acc:Q9SJ56] |
| AT2G36000 | 0,61 | 2,21E-05 | - | At2g36000/F11F19.9 [Source:UniProtKB/TrEMBL;Acc:Q9SJ50] |
| AT2G36090 | -1,98 | 2,63E-21 | - | Probable F-box protein At2g36090 [Source:UniProtKB/Swiss-Prot;Acc:Q9SIH5] |
| AT2G36120 | -2,88 | 2,65E-10 | DOT1 | Glycine-rich protein DOT1 [Source:UniProtKB/Swiss-Prot;Acc:Q9SIH2] |
| AT2G36145 | 0,78 | 3,84E-03 | - | Expressed protein [Source:UniProtKB/TrEMBL;Acc:Q8VXY0] |
| AT2G36200 | -0,80 | 2,09E-29 | - | P-loop containing nucleoside triphosphate hydrolases superfamily protein [Source:UniProtKB/TrEMBL;Acc:F4ILV6] |
| AT2G36220 | 2,23 | 1,85E-04 | - | At2g36220/F2H17.17 [Source:UniProtKB/TrEMBL;Acc:Q9SJN3] |
| AT2G36250 | 0,96 | 2,63E-90 | FTSZ2-1 | Cell division protein FtsZ homolog 2-1, chloroplastic [Source:UniProtKB/Swiss-Prot;Acc:O82533] |
| AT2G36270 | 1,64 | 2,28E-21 | ABI5 | Basic-leucine zipper (bZIP) transcription factor family protein [Source:TAIR;Acc:AT2G36270] |
| AT2G36300 | -0,86 | 4,80E-62 | - | Protein YIPF [Source:UniProtKB/TrEMBL;Acc:Q9SJM8] |
| AT2G36307 | -0,60 | 5,02E-04 | ZPR4 | ZPR4 (LITTLE ZIPPER 4); other RNA [Source:TAIR;Acc:AT2G36307] |
| AT2G36310 | -0,63 | 2,31E-17 | URH1 | Uridine nucleosidase 1 [Source:UniProtKB/Swiss-Prot;Acc:Q9SJM7] |
| AT2G36330 | 0,59 | 9,29E-07 | - | CASP-like protein 4A3 [Source:UniProtKB/Swiss-Prot;Acc:Q84WP5] |
| AT2G36340 | 0,79 | 3,09E-30 | GPL3 | GLABROUS1 enhancer-binding protein-like 3 [Source:UniProtKB/Swiss-Prot;Acc:Q9SJM4] |
| AT2G36430 | -5,07 | 1,20E-04 | - | Transmembrane protein, putative (DUF247) [Source:UniProtKB/TrEMBL;Acc:Q9SJR2] |
| AT2G36460 | 5,14 | 0,00E+00 | FBA6 | Fructose-bisphosphate aldolase 6, cytosolic [Source:UniProtKB/Swiss-Prot;Acc:Q9SJQ9] |
| AT2G36490 | -0,86 | 3,16E-23 | ROS1 | Protein ROS1 [Source:UniProtKB/Swiss-Prot;Acc:Q9SJQ6] |
| AT2G36540 | 3,53 | 2,22E-02 | - | Haloacid dehalogenase-like hydrolase (HAD) superfamily protein [Source:UniProtKB/TrEMBL;Acc:Q9SJQ4] |
| AT2G36620 | -1,13 | 1,18E-124 | RPL24A | 60S ribosomal protein L24-1 [Source:UniProtKB/Swiss-Prot;Acc:Q42347] |
| AT2G36630 | 1,35 | 4,72E-40 | - | Sulfite exporter TauE/SafE family protein 4 [Source:UniProtKB/Swiss-Prot;Acc:Q8S9J0] |
| AT2G36650 | 1,16 | 1,07E-02 | - | CHUP1-like protein [Source:UniProtKB/TrEMBL;Acc:Q5BPR9] |
| AT2G36660 | 1,12 | 2,84E-08 | PAB7 | Polyadenylate-binding protein 7 [Source:UniProtKB/Swiss-Prot;Acc:Q9ZQA8] |
| AT2G36690 | -1,73 | 1,10E-35 | - | 2-oxoglutarate (2OG) and Fe(II)-dependent oxygenase superfamily protein [Source:UniProtKB/TrEMBL;Acc:F4INZ9] |
| AT2G36780 | 1,43 | 2,82E-16 | UGT73C3 | UDP-glycosyltransferase 73C3 [Source:UniProtKB/Swiss-Prot;Acc:Q9ZQ96] |
| AT2G36800 | 7,17 | 9,34E-289 | UGT73C5 | Glycosyltransferase (Fragment) [Source:UniProtKB/TrEMBL;Acc:W8Q3B1] |
| AT2G36830 | 0,80 | 1,35E-08 | TIP1-1 | Aquaporin TIP1-1 [Source:UniProtKB/Swiss-Prot;Acc:P25818] |
| AT2G36840 | 0,67 | 4,25E-07 | ACR10 | ACT domain-containing protein ACR10 [Source:UniProtKB/Swiss-Prot;Acc:Q9SJM1] |
| AT2G36870 | 1,85 | 1,17E-09 | XTH32 | Probable xyloglucan endotransglucosylase/hydrolase protein 32 [Source:UniProtKB/Swiss-Prot;Acc:Q9SJL9] |
| AT2G36880 | -1,15 | 1,10E-227 | METK3 | S-adenosylmethionine synthase [Source:UniProtKB/TrEMBL;Acc:A0A178VXF8] |
| AT2G36930 | 0,66 | 5,86E-45 | - | Expressed protein [Source:UniProtKB/TrEMBL;Acc:Q9SJL4] |
| AT2G36950 | 1,81 | 3,14E-54 | HIPP05 | Heavy metal-associated isoprenylated plant protein 5 [Source:UniProtKB/Swiss-Prot;Acc:Q9SJL2] |
| AT2G36970 | 3,92 | 4,66E-02 | UGT86A1 | Glycosyltransferase (Fragment) [Source:UniProtKB/TrEMBL;Acc:W8Q6X6] |
| AT2G36980 | 0,61 | 1,88E-06 | PCMP-E73 | Pentatricopeptide repeat-containing protein At2g36980, mitochondrial [Source:UniProtKB/Swiss-Prot;Acc:Q9SJK9] |
| AT2G36985 | 2,48 | 1,21E-02 | ROT4 | At2g36985 [Source:UniProtKB/TrEMBL;Acc:Q7XXN8] |
| AT2G36990 | -0,64 | 3,12E-12 | SIGF | RNA polymerase sigma factor sigF, chloroplastic [Source:UniProtKB/Swiss-Prot;Acc:Q9LD95] |
| AT2G37000 | -0,60 | 2,35E-02 | TCP11 | Transcription factor TCP11 [Source:UniProtKB/Swiss-Prot;Acc:Q9SJK7] |
| AT2G37040 | -0,89 | 5,29E-15 | PAL1 | Phenylalanine ammonia-lyase 1 [Source:UniProtKB/Swiss-Prot;Acc:P35510] |
| AT2G37060 | 0,62 | 1,15E-10 | NFYB8 | Nuclear transcription factor Y subunit B-8 [Source:UniProtKB/Swiss-Prot;Acc:Q8VYK4] |
| AT2G37130 | -1,23 | 7,39E-10 | PER21 | Peroxidase 21 [Source:UniProtKB/Swiss-Prot;Acc:Q42580] |
| AT2G37150 | 1,17 | 1,80E-36 | - | RING/U-box superfamily protein [Source:UniProtKB/TrEMBL;Acc:F4IQ07] |
| AT2G37170 | -0,70 | 2,36E-07 | PIP2B | plasma membrane intrinsic protein 2 [Source:TAIR;Acc:AT2G37170] |
| AT2G37200 | 1,13 | 1,92E-29 | - | Uncharacterised protein family (UPF0497) [Source:TAIR;Acc:AT2G37200] |
| AT2G37240 | 1,05 | 1,19E-21 | - | Thioredoxin-like protein AAED1, chloroplastic [Source:UniProtKB/Swiss-Prot;Acc:Q9ZUU2] |
| AT2G37270 | -0,72 | 6,55E-104 | RPS5A | 40S ribosomal protein S5-1 [Source:UniProtKB/Swiss-Prot;Acc:Q9ZUT9] |
| AT2G37290 | -0,63 | 1,57E-05 | - | Ypt/Rab-GAP domain of gyp1p superfamily protein [Source:UniProtKB/TrEMBL;Acc:F4IQ29] |
| AT2G37300 | -0,82 | 1,58E-11 | - | unknown protein; Ha. [Source:TAIR;Acc:AT2G37300] |
| AT2G37310 | 0,62 | 8,22E-19 | PCMP-E49 | Pentatricopeptide repeat-containing protein At2g37310 [Source:UniProtKB/Swiss-Prot;Acc:Q9ZUT5] |
| AT2G37330 | -0,93 | 6,91E-10 | ALS3 | ALS3 [Source:UniProtKB/TrEMBL;Acc:A0A178VRI6] |
| AT2G37340 | 1,59 | 3,61E-220 | RS2Z33 | Serine/arginine-rich splicing factor RS2Z33 [Source:UniProtKB/Swiss-Prot;Acc:Q8VYA5] |
| AT2G37370 | 2,07 | 3,02E-04 | - | unknown protein; BEST Arabidopsis thaliana protein match is: unknown protein (TAIR:AT5G13560.1); Ha. [Source:TAIR;Acc:AT2G37370] |
| AT2G37380 | -0,75 | 5,03E-09 | MAKR3 | Probable membrane-associated kinase regulator 3 [Source:UniProtKB/Swiss-Prot;Acc:Q9ZUS8] |
| AT2G37410 | 0,91 | 6,32E-75 | TIM17-2 | Mitochondrial import inner membrane translocase subunit TIM17-2 [Source:UniProtKB/Swiss-Prot;Acc:Q9SP35] |
| AT2G37440 | -2,97 | 3,04E-54 | IP5P8 | Type I inositol polyphosphate 5-phosphatase 8 [Source:UniProtKB/Swiss-Prot;Acc:Q0WT19] |
| AT2G37450 | -2,17 | 1,90E-63 | - | WAT1-related protein [Source:UniProtKB/TrEMBL;Acc:A0A178W1D2] |
| AT2G37460 | 3,73 | 6,12E-03 | - | WAT1-related protein At2g37460 [Source:UniProtKB/Swiss-Prot;Acc:Q9ZUS1] |
| AT2G37470 | -1,05 | 1,49E-61 | - | Histone H2B.4 [Source:UniProtKB/Swiss-Prot;Acc:Q9ZUS0] |
| AT2G37510 | 1,17 | 2,30E-59 | - | RNA-binding (RRM/RBD/RNP motifs) family protein [Source:TAIR;Acc:AT2G37510] |
| AT2G37540 | -1,13 | 1,24E-44 | - | NAD(P)-binding Rossmann-fold superfamily protein [Source:UniProtKB/TrEMBL;Acc:O80924] |
| AT2G37550 | -0,65 | 4,42E-52 | AGD7 | ADP-ribosylation factor GTPase-activating protein AGD7 [Source:UniProtKB/Swiss-Prot;Acc:O80925] |
| AT2G37555 | -2,13 | 1,39E-02 | - | other RNA [Source:TAIR;Acc:AT2G37555] |
| AT2G37560 | -0,89 | 4,20E-12 | ORC2 | Origin of replication complex subunit 2 [Source:UniProtKB/Swiss-Prot;Acc:Q38899] |
| AT2G37660 | -0,78 | 6,14E-17 | - | Uncharacterized protein At2g37660, chloroplastic [Source:UniProtKB/Swiss-Prot;Acc:O80934] |
| AT2G37690 | 0,62 | 6,79E-55 | - | Phosphoribosylaminoimidazole carboxylase like protein [Source:UniProtKB/TrEMBL;Acc:Q84TI2] |
| AT2G37700 | -0,99 | 3,92E-16 | - | Fatty acid hydroxylase superfamily [Source:TAIR;Acc:AT2G37700] |
| AT2G37740 | -4,89 | 9,88E-09 | ZFP10 | Zinc finger protein 10 [Source:UniProtKB/Swiss-Prot;Acc:O80942] |
| AT2G37750 | 1,72 | 1,51E-02 | - | Expressed protein [Source:UniProtKB/TrEMBL;Acc:O80943] |
| AT2G37760 | 1,07 | 1,22E-156 | AKR4C8 | Aldo-keto reductase family 4 member C8 [Source:UniProtKB/Swiss-Prot;Acc:O80944] |
| AT2G37790 | 0,60 | 3,28E-33 | AKR4C10 | Aldo-keto reductase family 4 member C10 [Source:UniProtKB/Swiss-Prot;Acc:Q84TF0] |
| AT2G37820 | -4,32 | 2,15E-02 | - | At2g37820 [Source:UniProtKB/TrEMBL;Acc:Q6NM27] |
| AT2G37890 | 0,77 | 1,93E-33 | - | Mitochondrial carrier like protein [Source:UniProtKB/TrEMBL;Acc:Q8L7R0] |
| AT2G37920 | 0,93 | 2,85E-05 | emb1513 | Copper ion transmembrane transporter [Source:UniProtKB/TrEMBL;Acc:Q8LG21] |
| AT2G37925 | -2,26 | 4,80E-02 | COPT4 | Copper transporter 4 [Source:UniProtKB/Swiss-Prot;Acc:Q8SAA5] |
| AT2G37930 | -0,93 | 5,86E-04 | - | Protein of unknown function (DUF3527) [Source:TAIR;Acc:AT2G37930] |
| AT2G37940 | -0,81 | 7,02E-113 | IPCS2 | ERH1 [Source:UniProtKB/TrEMBL;Acc:A0A178VV03] |
| AT2G37960 | -1,29 | 2,45E-22 | - | Myosin-M heavy protein [Source:UniProtKB/TrEMBL;Acc:Q5S4W1] |
| AT2G37975 | -1,14 | 4,98E-50 | - | At2g37975 [Source:UniProtKB/TrEMBL;Acc:Q8LG42] |
| AT2G37980 | -0,94 | 5,40E-42 | OFUT19 | O-fucosyltransferase 19 [Source:UniProtKB/Swiss-Prot;Acc:Q9SH89] |
| AT2G38040 | -1,14 | 1,78E-117 | CAC3 | Acetyl-coenzyme A carboxylase carboxyl transferase subunit alpha, chloroplastic [Source:UniProtKB/Swiss-Prot;Acc:Q9LD43] |
| AT2G38060 | -1,02 | 9,25E-36 | PHT4;2 | phosphate transporter 4;2 [Source:TAIR;Acc:AT2G38060] |
| AT2G38100 | 1,90 | 2,55E-02 | - | proton-dependent oligopeptide transport (POT) family protein [Source:TAIR;Acc:AT2G38100] |
| AT2G38140 | -0,72 | 6,73E-28 | RPS31 | 30S ribosomal protein S31, chloroplastic [Source:UniProtKB/Swiss-Prot;Acc:O80439] |
| AT2G38152 | -3,08 | 4,18E-08 | - | Alpha 1,4-glycosyltransferase family protein [Source:UniProtKB/TrEMBL;Acc:F4IS01] |
| AT2G38160 | -0,66 | 3,41E-09 | - | unknown protein; BEST Arabidopsis thaliana protein match is: unknown protein (TAIR:AT2G40070.2); Ha. [Source:TAIR;Acc:AT2G38160] |
| AT2G38180 | -0,69 | 1,28E-27 | - | GDSL esterase/lipase At2g38180 [Source:UniProtKB/Swiss-Prot;Acc:O80443] |
| AT2G38185 | 0,64 | 1,72E-04 | - | RING/U-box superfamily protein [Source:UniProtKB/TrEMBL;Acc:F4IS08] |
| AT2G38210 | 1,56 | 2,63E-10 | PDX1L4 | Pyridoxal 5'-phosphate synthase PDX1-like 4 [Source:UniProtKB/Swiss-Prot;Acc:O80446] |
| AT2G38230 | 2,19 | 5,93E-89 | PDX11 | Pyridoxal 5'-phosphate synthase subunit PDX1.1 [Source:UniProtKB/Swiss-Prot;Acc:O80448] |
| AT2G38250 | 2,89 | 2,88E-26 | GT-3B | Trihelix transcription factor GT-3b [Source:UniProtKB/Swiss-Prot;Acc:O80450] |
| AT2G38255 | 3,05 | 2,23E-07 | - | Protein of Unknown Function (DUF239) [Source:TAIR;Acc:AT2G38255] |
| AT2G38270 | 0,59 | 4,28E-16 | GRXS16 | Bifunctional monothiol glutaredoxin-S16, chloroplastic [Source:UniProtKB/Swiss-Prot;Acc:Q8H7F6] |
| AT2G38300 | -0,90 | 5,32E-16 | - | Myb-like HTH transcriptional regulator family protein [Source:UniProtKB/TrEMBL;Acc:Q8GYE4] |
| AT2G38310 | 1,03 | 4,86E-86 | PYL4 | Abscisic acid receptor PYL4 [Source:UniProtKB/Swiss-Prot;Acc:O80920] |
| AT2G38320 | 0,67 | 3,53E-04 | TBL34 | Protein trichome birefringence-like 34 [Source:UniProtKB/Swiss-Prot;Acc:O80919] |
| AT2G38340 | 2,38 | 3,17E-09 | DREB2E | Dehydration-responsive element-binding protein 2E [Source:UniProtKB/Swiss-Prot;Acc:O80917] |
| AT2G38360 | -0,73 | 4,01E-40 | PRA1B4 | PRA1 family protein B4 [Source:UniProtKB/Swiss-Prot;Acc:O80915] |
| AT2G38370 | 0,93 | 9,48E-22 | - | Plant protein of unknown function (DUF827) [Source:TAIR;Acc:AT2G38370] |
| AT2G38400 | 1,65 | 1,19E-18 | AGT3 | Alanine:glyoxylate aminotransferase 3 [Source:UniProtKB/TrEMBL;Acc:F4ISY3] |
| AT2G38430 | 0,96 | 1,95E-09 | - | Putative uncharacterized protein [Source:UniProtKB/TrEMBL;Acc:Q58FY7] |
| AT2G38465 | 2,02 | 1,38E-03 | - | Expressed protein [Source:UniProtKB/TrEMBL;Acc:Q8S8P4] |
| AT2G38490 | -3,58 | 1,48E-09 | CIPK22 | CBL-interacting serine/threonine-protein kinase 22 [Source:UniProtKB/Swiss-Prot;Acc:O80902] |
| AT2G38500 | -3,49 | 1,00E-12 | - | 2-oxoglutarate (2OG) and Fe(II)-dependent oxygenase superfamily protein [Source:UniProtKB/TrEMBL;Acc:Q8GY13] |
| AT2G38510 | -3,50 | 4,10E-07 | DTX53 | Protein DETOXIFICATION 53 [Source:UniProtKB/Swiss-Prot;Acc:Q9ZVH5] |
| AT2G38530 | 1,91 | 4,10E-02 | LTP2 | Non-specific lipid-transfer protein [Source:UniProtKB/TrEMBL;Acc:A0A178W063] |
| AT2G38580 | -0,59 | 6,93E-30 | - | Mitochondrial ATP synthase D chain-related protein [Source:UniProtKB/TrEMBL;Acc:Q682E1] |
| AT2G38620 | -0,90 | 4,26E-23 | CDKB1-2 | Cyclin-dependent kinase B1-2 [Source:UniProtKB/Swiss-Prot;Acc:Q2V419] |
| AT2G38650 | -0,62 | 5,40E-52 | GAUT7 | Probable galacturonosyltransferase 7 [Source:UniProtKB/Swiss-Prot;Acc:Q9ZVI7] |
| AT2G38670 | -0,69 | 2,36E-52 | PECT1 | Ethanolamine-phosphate cytidylyltransferase [Source:UniProtKB/Swiss-Prot;Acc:Q9ZVI9] |
| AT2G38720 | -1,60 | 7,88E-18 | MAP65-5 | 65-kDa microtubule-associated protein 5 [Source:UniProtKB/Swiss-Prot;Acc:Q9ZVJ3] |
| AT2G38750 | -2,36 | 6,69E-27 | ANN4 | Annexin D4 [Source:UniProtKB/Swiss-Prot;Acc:Q9ZVJ6] |
| AT2G38760 | -1,06 | 5,46E-31 | ANN3 | Annexin [Source:UniProtKB/TrEMBL;Acc:A0A178W040] |
| AT2G38800 | 0,98 | 2,77E-16 | - | Plant calmodulin-binding protein-like protein [Source:UniProtKB/TrEMBL;Acc:Q9SII1] |
| AT2G38823 | 5,93 | 0,00E+00 | - | Putative uncharacterized protein [Source:UniProtKB/TrEMBL;Acc:Q5S4V8] |
| AT2G38830 | -0,87 | 3,81E-04 | - | Ubiquitin-conjugating enzyme/RWD-like protein [Source:UniProtKB/TrEMBL;Acc:F4ITY4] |
| AT2G38860 | -1,37 | 1,17E-121 | DJ1E | DJ-1 protein homolog E [Source:UniProtKB/Swiss-Prot;Acc:Q9ZV19] |
| AT2G38870 | -1,83 | 1,43E-66 | - | Putative protease inhibitor [Source:UniProtKB/TrEMBL;Acc:Q9ZV18] |
| AT2G38960 | -0,78 | 1,12E-41 | ERO2 | Endoplasmic reticulum oxidoreductins 2 [Source:UniProtKB/TrEMBL;Acc:F4IU09] |
| AT2G39000 | 0,67 | 4,28E-07 | - | Acyl-CoA N-acyltransferases (NAT) superfamily protein [Source:UniProtKB/TrEMBL;Acc:Q9ZV08] |
| AT2G39010 | -1,66 | 2,68E-43 | PIP2-6 | Probable aquaporin PIP2-6 [Source:UniProtKB/Swiss-Prot;Acc:Q9ZV07] |
| AT2G39040 | -0,93 | 1,59E-07 | PER24 | Peroxidase 24 [Source:UniProtKB/Swiss-Prot;Acc:Q9ZV04] |
| AT2G39080 | 0,66 | 7,05E-34 | - | NAD(P)-binding Rossmann-fold superfamily protein [Source:UniProtKB/TrEMBL;Acc:Q8VZB1] |
| AT2G39110 | 0,71 | 6,87E-28 | - | Protein kinase superfamily protein [Source:UniProtKB/TrEMBL;Acc:Q0WSF6] |
| AT2G39130 | -0,62 | 8,30E-47 | AVT1C | Amino acid transporter AVT1C [Source:UniProtKB/Swiss-Prot;Acc:F4IUW3] |
| AT2G39175 | 1,62 | 3,11E-02 | MIR160A | MIR160/MIR160A; miRNA [Source:TAIR;Acc:AT2G39175] |
| AT2G39180 | -0,64 | 4,06E-35 | CCR2 | Serine/threonine-protein kinase-like protein CCR2 [Source:UniProtKB/Swiss-Prot;Acc:O80963] |
| AT2G39230 | 1,61 | 1,53E-34 | LOJ | Pentatricopeptide repeat-containing protein At2g39230, mitochondrial [Source:UniProtKB/Swiss-Prot;Acc:O80958] |
| AT2G39290 | -0,60 | 1,54E-20 | PGPS1 | PGS1 [Source:UniProtKB/TrEMBL;Acc:A0A178VXZ6] |
| AT2G39300 | -0,73 | 2,26E-41 | - | unknown protein; INVOLVED IN: biological_process unknown; LOCATED IN: vacuole; EXPRESSED IN: 8 plant structures; EXPRESSED DURING: 4 anthesis, petal differentiation and expansion stage; BEST Arabidopsis thaliana protein match is: unknown protein (TA /.../G55060.1); Ha. [Source:TAIR;Acc:AT2G39300] |
| AT2G39310 | 5,09 | 5,32E-81 | JAL22 | Jacalin-related lectin 22 [Source:UniProtKB/Swiss-Prot;Acc:O80950] |
| AT2G39350 | 1,21 | 1,97E-63 | ABCG1 | ABC transporter G family member 1 [Source:UniProtKB/Swiss-Prot;Acc:O80946] |
| AT2G39390 | -0,89 | 1,02E-83 | RPL35B | 60S ribosomal protein L35-2 [Source:UniProtKB/Swiss-Prot;Acc:O80626] |
| AT2G39400 | -5,48 | 7,05E-09 | - | Alpha/beta-Hydrolases superfamily protein [Source:UniProtKB/TrEMBL;Acc:O80627] |
| AT2G39410 | -1,89 | 1,83E-13 | - | Alpha/beta-Hydrolases superfamily protein [Source:UniProtKB/TrEMBL;Acc:O80628] |
| AT2G39415 | 4,31 | 8,94E-03 | - | Putative F-box protein At2g39415 [Source:UniProtKB/Swiss-Prot;Acc:Q3EBJ8] |
| AT2G39435 | -1,27 | 1,98E-12 | - | Phosphatidylinositol N-acetyglucosaminlytransferase subunit P-like protein [Source:UniProtKB/TrEMBL;Acc:Q0WT61] |
| AT2G39460 | -0,99 | 1,59E-138 | RPL23AA | 60S ribosomal protein L23a-1 [Source:UniProtKB/Swiss-Prot;Acc:Q8LD46] |
| AT2G39500 | -0,85 | 2,38E-20 | - | At2g39500/F12L6.16 [Source:UniProtKB/TrEMBL;Acc:O80637] |
| AT2G39530 | -2,99 | 8,75E-15 | - | CASP-like protein 4D1 [Source:UniProtKB/Swiss-Prot;Acc:Q8GWD5] |
| AT2G39570 | 1,65 | 4,81E-32 | ACR9 | ACT domain-containing protein ACR9 [Source:UniProtKB/Swiss-Prot;Acc:O80644] |
| AT2G39650 | 1,16 | 5,53E-25 | - | At2g39650 [Source:UniProtKB/TrEMBL;Acc:O48813] |
| AT2G39660 | 1,00 | 3,71E-23 | BIK1 | BIK1 [Source:UniProtKB/TrEMBL;Acc:A0A178VYW0] |
| AT2G39681 | 1,20 | 2,93E-79 | TAS2 | TAS2; other RNA [Source:TAIR;Acc:AT2G39681] |
| AT2G39690 | 2,05 | 6,69E-06 | - | Protein of unknown function, DUF547 [Source:TAIR;Acc:AT2G39690] |
| AT2G39700 | 0,63 | 5,17E-29 | EXPA4 | Expansin-A4 [Source:UniProtKB/Swiss-Prot;Acc:O48818] |
| AT2G39720 | -0,60 | 1,14E-22 | RHC2A | Probable E3 ubiquitin-protein ligase RHC2A [Source:UniProtKB/Swiss-Prot;Acc:O22283] |
| AT2G39730 | 0,84 | 3,30E-09 | RCA | Ribulose bisphosphate carboxylase/oxygenase activase, chloroplastic [Source:UniProtKB/Swiss-Prot;Acc:P10896] |
| AT2G39800 | -1,71 | 0,00E+00 | P5CSA | Delta-1-pyrroline-5-carboxylate synthase A [Source:UniProtKB/Swiss-Prot;Acc:P54887] |
| AT2G39820 | 3,24 | 2,55E-09 | EIF6-1 | Eukaryotic translation initiation factor 6-1 [Source:UniProtKB/Swiss-Prot;Acc:O22290] |
| AT2G39890 | 0,74 | 2,05E-10 | PROT1 | Proline transporter 1 [Source:UniProtKB/Swiss-Prot;Acc:P92961] |
| AT2G39960 | -1,05 | 6,54E-108 | - | Probable signal peptidase complex subunit 2 [Source:UniProtKB/Swiss-Prot;Acc:P58684] |
| AT2G40020 | 1,41 | 1,05E-05 | - | Nucleolar histone methyltransferase-related protein [Source:TAIR;Acc:AT2G40020] |
| AT2G40080 | 2,21 | 6,20E-150 | ELF4 | Protein EARLY FLOWERING 4 [Source:UniProtKB/Swiss-Prot;Acc:O04211] |
| AT2G40085 | 1,29 | 1,41E-18 | - | unknown protein; Ha. [Source:TAIR;Acc:AT2G40085] |
| AT2G40095 | 0,86 | 3,58E-11 | - | Alpha/beta hydrolase related protein [Source:UniProtKB/TrEMBL;Acc:Q8GZ10] |
| AT2G40100 | 3,00 | 3,45E-03 | LHCB4.3 | Chlorophyll a-b binding protein, chloroplastic [Source:UniProtKB/TrEMBL;Acc:A0A178VTY4] |
| AT2G40140 | 1,70 | 2,29E-155 | CZF1 | Zinc finger CCCH domain-containing protein 29 [Source:UniProtKB/Swiss-Prot;Acc:Q9XEE6] |
| AT2G40150 | -0,60 | 9,79E-08 | TBL28 | Protein trichome birefringence-like 28 [Source:UniProtKB/Swiss-Prot;Acc:Q94K00] |
| AT2G40200 | 0,74 | 4,54E-02 | BHLH51 | Transcription factor bHLH51 [Source:UniProtKB/Swiss-Prot;Acc:Q9XEF0] |
| AT2G40240 | 0,78 | 6,02E-13 | - | Pentatricopeptide repeat-containing protein At2g40240, mitochondrial [Source:UniProtKB/Swiss-Prot;Acc:Q9S733] |
| AT2G40250 | 1,38 | 1,11E-16 | - | GDSL esterase/lipase At2g40250 [Source:UniProtKB/Swiss-Prot;Acc:Q9SIZ6] |
| AT2G40270 | 1,05 | 1,77E-30 | - | Inactive receptor-like serine/threonine-protein kinase At2g40270 [Source:UniProtKB/Swiss-Prot;Acc:Q9SIZ4] |
| AT2G40300 | -0,85 | 1,80E-29 | FER4 | Ferritin-4, chloroplastic [Source:UniProtKB/Swiss-Prot;Acc:Q9S756] |
| AT2G40330 | 0,83 | 3,48E-30 | PYL6 | Abscisic acid receptor PYL6 [Source:UniProtKB/Swiss-Prot;Acc:Q8S8E3] |
| AT2G40340 | 3,43 | 4,55E-66 | DREB2C | Integrase-type DNA-binding superfamily protein [Source:TAIR;Acc:AT2G40340] |
| AT2G40450 | 0,58 | 2,97E-03 | - | Putative BTB/POZ domain-containing protein At2g40450 [Source:UniProtKB/Swiss-Prot;Acc:O22890] |
| AT2G40480 | -1,92 | 1,35E-69 | - | WEB family protein At2g40480 [Source:UniProtKB/Swiss-Prot;Acc:Q5XVC7] |
| AT2G40490 | -1,33 | 3,94E-62 | HEME2 | Uroporphyrinogen decarboxylase 2, chloroplastic [Source:UniProtKB/Swiss-Prot;Acc:O22886] |
| AT2G40510 | -0,65 | 3,25E-53 | RPS26B | 40S ribosomal protein S26 [Source:UniProtKB/TrEMBL;Acc:A0A178VTX1] |
| AT2G40570 | 0,68 | 8,32E-22 | - | At2g40570 [Source:UniProtKB/TrEMBL;Acc:Q7Y230] |
| AT2G40610 | 0,74 | 1,40E-05 | EXPA8 | Expansin-A8 [Source:UniProtKB/Swiss-Prot;Acc:O22874] |
| AT2G40710 | 1,02 | 5,25E-06 | - | Hemolysin-III related integral membrane protein [Source:UniProtKB/TrEMBL;Acc:F4II26] |
| AT2G40711 | 0,93 | 2,31E-05 | - | Putative uncharacterized protein [Source:UniProtKB/TrEMBL;Acc:Q1G3R8] |
| AT2G40730 | -0,74 | 1,16E-80 | - | Kinase family with ARM repeat domain-containing protein [Source:UniProtKB/TrEMBL;Acc:F4II29] |
| AT2G40750 | -1,49 | 6,81E-15 | WRKY54 | Probable WRKY transcription factor 54 [Source:UniProtKB/Swiss-Prot;Acc:Q93WU8] |
| AT2G40790 | 0,96 | 2,28E-03 | CXXS2 | Thioredoxin-like protein CXXS2 [Source:UniProtKB/Swiss-Prot;Acc:Q8GXV2] |
| AT2G40810 | 0,77 | 4,09E-09 | ATG18C | Autophagy-related protein 18c [Source:UniProtKB/Swiss-Prot;Acc:Q8GYD7] |
| AT2G40830 | 1,06 | 2,46E-55 | RHC1A | Probable E3 ubiquitin-protein ligase RHC1A [Source:UniProtKB/Swiss-Prot;Acc:O22197] |
| AT2G40880 | -1,99 | 8,36E-03 | CYS3 | Cysteine proteinase inhibitor [Source:UniProtKB/TrEMBL;Acc:A0A178VXG7] |
| AT2G40890 | 0,66 | 1,88E-19 | CYP98A3 | Cytochrome P450 98A3 [Source:UniProtKB/Swiss-Prot;Acc:O22203] |
| AT2G40900 | -2,75 | 8,24E-25 | - | WAT1-related protein At2g40900 [Source:UniProtKB/Swiss-Prot;Acc:F4IJ08] |
| AT2G40920 | 1,06 | 5,14E-07 | - | F-box/LRR-repeat protein At2g40920 [Source:UniProtKB/Swiss-Prot;Acc:Q2V414] |
| AT2G40955 | 6,97 | 3,40E-10 | - | Uncharacterized protein At2g40955 [Source:UniProtKB/TrEMBL;Acc:Q8S8T7] |
| AT2G41070 | 1,53 | 1,46E-89 | EEL | Basic-leucine zipper (bZIP) transcription factor family protein [Source:TAIR;Acc:AT2G41070] |
| AT2G41082 | -0,65 | 2,19E-02 | - | unknown protein; LOCATED IN: endomembrane system; Ha. [Source:TAIR;Acc:AT2G41082] |
| AT2G41100 | -1,25 | 9,32E-10 | CML12 | Calmodulin-like protein 12 [Source:UniProtKB/Swiss-Prot;Acc:P25071] |
| AT2G41110 | -1,60 | 6,19E-136 | CAM2 | Calmodulin 2 [Source:UniProtKB/TrEMBL;Acc:F4IJ46] |
| AT2G41120 | 2,97 | 2,10E-02 | - | At2g41120 [Source:UniProtKB/TrEMBL;Acc:O80675] |
| AT2G41130 | 0,93 | 9,60E-16 | BHLH106 | Transcription factor bHLH106 [Source:UniProtKB/Swiss-Prot;Acc:O80674] |
| AT2G41140 | 1,31 | 5,25E-116 | CRK1 | CDPK-related kinase 1 [Source:UniProtKB/Swiss-Prot;Acc:O80673] |
| AT2G41160 | 0,69 | 4,21E-35 | RBL18 | Rhomboid-like protein 18 [Source:UniProtKB/Swiss-Prot;Acc:Q8RXQ2] |
| AT2G41170 | 0,88 | 5,14E-11 | - | F-box family protein [Source:TAIR;Acc:AT2G41170] |
| AT2G41200 | 0,78 | 3,55E-09 | - | Expressed protein [Source:UniProtKB/TrEMBL;Acc:O80667] |
| AT2G41210 | 1,96 | 7,98E-48 | PIP5K5 | Phosphatidylinositol 4-phosphate 5-kinase 5 [Source:UniProtKB/Swiss-Prot;Acc:Q9SLG9] |
| AT2G41220 | -0,81 | 4,78E-66 | GLU2 | Ferredoxin-dependent glutamate synthase 2, chloroplastic [Source:UniProtKB/Swiss-Prot;Acc:Q9T0P4] |
| AT2G41290 | 0,68 | 5,42E-27 | SSL2 | Protein STRICTOSIDINE SYNTHASE-LIKE 2 [Source:UniProtKB/Swiss-Prot;Acc:Q9SLG8] |
| AT2G41330 | -1,24 | 3,14E-11 | - | At2g41330/F13H10.12 [Source:UniProtKB/TrEMBL;Acc:Q9ZVB8] |
| AT2G41380 | 1,36 | 1,77E-70 | - | At2g41380 [Source:UniProtKB/TrEMBL;Acc:Q9ZVC3] |
| AT2G41445 | 5,07 | 1,40E-05 | - | unknown protein; FUNCTIONS IN: molecular_function unknown; INVOLVED IN: biological_process unknown; LOCATED IN: cellular_component unknown; BEST Arabidopsis thaliana protein match is: unknown protein (TAIR:AT2G41470.1); Ha. [Source:TAIR;Acc:AT2G41445] |
| AT2G41460 | 0,66 | 1,82E-08 | ARP | DNA-(apurinic or apyrimidinic site) lyase, chloroplastic [Source:UniProtKB/Swiss-Prot;Acc:P45951] |
| AT2G41470 | 2,09 | 8,60E-04 | - | unknown protein; FUNCTIONS IN: molecular_function unknown; INVOLVED IN: biological_process unknown; LOCATED IN: vacuole; BEST Arabidopsis thaliana protein match is: unknown protein (TAIR:AT2G41440.1); Ha. [Source:TAIR;Acc:AT2G41470] |
| AT2G41500 | 0,74 | 3,12E-56 | LIS | LIS [Source:UniProtKB/TrEMBL;Acc:A0A178VMW2] |
| AT2G41505 | 0,81 | 1,18E-04 | - | - |
| AT2G41640 | 0,61 | 3,18E-07 | - | At2g41640/T32G6.16 [Source:UniProtKB/TrEMBL;Acc:O22225] |
| AT2G41670 | 0,67 | 2,59E-33 | SIN2 | Short integuments 2, mitochondrial [Source:UniProtKB/Swiss-Prot;Acc:Q8L607] |
| AT2G41700 | 0,67 | 3,13E-20 | ABCA1 | ABC transporter A family member 1 [Source:UniProtKB/Swiss-Prot;Acc:Q84M24] |
| AT2G41730 | 5,62 | 0,00E+00 | - | Calcium-binding site protein [Source:UniProtKB/TrEMBL;Acc:O22947] |
| AT2G41740 | -0,80 | 1,50E-42 | VLN2 | VLN2 [Source:UniProtKB/TrEMBL;Acc:A0A178VT62] |
| AT2G41770 | -0,58 | 1,78E-30 | STL1 | Probable glycosyltransferase STELLO1 [Source:UniProtKB/Swiss-Prot;Acc:O22943] |
| AT2G41810 | -4,05 | 1,69E-34 | - | Uncharacterized protein At2g41810 [Source:UniProtKB/TrEMBL;Acc:O22939] |
| AT2G41820 | -0,96 | 1,37E-42 | PXC3 | Leucine-rich repeat receptor-like tyrosine-protein kinase PXC3 [Source:UniProtKB/Swiss-Prot;Acc:O22938] |
| AT2G41835 | 0,78 | 1,27E-17 | SAP11 | Zinc finger AN1 and C2H2 domain-containing stress-associated protein 11 [Source:UniProtKB/Swiss-Prot;Acc:Q8VZ42] |
| AT2G41970 | -5,10 | 1,83E-34 | - | Probable protein kinase At2g41970 [Source:UniProtKB/Swiss-Prot;Acc:P93749] |
| AT2G42040 | 0,66 | 2,06E-13 | - | Expressed protein [Source:UniProtKB/TrEMBL;Acc:P93743] |
| AT2G42060 | -3,30 | 6,45E-03 | - | At2g42060 [Source:UniProtKB/TrEMBL;Acc:P93741] |
| AT2G42065 | 7,00 | 1,03E-245 | - | DnaJ domain protein [Source:UniProtKB/TrEMBL;Acc:A0A1P8AYV4] |
| AT2G42110 | -1,08 | 9,29E-31 | - | At2g42110 [Source:UniProtKB/TrEMBL;Acc:O23683] |
| AT2G42150 | -1,52 | 1,37E-07 | - | DNA-binding bromodomain-containing protein [Source:UniProtKB/TrEMBL;Acc:O48523] |
| AT2G42170 | -1,97 | 4,39E-18 | - | Actin family protein [Source:UniProtKB/TrEMBL;Acc:Q8GWA5] |
| AT2G42190 | -0,84 | 1,65E-27 | - | Expressed protein [Source:UniProtKB/TrEMBL;Acc:O48526] |
| AT2G42200 | -2,59 | 2,27E-04 | SPL9 | Squamosa promoter-binding-like protein 9 [Source:UniProtKB/Swiss-Prot;Acc:Q700W2] |
| AT2G42220 | -1,63 | 9,49E-03 | STR9 | Rhodanese-like domain-containing protein 9, chloroplastic [Source:UniProtKB/Swiss-Prot;Acc:O48529] |
| AT2G42230 | -1,06 | 8,13E-139 | - | At2g42230 [Source:UniProtKB/TrEMBL;Acc:Q66GP5] |
| AT2G42247 | 0,75 | 2,19E-04 | - | other RNA [Source:TAIR;Acc:AT2G42247] |
| AT2G42260 | -1,48 | 6,28E-29 | PYM | Protein POLYCHOME [Source:UniProtKB/Swiss-Prot;Acc:O48533] |
| AT2G42270 | 0,67 | 4,42E-45 | BRR2B | DExH-box ATP-dependent RNA helicase DExH13 [Source:UniProtKB/Swiss-Prot;Acc:O48534] |
| AT2G42280 | 0,93 | 4,34E-21 | - | basic helix-loop-helix (bHLH) DNA-binding superfamily protein [Source:TAIR;Acc:AT2G42280] |
| AT2G42310 | -0,75 | 1,63E-60 | - | ESSS subunit of NADH:ubiquinone oxidoreductase (Complex I) protein [Source:UniProtKB/TrEMBL;Acc:Q9SLC8] |
| AT2G42320 | -0,70 | 2,07E-13 | - | Nucleolar protein gar2-like protein [Source:UniProtKB/TrEMBL;Acc:Q9SLC7] |
| AT2G42330 | 1,11 | 4,41E-37 | STIPL2 | Septin and tuftelin-interacting protein 1 homolog 2 [Source:UniProtKB/Swiss-Prot;Acc:Q9SLC6] |
| AT2G42350 | 1,46 | 4,90E-04 | ATL40 | RING-H2 finger protein ATL40 [Source:UniProtKB/Swiss-Prot;Acc:Q9SLC4] |
| AT2G42395 | 0,79 | 3,47E-10 | - | At2g42395 [Source:UniProtKB/TrEMBL;Acc:Q84TF7] |
| AT2G42450 | 0,65 | 1,82E-14 | - | Alpha/beta-Hydrolases superfamily protein [Source:UniProtKB/TrEMBL;Acc:F4IN30] |
| AT2G42480 | -3,20 | 4,18E-02 | - | MATH domain and coiled-coil domain-containing protein At2g42480 [Source:UniProtKB/Swiss-Prot;Acc:P0DKG7] |
| AT2G42485 | -1,01 | 1,20E-07 | - | other RNA [Source:TAIR;Acc:AT2G42485] |
| AT2G42510 | 0,65 | 1,52E-03 | - | FUNCTIONS IN: molecular_function unknown; INVOLVED IN: spliceosome assembly, nuclear mRNA splicing, via spliceosome; LOCATED IN: cellular_component unknown; CONTAINS InterPro DOMAIN/s: Survival motor neuron interacting protein 1 (InterPro:IPR007022) /.../ Arabidopsis thaliana protein match is: spliceosome protein-related (TAIR:AT1G54380.1); Ha. [Source:TAIR;Acc:AT2G42510] |
| AT2G42600 | 1,11 | 5,94E-105 | PPC2 | PPC2 [Source:UniProtKB/TrEMBL;Acc:A0A178VV64] |
| AT2G42650 | 0,80 | 1,51E-28 | - | Ribosomal protein L1p/L10e family [Source:UniProtKB/TrEMBL;Acc:Q8S8D2] |
| AT2G42660 | 1,77 | 3,78E-119 | - | At2g45350/F14N22.7 [Source:UniProtKB/TrEMBL;Acc:Q9SJJ0] |
| AT2G42720 | 1,17 | 7,61E-41 | - | F-box/LRR-repeat protein At2g42720 [Source:UniProtKB/Swiss-Prot;Acc:Q6DR13] |
| AT2G42750 | 0,95 | 1,22E-24 | - | At2g42750/F7D19.25 [Source:UniProtKB/TrEMBL;Acc:Q9SJI1] |
| AT2G42800 | 1,19 | 7,35E-03 | AtRLP29 | RLP29 [Source:UniProtKB/TrEMBL;Acc:A0A178VT48] |
| AT2G42850 | -3,93 | 1,10E-33 | CYP718 | CYP718 [Source:UniProtKB/TrEMBL;Acc:A0A178VUF2] |
| AT2G42870 | -1,02 | 2,84E-15 | PAR1 | Transcription factor PAR1 [Source:UniProtKB/Swiss-Prot;Acc:Q9SJH0] |
| AT2G42900 | -3,67 | 1,30E-34 | - | At2g42900 [Source:UniProtKB/TrEMBL;Acc:Q9SJG7] |
| AT2G42920 | 0,93 | 1,78E-27 | PCMP-E75 | Pentatricopeptide repeat-containing protein At2g42920, chloroplastic [Source:UniProtKB/Swiss-Prot;Acc:Q9SJG6] |
| AT2G42950 | 0,73 | 1,23E-14 | - | At2g42950 [Source:UniProtKB/TrEMBL;Acc:Q8GY73] |
| AT2G42955 | 2,30 | 1,50E-21 | - | unknown protein; BEST Arabidopsis thaliana protein match is: unknown protein (TAIR:AT2G30615.1); Ha. [Source:TAIR;Acc:AT2G42955] |
| AT2G42980 | 1,35 | 4,68E-05 | - | Eukaryotic aspartyl protease family protein [Source:UniProtKB/TrEMBL;Acc:Q9SJG1] |
| AT2G43050 | -1,84 | 1,35E-07 | PME16 | Probable pectinesterase/pectinesterase inhibitor 16 [Source:UniProtKB/Swiss-Prot;Acc:Q9SKX2] |
| AT2G43100 | 1,85 | 4,93E-05 | IPMI2 | IPMI2 [Source:UniProtKB/TrEMBL;Acc:A0A178VZE1] |
| AT2G43130 | -1,06 | 1,06E-98 | RABA5C | Ras-related protein RABA5c [Source:UniProtKB/Swiss-Prot;Acc:P28187] |
| AT2G43180 | 0,74 | 4,29E-11 | - | Phosphoenolpyruvate carboxylase family protein [Source:UniProtKB/TrEMBL;Acc:Q8GYI4] |
| AT2G43240 | -0,85 | 1,55E-50 | - | CMP-sialic acid transporter 2 [Source:UniProtKB/Swiss-Prot;Acc:Q8GY97] |
| AT2G43250 | 0,67 | 1,23E-09 | - | Transmembrane protein [Source:UniProtKB/TrEMBL;Acc:Q9ZW70] |
| AT2G43310 | -0,64 | 3,31E-02 | - | F14B2.25/F14B2.25 [Source:UniProtKB/TrEMBL;Acc:O22846] |
| AT2G43360 | -0,77 | 1,46E-63 | BIO2 | Biotin synthase, mitochondrial [Source:UniProtKB/Swiss-Prot;Acc:P54967] |
| AT2G43370 | 0,65 | 3,63E-17 | SNRNP35 | U11/U12 small nuclear ribonucleoprotein 35 kDa protein [Source:UniProtKB/Swiss-Prot;Acc:Q8VY74] |
| AT2G43375 | 1,20 | 8,81E-19 | - | other RNA [Source:TAIR;Acc:AT2G43375] |
| AT2G43420 | 0,71 | 1,78E-38 | 3BETAHSD/D3 | 3beta-hydroxysteroid-dehydrogenase/decarboxylase isoform 3 [Source:UniProtKB/Swiss-Prot;Acc:A9X4U2] |
| AT2G43445 | 1,54 | 5,94E-20 | - | F-box/kelch-repeat protein At2g43445 [Source:UniProtKB/Swiss-Prot;Acc:Q0WRU9] |
| AT2G43450 | -2,50 | 2,92E-02 | - | Uncharacterized protein At2g43450 [Source:UniProtKB/TrEMBL;Acc:O22859] |
| AT2G43470 | -1,27 | 1,32E-15 | - | Protein of unknown function (DUF3755) [Source:TAIR;Acc:AT2G43470] |
| AT2G43490 | -0,59 | 2,83E-45 | - | Ypt/Rab-GAP domain of gyp1p superfamily protein [Source:UniProtKB/TrEMBL;Acc:B3H765] |
| AT2G43530 | -1,41 | 1,48E-02 | ATTI3 | Defensin-like protein 194 [Source:UniProtKB/Swiss-Prot;Acc:O22867] |
| AT2G43550 | -1,53 | 1,39E-02 | ATTI6 | Defensin-like protein 197 [Source:UniProtKB/Swiss-Prot;Acc:O22869] |
| AT2G43570 | 4,18 | 8,68E-133 | CHI | CHI [Source:UniProtKB/TrEMBL;Acc:A0A178W3M3] |
| AT2G43610 | 0,81 | 3,92E-44 | - | Endochitinase At2g43610 [Source:UniProtKB/Swiss-Prot;Acc:O22842] |
| AT2G43620 | 1,57 | 1,73E-05 | - | Endochitinase At2g43620 [Source:UniProtKB/Swiss-Prot;Acc:O22841] |
| AT2G43640 | -0,79 | 2,09E-53 | SRP14 | Signal recognition particle 14 kDa protein [Source:UniProtKB/Swiss-Prot;Acc:O04421] |
| AT2G43660 | 3,67 | 4,71E-131 | - | Carbohydrate-binding X8 domain superfamily protein [Source:UniProtKB/TrEMBL;Acc:F4IS25] |
| AT2G43670 | -4,36 | 1,48E-03 | - | At2g43670 [Source:UniProtKB/TrEMBL;Acc:Q6IDB5] |
| AT2G43720 | 2,14 | 2,91E-126 | - | At2g43720/F18O19.17 [Source:UniProtKB/TrEMBL;Acc:O22831] |
| AT2G43790 | -0,65 | 1,09E-56 | MPK6 | Mitogen-activated protein kinase [Source:UniProtKB/TrEMBL;Acc:A0A178VTX8] |
| AT2G43820 | 1,29 | 4,27E-25 | UGT74F2 | UDP-glycosyltransferase 74F2 [Source:UniProtKB/Swiss-Prot;Acc:O22822] |
| AT2G43840 | -0,65 | 1,51E-02 | UGT74F1 | Glycosyltransferase [Source:UniProtKB/TrEMBL;Acc:F4IS54] |
| AT2G43870 | -1,11 | 2,02E-30 | - | Pectin lyase-like superfamily protein [Source:UniProtKB/TrEMBL;Acc:O22817] |
| AT2G43880 | -1,93 | 8,06E-26 | - | Pectin lyase-like superfamily protein [Source:UniProtKB/TrEMBL;Acc:Q9SLM8] |
| AT2G43890 | -4,26 | 9,79E-24 | - | Pectin lyase-like superfamily protein [Source:UniProtKB/TrEMBL;Acc:O80559] |
| AT2G43930 | 0,90 | 6,09E-03 | - | Protein kinase superfamily protein [Source:TAIR;Acc:AT2G43930] |
| AT2G43940 | -0,60 | 7,30E-22 | HOL3 | HOL3 [Source:UniProtKB/TrEMBL;Acc:A0A178VV10] |
| AT2G43960 | 6,76 | 1,00E-09 | - | At2g43960 [Source:UniProtKB/TrEMBL;Acc:Q58FX2] |
| AT2G44010 | 0,80 | 1,23E-04 | - | At2g44010 [Source:UniProtKB/TrEMBL;Acc:O80571] |
| AT2G44060 | -1,50 | 2,89E-144 | - | At2g44060 [Source:UniProtKB/TrEMBL;Acc:O80576] |
| AT2G44110 | -5,82 | 2,89E-29 | MLO15 | MLO-like protein [Source:UniProtKB/TrEMBL;Acc:A0A178VQX0] |
| AT2G44160 | -0,76 | 3,97E-75 | MTHFR2 | Methylenetetrahydrofolate reductase 2 [Source:UniProtKB/Swiss-Prot;Acc:O80585] |
| AT2G44210 | -1,47 | 1,68E-12 | - | AT2G44210 protein [Source:UniProtKB/TrEMBL;Acc:B9DGK6] |
| AT2G44230 | -5,25 | 1,94E-05 | - | At2g44230/F4I1.4 [Source:UniProtKB/TrEMBL;Acc:O64858] |
| AT2G44290 | 2,65 | 1,06E-34 | YLS3 | Protein YLS3 [Source:UniProtKB/Swiss-Prot;Acc:O64864] |
| AT2G44310 | -0,93 | 6,28E-34 | - | Calcium-binding EF-hand family protein [Source:UniProtKB/TrEMBL;Acc:O64866] |
| AT2G44360 | -0,66 | 1,15E-18 | - | Ecotropic viral integration site protein [Source:UniProtKB/TrEMBL;Acc:O64870] |
| AT2G44370 | -3,29 | 3,11E-08 | - | At2g44370 [Source:UniProtKB/TrEMBL;Acc:O64871] |
| AT2G44410 | 0,66 | 3,87E-23 | - | At2g44410 [Source:UniProtKB/TrEMBL;Acc:Q6NLR3] |
| AT2G44460 | 7,36 | 1,07E-13 | BGLU28 | Beta-glucosidase 28 [Source:UniProtKB/Swiss-Prot;Acc:Q4V3B3] |
| AT2G44590 | 1,83 | 2,16E-02 | DRP1D | At2g44590 [Source:UniProtKB/TrEMBL;Acc:B5X4Z5] |
| AT2G44610 | -0,93 | 6,44E-116 | RABH1B | Ras-related protein RABH1b [Source:UniProtKB/Swiss-Prot;Acc:O80501] |
| AT2G44620 | -0,59 | 2,41E-55 | MTACP1 | Acyl carrier protein 1, mitochondrial [Source:UniProtKB/Swiss-Prot;Acc:P53665] |
| AT2G44630 | 0,63 | 4,87E-04 | - | F-box/kelch-repeat protein At2g44630 [Source:UniProtKB/Swiss-Prot;Acc:O80502] |
| AT2G44670 | -1,34 | 9,13E-72 | FLZ3 | FCS-Like Zinc finger 3 [Source:UniProtKB/Swiss-Prot;Acc:O80506] |
| AT2G44690 | -1,07 | 1,22E-04 | ARAC9 | ROP8 [Source:UniProtKB/TrEMBL;Acc:A0A384LBU4] |
| AT2G44735 | -0,60 | 2,91E-05 | - | BEST Arabidopsis thaliana protein match is: F-box family protein (TAIR:AT3G18720.1); Ha. [Source:TAIR;Acc:AT2G44735] |
| AT2G44740 | -1,94 | 2,67E-130 | CYCU4-1 | Cyclin-U4-1 [Source:UniProtKB/Swiss-Prot;Acc:O80513] |
| AT2G44750 | 0,73 | 9,30E-18 | TPK2 | Thiamine pyrophosphokinase 2 [Source:UniProtKB/Swiss-Prot;Acc:F4IV16] |
| AT2G44850 | -0,62 | 9,83E-04 | - | Uncharacterized protein At2g44850 [Source:UniProtKB/TrEMBL;Acc:O22166] |
| AT2G44880 | 0,87 | 1,21E-10 | - | Pentatricopeptide repeat (PPR-like) superfamily protein [Source:TAIR;Acc:AT2G44880] |
| AT2G44900 | 0,75 | 5,11E-37 | FBX5 | Protein ARABIDILLO 1 [Source:UniProtKB/Swiss-Prot;Acc:O22161] |
| AT2G44910 | -1,43 | 2,68E-10 | ATHB-4 | Uncharacterized protein At2g44910 (Fragment) [Source:UniProtKB/TrEMBL;Acc:C0SV86] |
| AT2G44940 | -0,68 | 1,80E-05 | ERF034 | Ethylene-responsive transcription factor ERF034 [Source:UniProtKB/Swiss-Prot;Acc:Q8LBQ7] |
| AT2G44980 | 0,76 | 4,71E-26 | CHR10 | Probable helicase CHR10 [Source:UniProtKB/Swiss-Prot;Acc:F4IV45] |
| AT2G45070 | -1,36 | 1,08E-268 | SEC61 BETA | Protein transport protein Sec61 subunit beta [Source:UniProtKB/Swiss-Prot;Acc:P38389] |
| AT2G45130 | -2,59 | 3,00E-02 | SPX3 | SPX domain-containing protein 3 [Source:UniProtKB/Swiss-Prot;Acc:Q5PP62] |
| AT2G45140 | -0,75 | 5,33E-42 | PVA12 | Vesicle-associated protein 1-2 [Source:UniProtKB/Swiss-Prot;Acc:Q9SHC8] |
| AT2G45170 | 3,08 | 6,46E-78 | ATG8E | Autophagy-related protein 8e [Source:UniProtKB/Swiss-Prot;Acc:Q8S926] |
| AT2G45180 | 2,18 | 2,00E-265 | - | At2g45180/T14P1.1 [Source:UniProtKB/TrEMBL;Acc:Q42044] |
| AT2G45210 | 2,25 | 4,38E-43 | SAUR36 | Auxin-responsive protein SAUR36 [Source:UniProtKB/Swiss-Prot;Acc:O22150] |
| AT2G45300 | -1,26 | 6,34E-180 | - | 3-phosphoshikimate 1-carboxyvinyltransferase, chloroplastic [Source:UniProtKB/Swiss-Prot;Acc:P05466] |
| AT2G45330 | 0,89 | 1,13E-31 | emb1067 | RNA 2'-phosphotransferase, Tpt1 / KptA family [Source:UniProtKB/TrEMBL;Acc:F4IW51] |
| AT2G45380 | 0,60 | 6,45E-18 | - | FUNCTIONS IN: molecular_function unknown; INVOLVED IN: biological_process unknown; LOCATED IN: cellular_component unknown; EXPRESSED IN: inflorescence meristem, flower, cultured cell; EXPRESSED DURING: petal differentiation and expansion stage; BEST /.../dopsis thaliana protein match is: Calcium-binding EF-hand family protein (TAIR:AT4G34070.1); Ha. [Source:TAIR;Acc:AT2G45380] |
| AT2G45460 | 0,78 | 7,06E-21 | - | SMAD/FHA domain-containing protein [Source:UniProtKB/TrEMBL;Acc:F4IG63] |
| AT2G45470 | -1,03 | 4,28E-100 | FLA8 | Fasciclin-like arabinogalactan protein 8 [Source:UniProtKB/Swiss-Prot;Acc:O22126] |
| AT2G45480 | -1,05 | 8,20E-18 | GRF9 | Growth-regulating factor 9 [Source:UniProtKB/Swiss-Prot;Acc:Q8S9M3] |
| AT2G45540 | -0,85 | 1,47E-51 | BCHC2 | BEACH domain-containing protein C2 [Source:UniProtKB/Swiss-Prot;Acc:F4IG73] |
| AT2G45600 | -3,44 | 1,72E-02 | CXE8 | Probable carboxylesterase 8 [Source:UniProtKB/Swiss-Prot;Acc:O64640] |
| AT2G45660 | -2,56 | 6,49E-33 | SOC1 | SOC1 [Source:UniProtKB/TrEMBL;Acc:A0A178VZL4] |
| AT2G45670 | -0,98 | 2,77E-97 | LPEAT2 | Lysophospholipid acyltransferase LPEAT2 [Source:UniProtKB/Swiss-Prot;Acc:Q8S8S2] |
| AT2G45740 | 1,80 | 8,03E-08 | PEX11D | Peroxisomal membrane protein 11D [Source:UniProtKB/Swiss-Prot;Acc:O80845] |
| AT2G45750 | -5,05 | 2,76E-20 | - | Probable methyltransferase PMT16 [Source:UniProtKB/Swiss-Prot;Acc:O80844] |
| AT2G45790 | -1,01 | 1,84E-130 | PMM | Phosphomannomutase [Source:UniProtKB/Swiss-Prot;Acc:O80840] |
| AT2G45810 | -0,95 | 1,11E-100 | RH6 | DEAD-box ATP-dependent RNA helicase 6 [Source:UniProtKB/Swiss-Prot;Acc:Q94BV4] |
| AT2G45850 | 1,89 | 3,15E-07 | AHL9 | AT-hook motif nuclear-localized protein 9 [Source:UniProtKB/Swiss-Prot;Acc:O80834] |
| AT2G45890 | -4,15 | 1,41E-37 | ROPGEF4 | Rop guanine nucleotide exchange factor 4 [Source:UniProtKB/Swiss-Prot;Acc:Q0WNP7] |
| AT2G45900 | -2,40 | 8,43E-12 | - | TRM13 [Source:UniProtKB/TrEMBL;Acc:A0A384LCE0] |
| AT2G45920 | 0,81 | 3,73E-30 | PUB37 | U-box domain-containing protein 37 [Source:UniProtKB/Swiss-Prot;Acc:Q683D5] |
| AT2G45930 | 2,53 | 3,60E-09 | - | Expressed protein [Source:UniProtKB/TrEMBL;Acc:O80826] |
| AT2G45940 | 4,46 | 1,01E-08 | - | Protein of unknown function (DUF295) [Source:TAIR;Acc:AT2G45940] |
| AT2G45970 | 1,05 | 1,24E-05 | CYP86A8 | Cytochrome P450 86A8 [Source:UniProtKB/Swiss-Prot;Acc:O80823] |
| AT2G46000 | -1,17 | 2,38E-72 | - | At2g46000 [Source:UniProtKB/TrEMBL;Acc:O82812] |
| AT2G46050 | 0,69 | 2,85E-02 | PCMP-E39 | Pentatricopeptide repeat-containing protein At2g46050, mitochondrial [Source:UniProtKB/Swiss-Prot;Acc:O82363] |
| AT2G46070 | 1,07 | 2,72E-07 | ATMPK12 | MPK12 [Source:UniProtKB/TrEMBL;Acc:A0A178VX75] |
| AT2G46080 | 0,64 | 3,01E-14 | - | BPS2 [Source:UniProtKB/TrEMBL;Acc:A0A178VNI3] |
| AT2G46140 | -1,25 | 5,06E-73 | - | Desiccation-related protein At2g46140 [Source:UniProtKB/Swiss-Prot;Acc:O82355] |
| AT2G46150 | -0,77 | 3,49E-08 | - | Late embryogenesis abundant (LEA) hydroxyproline-rich glycoprotein family [Source:UniProtKB/TrEMBL;Acc:O82354] |
| AT2G46170 | -0,97 | 1,11E-70 | RTNLB5 | Reticulon-like protein B5 [Source:UniProtKB/Swiss-Prot;Acc:O82352] |
| AT2G46240 | 0,95 | 5,55E-54 | BAG6 | BAG family molecular chaperone regulator 6 [Source:UniProtKB/Swiss-Prot;Acc:O82345] |
| AT2G46250 | 1,75 | 9,22E-03 | - | At2g46250/T3F17.10 [Source:UniProtKB/TrEMBL;Acc:O82344] |
| AT2G46310 | 2,64 | 5,19E-42 | CRF5 | Ethylene-responsive transcription factor CRF5 [Source:UniProtKB/Swiss-Prot;Acc:O82339] |
| AT2G46330 | -1,51 | 5,04E-164 | AGP16 | ATAGP16 [Source:UniProtKB/TrEMBL;Acc:A0A178VYP5] |
| AT2G46410 | -0,98 | 7,89E-28 | CPC | Transcription factor CPC [Source:UniProtKB/Swiss-Prot;Acc:O22059] |
| AT2G46450 | -0,70 | 6,85E-31 | CNGC12 | Probable cyclic nucleotide-gated ion channel 12 [Source:UniProtKB/Swiss-Prot;Acc:Q8GWD2] |
| AT2G46480 | -1,49 | 8,10E-04 | GAUT2 | Hexosyltransferase (Fragment) [Source:UniProtKB/TrEMBL;Acc:A0A068FL09] |
| AT2G46505 | -0,58 | 1,12E-47 | SDH4 | SDH4 [Source:UniProtKB/TrEMBL;Acc:A0A178VQB2] |
| AT2G46535 | 1,86 | 9,25E-18 | - | Uncharacterized protein At2g46535 [Source:UniProtKB/TrEMBL;Acc:Q84JE7] |
| AT2G46550 | 0,64 | 1,05E-09 | - | Expressed protein [Source:UniProtKB/TrEMBL;Acc:Q9ZPY4] |
| AT2G46560 | -0,59 | 5,98E-36 | - | Transducin family protein / WD-40 repeat family protein [Source:UniProtKB/TrEMBL;Acc:F4IJ68] |
| AT2G46600 | 0,67 | 3,50E-11 | KIC | Calcium-binding protein KIC [Source:UniProtKB/Swiss-Prot;Acc:Q9ZPX9] |
| AT2G46610 | 1,72 | 6,51E-81 | RS31A | Serine/arginine-rich splicing factor RS31A [Source:UniProtKB/Swiss-Prot;Acc:Q9ZPX8] |
| AT2G46650 | -2,47 | 9,43E-09 | CYTB5-C | Cytochrome B5 isoform C [Source:UniProtKB/Swiss-Prot;Acc:Q9ZNV4] |
| AT2G46680 | 1,16 | 7,86E-06 | ATHB-7 | Homeobox-leucine zipper protein ATHB-7 [Source:UniProtKB/Swiss-Prot;Acc:P46897] |
| AT2G46735 | 1,07 | 1,17E-05 | - | At2g46730/F19D11.1 [Source:UniProtKB/TrEMBL;Acc:Q8VYU9] |
| AT2G46750 | 1,45 | 5,41E-11 | GULLO2 | L-gulonolactone oxidase 2 [Source:UniProtKB/Swiss-Prot;Acc:Q6NQ66] |
| AT2G46800 | 0,61 | 1,15E-68 | MTP1 | Metal tolerance protein 1 [Source:UniProtKB/Swiss-Prot;Acc:Q9ZT63] |
| AT2G46820 | -0,80 | 1,15E-07 | CURT1B | Protein CURVATURE THYLAKOID 1B, chloroplastic [Source:UniProtKB/Swiss-Prot;Acc:Q8LCA1] |
| AT2G46830 | -2,09 | 5,43E-64 | CCA1 | Protein CCA1 [Source:UniProtKB/Swiss-Prot;Acc:P92973] |
| AT2G46840 | 1,52 | 3,34E-05 | DUF4 | DUF4 [Source:UniProtKB/TrEMBL;Acc:A0A178VVQ8] |
| AT2G46860 | -1,66 | 8,63E-06 | PPA3 | PPa3 [Source:UniProtKB/TrEMBL;Acc:A0A178VZ15] |
| AT2G46890 | -0,72 | 1,12E-23 | - | 3-oxo-5-alpha-steroid 4-dehydrogenase (DUF1295) [Source:UniProtKB/TrEMBL;Acc:O81042] |
| AT2G46900 | 0,82 | 1,93E-72 | - | Expressed protein [Source:UniProtKB/TrEMBL;Acc:O80734] |
| AT2G46940 | 2,08 | 9,76E-04 | - | unknown protein; BEST Arabidopsis thaliana protein match is: unknown protein (TAIR:AT3G62070.1); Ha. [Source:TAIR;Acc:AT2G46940] |
| AT2G46950 | 1,95 | 5,39E-201 | CYP709B2 | Cytochrome P450 709B2 [Source:UniProtKB/Swiss-Prot;Acc:F4IK45] |
| AT2G46970 | 1,95 | 2,85E-30 | PIL1 | Transcription factor PIL1 [Source:UniProtKB/Swiss-Prot;Acc:Q8L5W8] |
| AT2G46980 | -0,88 | 9,72E-33 | ASY3 | Meiosis-specific protein ASY3 [Source:UniProtKB/Swiss-Prot;Acc:Q0WR66] |
| AT2G46990 | 0,71 | 3,81E-03 | IAA20 | Auxin-responsive protein [Source:UniProtKB/TrEMBL;Acc:A0A178W181] |
| AT2G47000 | 1,32 | 3,53E-235 | ABCB4 | ABC transporter B family member 4 [Source:UniProtKB/Swiss-Prot;Acc:O80725] |
| AT2G47010 | 3,73 | 2,73E-299 | - | At2g47010/F14M4.16 [Source:UniProtKB/TrEMBL;Acc:Q944A3] |
| AT2G47130 | 0,92 | 2,27E-36 | SDR3A | Short-chain dehydrogenase reductase 3a [Source:UniProtKB/Swiss-Prot;Acc:O80713] |
| AT2G47150 | -1,02 | 4,46E-02 | - | NAD(P)-binding Rossmann-fold superfamily protein [Source:UniProtKB/TrEMBL;Acc:O80711] |
| AT2G47160 | 0,65 | 7,30E-35 | BOR1 | HCO3-transporter family [Source:UniProtKB/TrEMBL;Acc:A8MS82] |
| AT2G47170 | -0,90 | 4,42E-157 | ARF1 | ADP-ribosylation factor 1 [Source:UniProtKB/Swiss-Prot;Acc:P36397] |
| AT2G47180 | 6,62 | 9,33E-83 | GOLS1 | Galactinol synthase 1 [Source:UniProtKB/Swiss-Prot;Acc:O22893] |
| AT2G47230 | 1,01 | 7,22E-32 | DUF6 | DOMAIN OF UNKNOWN FUNCTION 724 6 [Source:TAIR;Acc:AT2G47230] |
| AT2G47260 | 1,24 | 3,32E-09 | WRKY23 | WRKY transcription factor 23 [Source:UniProtKB/Swiss-Prot;Acc:O22900] |
| AT2G47270 | -1,63 | 1,39E-18 | UPB1 | UPB1 [Source:UniProtKB/TrEMBL;Acc:A0A178VV95] |
| AT2G47320 | -0,92 | 1,83E-67 | CYP21-3 | Peptidyl-prolyl cis-trans isomerase CYP21-3, mitochondrial [Source:UniProtKB/Swiss-Prot;Acc:Q94A16] |
| AT2G47360 | -1,48 | 1,33E-15 | - | Transmembrane protein [Source:UniProtKB/TrEMBL;Acc:O22910] |
| AT2G47370 | -0,66 | 2,22E-02 | - | At2g47370 [Source:UniProtKB/TrEMBL;Acc:O22911] |
| AT2G47400 | -1,42 | 3,50E-08 | CP12-1 | Calvin cycle protein CP12-1, chloroplastic [Source:UniProtKB/Swiss-Prot;Acc:O22914] |
| AT2G47450 | -2,27 | 1,60E-18 | CAO | Signal recognition particle 43 kDa protein, chloroplastic [Source:UniProtKB/Swiss-Prot;Acc:O22265] |
| AT2G47460 | 1,44 | 1,15E-123 | MYB12 | Transcription factor MYB12 [Source:UniProtKB/Swiss-Prot;Acc:O22264] |
| AT2G47470 | -0,61 | 9,83E-68 | PDIL2-1 | UNE5 [Source:UniProtKB/TrEMBL;Acc:A0A178W1F8] |
| AT2G47480 | -1,55 | 6,33E-04 | - | DUF3511 domain protein, putative (DUF3511) [Source:UniProtKB/TrEMBL;Acc:O22262] |
| AT2G47485 | -0,69 | 1,52E-19 | - | At2g47485 [Source:UniProtKB/TrEMBL;Acc:Q6DBG0] |
| AT2G47490 | -1,23 | 1,11E-109 | NDT1 | Nicotinamide adenine dinucleotide transporter 1, chloroplastic [Source:UniProtKB/Swiss-Prot;Acc:O22261] |
| AT2G47520 | 7,44 | 0,00E+00 | ERF071 | Ethylene-responsive transcription factor ERF071 [Source:UniProtKB/Swiss-Prot;Acc:O22259] |
| AT2G47530 | -5,07 | 3,37E-04 | - | Pollen Ole e 1 allergen and extensin family protein [Source:UniProtKB/TrEMBL;Acc:O22258] |
| AT2G47540 | -6,11 | 2,94E-17 | - | Pollen Ole e 1 allergen and extensin family protein [Source:UniProtKB/TrEMBL;Acc:O22257] |
| AT2G47550 | -1,42 | 1,83E-44 | PME20 | Probable pectinesterase/pectinesterase inhibitor 20 [Source:UniProtKB/Swiss-Prot;Acc:O22256] |
| AT2G47600 | -0,61 | 1,17E-22 | MHX | Magnesium/proton exchanger [Source:UniProtKB/Swiss-Prot;Acc:O22252] |
| AT2G47630 | 1,52 | 8,49E-60 | - | Alpha/beta-Hydrolases superfamily protein [Source:UniProtKB/TrEMBL;Acc:O22248] |
| AT2G47650 | -0,97 | 6,24E-97 | UXS4 | AT2G47650 protein [Source:UniProtKB/TrEMBL;Acc:C0Z2I3] |
| AT2G47680 | 0,65 | 1,30E-18 | - | DExH-box ATP-dependent RNA helicase DExH8 [Source:UniProtKB/Swiss-Prot;Acc:O22243] |
| AT2G47710 | 1,47 | 2,79E-114 | - | Adenine nucleotide alpha hydrolases-like superfamily protein [Source:UniProtKB/TrEMBL;Acc:O82240] |
| AT2G47750 | -1,37 | 3,17E-02 | GH3.9 | Putative indole-3-acetic acid-amido synthetase GH3.9 [Source:UniProtKB/Swiss-Prot;Acc:O82243] |
| AT2G47790 | 0,62 | 3,00E-42 | GTS1 | WD repeat-containing protein GTS1 [Source:UniProtKB/Swiss-Prot;Acc:Q944S2] |
| AT2G47890 | 2,98 | 0,00E+00 | COL13 | Zinc finger protein CONSTANS-LIKE 13 [Source:UniProtKB/Swiss-Prot;Acc:O82256] |
| AT2G47895 | 1,45 | 2,22E-06 | - | other RNA [Source:TAIR;Acc:AT2G47895] |
| AT2G48010 | 0,98 | 8,47E-64 | RKF3 | Probable LRR receptor-like serine/threonine-protein kinase RKF3 [Source:UniProtKB/Swiss-Prot;Acc:P93050] |
| AT2G48030 | 0,72 | 3,39E-10 | - | At2g48030 [Source:UniProtKB/TrEMBL;Acc:Q9ZU86] |
| AT2G48070 | 0,59 | 2,46E-06 | RPH1 | Resistance to phytophthora 1 [Source:UniProtKB/TrEMBL;Acc:F4IN59] |
| AT2G48080 | -2,74 | 4,90E-53 | - | Oxidoreductase, 2OG-Fe(II) oxygenase family protein [Source:UniProtKB/TrEMBL;Acc:F4IN63] |
| AT2G48121 | 2,12 | 3,70E-10 | - | LOCATED IN: mitochondrion; BEST Arabidopsis thaliana protein match is: Ribonuclease III family protein (TAIR:AT4G37510.1). [Source:TAIR;Acc:AT2G48121] |
| AT3G01015 | -1,08 | 2,72E-02 | - | TPX2 (Targeting protein for Xklp2) protein family [Source:UniProtKB/TrEMBL;Acc:Q5XVC4] |
| AT3G01040 | -0,68 | 3,20E-30 | GAUT13 | GAUT13 [Source:UniProtKB/TrEMBL;Acc:A0A384LCY9] |
| AT3G01190 | -1,14 | 1,15E-12 | PER27 | Peroxidase [Source:UniProtKB/TrEMBL;Acc:A0A178V802] |
| AT3G01210 | 3,43 | 7,36E-36 | - | RNA-binding (RRM/RBD/RNP motifs) family protein [Source:UniProtKB/TrEMBL;Acc:Q9MAD0] |
| AT3G01220 | -0,62 | 5,57E-21 | ATHB-20 | Homeobox-leucine zipper protein ATHB-20 [Source:UniProtKB/Swiss-Prot;Acc:Q8LAT0] |
| AT3G01235 | 0,95 | 4,91E-02 | - | - |
| AT3G01290 | -1,00 | 1,40E-20 | HIR3 | HIR2 [Source:UniProtKB/TrEMBL;Acc:A0A178V7M0] |
| AT3G01310 | 1,24 | 1,86E-116 | - | Phosphoglycerate mutase-like family protein [Source:UniProtKB/TrEMBL;Acc:F4J8C7] |
| AT3G01311 | 1,27 | 4,88E-03 | - | Actin cross-linking protein, putative (DUF569) [Source:UniProtKB/TrEMBL;Acc:F4J8C8] |
| AT3G01345 | -1,02 | 1,07E-19 | - | Expressed protein [Source:UniProtKB/TrEMBL;Acc:Q3EBD7] |
| AT3G01360 | -0,77 | 1,35E-36 | - | At3g01360 [Source:UniProtKB/TrEMBL;Acc:Q9SRI3] |
| AT3G01380 | -0,58 | 5,72E-26 | - | Sulfatase and phosphatidylinositolglycan class N domain-containing protein [Source:UniProtKB/TrEMBL;Acc:A0A1I9LQ21] |
| AT3G01390 | -1,02 | 2,09E-151 | VHA-G1 | V-type proton ATPase subunit G [Source:UniProtKB/TrEMBL;Acc:A0A178VL96] |
| AT3G01410 | -0,93 | 4,01E-28 | - | Polynucleotidyl transferase, ribonuclease H-like superfamily protein [Source:UniProtKB/TrEMBL;Acc:F4JEB4] |
| AT3G01420 | 5,40 | 1,98E-54 | DOX1 | Alpha-dioxygenase 1 [Source:UniProtKB/Swiss-Prot;Acc:Q9SGH6] |
| AT3G01430 | 1,01 | 1,53E-17 | - | NHL domain protein [Source:UniProtKB/TrEMBL;Acc:Q9SGH5] |
| AT3G01480 | -1,06 | 1,81E-15 | CYP38 | Peptidyl-prolyl cis-trans isomerase CYP38, chloroplastic [Source:UniProtKB/Swiss-Prot;Acc:Q9SSA5] |
| AT3G01520 | 1,05 | 2,43E-38 | - | Universal stress protein A-like protein [Source:UniProtKB/Swiss-Prot;Acc:Q8LGG8] |
| AT3G01600 | 4,10 | 5,65E-242 | anac044 | NAC domain containing protein 44 [Source:UniProtKB/TrEMBL;Acc:F4J4R5] |
| AT3G01630 | 2,30 | 4,04E-03 | - | F4P13.17 protein [Source:UniProtKB/TrEMBL;Acc:Q9SS92] |
| AT3G01640 | -0,80 | 4,09E-29 | ATGLCAK | Glucuronokinase G [Source:UniProtKB/TrEMBL;Acc:A0A1I9LRK4] |
| AT3G01670 | 1,32 | 2,77E-04 | - | Sieve element occlusion protein [Source:UniProtKB/TrEMBL;Acc:A0A1I9LMF2] |
| AT3G01690 | 0,67 | 2,21E-30 | - | Alpha/beta-Hydrolases superfamily protein [Source:UniProtKB/TrEMBL;Acc:Q9S7U7] |
| AT3G01720 | -0,93 | 2,73E-64 | SERGT1 | Peptidyl serine alpha-galactosyltransferase [Source:UniProtKB/Swiss-Prot;Acc:Q8VYF9] |
| AT3G01730 | -2,50 | 1,84E-03 | - | F28J7.6 protein [Source:UniProtKB/TrEMBL;Acc:Q9S7S5] |
| AT3G01750 | -0,61 | 2,89E-04 | - | Ankyrin repeat family protein [Source:UniProtKB/TrEMBL;Acc:Q9S7I4] |
| AT3G01770 | 1,77 | 9,37E-252 | GTE11 | Transcription factor GTE11 [Source:UniProtKB/Swiss-Prot;Acc:Q93ZB7] |
| AT3G01780 | -0,89 | 2,40E-76 | TPLATE | Protein TPLATE [Source:UniProtKB/Swiss-Prot;Acc:F4J8D3] |
| AT3G01795 | 1,83 | 1,67E-05 | - | - |
| AT3G01850 | 1,07 | 3,15E-39 | - | Ribulose-phosphate 3-epimerase [Source:UniProtKB/TrEMBL;Acc:Q94K13] |
| AT3G01890 | 0,69 | 1,45E-18 | - | At3g01890 [Source:UniProtKB/TrEMBL;Acc:Q147F4] |
| AT3G01900 | 0,63 | 1,81E-10 | CYP94B2 | Cytochrome P450, family 94, subfamily B, polypeptide 2 [Source:UniProtKB/TrEMBL;Acc:Q9S833] |
| AT3G01920 | 1,13 | 2,46E-24 | - | DHBP synthase RibB-like alpha/beta domain-containing protein [Source:UniProtKB/TrEMBL;Acc:A0A1I9LM38] |
| AT3G01970 | 3,23 | 1,81E-36 | WRKY45 | Probable WRKY transcription factor 45 [Source:UniProtKB/Swiss-Prot;Acc:Q9S763] |
| AT3G01990 | 1,67 | 3,02E-12 | ACR6 | ACT domain repeat 6 [Source:UniProtKB/TrEMBL;Acc:A0A1I9LSD7] |
| AT3G02020 | 2,29 | 5,35E-05 | AK3 | Aspartokinase 3, chloroplastic [Source:UniProtKB/Swiss-Prot;Acc:Q9S702] |
| AT3G02110 | 0,80 | 8,24E-19 | SCPL25 | Serine carboxypeptidase-like 25 [Source:UniProtKB/Swiss-Prot;Acc:Q8L9Y0] |
| AT3G02120 | -1,27 | 6,98E-68 | - | Hydroxyproline-rich glycoprotein family protein [Source:UniProtKB/TrEMBL;Acc:Q8L9N1] |
| AT3G02130 | 0,71 | 2,23E-29 | RPK2 | LRR receptor-like serine/threonine-protein kinase RPK2 [Source:UniProtKB/Swiss-Prot;Acc:Q9S7I6] |
| AT3G02150 | 2,96 | 1,54E-07 | TCP13 | Transcription factor TCP13 [Source:UniProtKB/Swiss-Prot;Acc:Q9S7W5] |
| AT3G02190 | -0,61 | 5,64E-29 | RPL39B | 60S ribosomal protein L39-2 [Source:UniProtKB/Swiss-Prot;Acc:Q8L8W6] |
| AT3G02210 | 1,07 | 1,57E-21 | COBL1 | COBRA-like protein [Source:UniProtKB/TrEMBL;Acc:A0A178VHK7] |
| AT3G02230 | -1,76 | 3,50E-293 | RGP1 | RGP1 [Source:UniProtKB/TrEMBL;Acc:A0A178VK37] |
| AT3G02240 | 1,53 | 4,49E-18 | RGF7 | Root meristem growth factor 7 [Source:UniProtKB/Swiss-Prot;Acc:Q6NNL3] |
| AT3G02242 | 1,78 | 8,55E-03 | GLV8 | Protein GOLVEN 8 [Source:UniProtKB/Swiss-Prot;Acc:B3H5Q2] |
| AT3G02250 | -0,65 | 7,33E-29 | OFUT21 | O-fucosyltransferase 21 [Source:UniProtKB/Swiss-Prot;Acc:Q93ZR8] |
| AT3G02350 | -0,84 | 3,61E-98 | GAUT9 | Hexosyltransferase [Source:UniProtKB/TrEMBL;Acc:A0A178VEE3] |
| AT3G02370 | 0,88 | 1,66E-10 | - | tRNA-splicing endonuclease subunit [Source:UniProtKB/TrEMBL;Acc:F4J8D6] |
| AT3G02380 | 0,70 | 1,19E-07 | COL2 | Zinc finger protein CONSTANS-LIKE 2 [Source:UniProtKB/Swiss-Prot;Acc:Q96502] |
| AT3G02410 | -1,02 | 3,11E-03 | ICMEL2 | Carboxylic ester hydrolase [Source:UniProtKB/TrEMBL;Acc:A0A178V7M1] |
| AT3G02430 | -1,76 | 3,27E-10 | DMP5 | Protein DMP5 [Source:UniProtKB/Swiss-Prot;Acc:Q9M897] |
| AT3G02435 | -1,97 | 8,20E-03 | - | - |
| AT3G02550 | 2,26 | 8,00E-57 | LBD41 | LOB domain-containing protein 41 [Source:UniProtKB/Swiss-Prot;Acc:Q9M886] |
| AT3G02555 | 1,12 | 1,42E-30 | - | At3g02555 [Source:UniProtKB/TrEMBL;Acc:Q8LF93] |
| AT3G02570 | -0,83 | 1,24E-57 | PMI1 | PMI1 [Source:UniProtKB/TrEMBL;Acc:A0A178VN24] |
| AT3G02590 | 1,01 | 3,32E-03 | HDF7 | Putative Delta(7)-sterol-C5(6)-desaturase 2 [Source:UniProtKB/Swiss-Prot;Acc:Q9M883] |
| AT3G02600 | -0,80 | 3,66E-58 | LPP3 | Putative lipid phosphate phosphatase 3, chloroplastic [Source:UniProtKB/Swiss-Prot;Acc:Q8LFD1] |
| AT3G02610 | 0,93 | 1,66E-04 | - | Plant stearoyl-acyl-carrier desaturase family protein [Source:UniProtKB/TrEMBL;Acc:A0A178V7P2] |
| AT3G02620 | 1,21 | 9,81E-27 | - | Plant stearoyl-acyl-carrier-protein desaturase family protein [Source:UniProtKB/TrEMBL;Acc:A0A178VGR6] |
| AT3G02630 | -0,58 | 4,06E-31 | S-ACP-DES5 | Stearoyl-[acyl-carrier-protein] 9-desaturase 5, chloroplastic [Source:UniProtKB/Swiss-Prot;Acc:Q9M879] |
| AT3G02640 | -0,88 | 1,47E-36 | - | F16B3.27 protein [Source:UniProtKB/TrEMBL;Acc:Q9M878] |
| AT3G02677 | -2,51 | 4,92E-06 | - | - |
| AT3G02690 | 0,68 | 1,56E-12 | - | WAT1-related protein At3g02690, chloroplastic [Source:UniProtKB/Swiss-Prot;Acc:Q93V85] |
| AT3G02695 | 2,13 | 1,02E-03 | - | - |
| AT3G02700 | 0,88 | 9,02E-21 | - | At3g02700 [Source:UniProtKB/TrEMBL;Acc:Q9M872] |
| AT3G02750 | -0,70 | 3,84E-42 | - | Protein phosphatase 2C family protein [Source:TAIR;Acc:AT3G02750] |
| AT3G02770 | -1,00 | 6,21E-36 | - | Putative 4-hydroxy-4-methyl-2-oxoglutarate aldolase 1 [Source:UniProtKB/Swiss-Prot;Acc:Q9M8R9] |
| AT3G02830 | 1,18 | 3,28E-34 | ZFN1 | Zinc finger CCCH domain-containing protein 33 [Source:UniProtKB/Swiss-Prot;Acc:Q8GXX7] |
| AT3G02870 | -0,63 | 1,03E-25 | VTC4 | Inositol-phosphate phosphatase [Source:UniProtKB/Swiss-Prot;Acc:Q9M8S8] |
| AT3G02885 | 4,08 | 2,61E-02 | GASA5 | Gibberellin-regulated protein 5 [Source:UniProtKB/Swiss-Prot;Acc:Q84J95] |
| AT3G02990 | -0,64 | 1,14E-09 | HSFA1E | Heat stress transcription factor A-1e [Source:UniProtKB/Swiss-Prot;Acc:Q9SCW5] |
| AT3G03020 | 1,07 | 8,01E-08 | - | AT3G03020 protein [Source:UniProtKB/TrEMBL;Acc:Q8GUN0] |
| AT3G03025 | 1,33 | 4,55E-02 | - | - |
| AT3G03050 | -0,98 | 4,39E-76 | CSLD3 | Glycosyltransferase (Fragment) [Source:UniProtKB/TrEMBL;Acc:W8Q6V3] |
| AT3G03060 | 1,01 | 1,26E-147 | - | P-loop containing nucleoside triphosphate hydrolases superfamily protein [Source:UniProtKB/TrEMBL;Acc:Q0WVF7] |
| AT3G03120 | 0,94 | 5,88E-30 | ATARFB1C | ATARFB1C [Source:UniProtKB/TrEMBL;Acc:A0A384KVK5] |
| AT3G03130 | -1,18 | 3,22E-49 | - | LisH domain-like protein [Source:UniProtKB/TrEMBL;Acc:Q9M9N2] |
| AT3G03150 | -0,73 | 9,74E-26 | - | At3g03150 [Source:UniProtKB/TrEMBL;Acc:Q84VW1] |
| AT3G03160 | -0,75 | 2,35E-71 | - | B-cell receptor-associated-like protein [Source:UniProtKB/TrEMBL;Acc:Q9M9N5] |
| AT3G03170 | 0,90 | 4,38E-08 | - | At3g03170 [Source:UniProtKB/TrEMBL;Acc:Q29PZ2] |
| AT3G03210 | -0,60 | 1,03E-10 | - | At3g03210 [Source:UniProtKB/TrEMBL;Acc:Q9M9N9] |
| AT3G03225 | -0,66 | 4,49E-02 | - | - |
| AT3G03250 | -1,39 | 2,16E-169 | UGP | UTP--glucose-1-phosphate uridylyltransferase [Source:UniProtKB/TrEMBL;Acc:A0A1I9LT02] |
| AT3G03265 | -4,69 | 9,20E-10 | - | - |
| AT3G03270 | 2,55 | 2,61E-260 | - | AT3G03270 protein [Source:UniProtKB/TrEMBL;Acc:Q8LFK2] |
| AT3G03272 | 2,89 | 5,55E-05 | - | Prolamin-like protein (DUF1278) [Source:UniProtKB/TrEMBL;Acc:A7RED5] |
| AT3G03290 | -2,09 | 2,41E-08 | - | Adenine nucleotide alpha hydrolases-like superfamily protein [Source:UniProtKB/TrEMBL;Acc:Q9M9P7] |
| AT3G03310 | 0,70 | 4,48E-38 | LCAT3 | Phospholipase A(1) LCAT3 [Source:UniProtKB/Swiss-Prot;Acc:Q93V61] |
| AT3G03330 | 0,84 | 7,75E-38 | - | AT3g03330/T21P5_25 [Source:UniProtKB/TrEMBL;Acc:Q8L7U0] |
| AT3G03370 | 0,95 | 2,63E-05 | - | BEST Arabidopsis thaliana protein match is: DegP protease 7 (TAIR:AT3G03380.1); Ha. [Source:TAIR;Acc:AT3G03370] |
| AT3G03450 | -0,93 | 1,11E-36 | RGL2 | RGL2 [Source:UniProtKB/TrEMBL;Acc:A0A178VH73] |
| AT3G03470 | 0,99 | 5,36E-15 | CYP89A9 | Cytochrome P450 89A9 [Source:UniProtKB/Swiss-Prot;Acc:Q9SRQ1] |
| AT3G03490 | 0,60 | 1,36E-10 | PEX19-1 | Peroxisome biogenesis protein 19-1 [Source:UniProtKB/Swiss-Prot;Acc:Q9SRQ3] |
| AT3G03520 | -2,84 | 3,10E-92 | NPC3 | NPC3 [Source:UniProtKB/TrEMBL;Acc:A0A178VBD6] |
| AT3G03530 | -3,23 | 7,20E-05 | NPC4 | Non-specific phospholipase C4 [Source:UniProtKB/Swiss-Prot;Acc:Q9SRQ7] |
| AT3G03560 | -0,66 | 3,02E-40 | - | unknown protein; LOCATED IN: plasma membrane; EXPRESSED IN: 22 plant structures; EXPRESSED DURING: 13 growth stages; BEST Arabidopsis thaliana protein match is: unknown protein (TAIR:AT5G23490.1); Ha. [Source:TAIR;Acc:AT3G03560] |
| AT3G03640 | -0,81 | 1,15E-53 | BGLU25 | Probable inactive beta-glucosidase 25 [Source:UniProtKB/Swiss-Prot;Acc:O82772] |
| AT3G03660 | 1,35 | 4,50E-08 | WOX11 | WUSCHEL related homeobox 11 [Source:UniProtKB/TrEMBL;Acc:A0A1I9LND8] |
| AT3G03680 | -1,40 | 1,07E-97 | - | C2 calcium/lipid-binding plant phosphoribosyltransferase family protein [Source:UniProtKB/TrEMBL;Acc:Q9SS68] |
| AT3G03690 | -1,86 | 2,30E-116 | UNE7 | Core-2/I-branching beta-1,6-N-acetylglucosaminyltransferase family protein [Source:UniProtKB/TrEMBL;Acc:Q9SS69] |
| AT3G03702 | 1,00 | 4,99E-02 | - | other RNA [Source:TAIR;Acc:AT3G03702] |
| AT3G03776 | 3,73 | 3,97E-02 | - | Hydroxyproline-rich glycoprotein family protein [Source:UniProtKB/TrEMBL;Acc:A0A1I9LP11] |
| AT3G03780 | -1,93 | 5,04E-96 | MS2 | 5-methyltetrahydropteroyltriglutamate--homocysteine methyltransferase 2 [Source:UniProtKB/Swiss-Prot;Acc:Q9SRV5] |
| AT3G03820 | -3,64 | 9,03E-03 | - | Putative auxin-induced protein [Source:UniProtKB/TrEMBL;Acc:Q9SRV9] |
| AT3G03880 | 0,61 | 3,73E-14 | - | Sterol O-acyltransferase, putative (DUF1639) [Source:UniProtKB/TrEMBL;Acc:Q84JV8] |
| AT3G03900 | 0,59 | 3,30E-13 | APK3 | Adenylyl-sulfate kinase 3 [Source:UniProtKB/Swiss-Prot;Acc:Q9SRW7] |
| AT3G03910 | -4,36 | 2,06E-03 | GSH3 | Glutamate dehydrogenase [Source:UniProtKB/TrEMBL;Acc:A0A178VBI2] |
| AT3G03930 | 4,93 | 2,54E-17 | - | F20H23.2 protein [Source:UniProtKB/TrEMBL;Acc:Q9S828] |
| AT3G03950 | 0,95 | 7,53E-110 | ECT1 | YTH domain-containing protein ECT1 [Source:UniProtKB/Swiss-Prot;Acc:Q3MK94] |
| AT3G03960 | -0,91 | 7,32E-119 | CCT8 | T-complex protein 1 subunit theta [Source:UniProtKB/Swiss-Prot;Acc:Q94K05] |
| AT3G04000 | 5,12 | 3,22E-29 | ChlADR2 | NADPH-dependent aldehyde reductase 2, chloroplastic [Source:UniProtKB/Swiss-Prot;Acc:Q9SQR2] |
| AT3G04010 | 1,07 | 1,94E-08 | - | At3g04010 [Source:UniProtKB/TrEMBL;Acc:Q9SQR1] |
| AT3G04030 | 2,37 | 9,46E-06 | MYR2 | Myb-related protein 2 [Source:UniProtKB/Swiss-Prot;Acc:Q9SQQ9] |
| AT3G04040 | -0,74 | 7,20E-03 | - | AT3G04040 protein [Source:UniProtKB/TrEMBL;Acc:Q6NNK6] |
| AT3G04050 | 1,27 | 3,62E-03 | - | Pyruvate kinase [Source:UniProtKB/TrEMBL;Acc:Q9SQQ7] |
| AT3G04140 | -0,58 | 3,97E-11 | - | Ankyrin repeat family protein [Source:UniProtKB/TrEMBL;Acc:Q9M8X0] |
| AT3G04155 | 1,48 | 2,32E-08 | - | - |
| AT3G04160 | 0,65 | 6,72E-19 | - | unknown protein; Ha. [Source:TAIR;Acc:AT3G04160] |
| AT3G04165 | 1,72 | 8,42E-08 | - | - |
| AT3G04175 | 1,37 | 6,48E-18 | - | - |
| AT3G04220 | -4,31 | 2,30E-02 | - | Disease resistance protein (TIR-NBS-LRR class) family [Source:UniProtKB/TrEMBL;Acc:Q9M8X8] |
| AT3G04330 | -1,70 | 3,82E-49 | - | Kunitz family trypsin and protease inhibitor protein [Source:UniProtKB/TrEMBL;Acc:Q9M8Y9] |
| AT3G04350 | 1,02 | 2,07E-12 | - | AT3g04350/T6K12_3 [Source:UniProtKB/TrEMBL;Acc:Q9M8Z1] |
| AT3G04355 | -1,21 | 2,23E-02 | - | - |
| AT3G04360 | 1,19 | 4,17E-08 | - | Calcium-dependent lipid-binding (CaLB domain) family protein [Source:UniProtKB/TrEMBL;Acc:F4J3N4] |
| AT3G04370 | 2,86 | 1,76E-02 | CRRSP39 | Cysteine-rich repeat secretory protein 39 [Source:UniProtKB/Swiss-Prot;Acc:Q6E263] |
| AT3G04400 | -0,90 | 1,01E-115 | RPL23A | 60S ribosomal protein L23 [Source:UniProtKB/Swiss-Prot;Acc:P49690] |
| AT3G04420 | 0,75 | 1,51E-08 | anac048 | AT3g04420/T27C4_6 [Source:UniProtKB/TrEMBL;Acc:Q8W4R4] |
| AT3G04485 | -0,90 | 2,73E-06 | - | other RNA [Source:TAIR;Acc:AT3G04485] |
| AT3G04530 | -0,80 | 6,95E-03 | PPCK2 | Phosphoenolpyruvate carboxylase kinase 2 [Source:UniProtKB/Swiss-Prot;Acc:Q93VK0] |
| AT3G04550 | 0,58 | 3,42E-03 | RAF1.2 | Rubisco accumulation factor 1.2, chloroplastic [Source:UniProtKB/Swiss-Prot;Acc:Q9SR19] |
| AT3G04630 | 0,69 | 8,32E-09 | WDL1 | WVD2-like 1 [Source:TAIR;Acc:AT3G04630] |
| AT3G04640 | 0,60 | 4,58E-03 | - | At3g04640 [Source:UniProtKB/TrEMBL;Acc:Q9SR10] |
| AT3G04660 | 4,64 | 2,68E-03 | - | F-box/kelch-repeat protein At3g04660 [Source:UniProtKB/Swiss-Prot;Acc:Q9SR08] |
| AT3G04670 | 0,74 | 5,48E-61 | WRKY39 | Probable WRKY transcription factor 39 [Source:UniProtKB/Swiss-Prot;Acc:Q9SR07] |
| AT3G04695 | 3,94 | 6,28E-03 | - | - |
| AT3G04720 | -1,27 | 8,26E-31 | HEL | Hevein-like preproprotein [Source:UniProtKB/Swiss-Prot;Acc:P43082] |
| AT3G04765 | -3,91 | 1,06E-02 | MIR167C | MIR167C; miRNA [Source:TAIR;Acc:AT3G04765] |
| AT3G04780 | -0,88 | 2,15E-80 | - | PITH domain-containing protein At3g04780 [Source:UniProtKB/Swiss-Prot;Acc:Q9SQZ9] |
| AT3G04800 | 1,81 | 2,25E-47 | TIM23-3 | Mitochondrial import inner membrane translocase subunit TIM23-3 [Source:UniProtKB/Swiss-Prot;Acc:Q9S837] |
| AT3G04830 | -0,98 | 1,90E-147 | - | Contains similarity to O-linked GlcNAc transferases [Source:UniProtKB/TrEMBL;Acc:Q9CAU9] |
| AT3G04840 | -1,08 | 1,68E-153 | RPS3AA | 40S ribosomal protein S3a-1 [Source:UniProtKB/Swiss-Prot;Acc:Q9CAV0] |
| AT3G04854 | -0,92 | 2,68E-03 | - | unknown protein; LOCATED IN: endomembrane system; Ha. [Source:TAIR;Acc:AT3G04854] |
| AT3G04910 | -0,64 | 1,96E-28 | WNK1 | Serine/threonine-protein kinase WNK1 [Source:UniProtKB/Swiss-Prot;Acc:Q9CAV6] |
| AT3G04940 | -0,86 | 3,12E-44 | CYSD1 | Bifunctional L-3-cyanoalanine synthase/cysteine synthase D1 [Source:UniProtKB/Swiss-Prot;Acc:Q9S6Z7] |
| AT3G05020 | -1,04 | 8,52E-140 | ACP1 | Acyl carrier protein [Source:UniProtKB/TrEMBL;Acc:Q0WT41] |
| AT3G05140 | 1,30 | 1,72E-03 | RBK2 | RBK2 [Source:UniProtKB/TrEMBL;Acc:A0A384KHI2] |
| AT3G05155 | -6,16 | 3,85E-26 | - | Major facilitator superfamily protein [Source:TAIR;Acc:AT3G05155] |
| AT3G05165 | 0,93 | 1,92E-28 | - | Major facilitator superfamily protein [Source:UniProtKB/TrEMBL;Acc:A0A1I9LTG5] |
| AT3G05180 | 1,21 | 2,08E-02 | - | GDSL esterase/lipase At3g05180 [Source:UniProtKB/Swiss-Prot;Acc:Q9MAA1] |
| AT3G05230 | -1,04 | 1,89E-77 | - | Signal peptidase complex subunit 3 [Source:UniProtKB/TrEMBL;Acc:A0A178V7D8] |
| AT3G05250 | 0,78 | 4,08E-18 | - | At3g05250 [Source:UniProtKB/TrEMBL;Acc:Q8GYA2] |
| AT3G05280 | -0,77 | 7,30E-79 | - | Protein YIPF [Source:UniProtKB/TrEMBL;Acc:Q8GWB3] |
| AT3G05330 | -1,08 | 1,21E-18 | TAN | Probable microtubule-binding protein TANGLED [Source:UniProtKB/Swiss-Prot;Acc:Q84M91] |
| AT3G05335 | -0,66 | 1,73E-02 | - | - |
| AT3G05345 | 0,85 | 2,00E-08 | - | Chaperone DnaJ-domain superfamily protein [Source:UniProtKB/TrEMBL;Acc:F4J794] |
| AT3G05350 | 0,79 | 2,16E-26 | APP2 | Aminopeptidase P2 [Source:UniProtKB/Swiss-Prot;Acc:Q8RY11] |
| AT3G05355 | -0,67 | 1,34E-06 | - | - |
| AT3G05360 | 2,02 | 1,72E-92 | RLP30 | Receptor-like protein 30 [Source:UniProtKB/Swiss-Prot;Acc:Q9MA83] |
| AT3G05400 | 9,06 | 1,32E-17 | SUGTL5 | Sugar transporter ERD6-like 12 [Source:UniProtKB/Swiss-Prot;Acc:Q8VZT3] |
| AT3G05420 | -0,76 | 8,86E-65 | ACBP4 | acyl-CoA binding protein 4 [Source:TAIR;Acc:AT3G05420] |
| AT3G05430 | 0,61 | 1,37E-05 | - | F22F7.12 protein [Source:UniProtKB/TrEMBL;Acc:Q9MA56] |
| AT3G05500 | -0,69 | 1,64E-61 | - | REF/SRPP-like protein At3g05500 [Source:UniProtKB/Swiss-Prot;Acc:Q9MA63] |
| AT3G05560 | -1,00 | 5,05E-175 | RPL22B | 60S ribosomal protein L22-2 [Source:UniProtKB/Swiss-Prot;Acc:Q9M9W1] |
| AT3G05590 | -0,64 | 5,17E-65 | RPL18B | RPL18 [Source:UniProtKB/TrEMBL;Acc:A0A178V8M9] |
| AT3G05640 | -2,09 | 2,48E-06 | - | Probable protein phosphatase 2C 34 [Source:UniProtKB/Swiss-Prot;Acc:Q9M9W9] |
| AT3G05655 | -2,98 | 3,40E-14 | - | - |
| AT3G05660 | 1,84 | 2,10E-02 | AtRLP33 | Receptor-like protein 33 [Source:UniProtKB/Swiss-Prot;Acc:F4J8G2] |
| AT3G05685 | 1,31 | 1,08E-28 | - | Cystatin/monellin superfamily protein [Source:UniProtKB/TrEMBL;Acc:F4J8G8] |
| AT3G05710 | -0,76 | 4,72E-42 | SYP43 | Syntaxin-43 [Source:UniProtKB/Swiss-Prot;Acc:Q9SUJ1] |
| AT3G05720 | -1,04 | 2,71E-02 | IMPA7 | Importin subunit alpha-7 [Source:UniProtKB/Swiss-Prot;Acc:Q9M9X7] |
| AT3G05790 | 1,18 | 4,96E-08 | LON4 | Lon protease homolog 4, chloroplastic/mitochondrial [Source:UniProtKB/Swiss-Prot;Acc:Q9M9L7] |
| AT3G05800 | -1,29 | 5,42E-13 | BHLH150 | Transcription factor bHLH150 [Source:UniProtKB/Swiss-Prot;Acc:Q9M9L6] |
| AT3G05820 | 1,13 | 5,55E-22 | INVH | Probable alkaline/neutral invertase A, chloroplastic [Source:UniProtKB/Swiss-Prot;Acc:Q84JL5] |
| AT3G05858 | -3,52 | 1,72E-04 | - | Putative uncharacterized protein [Source:UniProtKB/TrEMBL;Acc:Q1G3M8] |
| AT3G05880 | 1,18 | 1,93E-31 | RCI2A | RCI2A [Source:UniProtKB/TrEMBL;Acc:A0A178V7D5] |
| AT3G05920 | -0,66 | 2,26E-08 | HIPP43 | Heavy metal-associated isoprenylated plant protein 43 [Source:UniProtKB/Swiss-Prot;Acc:Q9SFF7] |
| AT3G05932 | 1,51 | 1,08E-04 | - | Potential natural antisense gene, locus overlaps with AT3G05930 [Source:TAIR;Acc:AT3G05932] |
| AT3G05936 | -0,98 | 6,55E-03 | - | Putative uncharacterized protein [Source:UniProtKB/TrEMBL;Acc:Q1G3B3] |
| AT3G05937 | 1,80 | 3,55E-10 | - | unknown protein; Ha. [Source:TAIR;Acc:AT3G05937] |
| AT3G05945 | -1,16 | 2,94E-08 | - | - |
| AT3G05955 | 2,15 | 5,70E-22 | - | - |
| AT3G05980 | -2,24 | 5,46E-58 | - | At3g05980 [Source:UniProtKB/TrEMBL;Acc:Q9SFG2] |
| AT3G05990 | 0,70 | 6,22E-22 | - | At3g05990 [Source:UniProtKB/TrEMBL;Acc:Q9SFG3] |
| AT3G05995 | 1,32 | 2,37E-04 | - | - |
| AT3G06025 | 3,77 | 6,72E-09 | - | - |
| AT3G06030 | -0,69 | 1,51E-22 | ANP3 | Mitogen-activated protein kinase kinase kinase 3 [Source:UniProtKB/Swiss-Prot;Acc:O22042] |
| AT3G06035 | -1,89 | 2,45E-189 | - | Uncharacterized protein At3g06035 [Source:UniProtKB/TrEMBL;Acc:Q0WSE0] |
| AT3G06070 | -0,61 | 4,00E-05 | - | At3g06070 [Source:UniProtKB/TrEMBL;Acc:Q8LED7] |
| AT3G06100 | -4,79 | 3,09E-03 | NIP7-1 | NLM8 [Source:UniProtKB/TrEMBL;Acc:A0A178V7E3] |
| AT3G06110 | 0,76 | 1,15E-22 | DSPTP1B | Dual specificity protein phosphatase 1B [Source:UniProtKB/Swiss-Prot;Acc:Q9M8K7] |
| AT3G06125 | -0,99 | 5,53E-15 | - | other RNA [Source:TAIR;Acc:AT3G06125] |
| AT3G06170 | 0,67 | 7,06E-20 | - | At3g06170 [Source:UniProtKB/TrEMBL;Acc:Q494Q0] |
| AT3G06260 | 2,88 | 9,03E-14 | GATL4 | Probable galacturonosyltransferase-like 4 [Source:UniProtKB/Swiss-Prot;Acc:Q9M8J2] |
| AT3G06270 | 0,67 | 1,91E-24 | - | Probable protein phosphatase 2C 35 [Source:UniProtKB/Swiss-Prot;Acc:Q7XJ53] |
| AT3G06300 | -1,24 | 5,28E-95 | P4H2 | Prolyl 4-hydroxylase 2 [Source:UniProtKB/Swiss-Prot;Acc:F4JAU3] |
| AT3G06345 | 0,98 | 4,73E-33 | - | - |
| AT3G06355 | 1,93 | 1,94E-12 | - | - |
| AT3G06370 | -1,71 | 3,11E-04 | NHX4 | NHX4 [Source:UniProtKB/TrEMBL;Acc:A0A384KPV5] |
| AT3G06380 | 1,09 | 1,17E-23 | TULP9 | Tubby-like F-box protein 9 [Source:UniProtKB/Swiss-Prot;Acc:Q9SQU1] |
| AT3G06420 | 0,77 | 9,37E-23 | ATG8H | Autophagy-related protein 8h [Source:UniProtKB/Swiss-Prot;Acc:Q8S925] |
| AT3G06430 | 0,90 | 3,13E-33 | EMB2750 | Pentatricopeptide repeat-containing protein At3g06430, chloroplastic [Source:UniProtKB/Swiss-Prot;Acc:Q9SQU6] |
| AT3G06460 | -2,39 | 1,04E-22 | - | F24P17.4 protein [Source:UniProtKB/TrEMBL;Acc:Q9SQU9] |
| AT3G06470 | -0,64 | 3,58E-07 | - | F24P17.3 protein [Source:UniProtKB/TrEMBL;Acc:Q9SQV0] |
| AT3G06495 | 1,87 | 2,02E-02 | - | - |
| AT3G06500 | -0,62 | 1,12E-31 | INVC | Alkaline/neutral invertase C, mitochondrial [Source:UniProtKB/Swiss-Prot;Acc:B9DFA8] |
| AT3G06520 | 1,18 | 2,61E-07 | - | Agenet domain-containing protein [Source:UniProtKB/TrEMBL;Acc:Q9C8Z3] |
| AT3G06550 | -0,68 | 5,56E-49 | - | O-acetyltransferase family protein [Source:TAIR;Acc:AT3G06550] |
| AT3G06570 | 1,18 | 6,06E-32 | - | F-box/kelch-repeat protein At3g06570 [Source:UniProtKB/Swiss-Prot;Acc:Q94K34] |
| AT3G06590 | -0,66 | 2,13E-24 | BHLH148 | Transcription factor bHLH148 [Source:UniProtKB/Swiss-Prot;Acc:Q9C8Z9] |
| AT3G06600 | -1,92 | 2,10E-25 | SINE3 | Protein SINE3 [Source:UniProtKB/Swiss-Prot;Acc:Q9C900] |
| AT3G06630 | -1,04 | 5,53E-19 | - | Protein kinase family protein [Source:UniProtKB/TrEMBL;Acc:A0A1I9LPU6] |
| AT3G06635 | 3,70 | 1,32E-02 | - | - |
| AT3G06640 | -2,70 | 5,33E-13 | - | PAS domain-containing protein tyrosine kinase family protein [Source:UniProtKB/TrEMBL;Acc:F4JC18] |
| AT3G06650 | -0,84 | 6,91E-104 | ACLB-1 | ACLB-1 [Source:UniProtKB/TrEMBL;Acc:A0A178V742] |
| AT3G06660 | 0,85 | 1,29E-19 | - | PAPA-1-like family protein / zinc finger (HIT type) family protein [Source:UniProtKB/TrEMBL;Acc:Q94CE6] |
| AT3G06680 | -0,62 | 6,67E-32 | - | 60S ribosomal protein L29 [Source:UniProtKB/TrEMBL;Acc:F4JC32] |
| AT3G06690 | 0,72 | 7,54E-12 | - | Putative acyl-coenzyme A oxidase At3g06690 [Source:UniProtKB/Swiss-Prot;Acc:P0CZ24] |
| AT3G06700 | -1,13 | 7,84E-160 | RPL29A | 60S ribosomal protein L29-1 [Source:UniProtKB/Swiss-Prot;Acc:Q9M7X7] |
| AT3G06710 | 1,06 | 4,68E-07 | - | E3 ubiquitin ligase [Source:UniProtKB/TrEMBL;Acc:Q6E260] |
| AT3G06740 | -1,28 | 6,89E-57 | GATA15 | GATA transcription factor 15 [Source:UniProtKB/Swiss-Prot;Acc:Q8LG10] |
| AT3G06770 | -1,26 | 4,71E-46 | - | Pectin lyase-like superfamily protein [Source:UniProtKB/TrEMBL;Acc:A0A1I9LN94] |
| AT3G06780 | 0,59 | 1,17E-03 | - | F3E22.8 protein [Source:UniProtKB/TrEMBL;Acc:Q9M7Y4] |
| AT3G06840 | -1,01 | 3,51E-07 | - | Putative uncharacterized protein [Source:UniProtKB/TrEMBL;Acc:Q8LFJ2] |
| AT3G06868 | -1,07 | 4,98E-08 | - | Vitellogenin-like protein [Source:UniProtKB/TrEMBL;Acc:Q0WM46] |
| AT3G06890 | -2,55 | 1,35E-84 | - | At3g06890 [Source:UniProtKB/TrEMBL;Acc:Q9M910] |
| AT3G06950 | 1,19 | 1,57E-17 | - | tRNA pseudouridine synthase [Source:UniProtKB/TrEMBL;Acc:A1A6J3] |
| AT3G06955 | -3,32 | 2,87E-04 | - | - |
| AT3G07010 | -1,35 | 5,41E-71 | - | Probable pectate lyase 8 [Source:UniProtKB/Swiss-Prot;Acc:Q9M8Z8] |
| AT3G07040 | 2,04 | 5,92E-08 | RPM1 | Disease resistance protein RPM1 [Source:UniProtKB/Swiss-Prot;Acc:Q39214] |
| AT3G07070 | -4,41 | 6,03E-14 | PBL26 | Probable serine/threonine-protein kinase PBL26 [Source:UniProtKB/Swiss-Prot;Acc:Q9SFT7] |
| AT3G07110 | -0,86 | 1,59E-104 | - | Ribosomal protein L13 family protein [Source:UniProtKB/TrEMBL;Acc:F4JD96] |
| AT3G07120 | -0,76 | 5,75E-07 | - | At3g07120 [Source:UniProtKB/TrEMBL;Acc:Q9SFU2] |
| AT3G07150 | 2,03 | 1,77E-04 | - | Amino acid-ligase [Source:UniProtKB/TrEMBL;Acc:Q9SFU5] |
| AT3G07170 | -0,68 | 5,16E-51 | - | AT3g07170/T1B9_17 [Source:UniProtKB/TrEMBL;Acc:Q9SFU7] |
| AT3G07195 | -3,58 | 2,46E-08 | - | RPM1-interacting protein 4 (RIN4) family protein [Source:UniProtKB/TrEMBL;Acc:A0A1I9LSZ8] |
| AT3G07255 | -6,44 | 5,58E-08 | - | Nuclear transport factor 2/RNA recognition motif protein [Source:UniProtKB/TrEMBL;Acc:A0A1I9LNF2] |
| AT3G07275 | 3,61 | 1,58E-02 | - | - |
| AT3G07310 | 1,37 | 1,20E-02 | - | F21O3.2 protein [Source:UniProtKB/TrEMBL;Acc:Q9S7A6] |
| AT3G07320 | -1,14 | 6,02E-78 | - | O-Glycosyl hydrolases family 17 protein [Source:UniProtKB/TrEMBL;Acc:Q9SRT4] |
| AT3G07330 | -1,00 | 9,20E-147 | CSLC6 | Probable xyloglucan glycosyltransferase 6 [Source:UniProtKB/Swiss-Prot;Acc:Q9SRT3] |
| AT3G07340 | -1,76 | 1,55E-63 | BHLH62 | Transcription factor bHLH62 [Source:UniProtKB/Swiss-Prot;Acc:Q9SRT2] |
| AT3G07350 | 0,77 | 1,67E-04 | - | F21O3.6 protein [Source:UniProtKB/TrEMBL;Acc:Q9SRT1] |
| AT3G07360 | 0,84 | 6,75E-26 | PUB9 | RING-type E3 ubiquitin transferase [Source:UniProtKB/TrEMBL;Acc:A0A178VJC7] |
| AT3G07425 | -2,16 | 7,12E-12 | - | Transmembrane protein [Source:UniProtKB/TrEMBL;Acc:Q3EBA9] |
| AT3G07430 | -0,93 | 7,45E-108 | YLMG1-1 | YlmG homolog protein 1-1, chloroplastic [Source:UniProtKB/Swiss-Prot;Acc:Q9SRS3] |
| AT3G07450 | 3,75 | 6,23E-03 | - | Bifunctional inhibitor/lipid-transfer protein/seed storage 2S albumin superfamily protein [Source:UniProtKB/TrEMBL;Acc:Q9SRS1] |
| AT3G07500 | 1,50 | 2,90E-06 | - | Far-red impaired responsive (FAR1) family protein [Source:UniProtKB/TrEMBL;Acc:Q84JA7] |
| AT3G07510 | -0,73 | 5,17E-05 | - | Maternal effect embryo arrest protein [Source:UniProtKB/TrEMBL;Acc:A0A1I9LQA1] |
| AT3G07520 | 0,79 | 3,59E-07 | GLR1.4 | Glutamate receptor 1.4 [Source:UniProtKB/Swiss-Prot;Acc:Q8LGN1] |
| AT3G07560 | 0,77 | 2,38E-65 | PEX13 | Peroxisomal membrane protein 13 [Source:UniProtKB/Swiss-Prot;Acc:Q9SRR0] |
| AT3G07570 | -1,33 | 6,82E-67 | - | Cytochrome b561 and DOMON domain-containing protein At3g07570 [Source:UniProtKB/Swiss-Prot;Acc:Q0WRW8] |
| AT3G07580 | -0,77 | 8,19E-09 | - | At3g07580 [Source:UniProtKB/TrEMBL;Acc:Q6NME2] |
| AT3G07600 | -1,91 | 3,78E-03 | HIPP16 | Heavy metal-associated isoprenylated plant protein 16 [Source:UniProtKB/Swiss-Prot;Acc:Q9SSF0] |
| AT3G07635 | 1,71 | 1,13E-02 | - | - |
| AT3G07650 | 1,51 | 5,55E-17 | COL9 | COL9 [Source:UniProtKB/TrEMBL;Acc:A0A384KVH3] |
| AT3G07680 | -0,76 | 6,50E-72 | - | Transmembrane emp24 domain-containing protein p24beta2 [Source:UniProtKB/Swiss-Prot;Acc:Q9S7M9] |
| AT3G07700 | 0,97 | 8,32E-44 | - | Protein kinase superfamily protein [Source:UniProtKB/TrEMBL;Acc:F4JFM1] |
| AT3G07750 | 0,64 | 3,72E-28 | - | 3'-5'-exoribonuclease family protein [Source:UniProtKB/TrEMBL;Acc:Q9FPI0] |
| AT3G07770 | 0,91 | 8,28E-186 | HSP90-6 | Heat shock protein 90-6, mitochondrial [Source:UniProtKB/Swiss-Prot;Acc:F4JFN3] |
| AT3G07800 | -1,43 | 2,47E-47 | TK1A | Thymidine kinase a [Source:UniProtKB/Swiss-Prot;Acc:Q9S750] |
| AT3G07810 | -1,00 | 3,78E-93 | - | RNA-binding (RRM/RBD/RNP motifs) family protein [Source:UniProtKB/TrEMBL;Acc:F4JFN7] |
| AT3G07860 | 0,92 | 2,24E-53 | SNRNP25 | Ubiquitin-like superfamily protein [Source:UniProtKB/TrEMBL;Acc:A0A178V8D0] |
| AT3G07900 | -2,18 | 8,96E-08 | OFUT24 | O-fucosyltransferase 24 [Source:UniProtKB/Swiss-Prot;Acc:Q9SFC4] |
| AT3G07920 | 3,64 | 3,20E-39 | - | Translation initiation factor IF2/IF5 [Source:UniProtKB/TrEMBL;Acc:F4JFQ0] |
| AT3G07930 | 0,88 | 6,31E-09 | MBD4L | Methyl-CpG-binding domain protein 4-like protein [Source:UniProtKB/Swiss-Prot;Acc:Q0IGK1] |
| AT3G07950 | -0,63 | 7,86E-43 | RBL19 | Rhomboid-like protein 19 [Source:UniProtKB/Swiss-Prot;Acc:Q8LF05] |
| AT3G07980 | -0,65 | 7,62E-15 | MAP3KE2 | MAP3K epsilon protein kinase 2 [Source:UniProtKB/Swiss-Prot;Acc:Q9SFB6] |
| AT3G07990 | -1,54 | 7,73E-113 | SCPL27 | Carboxypeptidase [Source:UniProtKB/TrEMBL;Acc:A0A178VCK8] |
| AT3G08040 | 7,01 | 1,86E-09 | DTX43 | Protein DETOXIFICATION 43 [Source:UniProtKB/Swiss-Prot;Acc:Q9SFB0] |
| AT3G08065 | 1,22 | 2,53E-35 | - | - |
| AT3G08275 | -0,65 | 2,19E-04 | - | - |
| AT3G08360 | 1,48 | 7,92E-03 | - | - |
| AT3G08410 | 5,12 | 1,78E-14 | - | - |
| AT3G08500 | 1,26 | 1,29E-02 | MYB83 | Transcription factor MYB83 [Source:UniProtKB/Swiss-Prot;Acc:Q9C6U1] |
| AT3G08520 | -1,03 | 5,98E-20 | RPL41G | 60S ribosomal protein L41 [Source:UniProtKB/Swiss-Prot;Acc:P62120] |
| AT3G08530 | -0,60 | 6,54E-58 | CHC2 | Clathrin heavy chain 2 [Source:UniProtKB/Swiss-Prot;Acc:Q0WLB5] |
| AT3G08560 | 2,19 | 3,91E-02 | VHA-E2 | VHA-E2 [Source:UniProtKB/TrEMBL;Acc:A0A178VP35] |
| AT3G08580 | -0,92 | 5,50E-169 | AAC1 | AAC1 [Source:UniProtKB/TrEMBL;Acc:A0A384KYV2] |
| AT3G08590 | 1,78 | 0,00E+00 | - | IPGAM2 [Source:UniProtKB/TrEMBL;Acc:A0A384KAQ5] |
| AT3G08630 | -0,65 | 1,96E-29 | RER2 | Protein RETICULATA-RELATED 2, chloroplastic [Source:UniProtKB/Swiss-Prot;Acc:Q9C9Z3] |
| AT3G08660 | -5,08 | 1,17E-07 | - | Putative BTB/POZ domain-containing protein At3g08660 [Source:UniProtKB/Swiss-Prot;Acc:Q9C9Z0] |
| AT3G08690 | 1,18 | 3,71E-97 | UBC11 | Ubiquitin-conjugating enzyme E2 11 [Source:UniProtKB/Swiss-Prot;Acc:P35134] |
| AT3G08730 | -1,14 | 1,73E-133 | ATPK1 | Non-specific serine/threonine protein kinase [Source:UniProtKB/TrEMBL;Acc:A0A178VLF0] |
| AT3G08740 | 0,84 | 3,31E-29 | - | Elongation factor P (EF-P) family protein [Source:UniProtKB/TrEMBL;Acc:Q8VZW6] |
| AT3G08760 | 0,61 | 4,23E-20 | ATSIK | ATSIK [Source:UniProtKB/TrEMBL;Acc:A0A384KX76] |
| AT3G08810 | 1,84 | 4,32E-02 | - | F-box/kelch-repeat protein At3g08810 [Source:UniProtKB/Swiss-Prot;Acc:Q9C9X9] |
| AT3G08840 | 0,98 | 5,64E-65 | - | D-alanine-D-alanine ligase family [Source:UniProtKB/TrEMBL;Acc:A0A1I9LPE3] |
| AT3G08880 | -1,35 | 1,83E-62 | SPC24 | Kinetochore protein SPC24 homolog [Source:UniProtKB/Swiss-Prot;Acc:Q67XT3] |
| AT3G08890 | -1,14 | 2,22E-36 | - | At3g08890 [Source:UniProtKB/TrEMBL;Acc:Q9SR89] |
| AT3G08920 | -0,79 | 2,36E-03 | STR10 | Rhodanese-like domain-containing protein 10 [Source:UniProtKB/Swiss-Prot;Acc:Q9SR92] |
| AT3G08960 | -0,61 | 3,59E-42 | - | ARM repeat superfamily protein [Source:UniProtKB/TrEMBL;Acc:F4IYK6] |
| AT3G08970 | 1,18 | 9,36E-31 | ERDJ3A | TMS1 [Source:UniProtKB/TrEMBL;Acc:A0A178VJB6] |
| AT3G08980 | -1,11 | 3,21E-34 | - | At3g08980 [Source:UniProtKB/TrEMBL;Acc:Q9S724] |
| AT3G08990 | 0,63 | 6,33E-11 | - | Protein yippee-like At3g08990 [Source:UniProtKB/Swiss-Prot;Acc:Q9SR97] |
| AT3G09005 | -1,88 | 1,30E-02 | - | - |
| AT3G09010 | 1,74 | 2,03E-08 | - | Protein kinase superfamily protein [Source:UniProtKB/TrEMBL;Acc:Q8LF75] |
| AT3G09032 | 1,02 | 9,88E-03 | - | At3g09032 [Source:UniProtKB/TrEMBL;Acc:Q6ID69] |
| AT3G09080 | -0,78 | 3,38E-12 | - | Transducin/WD40 repeat-like superfamily protein [Source:UniProtKB/TrEMBL;Acc:A0A1I9LSE8] |
| AT3G09120 | -1,12 | 3,93E-02 | - | Putative uncharacterized protein [Source:UniProtKB/TrEMBL;Acc:Q5Q0C9] |
| AT3G09160 | 4,72 | 3,33E-04 | - | RNA-binding (RRM/RBD/RNP motifs) family protein [Source:UniProtKB/TrEMBL;Acc:F4IZU8] |
| AT3G09190 | 0,61 | 4,58E-06 | - | Concanavalin A-like lectin family protein [Source:UniProtKB/TrEMBL;Acc:F4IZV1] |
| AT3G09200 | -1,16 | 1,66E-151 | RPP0B | 60S acidic ribosomal protein P0-2 [Source:UniProtKB/Swiss-Prot;Acc:Q42112] |
| AT3G09245 | -3,60 | 2,19E-02 | - | - |
| AT3G09250 | -0,92 | 2,14E-09 | - | Nuclear transport factor 2 (NTF2) family protein [Source:UniProtKB/TrEMBL;Acc:F4IZV9] |
| AT3G09270 | -1,29 | 3,88E-142 | GSTU8 | Glutathione S-transferase U8 [Source:UniProtKB/Swiss-Prot;Acc:Q9SR36] |
| AT3G09300 | -0,74 | 3,53E-61 | ORP3B | ORP3B [Source:UniProtKB/TrEMBL;Acc:A0A178VKM3] |
| AT3G09330 | -10,19 | 3,09E-22 | AVT1G | Amino acid transporter AVT1G [Source:UniProtKB/Swiss-Prot;Acc:Q1PER9] |
[truncated: 479,937 more chars]
