# Supplementary material for: Root Hair Development Is Suppressed by Long‐Term Mild Heat Through Down‐Regulation of RHD6 and RHD6‐like Genes
Source: Plant Cell Environ. 2025 Apr 18;48(8):5861–73. doi: 10.1111/pce.15563 (PMC12223709; doi:10.1111/pce.15563)
Supplement: Supplementary file 7 — Supplemental Table S2 Sequence of primers used for qRT‐PCR. [file PCE-48-5861-s007.doc]

**Supplemental Table S2 Sequence of primers used for qRT-PCR.**

| **Gene Name** | **Gene ID** | **Primer Name** | **Sequence (5' to 3')** |
| --- | --- | --- | --- |
| *UBC19* | AT3G20060 | qUBC19_F | GCCACCGGATCAAAGCAATC |
|  |  | qUBC19_R | CCGGATCAGCTCCCATCATC |
| *EIF4A3* | AT3G19760 | qEIF4A3_F | GATTCGGGCGTAAGGGTGTT |
|  |  | qEIF4A3_R | AGACACTTCCCCGCTGAAAG |
| *COBL9* | AT5G49270 | qCOBL9_F | CTGTGCCTACTCTTCAAAGCAA |
|  |  | qCOBL9_R | GCTCGGATCACGTCGTACAT |
| *EXPA7* | AT1G12560 | qEXPA7_F | CTTACAAGAGCACCGCTAATTTCC |
|  |  | qEXPA7_R | CAAAAGGCCACCGAAACTCTTAAC |
| *GL2* | AT1G79840 | qGL2_F | TGGACAAGACATGCGGGTTT |
|  |  | qGL2_R | CAAAGCATCCCACTCATGCC |
| *RHD6* | AT1G66470 | qRHD6_F | TGATTTGGTGACAATGCTTGA |
|  |  | qRHD6_R | GGAGAGAATGGCATCAATGG |
| *RSL2* | AT4G33880 | qRSL2_F | CCCCAATGGAACAAAGGTC |
|  |  | qRSL2_R | TCTCGGTGAGCTGAGACCAA |
| *RSL4* | AT1G27740 | qRSL4_F | GCCTTTATGCTCGGAAACGA |
|  |  | qRSL4_R | GCCGTTGTAAGCCAATGGTG |
